# Studying the Dynamics of a Complex G-Quadruplex System: Insights into the Comparison of MD and NMR Data

**DOI:** 10.1021/acs.jctc.2c00291

**Published:** 2022-06-06

**Authors:** Matteo Castelli, Filippo Doria, Mauro Freccero, Giorgio Colombo, Elisabetta Moroni

**Affiliations:** † Department of Chemistry, University of Pavia, V.le Taramelli 12, 27100 Pavia, Italy; ‡ Institute of Chemical Sciences and Technologies SCITEC-CNR, Via Mario Bianco, 9, 20131 Milano, Italy

## Abstract

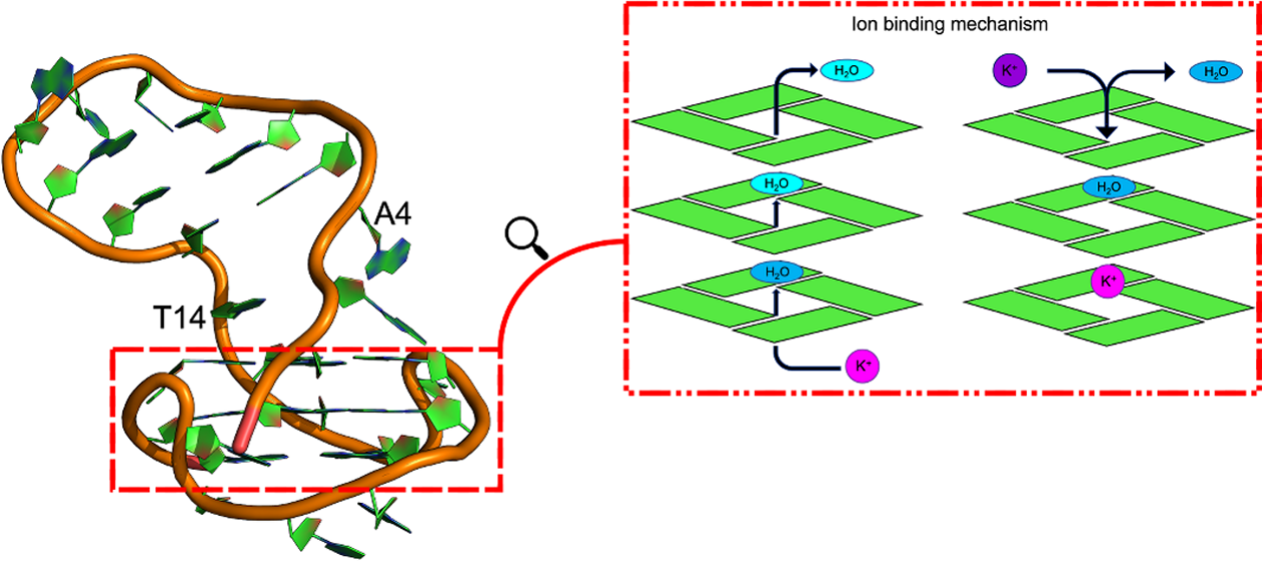

Molecular dynamics (MD) simulations are coming of age in the study of nucleic acids, including specific tertiary structures such as G-quadruplexes. While being precious for providing structural and dynamic information inaccessible to experiments at the atomistic level of resolution, MD simulations in this field may still be limited by several factors. These include the force fields used, different models for ion parameters, ionic strengths, and water models. We address various aspects of this problem by analyzing and comparing microsecond-long atomistic simulations of the G-quadruplex structure formed by the human immunodeficiency virus long terminal repeat (HIV LTR)-III sequence for which nuclear magnetic resonance (NMR) structures are available. The system is studied in different conditions, systematically varying the ionic strengths, ion numbers, and water models. We comparatively analyze the dynamic behavior of the G-quadruplex motif in various conditions and assess the ability of each simulation to satisfy the nuclear magnetic resonance (NMR)-derived experimental constraints and structural parameters. The conditions taking into account K^+^-ions to neutralize the system charge, mimicking the intracellular ionic strength, and using the four-atom water model are found to be the best in reproducing the experimental NMR constraints and data. Our analysis also reveals that in all of the simulated environments residues belonging to the duplex moiety of HIV LTR-III exhibit the highest flexibility.

## Introduction

Guanine-rich DNA sequences can form high-order noncanonical secondary structures, called G-quadruplexes (G4s). G4s represent an expansion of the possible three-dimensional (3D) arrangements of DNA besides the classical right-handed double helix arrangement. In these assemblies, four square planar guanines self-associate together via eight Hoogsteen-type hydrogen bonds, defining a G-tetrad.^1^ Two or more tetrads can stack on top of one another forming G4 structural motifs, further stabilized by interactions with metal cations (such as Na^+^ and K^+^) at the central cavity.^2,3^ Guanine repeats are connected by short variable sequences, which link the G-tetrads and form the loops, dictating the overall G4 topology as a function of their length and composition. Together with the above-mentioned features, further differentiating characters (topologies, strand stoichiometry, and groove conformation) outline an extremely diversified G4 structural landscape.^4^

G4s have been widely described and mapped in the human genome observing a nonrandom distribution.^5,6^ A general enrichment of putative G4 sequences (PQSs) is observed in genome regions such as telomeres,^7^ gene promoters,^8^ DNA replication origins,^9^ and untranslated regions (UTRs).^10^ These regions are associated with crucial functions such as replication, transcription, repair, gene expression, epigenetic regulation, and genome stability.^7,11^ Furthermore, G4s appear to be involved in several human diseases, including cancer,^12^ infections, and neurodegenerative pathologies.^13^ A considerable presence of PQS was observed not only in the human genome but also in other mammalians,^14^ bacteria,^15,16^ protozoa,^17^ yeasts,^18^ and viral genomes.^19^ Recently, G4 localization in the viral genome has attracted particular attention, as emerging evidence suggests that the role of G4s could be implicated in several crucial processes in the viral life cycle such as the regulation of replication and transcriptional steps.^20^

The formation of G4s has also been reported in the unique long terminal repeat (LTR) promoter region in the genome of the human immunodeficiency virus 1 (HIV-1).^21^ Folding/unfolding of the LTR region into G4 conformations through a complex interplay between viral regulatory proteins and cellular proteins that interact with it has been shown to regulate transcriptional activity in HIV-1:^22,23^ stabilization by G4 ligands represses viral transcription initiation, while cellular transcription factors modulate genome transcription by unfolding LTR G4.^21,24−26^

The sequence of the LTR promoter is characterized by three mutually exclusive G4-forming components, namely, LTR II, LTR-III, and LTR IV.^22,27^ Interestingly, cellular proteins nucleolin and hnRNP A2/B1, which bind LTR G4 structures and repress/activate viral transcription, respectively, do not affect the activity of promoters carrying mutations that completely or partially abolish LTR-III G4 formation compared to the wild-type sequence.^25,26^ These results suggest that LTR-III G4 has a key role in the regulatory events of HIV-1 transcription. Therefore, the identification of ligands able to selectively target the LTR-III G4 conformation may represent an effective strategy to inhibit virus replication.^28^

So far, the design of G4-binding molecules has been guided by considering π–π stacking and electrostatics interactions as the driving forces required for G4 stabilization by ligand binding. Empirical approaches have been applied as well. However, very few compounds have been shown to recognize G4 structures in a selective way.^29,30^ Despite numerous efforts, G4 ligand selectivity was obtained only over other DNA secondary structures (e.g., B-DNA vs G4)^31^ and over different G4 topologies.^32,33^ In general, ligand selectivity with respect to a particular G4 structure over other G4s has not been achieved yet, and neither has ligand selectivity toward LTR-III G4 over other G4 structures.

Structure-based drug design of G4-interacting compounds is indeed challenging: structural studies have revealed that they are highly polymorphic and dynamic structures, in agreement with biophysical studies in solutions.^34−36^ This makes it hard to define a clear structural target as a starting point for structure-based design. Rather, the intrinsically dynamic nature of G4s suggests that ligand design cannot rely on a single structure, but conformational diversity should be taken explicitly into account to effectively design specific binders.

Classical molecular dynamics (MD) simulation is the computational method of choice to study the dynamics behavior of biomolecules and the way they interact, providing detailed atomic structural dynamics information occurring on the microsecond time scale that cannot be accessed by experimental measurements. Numerous studies employed classical MD simulations and enhanced sampling techniques to characterize intermediates of G4 folding to study kinetic partitioning, metastable states, and ligand binding.^37−43^ However, simulation studies have their limitations: in particular, it is well known that the results are influenced by the capability of the force field to properly model the forces between atoms in these systems. Several studies have demonstrated that force fields used to simulate G4s still present some limitations, in particular, with regard to G4 folding/unfolding events, where large conformational changes occur.^44−49^

On the other hand, simulation studies of fully folded G4 structures established from experiments are less affected by these imbalances, reporting excellent performance of the force fields. This is probably due to the stiffness and long lifetime of G4 conformations observed in folding experiments,^50−54^ which extends beyond the simulation time scale. In other words, in these simulations, the systems undergo thermal fluctuations, exploring the basin of possible conformations near the starting structures without undergoing large structural transitions because of the high-energy barriers, which characterize the free-energy landscape of G4s.^48^

Using ensembles of conformational substrates visited during MD simulations around the folded state of G4 elements can thus prove beneficial to structure-based ligand design, accounting for (subtle) differences in the shapes of potential binding sites, such as cation distribution, backbone and loop conformations, and H-bond formation or disruption.

In this context, a series of benchmark studies on G4 simulations analyzed the performance of force fields and ions and water parameterizations.^49,55−58^ The majority of G4 simulations in the literature were performed with various versions of the AMBER nucleic acid force fields and the CHARMM force fields. The introduction of polarizable force fields (e.g., the Drude force field^59,60^) underscored the importance of electronic polarization as an important determinant of nucleic acid structures and their dynamics. When simulating noncanonical DNA structures, polarizable force fields lead to higher stability, with respect to more frequently used force fields (e.g., OL15^61^ and Parmbsc1^62^). A possible limitation of polarizable force fields may be represented by the onset of over-polarization effects. To date, the definition of the most suitable general use set of classical simulation parameters for canonical and noncanonical DNA structures still remains an open question.^55,63,64^ A recent study by Li et al. provides a comprehensive view on this issue.^65^ Different ion parameters and water models have also been evaluated. In particular, many recent simulations make use of the TIP3P and TIP4P-Ew water models in combination with Amber-adapted Åqvist^66^ and Joung and Cheatham parameters for ions,^67^ which improve the bulk behavior of ions in water solution. Moreover, salt concentration seems to affect structure stability: in particular, high salt concentration increases atomic fluctuations leading to unusual conformations of G4s.^58^

It is clear from the literature that G4 MD simulations are sensitive to different simulation setups. Besides the force field used, the ion parameters, water models, and ionic strengths can influence the outcome of simulations. Parmbsc0 has been largely tested by several groups,^58^ while the performance of its evolution, namely, Parmbsc1, with regard to the use of different water and ion parameters has not been investigated extensively.

**Figure 1:**
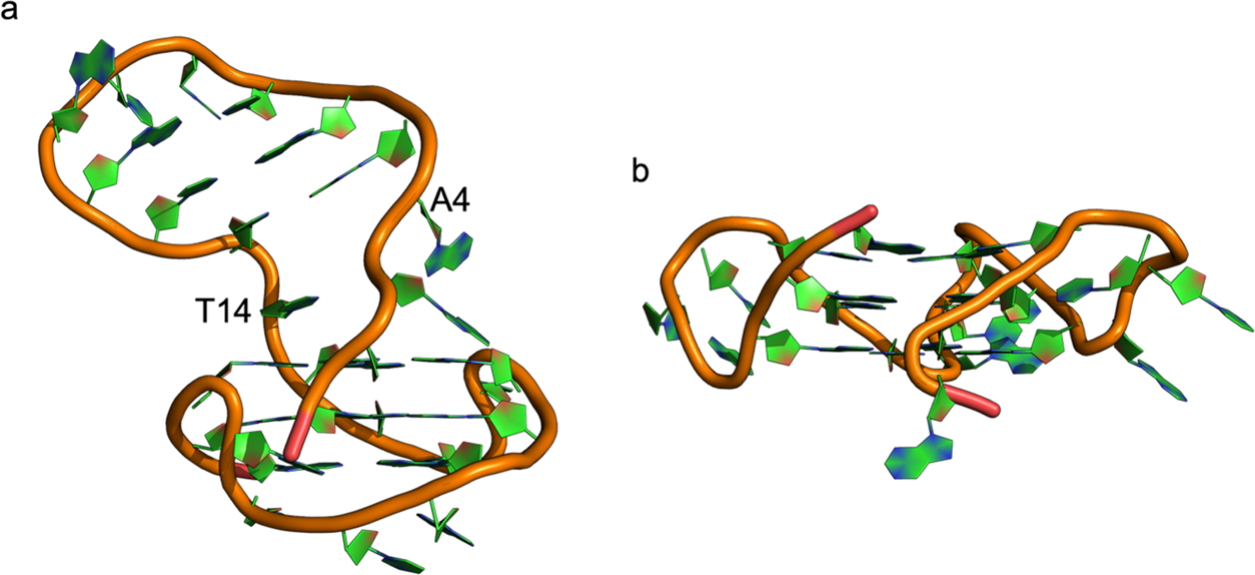
Comparison of HIV LTR-III (a) with minimal G-quadruplex molecular structure (b) (PDB: 3CDM) to provide a pictorial view of the complexity of LTR-III compared to simpler, classical G4 structures.

Here, we set out to analyze how different combinations of water models and ion concentrations in MD simulations can influence the structural stability, the dynamic states, and the interactions with ions in a complex G4 system such as HIV LTR-III (see Figure 1a). To this end, we use the AMBER force field Parmbsc1^62^ with the Joung and Cheatham^67^ parameters to model counterions and different water models (TIP3P and TIP4P-Ew). While most previous analyses and benchmark studies were conducted on minimal G4s comprising only short loops bridging the tetrads, here we focus on a complex system, whose structure was solved in solution via nuclear magnetic resonance (NMR) spectroscopy (Protein Data Bank (PDB) id: 6H1K, consisting of an ensemble of 10 G4 conformations). To provide a pictorial view of the complexity of LTR-III compared to simpler, classical G4 structures studied previously, we also report in Figure 1b a minimal G-quadruplex molecular structure.^22^ HIV LTR-III G4 has unique structural features such as a 12-nucleotide diagonal loop containing a conserved duplex-stem linked to the 12-nucleotide quadruplex through a peculiar 2-nucleotide junction. To the best of our knowledge, this work represents the first example of a benchmark study on a structure where both quadruplex and duplex structures are simultaneously present.

The results of the study of the stability and dynamics of HIV LTR-III in different conditions, consisting in a total of 12 μs of all-atom simulations, are then compared with experimentally derived NMR structural data. We focus on the 433 nuclear Overhauser effect (NOE) restraints available from the Protein Data Bank (code 6H1K).^22^ We analyze various aspects of structural dynamics for this biomolecule from the stability of hydrogen-bonding (H-bonding) patterns to the distribution of torsional angles obtainable from the simulations compared to the data available from the NMR bundle. Specifically, we evaluate the number of NOE restraints that are violated under the different simulation conditions used. In particular, sets of different structures are shown to satisfy NMR-derived distance restraints and a possible protocol for the simulation of these types of systems is proposed. Indeed, small differences in water models and ion concentrations influence the interaction of nucleic acids with the solvent, and the effect of these local interactions can spread over a large portion of the DNA.

We discuss how dynamic structural changes and their relationships to experimental data indeed are critical in characterizing ensembles of conformations as targets for subsequent ligand design.

## Results and Discussion

As mentioned above, the starting point for our MD simulations is the NMR-derived HIV-1 LTR-III (PDB id: 6H1K) structure.

LTR-III is a 28-nucleotide sequence d[GGGAGGCGTGGCCTGGGCGGGACTGGGG] that folds into an intramolecular G4 structure and exhibits peculiar structural features. In particular, LTR-III consists of a 1-nt propeller loop (C18), a V-shaped loop (G25 and G26), a 3-nt lateral loop (from A22 to T24), and a 12-nt diagonal loop (from G3 to T14) containing a conserved duplex-stem. The quadruplex–duplex junction is composed of two principal residues (A4 and T14) that play an important role in the duplex stability. It has been observed that A4 and T14 are able to flip in and out with respect to the central axis of DNA molecules.^22^

The unique structural features of this junction make its targeting an attractive strategy for the inhibition of viral transcription: in this context, one may envisage exploiting the information provided here to design (stabilizing) ligands able to simultaneously engage both the G4 and duplex moieties.

Here, we report and discuss the results of the comparison between different MD simulation settings.

### Molecular Dynamics Simulations

We simulated LTR-III in four different solvent environments in explicit water, with the force field Amber Parmbsc1^62^ and the Joung and Cheatham^67^ ion parameters. Potassium counterions were used to neutralize the charge of the systems.

In the following, we name the four simulated environments **K-TIP3P**, **KCl-TIP3P**, **K-TIP4P**, and **KCl-TIP4P**, where “K” indicates the use of K^+^-ions to neutralize the system charge and “KCl” indicates the presence of KCl. The latter is added to reach a concentration value of 100 mM to fully reproduce experimental conditions used for structure resolution,^22^ while TIP3P^68^ and TIP4P-Ew^69^ are the three/four-atom water models used in MD simulations, respectively. It is to be underlined that the term TIP4P in the labels is used for brevity. The actual model used in these simulations is TIP4P-Ew.^69^ TIP3P describes canonical water molecules, while TIP4P-Ew adds a virtual site and features improved Lennard-Jones, charge, and virtual site parameters (relative to the original TIP4P^70^) to improve the electrostatics around the oxygen atom and permit an optimal use with Ewald electrostatic schemes.^69^ Three independent replicates were carried out (1 μs in time length) for each solvent environment for a total of 12 μs of simulation time.

To investigate the effects of the water models and ion concentrations on the LTR-III structure, we performed several structural and dynamic analyses on the MD trajectories using as reference the 10 NMR conformations of LTR-III (PDB id: 6H1K).

### Modeling K^+^ Diffusion and Water Distribution around LTR-III

From the physical-chemistry point of view, the stability of G4 structures depends on a subtle balance of different factors: stacking interactions, hydrogen bonding, solvation, and cation binding.

It has been widely demonstrated that cation coordination stabilizes the G-tetrad stacks, while ionic strength is required to compensate for electrostatic repulsion between the phosphate oxygens of four strands in G4s.

Molecular dynamics studies have shown that G4s with coordinated alkali metal cations are very stable, while they become highly unstable without any coordinated cations in the central anionic cavity, confirming that ions are integral components of G4 molecules.

Starting from the structure of LTR-III without any coordinated ions, surrounded by explicit water molecules and randomly placed K^+^ counterions, with or without KCl salt to reach a concentration value of 100 mM, in all simulated environments, we observed the diffusion of ions from the solvent into the central channel; the ions are then captured and coordinated by the guanines forming the tetrads via their O6 oxygen. In this context, we calculated an estimate of the free energy of ion binding to the G4 structure from our trajectories: we calculated the number of bound vs unbound states and on this basis estimated an equilibrium constant, which was eventually translated into a Gibbs free-energy difference. The results of the calculation show that in all solvent environments ion binding is favored, with DGs ranging from −1.01 kJ/mol in simulation KCl-TIP3P to −2.90 kJ/mol in simulation KCl-TIP4P. The full details of the calculation and the raw data are reported in the Supporting Information Free Energy Estimation section.

To analyze the possible mechanisms of K^+^-ion entrance in the G-quadruplex channel, we monitored the distances between the O6 atoms of the G4 guanine bases and the potassium ions (see the Supporting Information, Figures S1–S12, reporting on all of the time-dependent distances between K^+^-ions and each O6 atom in the G4s).

**Figure 2:**
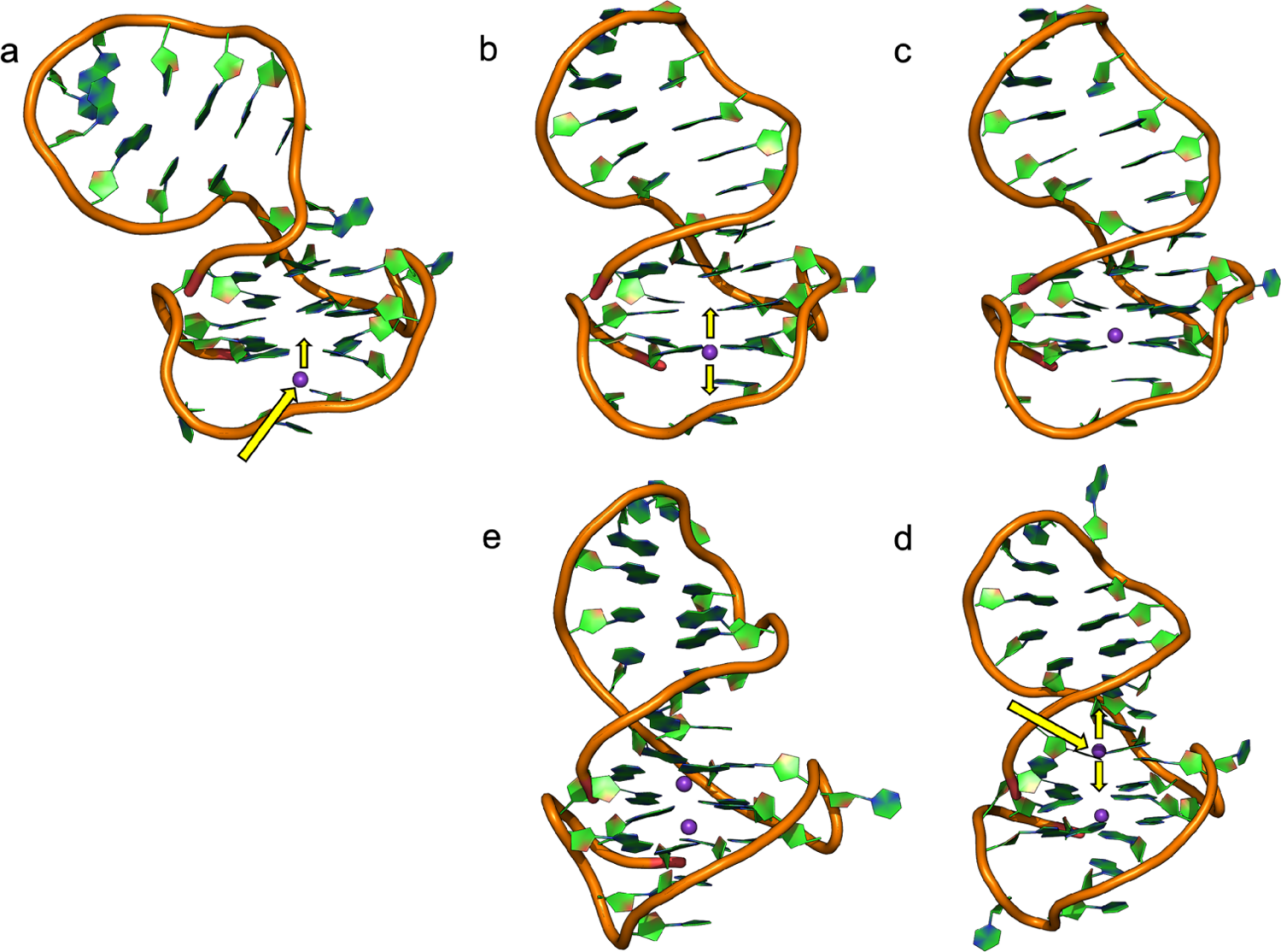
Schematic representation of a generalized inclusive mechanism of K^+^ in the G4 central cavity. Representative frames derive from KCl-TIP4P replica-2. (a) First approach of K^+^ to G4 (at 3 ns), (b) temporary placement of K^+^ (at 4.18 ns), (c) stable positioning of K^+^ between two tetrad planes (at 4.19 ns), (d) approach of a second K^+^ (at 18.1 ns), and (e) stable arrangement with two included K^+^ in the central cavity (at 91.28 ns).

In Figure 2, we exemplify a consistent general mechanism through which K^+^-ions can enter the G-quadruplex’s central cavity. In panel (a), the ion approaches the G4 in the absence of other coordinated ions; at the same time, G4 undergoes a conformational rearrangement deviating from NMR structures. Subsequently, the ion temporarily oscillates between the position in panel (a) and the position in panel (b) (i.e., in the plane of the tetrad), before eventually crossing the plane of the tetrad to reach the cavity and octa-coordination (c). Once these interactions are established, the ion position is fixed till the end of simulation; we never observed the ion exit from the cavity. As the second ion approaches the G4 (d), we observe similar structural rearrangements as for the first ion. Also in this framework, it is possible to observe switching between the position in panel (d) and in the plane of the tetrad, as between panels (a) and (b), before converging to the octa-coordinated position (e).

To gain insight into the K^+^ distribution and their interaction with LTR-III along the simulation, we calculated the radial distribution function (RDF), which describes how K^+^ density varies as a function of distance from a reference atom, and the spatial distribution function (SDF), which determines a three-dimensional density distribution of K^+^ around LTR-III.

RDFs were calculated between the O6 atoms of the G4-guanines (O6-K^+^), the OP2 atoms of the sugar-phosphate moieties constituting the backbone (OP2-K^+^), and the K^+^ counterions. RDFs were calculated as the RDF of the K^+^ with respect to the center of mass of the selected solute atoms (for details, see the Materials and Methods section).

O6-K^+^ RDFs show similar results over the four different simulation sets (see Figure S13, Supporting Information); a first peak at 2 Å represents the two K^+^-ions entered in the G4 central cavity. In KCl-TIP4P and K-TIP3P, it is possible to observe a slightly higher peak at 2 Å compared to all of the other simulation setups, indicating a stable distribution of K^+^ in the central cavity during the simulation time. Given that for two ions in the G4 cavity along the entire trajectory the integral must be equal to two, the integrals (Figure S13, red line) describe a stable positioning of K^+^-ions.

OP2-K^+^ RDFs exhibit a more variable positioning of K^+^ with respect to the backbone (see Figure S14, Supporting Information). In the absence of added ionic strengths (K-TIP3P and K-TIP4P), we identified three principal peaks at around 2.5 Å, between 5.0 and 7.0 Å and between 7.0 and 10.0 Å. For distances greater than 10 Å, we observed a broadened peak. To better dissect the distribution of K^+^ around the backbone, we split up the RDF analysis considering separately the OP2 atoms of residues of the duplex (OP2-duplex-K^+^) and of the quadruplex (OP2-G4-K^+^) moieties and the counterions (see Figures S15 and S16, Supporting Information). It is worth noting here that the duplex does not undergo large conformational changes or major swaying/twisting motions. Concerning OP2-duplex-K^+^, at short distances at short distances (5 < *d* < 10 Å), no significant differences over the four different simulation setups were observed. The major difference between OP2-duplex-K^+^ and OP2-G4-K^+^ is observed for distances >10 Å.

In this context, the calculation of RDF distributions calls for a word of caution. RDF profiles have been obtained by counting the number of ions *n*(*r*) in a thin spherical shell around the center of mass of the reference group of atoms of the solute as a function of the distance *r*, normalized by the expected number of particles at that distance, *n*_exp_ = ρ exp *V*_exp_(*r*) = ρ_exp_*4π*r*^2^ d*r*, and averaged over each system configuration generated through MD simulations.

This scheme can be optimal to evaluate the RDF in the case of a single atomic solute or for proteins whose shape can be approximated by a sphere. However, this approach has limitations for irregularly shaped structures such as our duplex–quadruplex system. In particular, two factors can affect the RDF profiles, especially near the solute surface, (i) the shape of the solute and (ii) its shape variation during MD simulations. As for the latter, MD simulation allows the movement of all atoms in the system, which may eventually result in the modification of the geometric parameters used for determining the spherical shells for each configuration. In our case, we did not observe large variations of the positions of the reference atoms of the solute, which can affect the significance of the average, supporting the qualitative validity of the calculation described above. With regard to the shape of the solute, the distribution pattern of ions is dependent on this shape and an important factor affecting the RDF profiles using a spherical shell scheme is the volume occupied by the solvent. This scheme indeed underestimates the density of ions at the same distance from the surface of the duplex–quadruplex, as the presence of the solute restricts the volume occupied by the ions near the solute surface and alters the normalization factor, as compared to the density observed in the bulk. The inclusion of the excluded volume correction would not impact the position of the maxima and minima of the RDF profile, but instead the peaks would be relatively lower compared to those found using the spherical shell scheme. Schemes to deal with irregularly shaped solutes have been proposed,^71^ which entail modeling atoms within a solute by overlapping spheres to construct a molecular domain. The volume of the molecular domain (solute) is then calculated by numerical integration via the union of spheres and is used to calculate the volumes of solvation shells. This scheme was shown to return correct distributions for simple irregularly shaped systems made up of a low number of atoms. However, its application to large solutes over microsecond-long simulations may turn out to be too computationally involved for routine applications. Alternatively, approximations of solutes (in particular, proteins) with ellipsoidal shapes, and corrections based on this approximation, also appeared:^72^ this shape approximation, however, may not be valid in systems such as the one we are presenting here.

In general, calculated RDFs as presented here provide an overall qualitatively reliable representation of the distribution of ions in solution. Furthermore, as discussed at multiple points throughout the paper, our simulations provide a chemically sensitive picture of the mechanisms of ion diffusion and complexation by G4 nucleic bases.

RDFs alone cannot give a complete picture of the three-dimensional distribution of the ions around the molecule. Consequently, we set out to run an SDF analysis for K^+^-ions.

**Figure 3:**
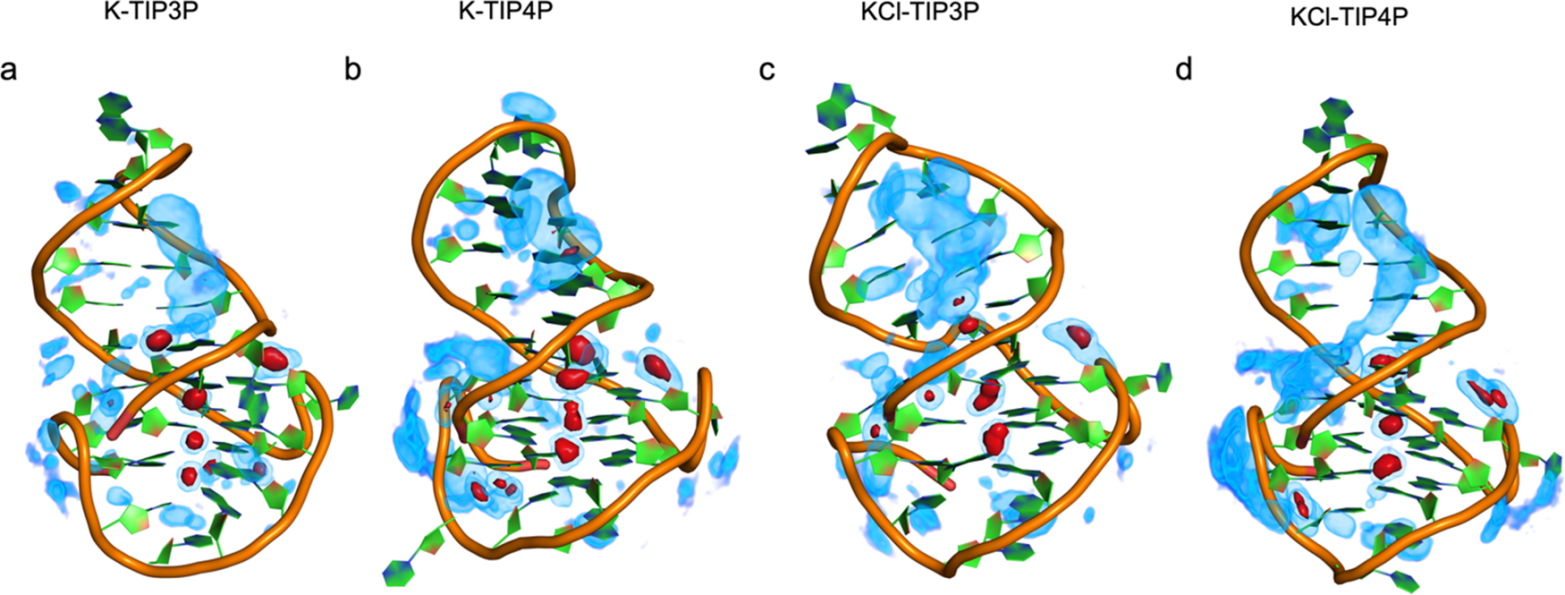
SDFs of K^+^-ions around the most populated cluster for each simulation environment (for detail, see Figure 7). Ion distribution is calculated through a 3D grid and normalized by density (default particle density for water based on 1.0 g/mL). Higher values represent a more stable presence of K^+^-ions. Regions in red represent values larger than 1, while blue regions are referred to values between 0 and 1.

SDF analysis was carried out to determine the spatial distribution of K^+^ around the LTR-III structure. This analysis, together with RDF data, allows us to decipher the differences between the four simulation setups (Figure 3). In all four systems, the highest ion density is observed in the central cavity of the G4 and in neighboring regions, consistent with RDF calculations.

To analyze the effect of water models (TIP3P and TIP4P-Ew) on K^+^ distribution, we compared **K(Cl)-TIP3P** with **K(Cl)-TIP4P**. In **K(Cl)-TIP4P**, we observed a slightly higher density of K^+^-ions in the grooves compared to **K(Cl)-TIP3P**. Moreover, in **K(Cl)-TIP4P**, the ion distribution in the groove is more continuous compared to **K(Cl)-TIP3P**.

The effects of ionic strengths were addressed by comparing **K-TIP3(4)P** with **KCl-TIP3(4)P**. In **KCl-TIP3(4)P**, we noted a higher density of K^+^-ions in the grooves with respect to **K-TIP3(4)P**.

Both ionic strength and TIP4P-Ew water model increase the K^+^-ion affinity for the DNA backbone, in particular, for the groove regions, bringing a homogeneous distribution of potassium ions.

To understand the local interactions between water and solute, we next analyzed preferential water distributions through SDF calculations. In general, the analysis of persistence of water around the DNA shows water density in the grooves, tracing the phosphate backbone (see Figure S17, Supporting Information). In the presence of larger concentrations of ions (KCl-TIP3P and KCl-TIP4P), the water molecules are displaced by the metals: the latter outcompetes them, establishing interactions with the backbone, consistent with what we observed in the K^+^ SDF calculations.

**Figure 4:**
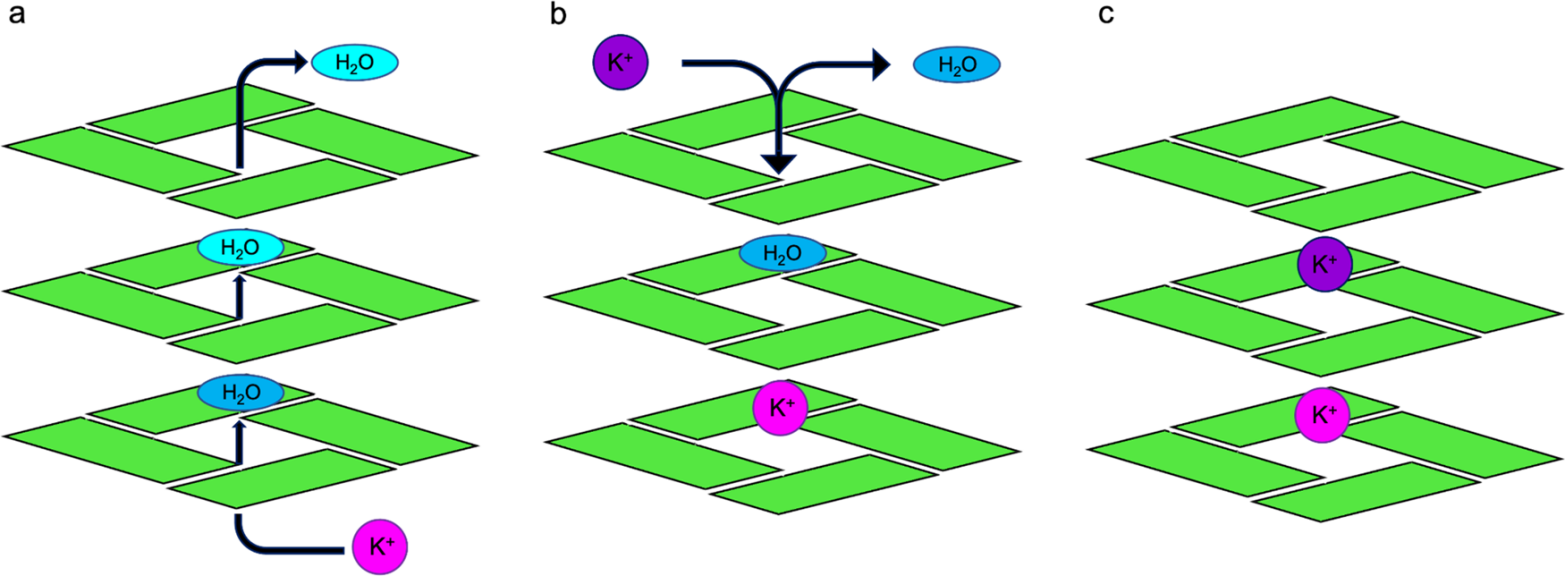
Simplified scheme of water exclusion from the G4 cavity due to the entry of ions. (a) The first approach of K^+^ with the subsequent water exit from the G4 channel; (b) approach of the second K^+^ from the opposite side of the G4; exclusion of the second water molecule; and (c) stable arrangement with two included K^+^ in the central cavity.

It is worth noting that SDF calculations detect water molecules in the G4 cavity. Consequently, we analyzed the effects of solvation over K^+^ diffusion. Previously, we focused on G4 residues evaluating the mechanisms of ion penetration in the G4 planes. In this context, we observed that initially waters occupy the cavities in the DNA structure between two tetrad planes (as seen for metal ions). Water molecules are then displaced by the entry of K^+^-ions that end up being complexed by the oxygen atoms of the tetrad. In Figure 4, we provide a schematic explanation of the mechanism based on that described in Figure 2. First, the approach and the following penetration of one K^+^-ion displace one water molecule from its initial placement. The water molecule, in turn, moves through the G4 cavity pushing out another water molecule, generating a sequential model of ion entry and reorganization of water molecules within the DNA structure (Figure 4a). Interestingly, if a K^+^ is already present in one layer, the second one will enter from the opposite face of the other layer, forcing a water molecule to get out (Figure 4b). Furthermore, we noted that initially water occupies the pocket in the DNA structure present when the junction is open. It should be noted that our simulations start from an open state of the junction, which converges quickly to a closed conformation and stabilizes until the end of the simulations. The initial water presence excludes that the junction closure is due to an empty space between the nucleobases.

### Structural Evolution of LTR-III

**Figure 5:**
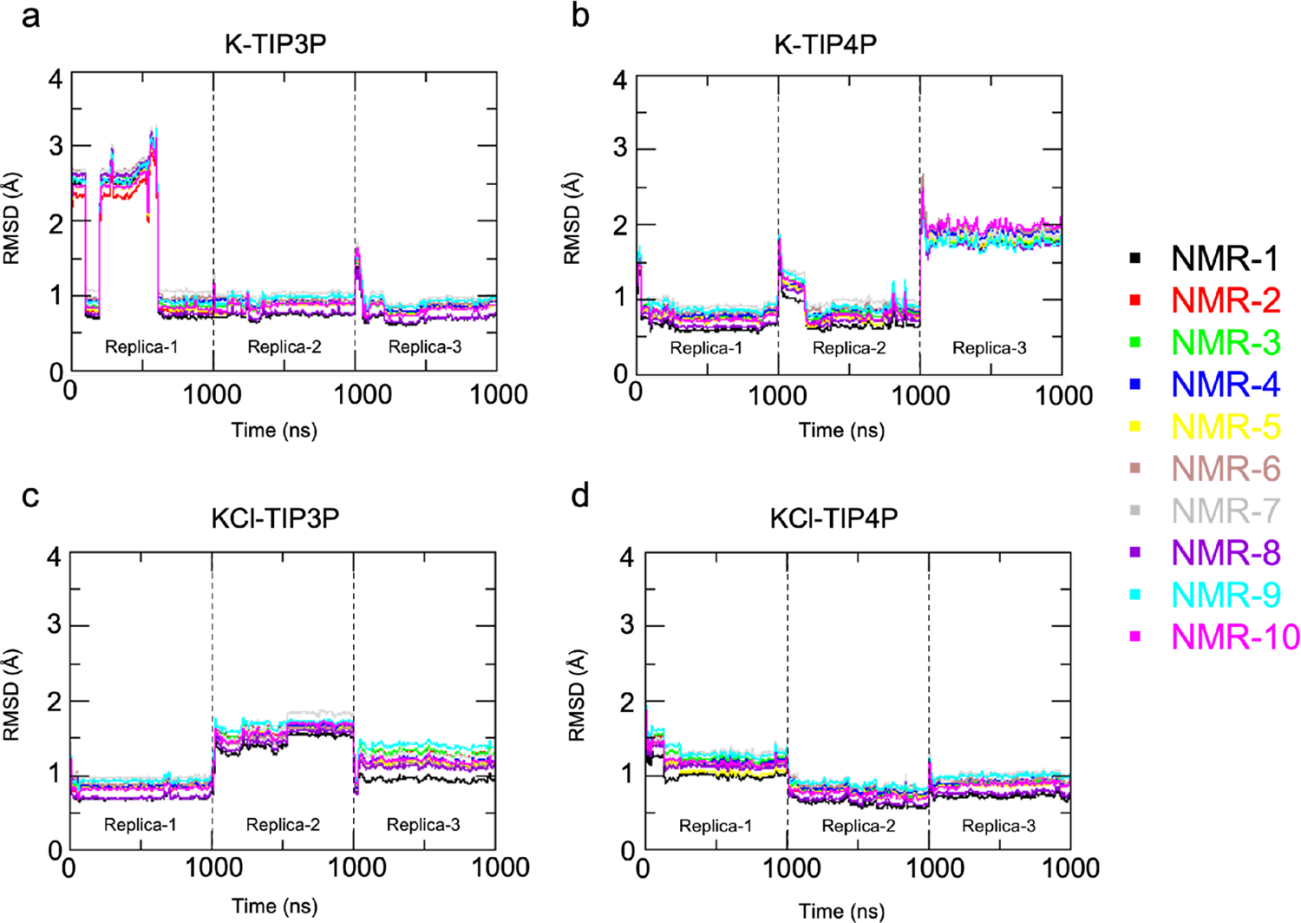
Time evolution of RMSD of the G4 nucleobase atomic position from the 10 structures of NMR bundle during simulation with different solvent environments: (a) K-TIP3P, (b) K-TIP4P, (c) KCl-TIP3P, and (d) KCl-TIP4P.

In all of the simulated systems, we observed a stabilizing effect of ion coordination on the LTR-III structure, as shown by the time evolution of the RMSDs, calculated with respect to the 10 NMR structures along the entire trajectories (Figure 5).

To discern the different contributions to RMSD from the different structural elements of LTR-III, we split the all-atom RMSD calculation (see Figure S18, Supporting Information) into different parts: over the entire backbone (RMSD-BB) over the duplex moiety backbone (from residue 5 to residue 13, RMSD-duplex-BB) and over the G-quadruplex guanine bases (RMSD-G4).

Inspection of RMSD-G4 trends in **K-TIP3P** (Figure 5a) provides insight into the conformational variations occurring during the simulations: the RMSD value increases to a maximum value of almost 4 Å and then it decreases to a stable value of about 0.75 Å. From the structural standpoint, in the conformational ensemble with large RMSD, one K^+^-ion interacts with the O6 atoms of the G-17-21-25-28 tetrad without entering the cavity, leaving the nucleic acid free to fluctuate. Next, as a K^+^-ion enters the central anionic cavity of the G4, a structural change is observed, whereby G4 samples conformations more similar to the NMR-derived ones, as shown by the drop of G4-RMSDs to ≈0.75 Å.

A similar behavior is observed in K-TIP4P (Figure 5b), where it is possible to distinguish both the entrance of the first K^+^-ion (after ≈10 ps) and the second K^+^-ion (after ≈200 ps), which causes the drop of the RMSD values. As for KCl-TIP3P (Figure 5c) and KCl-TIP4P (Figure 5d), already at the beginning of the simulations, we observed the entry of two K^+^-ions in the central cavity of the G4 moiety and the consequent stabilization of the G4-RMSD value of around 0.75 Å. In all four simulation setups, the 10 RMSD-G4 trends are very similar between them, and once the ions occupy the central cavity, the RMSD-G4 values settle at a value of around 0.75 Å. These observations are in agreement with the NMR bundle where the 10 structures show a very similar G4 moiety. The very low RMSD-G4 values indicate that the quadruplex moiety is well represented in all four simulation setups.

Concerning RMSD-BB, it is possible to note a wide differentiation among the 10 RMSD trends with values starting from 4 Å to a maximum of 9 Å (see Figure S19, Supporting Information). In this context, we observed a general trend over the four solvent systems where the NMR-6 (brown) shows the lowest RMSD values. NMR-3 (green) and NMR-1 (black) show slightly major values with respect to NMR-6 (brown). The increase in the RMSD value can be reconnected to the structural changes observed in the junction and top loop regions (see also root-mean-square fluctuation (RMSF) and NOE violation calculations), where the highest flexibility and tendency to conformational transitions are observed.

As for RMSD-duplex-BB, the 10 RMSD trends consistently show slightly higher values compared to the rather rigid G4 region. RMSD values for the duplex backbone vary from around 2 Å to around 4 Å, with observed trends overall very similar among the various simulations (see Figure S20, Supporting Information). This is in agreement with what is observed in the NMR bundle, where the duplex moiety populates different structures within a limited conformational ensemble: indeed, we observe that no major twisting or swaying of the duplex can be observed during the simulations. In this framework, no relevant differences between the four solvent environments are observed.

### Evaluation of Structural Flexibility and Definition of the Major Conformational Families

Next, we calculated the root-mean-square fluctuation (RMSF), averaging the atomic fluctuations per residue with respect to the average structure as measures of the structure’s flexibility.

**Figure 6:**
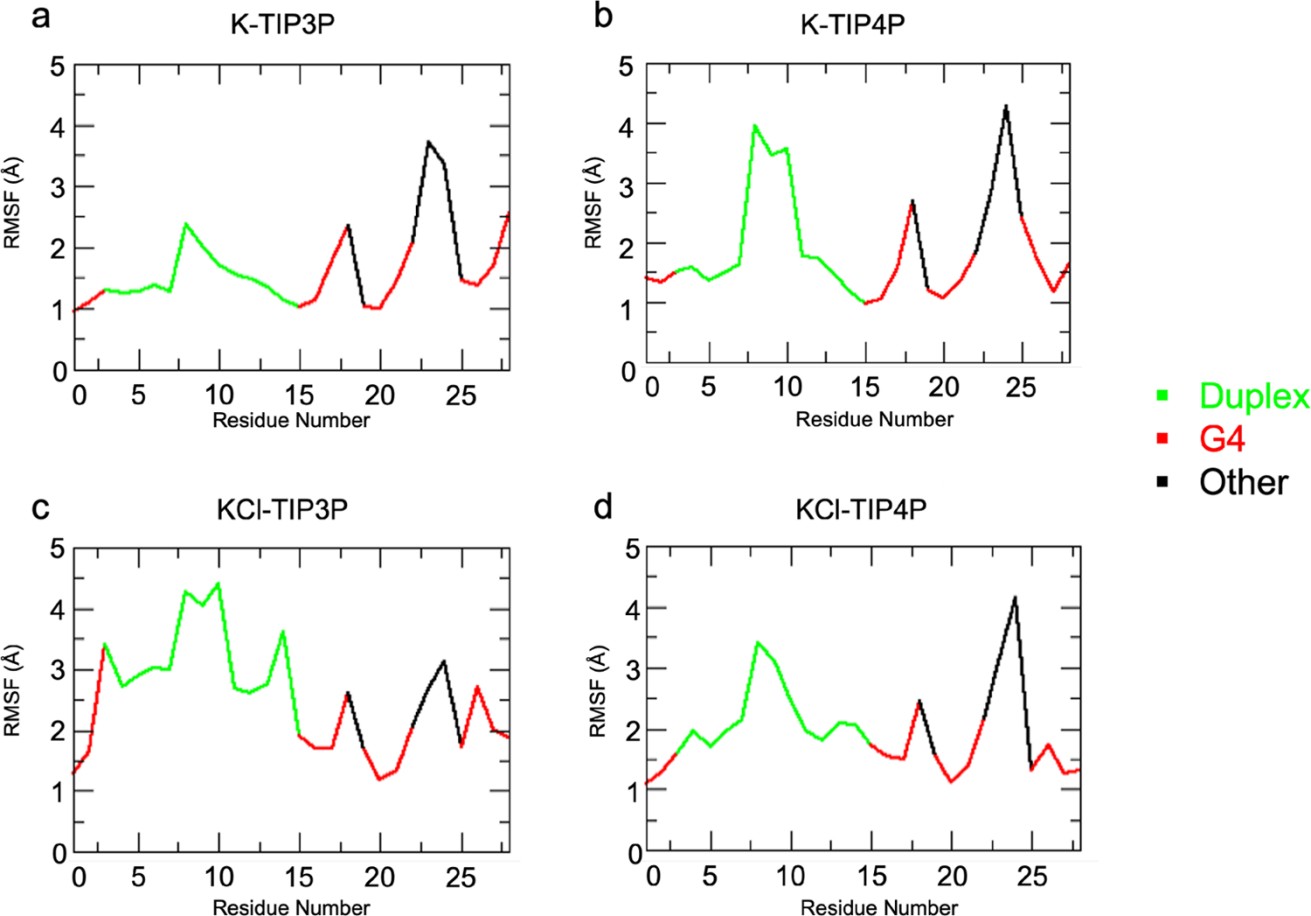
RMSFs of all atomic positions calculated with respect to the averaged structure of each system. Duplex residues are reported in green, G4 in red, and the other in black.

This analysis reveals that in all of the simulated environments, residues belonging to the duplex moiety (especially, residues 8–10) and residues exposed to the solvent (residues 18, 22–24) undergo the highest fluctuations (Figure 6). However, ionic strength seems to influence the flexibility of the duplex moiety, which shows slightly higher fluctuations in the **KCl-TIP3P** and **KCl-TIP4P** environments than in **K-TIP3** and **K-TIP4P**.

**Figure 7:**
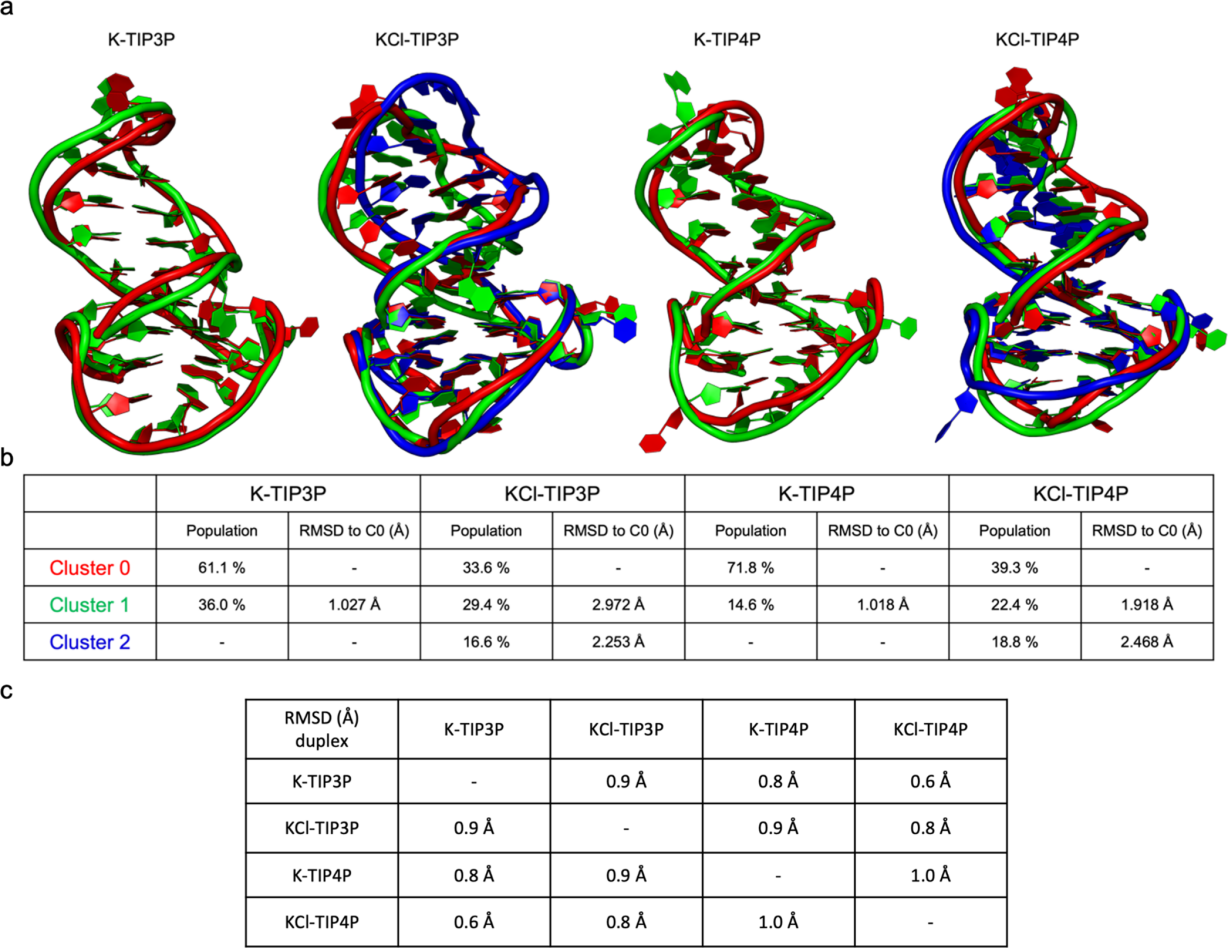
Clustering calculations were carried out to highlight duplex twisting. Trajectories were aligned over the G4, and clustering was focused on the duplex. (a) Different conformations are superimposed to reach 80% representativeness of the system. (b) All-atom root-mean-square deviations (RMSDs) were calculated with respect to the most populated conformation of each environment. (c) Duplex RMSDs were calculated with respect to the most populated conformation of each environment.

To gain further insights into the duplex flexibility, we carried out clustering calculations over the trajectories aligned on the G4 moieties. Then, the duplex’s residues are considered to define the different conformational families (for detail, see the Materials and Methods section). This analysis allows the identification of the potential flexibility of the duplex. To quantify the structural diversity explored during simulations, for each solvent environment, we calculated the duplex RMSD between the most representative conformation and the other conformations in the trajectory until the 80% representativeness is reached. We also highlighted the solvent environmental influence on duplex conformations, comparing the most populated cluster of each simulation setup. The results show that the variability among the most representative structures is substantially limited: no major structural variation or twisting in the duplex is observed (Figure 7). Moreover, the effect of simulating ionic strength on the dynamics of the duplex is confirmed by clustering results: with ionic strength conditions, a larger number of different conformations are needed to reach the level of 80% representativeness.

### Validation of the Simulation Conditions by Computed vs Experimental NOE

To gain further insights into the different effects of the four simulation conditions on LTR-III structural properties, we calculated interproton distances from the simulations as ⟨*r*^–6^⟩^–1/6^ averages to compare them to experimentally determined NOE constraints. The averages for each system were calculated on the equilibrated parts of the respective trajectories based on the entire structural RMSD. Experimentally, each distance is characterized by an average value, a lower limit (minimum value), and an upper limit (maximum value). Considering the various uncertainties attached to deriving NOE upper and lower limits from NMR experiments, we consider a violation of an NOE when the limits are exceeded by more than 1 Å.^73,74^

**Figure 8:**
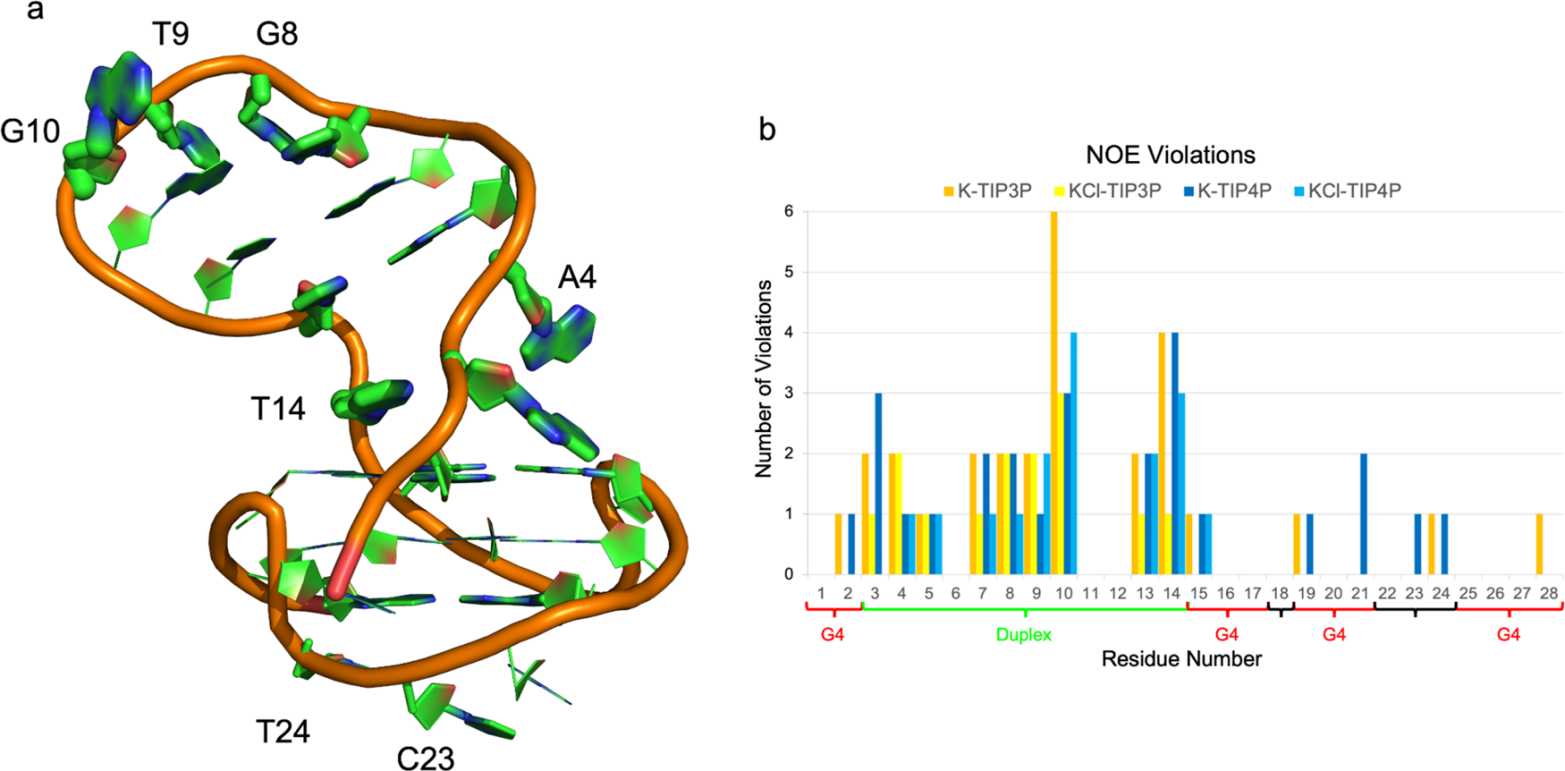
(a) Schematic representation of NOE violations. Residue dimension is directly proportional to the number of violations. (b) Histogram representing the number of violations per residues with different solvent environments.

The NOE distance-bound violations, summarized in Table S1, show that the simulations sample regions of conformational space in which LTR-III fulfills most of the available 433 NMR-derived constraints. A minimal amount of violations were observed in **KCl-TIP3P** and **KCl-TIP4P** with seven violations and eight violations, respectively. In **K-TIP3P** and **K-TIP4P**, the number of violations almost duplicate with 14 and 13 violations, respectively. It is worth noting that the majority of violations derive from the duplex moiety and the unique quadruplex–duplex junction, regardless of the simulation setups (Figure 8). However, sporadically, G4 residues may be involved in NOE violations as well, particularly in **K-TIP3P** and **K-TIP4P**. Here, consistent with the observed low RMSD-G4, the structural deviation that leads to NOE violations does not derive from G4 residues but from residues outside the tetrad motifs. In other words, NOE violations do not appear to stem from G4 structural variations.

To understand the origin of the violations, we focused on the structural variations of the residues involved in their observation. In detail, violations from residues A4 and T14 derive from the closed conformation observed in simulations, while in the NMR ensemble, the junction is mostly open, in spite of the fact that the two bases at the opposite sides of the junction are complementary and thus prone to pair-forming a Watson–Crick pair (see Figure 8; residues undergoing conformational changes linked to NOE violations are shown with larger volumes). Here, NOE violations are due to a preferential conformation adopted in the simulations that differs from what is observed in NMR experiments. Indeed, our simulations start from an open state of the junction, which converges quickly to a closed conformation (with the two hydrogen bonds of the complementary AT base pair formed) and stabilizes until the end of the simulations. This observation is preserved over different solvent environments. We did not observe the transition from the closed conformation to the open one, probably because of the limited time scale of the simulation. Moreover, these data suggest that the closed conformation with double hydrogen bonding is more stable than the open one. On the other hand, it is possible to hypothesize that the force field favors this complementary base pairing preventing junction reopening.

Further structural variations concern solvent-exposed residues: G3, G8, T9, G10, C23, and T24. All of these residues are not interacting with other nucleobases through Hoogsteen or Watson–Crick base pairing. Consequently, these residues are free to float in the solvent leading to NOE violations. In this context, violations are not due to a preferential conformation but rather to a large movement that deviates from the experimentally resolved structures, obtained by imposing structural restraints.

The models appear to be sensitive to different solvent environments. Indeed, violations from both junction and from solvent-exposed residues decrease when ionic strength is considered (KCl-TIP3P and KCl-TIP4P) (see Table S1). Considering that also the NMR experiments were conducted with added ionic strengths, it is possible to say that the force field, under simulation conditions reproducing this situation, works well in generating a conformational ensemble more consistent with the experimental one with respect to K-TIP3P and K-TIP4P.

Taken together, the comparison between the experimental NOEs and the distances averaged from the simulations indicates that overall the simulations satisfy the experimental constraints. It is important to note that different systems appear to satisfy different sets of NOEs. Interestingly, **KCl-TIP4P** and **KCl-TIP3P** show the highest flexibility in the duplex and the minimum number of NOE violations. These data suggest that in these conditions, the system samples more efficiently an ensemble of states characterized by the presence of different structures for the duplex, which together satisfy the NOEs. Indeed, NOE-derived distances represent an upper bound of distances in an ensemble of structures. As a consequence, sets of different structures can concur to match the experimental NOE distances.

We also analyzed the NOE violations from 10 structures from the NMR bundle. Only structures NMR-5 and NMR-9 violate one NOE restraint each (see Table S2, Supporting Information). Interestingly, these violations derive from residues making up the unique quadruplex–duplex junction (residues A4 and T14). Interestingly, violations due to residues 4 and 14 recur in all our simulation setups. The NOE concerning these residues refers to an open state of the junction, which we do not observe in our simulations. As mentioned above, NMR experiments show these residues in both closed and open conformations.^22^

Additionally, we analyzed the distribution of the distances and angles that describe the H-bond formation between bases and play an important role in maintaining the stability of the structure.

We differentiated the analysis between adjacent guanines of the three tetrads in the G4 and three base pairs of guanine–cytosine in the duplex (we considered the H-bonds only for residues involved in base pairing in the experimental structure) and among solvent environments. We then compared the distribution from MD simulations with the data from the NMR bundle.

Concerning G4 residues, guanines adopt a tetrad conformation forming a circular hydrogen-bonding scheme with two types of bonds, named N1–O6 and N2–N7; each tetrad contains four N1–O6 and four N2–N7 hydrogen bonds. We thus inspected the distributions of 24 distances between acceptor and donor atoms and the corresponding angles, which define the H-bond network of the three tetrads. The distributions are presented in Figures S21–S32, where we also reported the corresponding average value (red vertical line) calculated from the NMR structures. In general, no differences were observed across different solvent environments. The distances of N2–N7 in all tetrads agree well with the experimental structure, while the angle distributions show that most of the values are higher than the experimental ones. As for the N1–O6 bond, the distance distributions are peaked around slightly shorter values compared to the experimental ones and the upper tails go to zero for values lower than 3.5 Å, while also, in this case, the peaks of the angle distributions are at higher values than the experimental ones.

For each hydrogen bond, we evaluate the persistence during the MD simulations, using a distance cutoff of 3.0 Å between donor and acceptor and an angle cutoff of 135° to consider when a hydrogen bond is formed (see Table S3, Supporting Information).

The overall performance of the force field supports its use in other studies of these systems, also considering that we did not observe deformations of the tetrads of G4, an indication that they maintain their stability along the simulation time.

In addition, we inspected the existence of the bifurcated N1–N7 hydrogen bonds in the MD simulations, which is considered as an artifact due to the inaccuracy of the force field (see Table S4 and Figures S33–S38, Supporting Information). As can be seen from the distributions and the persistence analysis, N1–N7 hydrogen bonds exist occasionally in all tetrads and simulated systems throughout the MD simulation, and bifurcated hydrogen bonds can form with a very short lifetime giving an overall negligible population.

For the duplex, we monitored nine distances and the angles of the three H-bonds that can be formed between guanine and cytosine, named O6–N4, N1–N3, and N2–O2 (see Figures S39–S44, Supporting Information). Also, in this case, we observed a general agreement with the experimental data in the distance distributions, while the angle distributions show that the most frequent values are higher than the experimental ones. Distributions of the KCl-TIP3P show minor differences from the other solvent environments, with slightly larger deviations from the experimental data.

To further examine the performance of the force field, we computed the time distribution of dihedrals in the 28 deoxyribonucleotides forming the simulated system and compared with experimental values of the 10 NMR structures available in 6H1K. The distributions were obtained using a bin size of 15° and afterward normalized. The results have been reported in a compact matrix representation (see Figures S45–S72, Supporting Information), where each column represents the six monitored dihedrals, while the rows correspond to the clustered torsion angle values. The color intensity reflects the percentage of occupation of each dihedral obtained from MD simulations, while the black dots indicate the corresponding torsion angle value in each of the 10 NMR structures.

Overall, the distributions are similar for each residue in all of the simulated environments; in some cases, slight differences are observed between systems with different water models, while ionic strength does not seem to strongly affect the visited microstates described by these dihedrals. There are no substantial differences between the behavior of torsions in the duplex and the quadruplex.

Experimental values of some dihedrals span a wide range, and the same behavior is observed from the simulation-based distributions. In some cases, the torsion angles cover the entire range of values, while others stabilize different geometries compared to those in the NMR structures. In particular, the force field does not match the experimental values of δ and γ dihedrals; the deviation from the experimental values is more evident for δ, which shows low variability in the NMR structures, differently from γ that spans over a wider number of possible values. However, in many residues, the most frequent experimental values of these two dihedrals are close to the most populated value ensembles observed in MD simulations.

As for the other monitored dihedrals, a combination of values with increased probability similar for all residues is consistent with experimental distributions for α, β, and ζ. In some residues of G4, ε (which notably shows high variability in the NMR structures) deviates from the experimental values.

Overall, the combination of torsion angles favored by the force field results in a general stable geometry of the simulated structure, which substantially agrees with the NMR-derived conformational parameters.

Summarizing, different combinations of water models and salt concentrations used in our simulations impact the stability of the entire structure and the interactions with counterions either with added salts or with no ionic strengths.

Our simulations confirm the importance of the ions in the central anionic channel in the stability of G4 structures, as the lack of ions at the beginning of the simulations induces significant structural modification compared to the experimental ones.

Using the TIP3P or the TIP4P-Ew water models, the quadruplex was capable of attracting the ions in the channel. The mechanism of ion entry is affected by both water models and ionic strengths. In particular, we observed that both TIP4P-Ew and ionic strength (100 mM KCl) speed up this process. No ion exits from the channel were observed in a total of 12 μs of simulation time.

Addition of KCl (to 100 mM) leads to higher structural fluctuations in the duplex moiety for both water models, as shown by the RMSF profiles and clustering analysis. Moreover, from NOE analysis, we observed that ionic strength implementation leads to a lower number of violations compared to K-TIP3P and K-TIP4P. Taken together, these observations define an overall effect of ionic strength that leads to the exploration of a conformational ensemble that better matches the experimental data compared to K-TIP3P and K-TIP4P.

Overall, we observed that differences in simulations arise from different ionic strength conditions. The results indicate that the **KCl-TIP3P** and **KCl-TIP4P** simulations return structural ensembles for the LTR-III structure that are more consistent with NOE-derived data than the structural ensembles determined by **K-TIP3P** and **K-TIP4P**. Subtler differences result from different water models used in simulations. In this context, **KCl-TIP4P** (based on the TIP4P-Ew water model) shows a more stable presence of K^+^-ions in the quadruplex central cavity and a more continuous K^+^-ion distribution around LTR-III grooves compared to the other three simulation setups, in particular, to **K-TIP3P**. Taken together, these results suggest the **KCl-TIP4P** environment setting as the best one for further investigation of the LTR-III system with MD simulations.

## Conclusions

This work represents one of the first steps in the development of a general approach for the characterization of the dynamics of a complex G-quadruplex structure of key pharmacological relevance, namely, the LTR-III region from HIV-1. Interestingly, the system we studied comprises different structural features that make it interesting and challenging: a stable G4 core, flexible junctions, and a solvent-exposed duplex region. Molecular dynamics simulations allowed us to identify the preferential conformational ensembles of LTR-III. By combining and comparing MD simulation results with experimental NMR data, we could shed light on the behavior of LTR-III at atomistic resolution. In general, we find an excellent agreement between simulation-derived data and experimental data. Analysis of structural parameters (H-bond patterns, dihedral angle distributions) shows substantial agreement with the values in the NMR bundle that have been determined experimentally. Unbiased long simulations violate a minimum of 7 to a maximum of 14 NOEs (located in the aforementioned highly flexible regions) on a total of more than 400 experimental NOEs. In this context, the simulations violate a very low number of NOEs: importantly, violations are concentrated on residues and regions that are highly solvent-exposed or belong to very flexible regions. The violations in the junction region are observed after two bases A and T, which point in opposite directions toward the solvent in the experimental bundle, form the complementary H-bonding interactions expected for a Watson–Crick pairing. In this context, it is worth considering that NOE intensities are the measured average values that may not correspond to an energetically accessible conformation of the solute actually existing in solution. Indeed, different DNA conformational families can be present in solution, and a subpopulation of these conformations may be sufficient to satisfy restraints.^75^ Unrestrained MD simulations can contribute to enrich and expand the interpretation of experimental data in terms of conformational distributions. Here, we propose a dynamic model of LTR-III, whereby the G4 motif preferentially populates a single stable conformation, while the duplex moiety is flexible and shows conformational variability, particularly evident in the junction and unpaired loop residues. Keeping such dynamic variability into account coupled with the detailed definition of regions where ions and water stably engage the DNA may be useful in establishing computational protocols for future design applications. In this context, we propose that the structures explored for the double-stranded moiety, where water molecules are not stably engaged in interactions with the nucleic acid and can be easily displaced, can constitute engagement points to diversify/expand G4-targeting derivatives, generating molecules with LTR-III selectivity profiles.

## Materials and Methods

MD simulations were performed using Amber18^76^ pmemd.CUDA with the all-atom BSC1 DNA force field^62^ under periodic boundary conditions.

The starting structure was downloaded from the Protein Data Bank, PDB id 6H1K. The solute was explicitly solvated in a triclinic simulative box and buffered with a 1 nm layer of TIP3P/TIP4P-Ew water molecules^68,69^ and then rendered electroneutral by the addition of potassium counterions. KCl salt was added in two systems to reach a concentration value of 100 mM. A total of four systems were prepared, each of which was simulated in three independent replicates of 1 μs length. The initial structure is common to all systems. The independence of the replicates was ensured by randomizing the initial velocity for each simulation at the beginning of the equilibration stage (vide infra).

To remove any bad contacts between solute and solvent, every system was minimized with position restraints on the solute coordinates, with 500 steps of steepest descent followed by 500 steps of conjugate gradient.

The whole system was then minimized with 2500 conjugate gradient steps without restraints. The temperature of the system was then increased from 0 to 300 K in the NVT ensemble, running 20 ps of MD with weak positional restraints on the DNA with the Langevin thermostat to avoid any large fluctuations.

The systems were then equilibrated at 300 K for 100 ps with a 2 fs time step in periodic boundary conditions in the NPT ensemble, with initial velocities for each replicate obtained from a Maxwellian distribution at the initial temperature of 300 K. The electrostatic interactions were treated using the particle mesh Ewald method^77^ with a cutoff of 10 Å. The same cutoff was used even for short-range Lennard-Jones interactions. Bonds involving hydrogen atoms were constrained with the SHAKE algorithm.^78^ Production runs of each system were extended to 1 μs with an identical setup to the final equilibration conditions.

Trajectories were analyzed using the cpptraj module in the Amber18 package.^79^ To decrease the clutter and to increase the clarity in root-mean-square deviation (RMSD) plots, we calculated the running averages over 1000 neighbor points.

Radial distribution functions (RDFs) were calculated using the “radial” command. Accordingly, RDFs were calculated from the histogram of the number of particles found as a function of distance R (unaltered RDF) and normalized by the expected number of particles at that distance. We calculated all of the RDFs keeping into account the expected density of ions in the bulk. More specifically, the RDF was calculated, counting all particles that are at a distance between *r* and *r +* d*r* away from the particle we were considering, and this number has been normalized by the expected number of particles at that distance ρ*4π*r*^2^ d*r*, where ρ is the reference bulk density. As a reference bulk density, we used the density appearing in a spherical shell at 30 Å from the center of mass of the DNA. RDFs were calculated as the RDF of the K^+^ with respect to the center of mass of the selected solute atoms.

Spatial distribution functions (SDFs) were calculated using the “grid” command in cpptraj. Both analyses were normalized by a particle density value of 0.0033546 Å^–3^, which corresponds to a density of water of approximately 1.0 g/mL. SDFs were calculated on grid volumes of 0.125 Å^3^ and displayed around the average structure computed over all simulation data.

Cluster analyses were carried out using the hierarchical agglomerative algorithm from cluster command in cpptraj. Clustering is carried out in the following way: first, the structures from the respective combined trajectory are aligned on the quadruplex; then, the structures of the duplex are used to define the different conformational families. For each system, six representative conformations were collected.

H-bond analyses were performed, monitoring angles and distance distribution over the entire trajectories for each solvent environment. For each frame, the angle and the distance of each H-bond were calculated using “angle” and “distance” commands in cpptraj, respectively. Thus, for each frame, we obtained 24 values of angle and distance for G4 residues and 9 for duplex residues. From the resulting trajectories, we calculated the histograms with “histogram” command in Xmgrace.

Structural representations were created using PyMol.

## Supporting Information Available

Representative time evolution of distances (Figures S1–S12); RDFs (Figures S13–S16); SDFs of water around the most populated cluster for each simulation environment (Figure S17); time evolution of RMSD (Figures S18–S20); summary of NOE violations from simulations (Table S1); summary of NOE violations from NMR bundle (Table S2); H-bond persistence is reported as a percentage over the entire trajectory (Table S3); bifurcated N1–N7/N2–N7 H-bond persistence is represented as a percentage over the entire trajectory (Table S4); and free-energy barrier for ion binding process is roughly estimated from the distribution of the bound and unbound states along the trajectories (Table S5) (PDF)
Time distributions of the parameters defining H-bonds and the distributions of dihedral angles, compared to the experimental values; each figure is identified by the title, reporting on the simulation conditions and the specific quantity being reported (Figures S21–S72) (ZIP )

## References

[ref1] Burge S.; Parkinson G. N.; Hazel P.; Todd A. K.; Neidle S. Quadruplex DNA: sequence, topology and structure. Nucleic Acids Res. 2006, 34, 5402–5415. 10.1093/nar/gkl655. 17012276 PMC1636468

[ref2] Bhattacharyya D.; Arachchilage G. M.; Basu S. Metal Cations in G-Quadruplex Folding and Stability. Front. Chem. 2016, 4, 38 10.3389/fchem.2016.00038. 27668212 PMC5016522

[ref3] Dvorkin S. A.; Karsisiotis A. I.; da Silva M. W. Encoding canonical DNA quadruplex structure. Sci. Adv. 2018, 4, eaat3007 10.1126/sciadv.aat3007. 30182059 PMC6118410

[ref4] Lightfoot H. L.; Hagen T.; Tatum N. J.; Hall J. The diverse structural landscape of quadruplexes. FEBS Lett. 2019, 593, 2083–2102. 10.1002/1873-3468.13547. 31325371

[ref5] Huppert J. H.; Balasubramanian S. Prevalence of quadruplexes in human genome. Nucleic Acids Res. 2005, 33, 2908–2916. 10.1093/nar/gki609. 15914667 PMC1140081

[ref6] Hänsel-Hertsch R.; Di Antonio M.; Balasubramanian S. DNA G-quadruplexes in the human genome: detection, functions and therapeutic potential. Nat. Rev. Mol. Cell. Biol. 2017, 18, 279 10.1038/nrm.2017.3. 28225080

[ref7] Neidle S. Human telomeric G-quadruplex: the current status of telomeric G-quadruplexes as therapeutic targets in human cancer. FEBS J. 2010, 277, 1118–1125. 10.1111/j.1742-4658.2009.07463.x. 19951354

[ref8] Huppert J. L.; Balasubramanian S. G-quadruplexes in promoters throughout the human genome. Nucleic Acid Res. 2007, 35, 406–413. 10.1093/nar/gkl1057. 17169996 PMC1802602

[ref9] Prioleau M. N. G-Quadruplexes and DNA Replication Origins. DNA Replication, Advances in Experimental Medicine and Biology; Springer, 2017; Vol. 1042, pp 273–286. 10.1007/978-981-10-6955-0_13 29357063

[ref10] Kwok C. K.; Marsico G.; Balasubramanian S. Detecting RNA G-Quadruplexes (rG4s) in the Transcriptome. Cold Spring Harbor Perspect. Biol. 2018, 10 10.1101/cshperspect.a032284. PMC6028067 29967010

[ref11] Rhodes D.; Lipps H. J. G-quadruplexes and their regulatory roles in biology. Nucleic Acid Res. 2015, 43, 8627–8637. 10.1093/nar/gkv862. 26350216 PMC4605312

[ref12] Banerjee N.; Panda S.; Chatterjee S. G-Quadruplex targeting ligands: A hope and a new horizon in Cancer Therapeutics. Preprints 2018, 2018110409 10.20944/preprints201811.0409.v1.

[ref13] Taylor J. P. Neurodegenerative diseases: G-quadruplex poses quadruple threat. Nature 2014, 507, 175–177. 10.1038/nature13067. 24598546

[ref14] Verma A.; Halder K.; Halder R.; Yadav V. K.; Rawal P.; Thakur R. K.; Mohd F.; Sharma A.; Chowdhury S. Genome-wide computational and expression analyses reveal G-quadruplex DNA motifs as conserved cis-regulatory elements in human and related species. J. Med. Chem. 2008, 51, 5641–5649. 10.1021/jm800448a. 18767830

[ref15] Beaume N.; Pathak R.; Yadav V. K.; Kota S.; Misra H. S.; Gautam H. K.; Chowdhury S. Genome-wide study predicts promoter-G4 DNA motifs regulate selective functions in bacteria: radioresistance of D. radiodurans involves G4 DNA-mediated regulation. Nucleic Acid Res. 2013, 41, 76–89. 10.1093/nar/gks1071. 23161683 PMC3592403

[ref16] Bartas M.; Čutová M.; Brázda V.; Kaura P.; Št’astný J.; Kolomazník J.; Coufal J.; Goswami P.; Červeň J.; Pečinka P. The Presence and Localization of G-Quadruplex Forming Sequences in the Domain of Bacteria. Molecules 2019, 24, 1711 10.3390/molecules24091711. PMC6539912 31052562

[ref17] Belmonte-Reche E.; Martínez-García M.; Guédin A.; Zuffo M.; Arévalo-Ruiz M.; Doria F.; Campos-Salinas J.; Maynadier M.; López-Rubio J. J.; Freccero M.; Mergny J.; Pérez-Victoria J. M.; Morales J. C. G-Quadruplex Identification in the Genome of Protozoan Parasites Points to Naphthalene Diimide Ligands as New Antiparasitic Agents. J. Med. Chem. 2018, 61, 1231–1240. 10.1021/acs.jmedchem.7b01672. 29323491 PMC6148440

[ref18] Singh S.; Berroyer A.; Kim M.; Kim N. Yeast Nucleolin Nsr1 Impedes Replication and Elevates Genome Instability at an Actively Transcribed Guanine-Rich G4-DNA Forming Sequence. Genetics 2020, 216, 1023–1037. 10.1534/genetics.120.303736. 33106247 PMC7768239

[ref19] Lavezzo E.; Berselli M.; Frasson I.; Perrone R.; Palù G.; Brazzale A. R.; Richter S. N.; Toppo S. G-quadruplex forming sequences in the genome of all known human viruses: A comprehensive guide. PLoS Comput. Biol. 2018, 14, e1006675 10.1371/journal.pcbi.1006675. 30543627 PMC6307822

[ref20] Ruggiero E.; Richter S. N. G-quadruplexes and G-quadruplex ligands: targets and tools in antiviral therapy. Nucleic Acids Res. 2018, 46, 3270–3283. 10.1093/nar/gky187. 29554280 PMC5909458

[ref21] Perrone R.; Nadai M.; Frasson I.; Poe J. A.; Butovskaya E.; Smithgall T. E.; Palumbo M.; Palù G.; Richter S. N. A dynamic G-quadruplex region regulates the HIV-1 long terminal repeat promoter. J. Med. Chem. 2013, 56, 6521–6530. 10.1021/jm400914r. 23865750 PMC3791109

[ref22] Butovskaya E.; Heddi B.; Bakalar B.; Richter S. N.; Phan A. T. Major G-Quadruplex Form of HIV-1 LTR Reveals a (3+1) Folding Topology Containing a Stem-Loop. J. Am. Chem. Soc. 2018, 140, 13654–13662. 10.1021/jacs.8b05332. 30299955 PMC6202629

[ref23] De Nicola B.; Lech C. J.; Heddi B.; Regmi S.; Frasson I.; Perrone R.; Richter S. N.; Phan A. T. Structure and possible function of a G-quadruplex in the long terminal repeat of the proviral HIV-1 genome. Nucleic Acids Res. 2016, 44, 6442–6451. 10.1093/nar/gkw432. 27298260 PMC5291261

[ref24] Perrone R.; Butovskaya E.; Daelemans D.; Palù G.; Pannecouque C.; Richter S. N. Anti-HIV-1 activity of the G-quadruplex ligand BRACO-19. J. Antimicrob. Chemother. 2014, 69, 3248–3258. 10.1093/jac/dku280. 25103489

[ref25] Tosoni E.; Frasson I.; Scalabrin M.; Perrone R.; Butovskaya E.; Nadai M.; Palù G.; Fabris D.; Richter S. N. Nucleolin stabilizes G-quadruplex structures folded by the LTR promoter and silences HIV-1 viral transcription. Nucleic Acids Res. 2015, 43, 8884–8897. 10.1093/nar/gkv897. 26354862 PMC4605322

[ref26] Scalabrin M.; Frasson I.; Ruggiero E.; Perrone R.; Tosoni E.; Lago S.; Tassinari M.; Palù G.; Richter S. N. The cellular protein hnRNP A2/B1 enhances HIV-1 transcription by unfolding LTR promoter G-quadruplexes. Sci. Rep. 2017, 7, 45244 10.1038/srep45244. 28338097 PMC5364415

[ref27] Tassinari M.; Zuffo M.; Nadai M.; Pirota V.; Sevilla Montalvo A. C.; Doria F.; Freccero M.; Richter S. N. Selective targeting of mutually exclusive DNA G-quadruplexes: HIV-1 LTR as paradigmatic model. Nucleic Acids Res. 2020, 48, 4627–4642. 10.1093/nar/gkaa186. 32282912 PMC7229848

[ref28] Nadai M.; Doria F.; Scalabrin M.; Pirota V.; Grande V.; Bergamaschi G.; Amendola V.; Winnerdy F. R.; Phan A. T.; Richter S. N.; Freccero M. A Catalytic and Selective Scissoring Molecular Tool for Quadruplex Nucleic Acids. J. Am. Chem. Soc. 2018, 140, 14528–14532. 10.1021/jacs.8b05337. 30351011 PMC6242190

[ref29] Liu H. Y.; Zhao Q.; Zhang T. P.; Wu Y.; Xiong Y. X.; Wang S. K.; Ge Y. L.; He J. H.; Lv P.; Ou T. M.; Tan J. H.; Li D.; Gu L. Q.; Ren J.; Zhao Y.; Huang Z. S. Conformation Selective Antibody Enables Genome Profiling and Leads to Discovery of Parallel G-Quadruplex in Human Telomeres. Cell Chem. Biol. 2016, 23, 1261–1270. 10.1016/j.chembiol.2016.08.013. 27693060

[ref30] Jana J.; Mondal S.; Bhattacharjee P.; Sengupta P.; Roychowdhury T.; Saha P.; Kundu P.; Chatterjee S. Chelerythrine down regulates expression of VEGFA, BCL2 and KRAS by arresting G-Quadruplex structures at their promoter regions. Sci. Rep. 2017, 7, 40706 10.1038/srep40706. 28102286 PMC5244364

[ref31] Benz A.; Singh V.; Mayer T. U.; Hartig J. S. Identification of novel quadruplex ligands from small molecule libraries by FRET-based high-throughput screening. ChemBioChem 2011, 12, 1422–1426. 10.1002/cbic.201100094. 21618675

[ref32] Zuffo M.; Guédin A.; Leriche E. D.; Doria F.; Pirota V.; Gabelica V.; Mergny J. L.; Freccero M. More is not always better: finding the right trade-off between affinity and selectivity of a G-quadruplex ligand. Nucleic Acids Res. 2018, 46, e115 10.1093/nar/gky607. 29986058 PMC6212845

[ref33] Neidle S. Quadruplex nucleic acids as targets for anticancer therapeutics. Nat. Rev. Chem. 2017, 1, 0041 10.1038/s41570-017-0041.

[ref34] Rodriguez R.; Müller S.; Yeoman J. A.; Trentesaux C.; Riou J. F.; Balasubramanian S. A novel small molecule that alters shelterin integrity and triggers a DNA-damage response at telomeres. J. Am. Chem. Soc. 2008, 130, 15758–15759. 10.1021/ja805615w. 18975896 PMC2746963

[ref35] Ying L.; Green J. J.; Li H.; Klenerman D.; Balasubramanian S. Studies on the structure and dynamics of the human telomeric G quadruplex by single-molecule fluorescence resonance energy transfer. Proc. Natl. Acad. Sci. U.S.A. 2003, 100, 14629–14634. 10.1073/pnas.2433350100. 14645716 PMC299749

[ref36] Mitra J.; Makurath M. A.; Ngo T. T. M.; Troitskaia A.; Chemla Y. R.; Ha T. Extreme mechanical diversity of human telomeric DNA revealed by fluorescence-force spectroscopy. Proc. Natl. Acad. Sci. U.S.A. 2019, 116, 8350–8359. 10.1073/pnas.1815162116. 30944218 PMC6486787

[ref37] Stadlbauer P.; Krepl M.; Cheatham T. E.; Koca J.; Sponer J. Structural dynamics of possible late-stage intermediates in folding of quadruplex DNA studied by molecular simulations. Nucleic Acids Res. 2013, 41, 7128–7143. 10.1093/nar/gkt412. 23700306 PMC3737530

[ref38] Islam B.; Stadlbauer P.; Krepl M.; Koca J.; Neidle S.; Haider S.; Sponer J. Extended molecular dynamics of a c-kit promoter quadruplex. Nucleic Acids Res. 2015, 43, 8673–8693. 10.1093/nar/gkv785. 26245347 PMC4605300

[ref39] Stadlbauer P.; Trantírek L.; Cheatham T. E.; Koča J.; Sponer J. Triplex intermediates in folding of human telomeric quadruplexes probed by microsecond-scale molecular dynamics simulations. Biochimie 2014, 105, 22–35. 10.1016/j.biochi.2014.07.009. 25038568

[ref40] Stadlbauer P.; Mazzanti L.; Cragnolini T.; Wales D. J.; Derreumaux P.; Pasquali S.; Šponer J. Coarse-Grained Simulations Complemented by Atomistic Molecular Dynamics Provide New Insights into Folding and Unfolding of Human Telomeric G-Quadruplexes. J. Chem. Theory Comput. 2016, 12, 6077–6097. 10.1021/acs.jctc.6b00667. 27767303

[ref41] Stadlbauer P.; Kührová P.; Vicherek L.; Banáš P.; Otyepka M.; Trantírek L.; Šponer J. Parallel G-triplexes and G-hairpins as potential transitory ensembles in the folding of parallel-stranded DNA G-Quadruplexes. Nucleic Acids Res. 2019, 47, 7276–7293. 10.1093/nar/gkz610. 31318975 PMC6698752

[ref42] Stadlbauer P.; Kührová P.; Banáš P.; Koča J.; Bussi G.; Trantírek L.; Otyepka M.; Šponer J. Hairpins participating in folding of human telomeric sequence quadruplexes studied by standard and T-REMD simulations. Nucleic Acids Res. 2015, 43, gkv994 10.1093/nar/gkv994. PMC4787745 26433223

[ref43] Havrila M.; Stadlbauer P.; Kührová P.; Banáš P.; Mergny J. L.; Otyepka M.; Šponer J. Structural dynamics of propeller loop: towards folding of RNA G-quadruplex. Nucleic Acids Res. 2018, 46, 8754–8771. 10.1093/nar/gky712. 30165550 PMC6158699

[ref44] Galindo-Murillo R.; Robertson J. C.; Zgarbová M.; Šponer J.; Otyepka M.; Jurečka P.; Cheatham T. E. Assessing the Current State of Amber Force Field Modifications for DNA. J. Chem. Theory Comput. 2016, 12, 4114–4127. 10.1021/acs.jctc.6b00186. 27300587 PMC4980684

[ref45] Šponer J.; Cang X.; Cheatham T. E. Molecular dynamics simulations of G-DNA and perspectives on the simulation of nucleic acid structures. Methods 2012, 57, 25–39. 10.1016/j.ymeth.2012.04.005. 22525788 PMC3775459

[ref46] Gkionis K.; Kruse H.; Platts J. A.; Mládek A.; Koča J.; Šponer J. Ion Binding to Quadruplex DNA Stems. Comparison of MM and QM Descriptions Reveals Sizable Polarization Effects Not Included in Contemporary Simulations. J. Chem. Theory Comput. 2014, 10, 1326–1340. 10.1021/ct4009969. 26580197

[ref47] Song J.; Ji C.; Zhang J. Z. The critical effect of polarization on the dynamical structure of guanine quadruplex DNA. Phys. Chem. Chem. Phys. 2013, 15, 3846–3854. 10.1039/c2cp44100d. 23399949

[ref48] Šponer J.; Bussi G.; Stadlbauer P.; Kührová P.; Banáš P.; Islam B.; Haider S.; Neidle S.; Otyepka M. Folding of guanine quadruplex molecules-funnel-like mechanism or kinetic partitioning? An overview from MD simulation studies. Biochim. Biophys. Acta, Gen. Subj. 2017, 1861, 1246–1263. 10.1016/j.bbagen.2016.12.008. 27979677

[ref49] Havrila M.; Stadlbauer P.; Islam B.; Otyepka M.; Šponer J. Effect of Monovalent Ion Parameters on Molecular Dynamics Simulations of G-Quadruplexes. J. Chem. Theory Comput. 2017, 13, 3911–3926. 10.1021/acs.jctc.7b00257. 28657760

[ref50] Bessi I.; Jonker H. R.; Richter C.; Schwalbe H. Involvement of Long-Lived Intermediate States in the Complex Folding Pathway of the Human Telomeric G-Quadruplex. Angew. Chem., Int. Ed. 2015, 54, 8444–8448. 10.1002/anie.201502286. 26036989

[ref51] Long X.; Stone M. D. Kinetic partitioning modulates human telomere DNA G-quadruplex structural polymorphism. PLoS One 2013, 8, e83420 10.1371/journal.pone.0083420. 24367594 PMC3867459

[ref52] Gray R. D.; Trent J. O.; Chaires J. B. Folding and unfolding pathways of the human telomeric G-quadruplex. J. Mol. Biol. 2014, 426, 1629–1650. 10.1016/j.jmb.2014.01.009. 24487181 PMC3969789

[ref53] Aznauryan M.; Søndergaard S.; Noer S. L.; Schiøtt B.; Birkedal V. A direct view of the complex multi-pathway folding of telomeric G-quadruplexes. Nucleic Acids Res. 2016, 44, 11024–11032. 10.1093/nar/gkw1010. 27799468 PMC5159523

[ref54] Marchand A.; Gabelica V. Folding and misfolding pathways of G-quadruplex DNA. Nucleic Acids Res. 2016, 44, 10999–11012. 10.1093/nar/gkw970. 27924036 PMC5159560

[ref55] Fadrná E.; Špačková N.; Sarzyñska J.; Koča J.; Orozco M.; Cheatham T. E.; Kulinski T.; Šponer J. Single Stranded Loops of Quadruplex DNA As Key Benchmark for Testing Nucleic Acids Force Fields. J. Chem. Theory Comput. 2009, 5, 2514–2530. 10.1021/ct900200k. 26616629

[ref56] Islam B.; Stadlbauer P.; Gil-Ley A.; Pérez-Hernández G.; Haider S.; Neidle S.; Bussi G.; Banas P.; Otyepka M.; Sponer J. Exploring the Dynamics of Propeller Loops in Human Telomeric DNA Quadruplexes Using Atomistic Simulations. J. Chem. Theory Comput. 2017, 13, 2458–2480. 10.1021/acs.jctc.7b00226. 28475322 PMC5514396

[ref57] Islam B.; Stadlbauer P.; Krepl M.; Havrila M.; Haider S.; Sponer J. Structural Dynamics of Lateral and Diagonal Loops of Human Telomeric G-Quadruplexes in Extended MD Simulations. J. Chem. Theory Comput. 2018, 14, 5011–5026. 10.1021/acs.jctc.8b00543. 30183284

[ref58] Rebič M.; Laaksonen A.; Šponer J.; Uličný J.; Mocci F. Molecular Dynamics Simulation Study of Parallel Telomeric DNA Quadruplexes at Different Ionic Strengths: Evaluation of Water and Ion Models. J. Phys. Chem. B 2016, 120, 7380–7391. 10.1021/acs.jpcb.6b06485. 27379924

[ref59] Lemkul J. A.; MacKerell A. D. Jr. Polarizable Force Field for DNA Based on the Classical Drude Oscillator: I. Refinement Using Quantum Mechanical Base Stacking and Conformational Energetics. J. Chem. Theory Comput. 2017, 13, 2053–2071. 10.1021/acs.jctc.7b00067. 28399366 PMC5484419

[ref60] Lemkul J. A.; MacKerell A. D. Jr. Polarizable Force Field for DNA Based on the Classical Drude Oscillator: II. Microsecond Molecular Dynamics Simulations of Duplex DNA. J. Chem. Theory Comput. 2017, 13, 2072–2085. 10.1021/acs.jctc.7b00068. 28398748 PMC5485260

[ref61] Zgarbová M.; Sponer J.; Otyepka M.; Cheatham T. E. III; Galindo-Murillo R.; Jurecka P. Refinement of the Sugar-Phosphate Backbone Torsion Beta for AMBER Force Fields Improves the Description of Z- and B-DNA. J. Chem. Theory Comput. 2015, 11, 5723–5736. 10.1021/acs.jctc.5b00716. 26588601

[ref62] Ivani I.; Dans P. D.; Noy A.; Pérez A.; Faustino I.; Hospital A.; Walther J.; Andrio P.; Goñi R.; Balaceanu A.; Portella G.; Battistini F.; Gelpì J. L.; Gonzàlez C.; Vendruscolo M.; Laughton C. A.; Harris S. A.; Case D. A.; Orozco M. Parmbsc1: a refined force field for DNA simulations. Nat. Methods 2016, 13, 55–58. 10.1038/nmeth.3658. 26569599 PMC4700514

[ref63] Lemkul J. A. Same fold, different properties: polarizable molecular dynamics simulations of telomeric and TERRA G-quadruplexes. Nucleic Acids Res. 2020, 48, 561–575. 10.1093/nar/gkz1154. 31807754 PMC6954416

[ref64] Salsbury A. M.; Lemkul J. A. Molecular Dynamics Simulations of the c-kit1 Promoter G-Quadruplex: Importance of Electronic Polarization on Stability and Cooperative Ion Binding. J. Phys. Chem. B 2019, 123, 148–159. 10.1021/acs.jpcb.8b11026. 30525627

[ref65] Li N.; Gao Y.; Qiu F.; Zhu T. Benchmark Force Fields for the Molecular Dynamic Simulation of G-Quadruplexes. Molecules 2021, 26, 5379 10.3390/molecules26175379. 34500812 PMC8434458

[ref66] Ȧqvist J. Ion-water interaction potentials derived from free energy perturbation simulations. J. Phys. Chem. A 1990, 94, 8021–8024. 10.1021/j100384a009.

[ref67] Joung I. S.; Cheatham T. E. Determination of alkali and halide monovalent ion parameters for use in explicitly solvated biomolecular simulations. J. Phys. Chem. B 2008, 112, 9020–9041. 10.1021/jp8001614. 18593145 PMC2652252

[ref68] Jorgensen W. L.; Chandrasekhar J.; Madura J. D.; Impey R. W.; Klein M. L. Comparison of simple potential functions for simulating liquid water. J. Chem. Phys. 1983, 79, 926 10.1063/1.445869.

[ref69] Horn H. W.; Swope W. C.; Pitera J. W.; Madura J. D.; Dick T. J.; Hura G. L.; Head-Gordon T. Development of an improved four-site water model for biomolecular simulations: TIP4P-Ew. J. Chem. Phys. 2004, 120, 9665–9678. 10.1063/1.1683075. 15267980

[ref70] Jorgensen W. L.; Madura J. D. Temperature and size dependence for Monte Carlo simulations of TIP4P water. Mol. Phys. 1985, 56, 1381 10.1080/00268978500103111.

[ref71] Vchirawongkwin S.; Vchirawongkwin V. Evaluation of molecular radial distribution function and solvent-excluded volume with the numerical integration of the union of spheres. Comput. Theor. Chem. 2011, 974, 26–30. 10.1016/j.comptc.2011.07.007.

[ref72] Rani P.; Biswas P. Shape dependence of the radial distribution function of hydration water around proteins. J. Phys.: Condens. Matter 2014, 26, 335102 10.1088/0953-8984/26/33/335102. 25053697

[ref73] Soares T. A.; Daura X.; Oostenbrink C.; Smith L. J.; van Gunsteren W. F. Validation of the GROMOS force-field parameter set 45Alpha3 against nuclear magnetic resonance data of hen egg lysozyme. J. Biomol. NMR 2004, 30, 407–422. 10.1007/s10858-004-5430-1. 15630561

[ref74] Gattin Z.; Schwartz J.; Mathad R. I.; Jaun B.; van Gunsteren W. F. Interpreting experimental data by using molecular simulation instead of model building. Chem.—Eur. J. 2009, 15, 6389–6398. 10.1002/chem.200802523. 19462385

[ref75] van Gunsteren W. F.; Bakowies D.; Baron R.; Chandrasekhar I.; Christen M.; Daura X.; Gee P.; Geerke D. P.; Glättli A.; Hünenberger P. H.; Kastenholz M. A.; Oostenbrink C.; Schenk M.; Trzesniak D.; van der Vegt N. F. A.; Yu H. B. Biomolecular Modeling: Goals, Problems, Perspectives. Angew. Chem., Int. Ed. 2006, 45, 4064–4092. 10.1002/anie.200502655. 16761306

[ref76] AMBER 2018; University of California: San Francisco, 2018.

[ref77] Darden T.; York D.; Pedersen L. Particle mesh Ewald: An Nlog(N) method for Ewald sums in large systems. J. Chem. Phys. 1993, 98, 10089–10092. 10.1063/1.464397.

[ref78] Miyamoto S.; Kollman P. A. Settle: An analytical version of the SHAKE and RATTLE algorithm for rigid water models. J. Comput. Chem. 1992, 13, 952 10.1002/jcc.540130805.

[ref79] Roe D. R.; Cheatham T. E. PTRAJ and CPPTRAJ: Software for Processing and Analysis of Molecular Dynamics Trajectory Data. J. Chem. Theory Comput. 2013, 9, 3084–3095. 10.1021/ct400341p. 26583988

