# Supplementary material for: Studying the Dynamics of a Complex G-Quadruplex System: Insights into the Comparison of MD and NMR Data
Source: J Chem Theory Comput. 2022 Jun 6;18(7):4515–28. doi: 10.1021/acs.jctc.2c00291 (PMC9281369; doi:10.1021/acs.jctc.2c00291)
Supplement: Supplementary file 1 — ct2c00291_si_001.pdf [file ct2c00291_si_001.pdf]

Supporting Information

Studying the dynamics of a complex G-quadruplex system: insights from the comparison of MD and NMR data

Matteo Castelli[1], Filippo Doria[1], Mauro Freccero[1], Giorgio Colombo[1,2] and Elisabetta Moroni*[2]*

[1] Department of Chemistry, University of Pavia, V.le Taramelli 12, 27100 Pavia, Italy
[2] Institute of Chemical Sciences and Technologies SCITEC-CNR

- Modelling K^+ diffusion

Here, we monitored all the distances between the O6 atoms of the G4 guanine bases and the potassium ions. Once the ion(s) in the channel has been identified, we focused on the eight K^+-O6 distances that characterize the positioning between two G-tetrads. We observed that these distances and their time evolution are almost identical considering one ion at a time. Therefore, as example, we report one K^+-O6 distance for each ion entered in the G4 cavity. This representation allows us to know at what point of the simulation the ion(s) enter the cavity. In most simulations we observed two ions in the cavity, in some cases only one ion enters the cavity (where only one distance is reported).

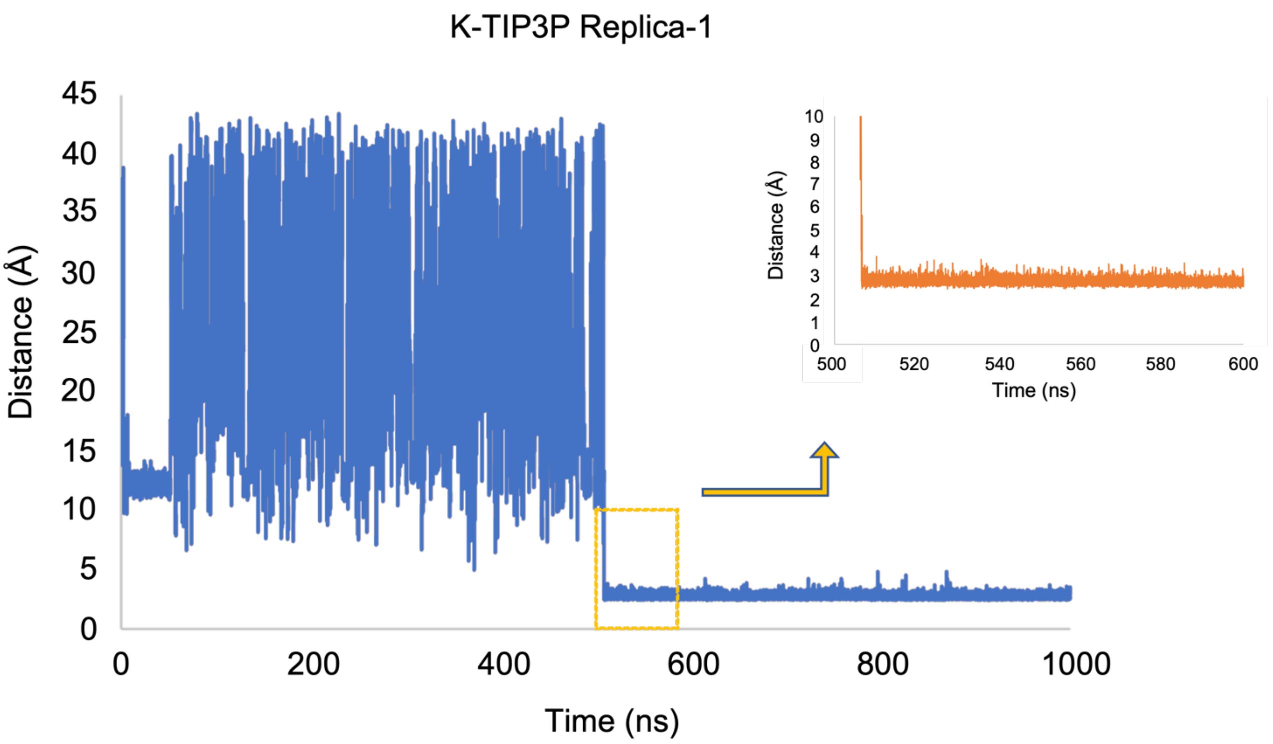

Figure S1: Representative time evolution of distances between G25-O^6 atom and K^+ entering the cavity during K-TIP3P replica-1.

a

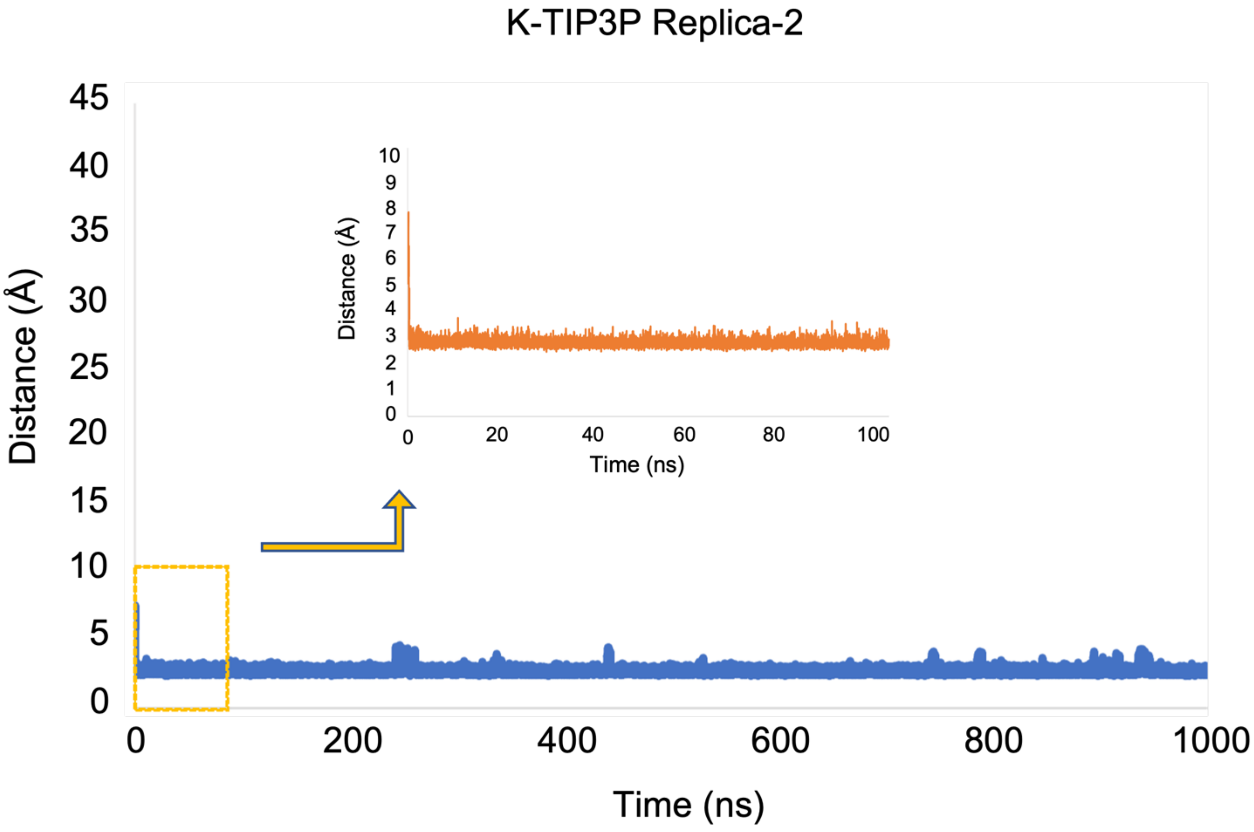

b

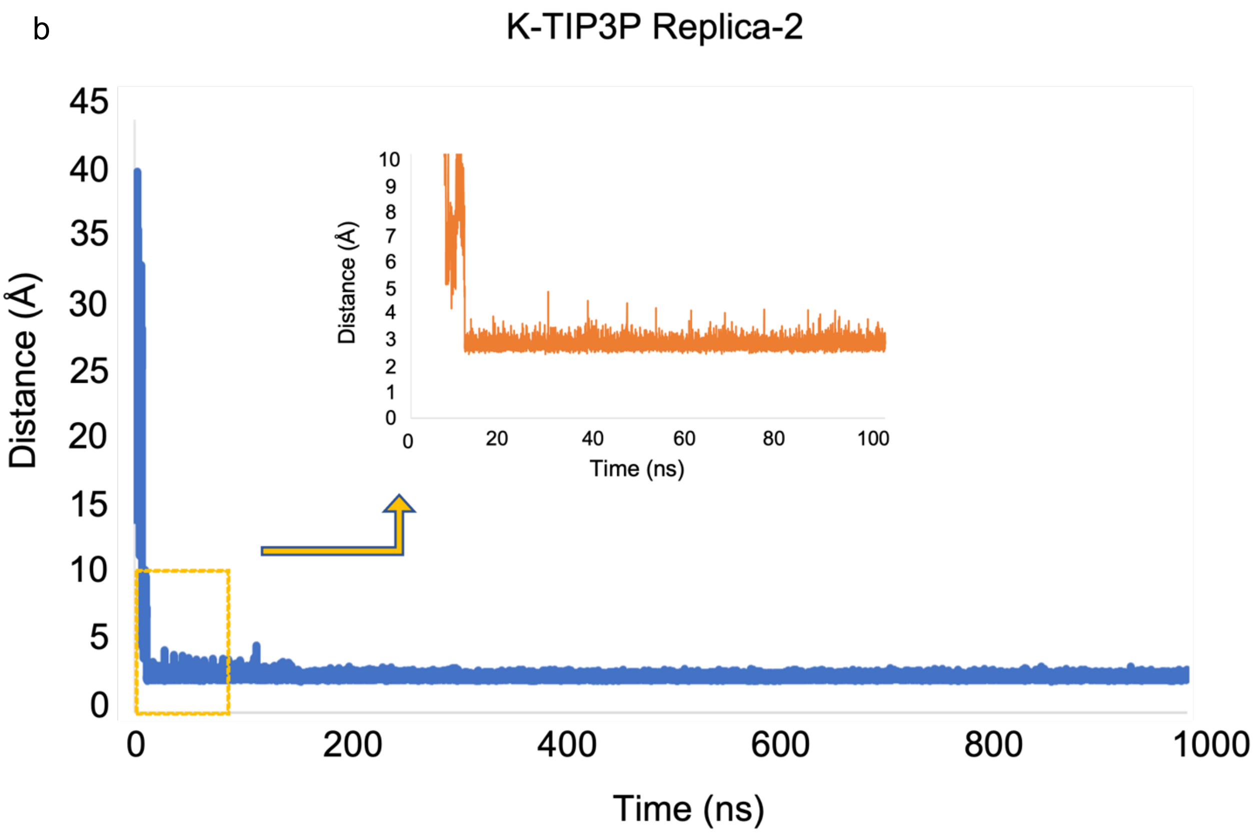

Figure S2: Representative time evolution of distances between G4-O^6 atom and K^+ entering the cavity during K-TIP3P replica-2. (a) K^+/G19-O^6 distance (b) K^+/G21-O^6

a

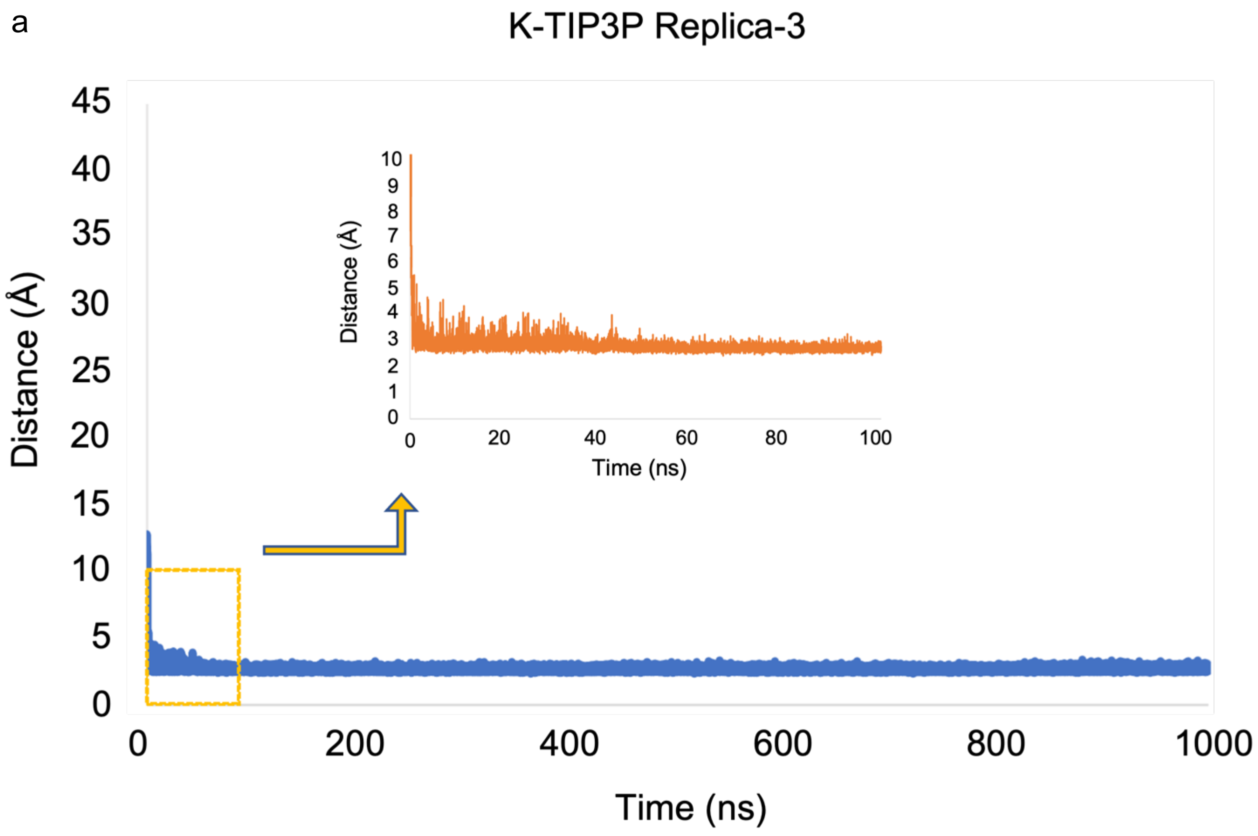

b

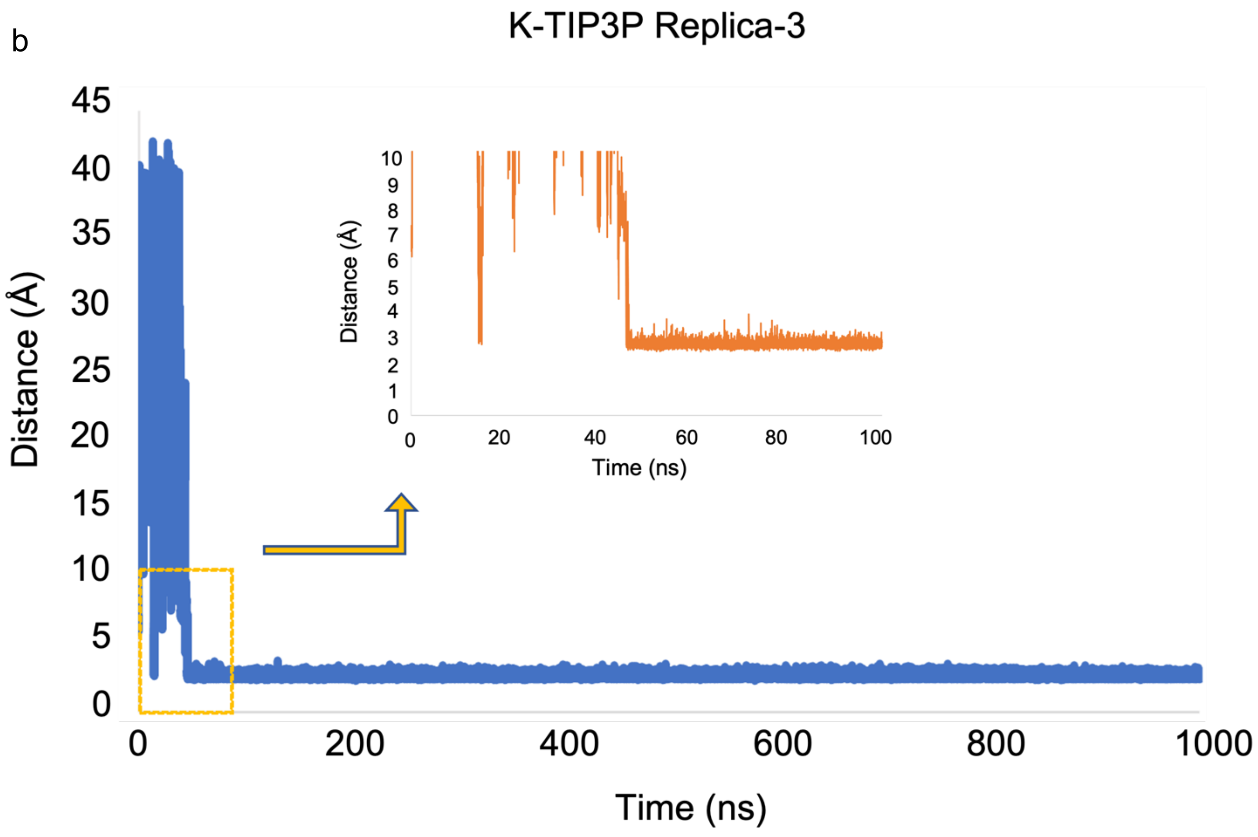

Figure S3: Representative time evolution of distances between G4-O^6 atom and K^+ entering the cavity during K-TIP3P replica-3. (a) K^+/G26-O^6 distance (b) K^+/G17-O^6

a

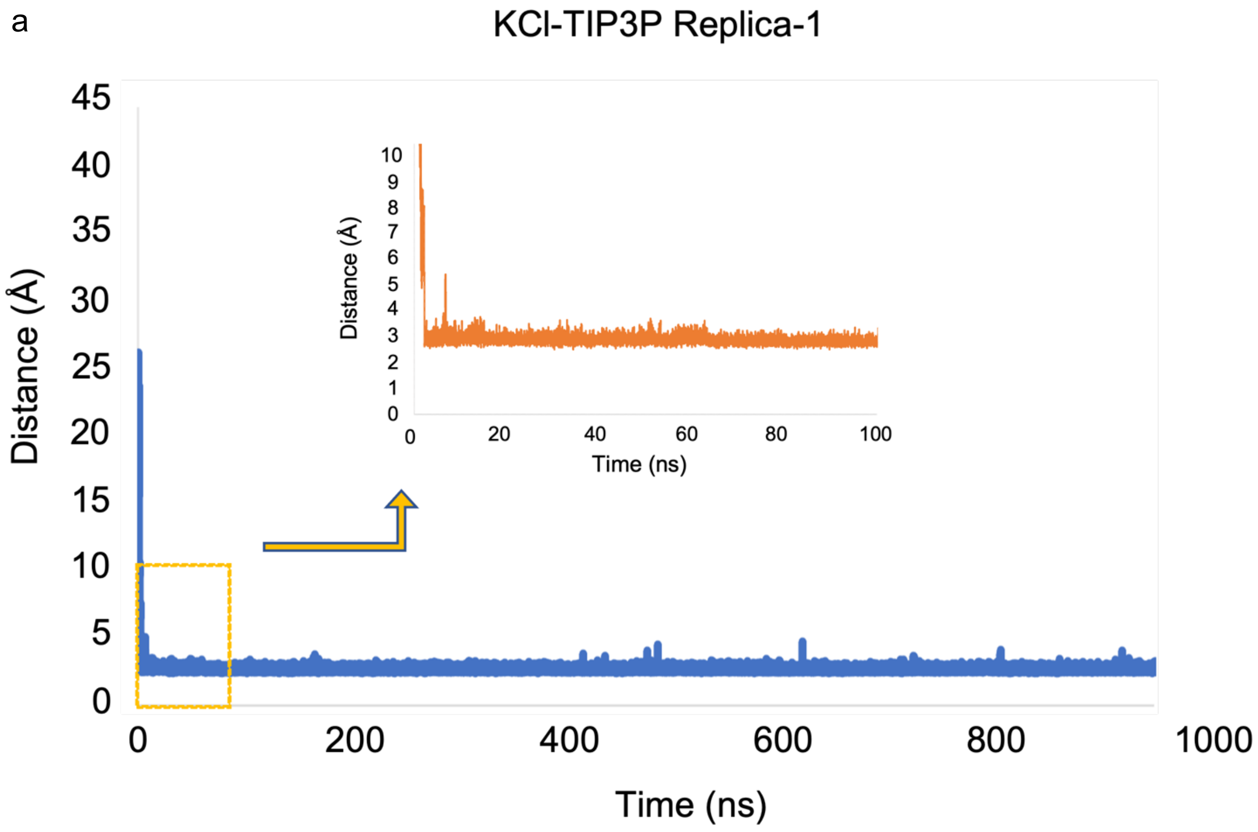

b

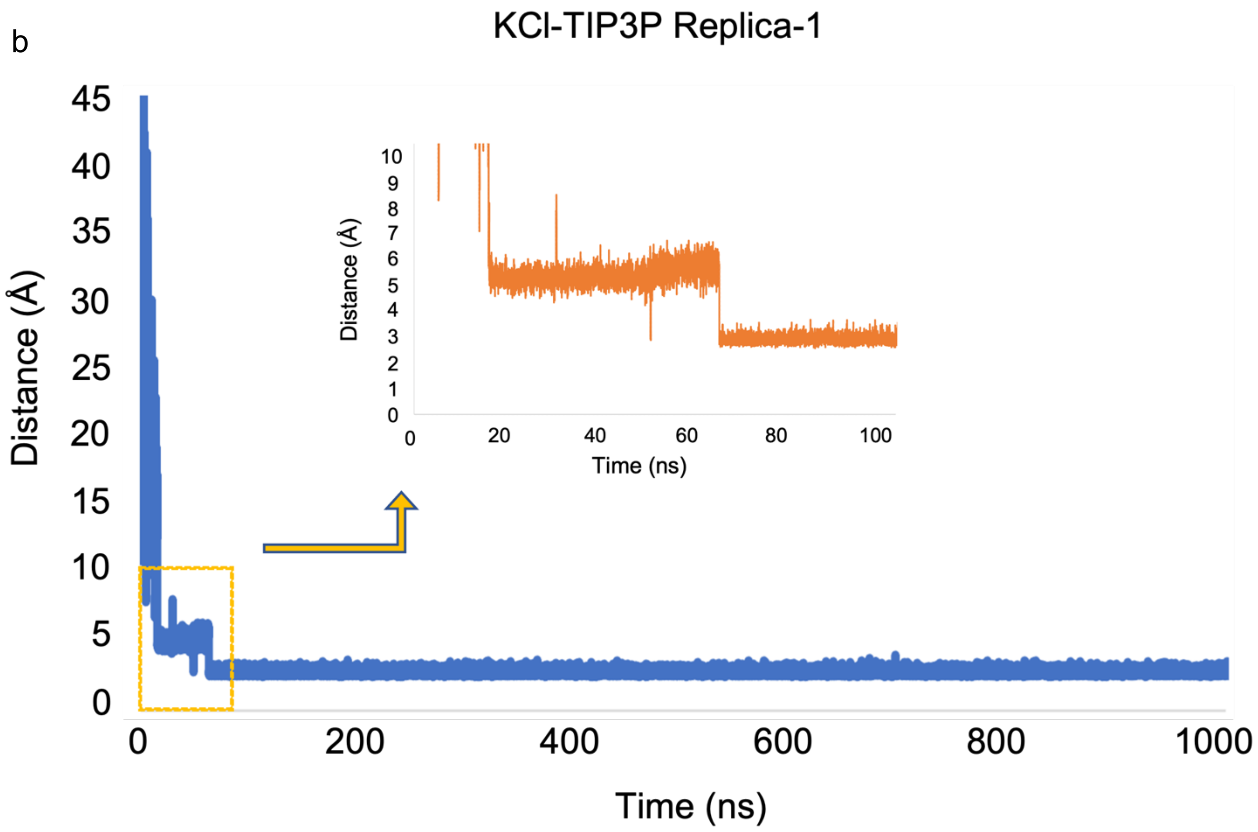

Figure S4: Representative time evolution of distances between G4-O^6 atom and K^+ entering the cavity during KCl-TIP3P replica-1. (a) K^+/G19-O^6 distance (b) K^+/G16-O^6

a

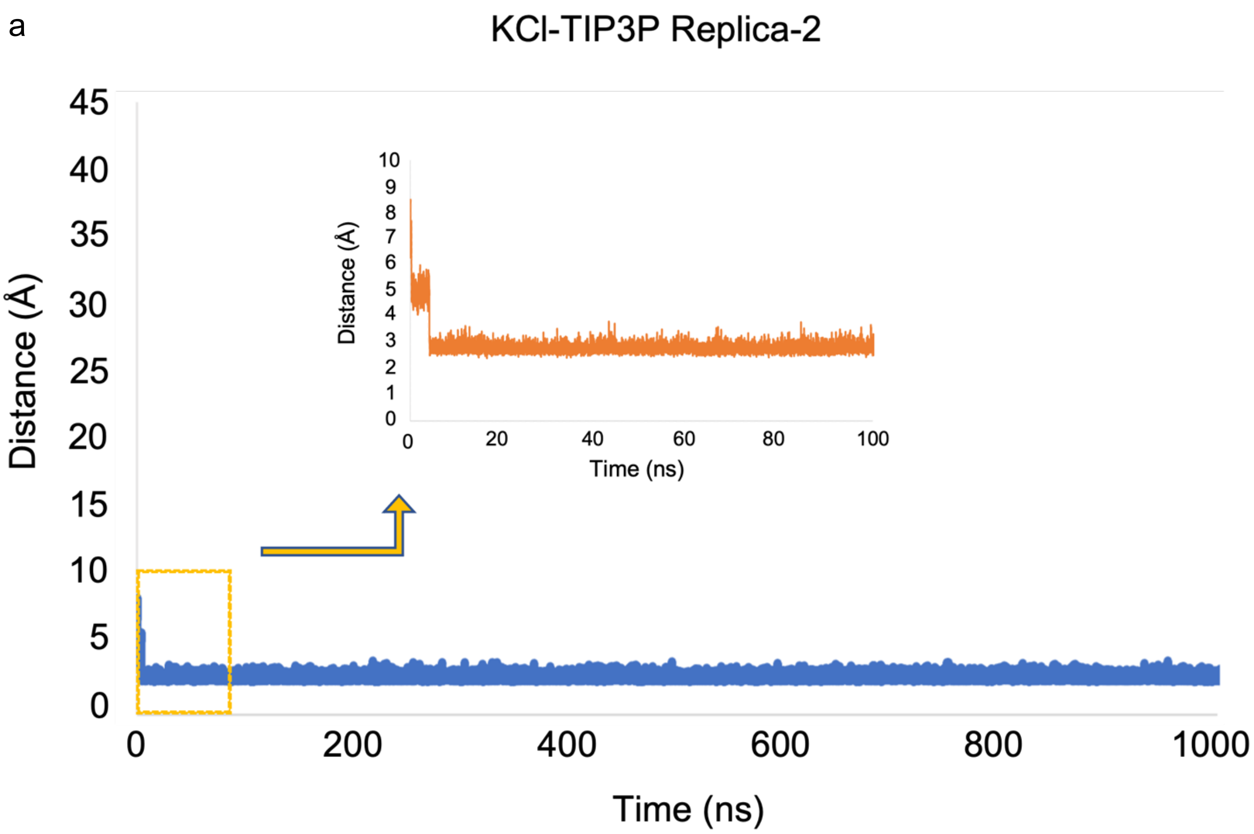

b

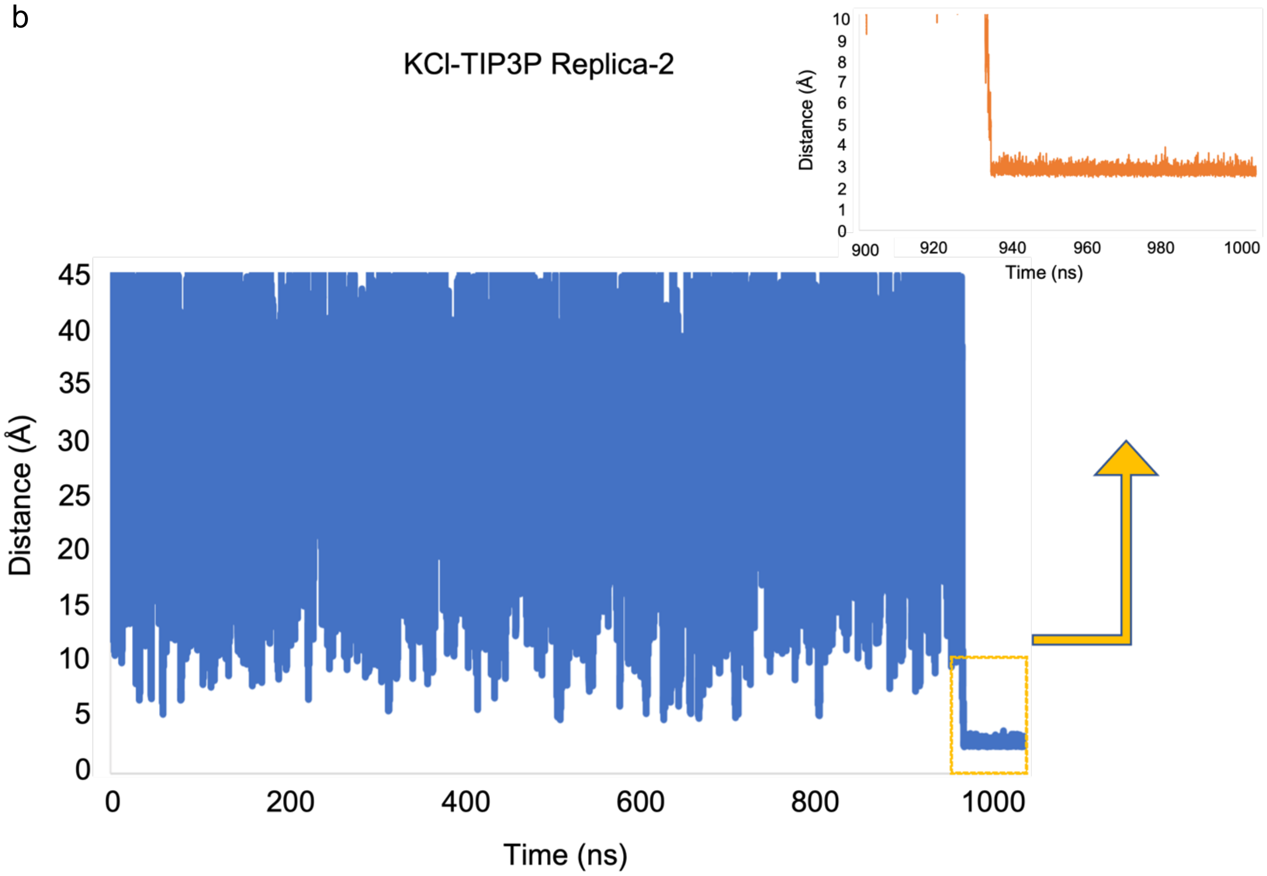

Figure S5: Representative time evolution of distances between G4-O^6 atom and K^+ entering the cavity during KCl-TIP3P replica-2. (a) K^+/G16-O^6 distance (b) K^+/G19-O^6

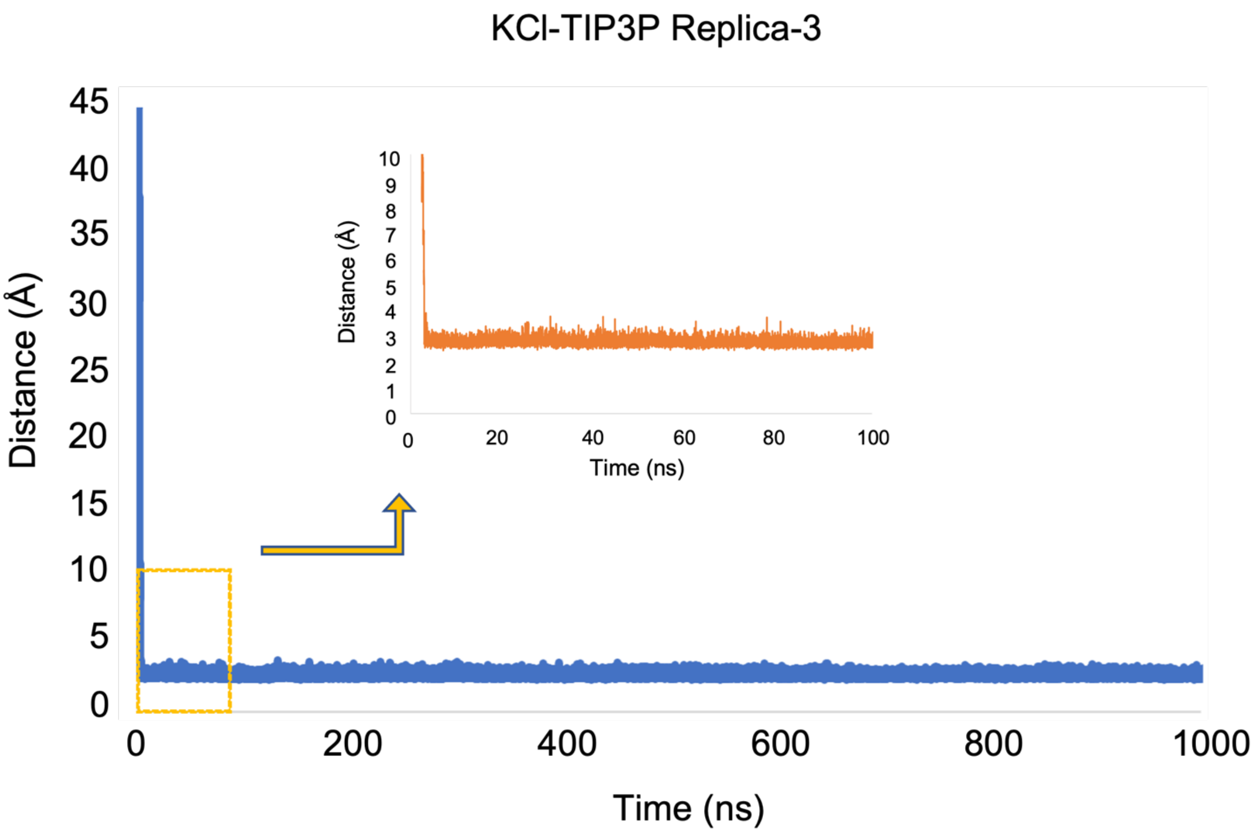

Figure S6: Representative time evolution of distances between G27-O^6 atom and K^+ entering the cavity during KCl-TIP3P replica-1.

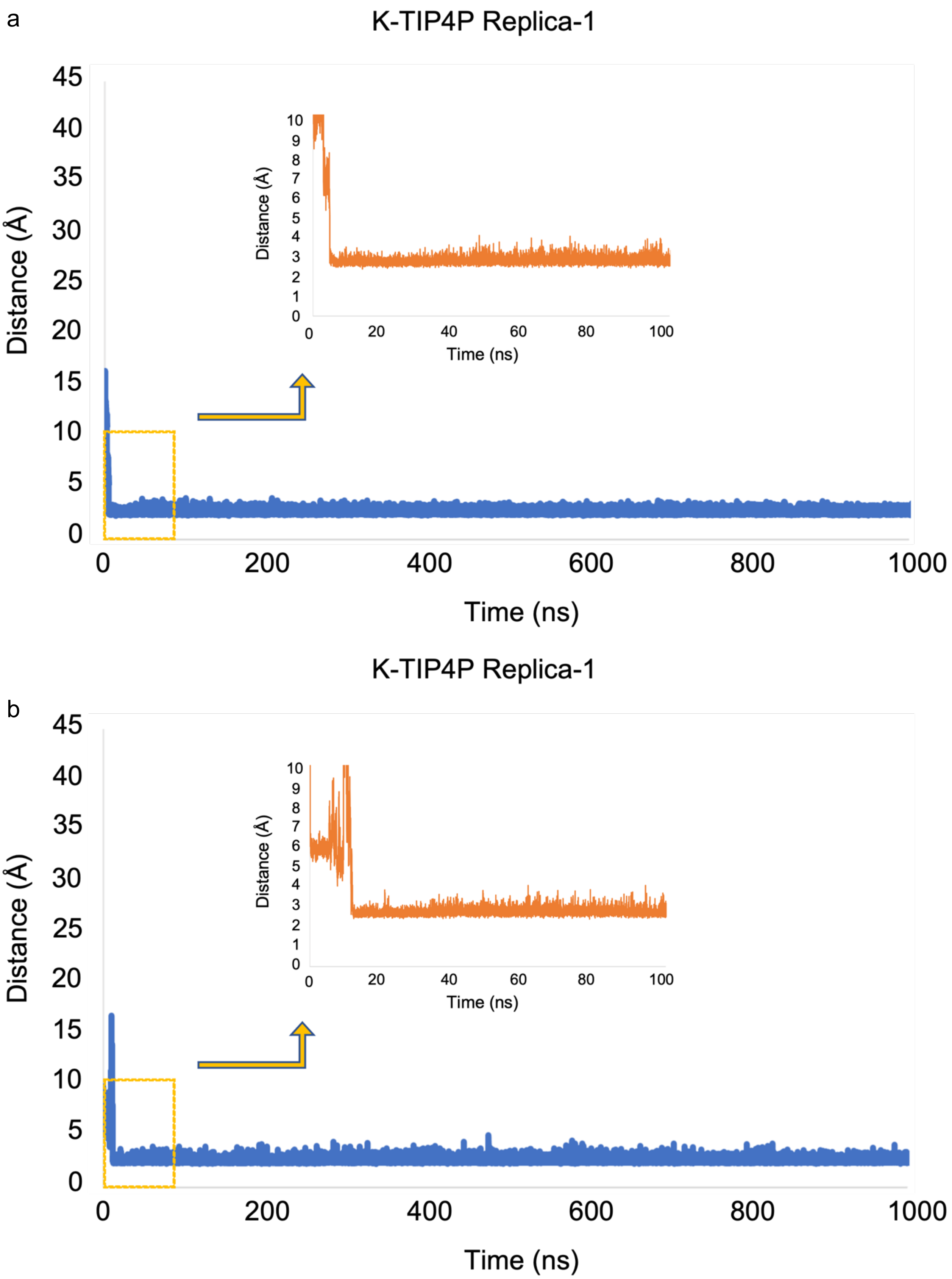

Figure S7: Representative time evolution of distances between G4-O^6 atom and K^+ entering the cavity during K-TIP4P replica-1. (a) K^+/G27-O^6 distance (b) K^+/G19-O^6

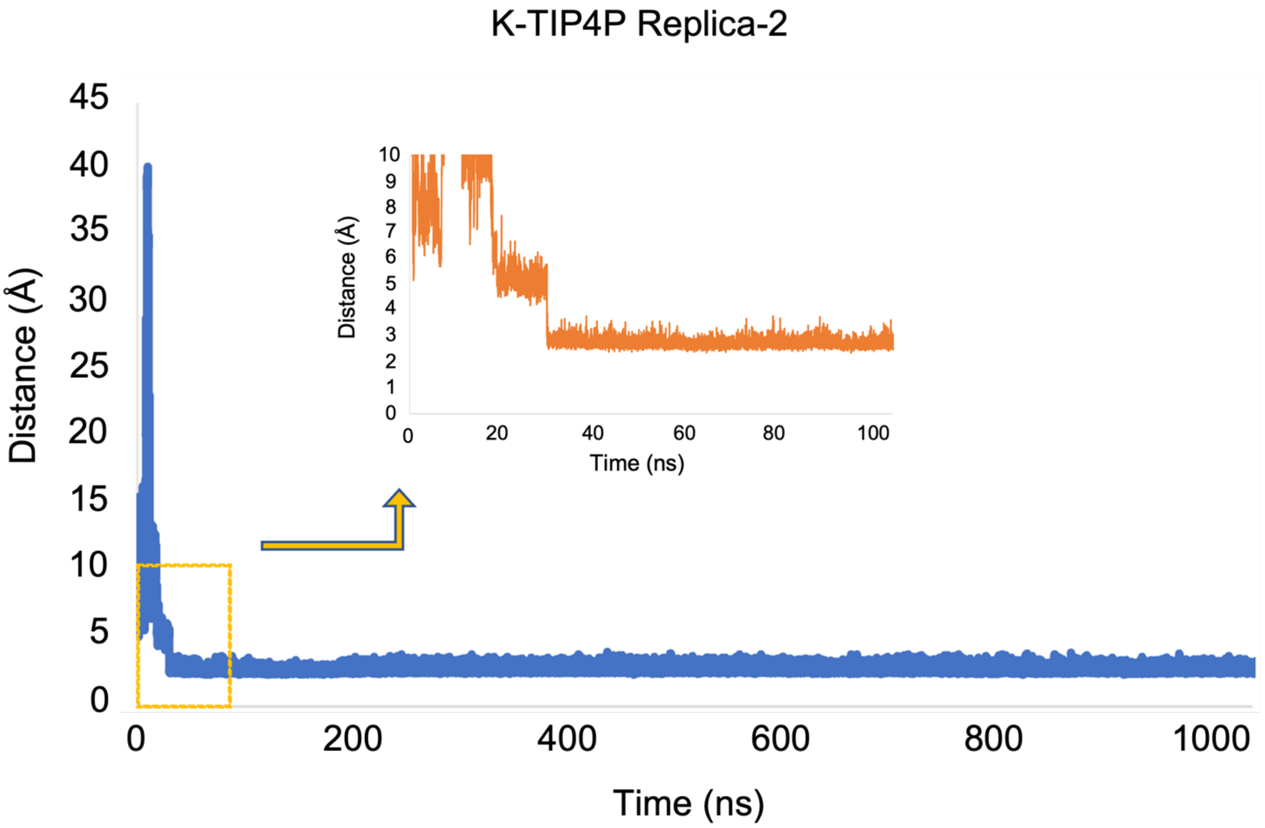

Figure S8: Representative time evolution of distances between G1-O^6 atom and K^+ entering the cavity during K-TIP4P replica-2.

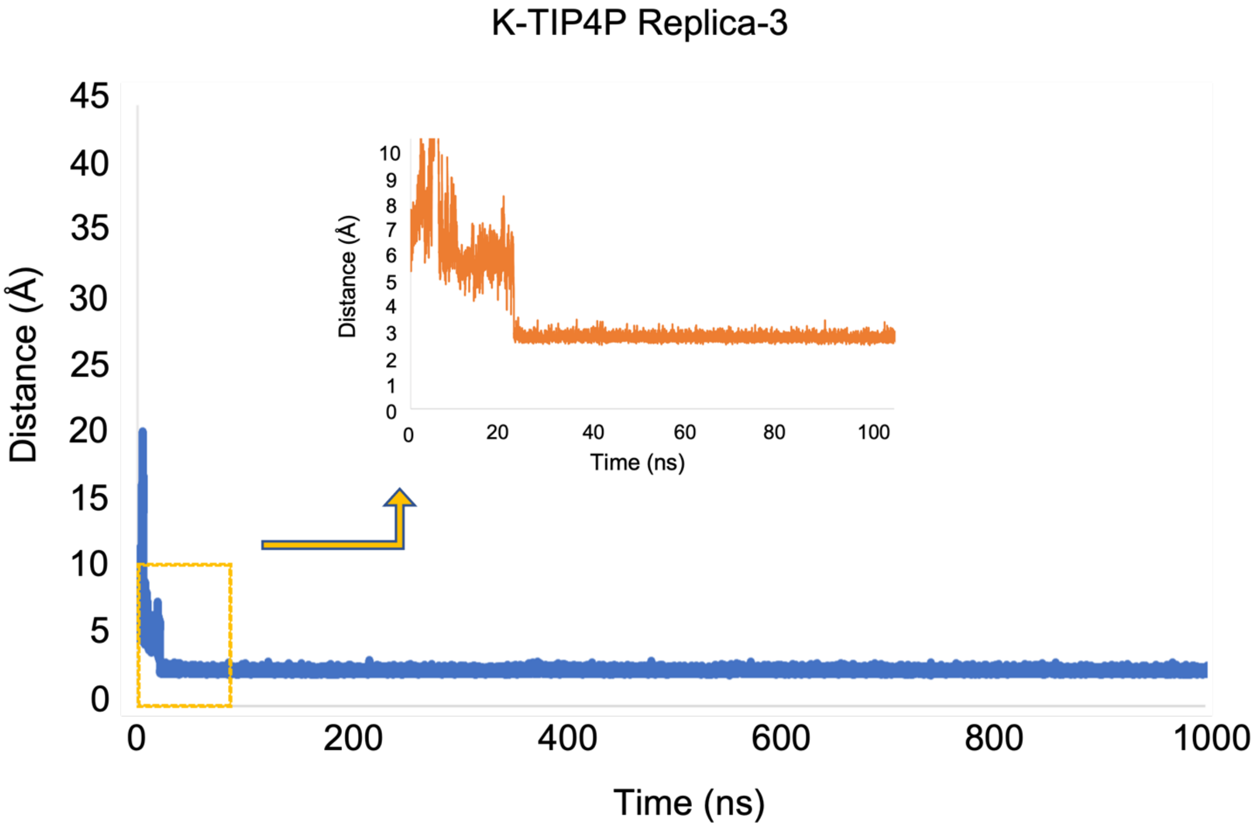

Figure S9: Representative time evolution of distances between G16-O^6 atom and K^+ entering the cavity during K-TIP4P replica-3.

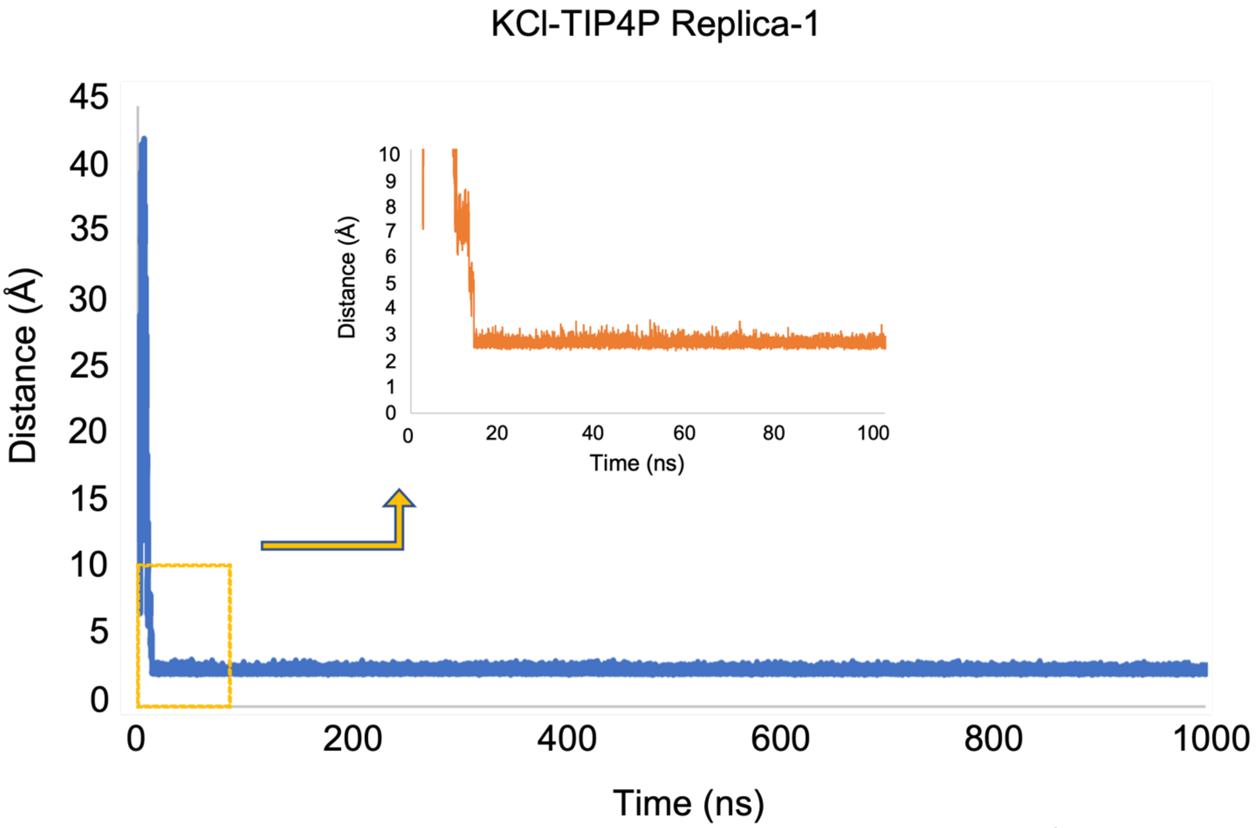

Figure S10: Representative time evolution of distances between G16-O^6 atom and K^+ entering the cavity during KCl-TIP4P replica-1.

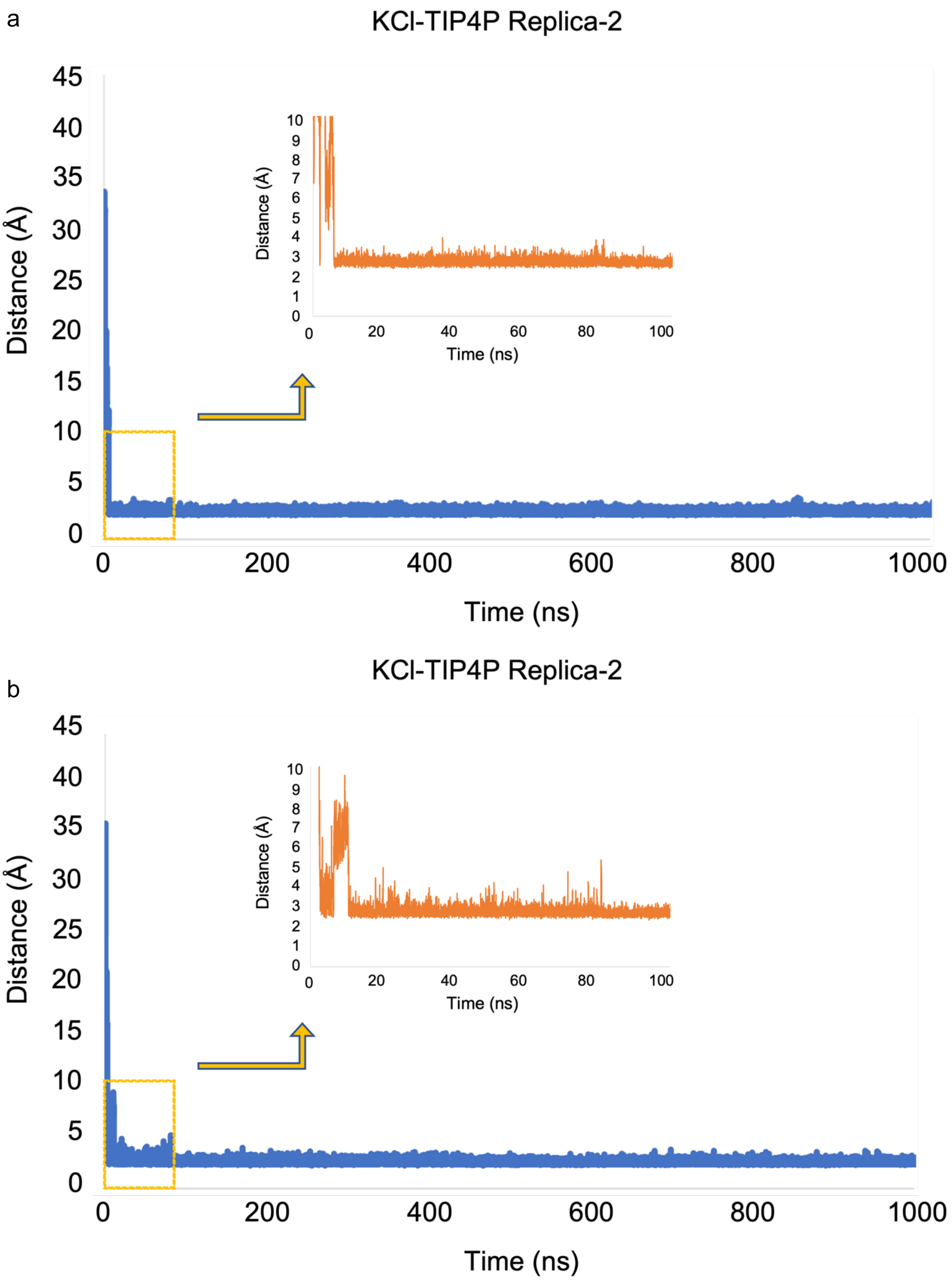

Figure S11: Representative time evolution of distances between G4-O^6 atom and K^+ entering the cavity during KCl-TIP4P replica-2. (a) K^+/G19-O^6 distance (b) K^+/G21-O^6

a

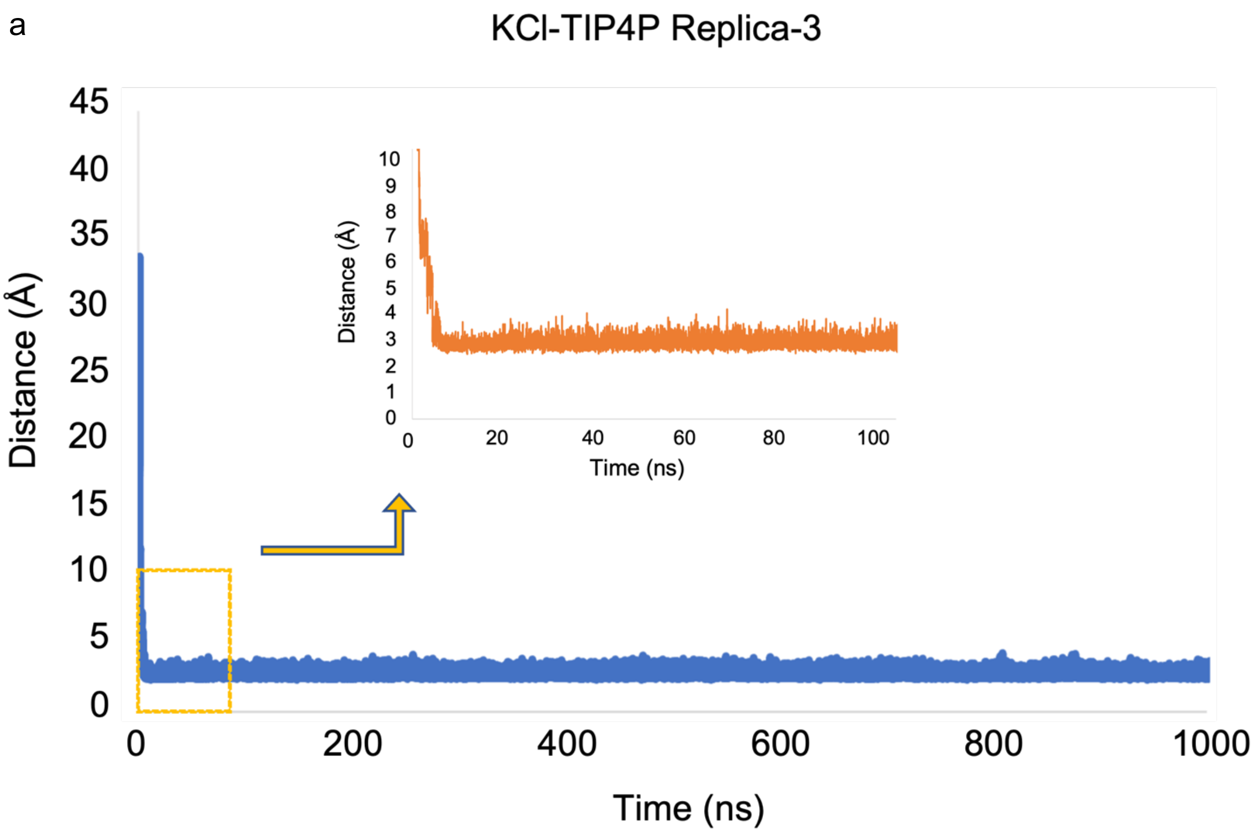

b

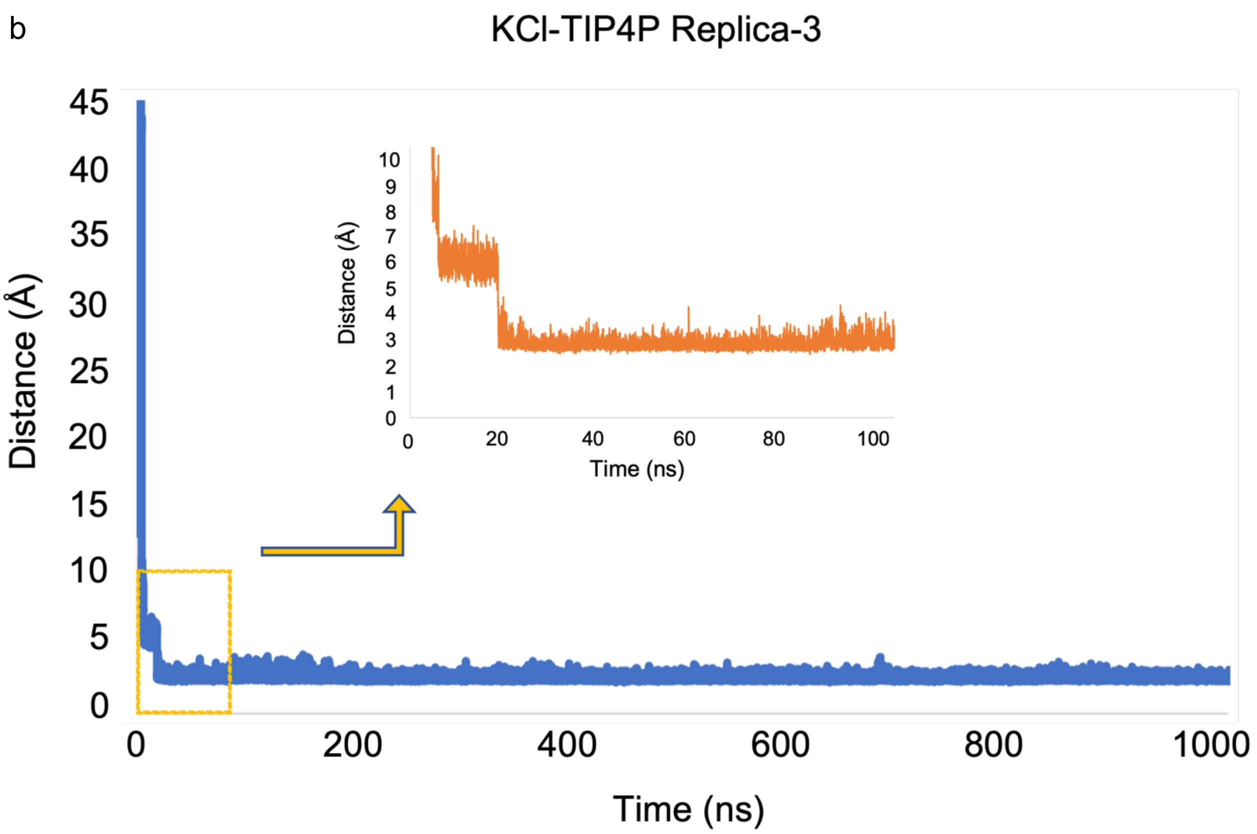

Figure S12: Representative time evolution of distances between G4-O^6 atom and K^+ entering the cavity during KCl-TIP4P replica-3. (a) K^+/G15-O^6 distance (b) K^+/G27-O^6

a

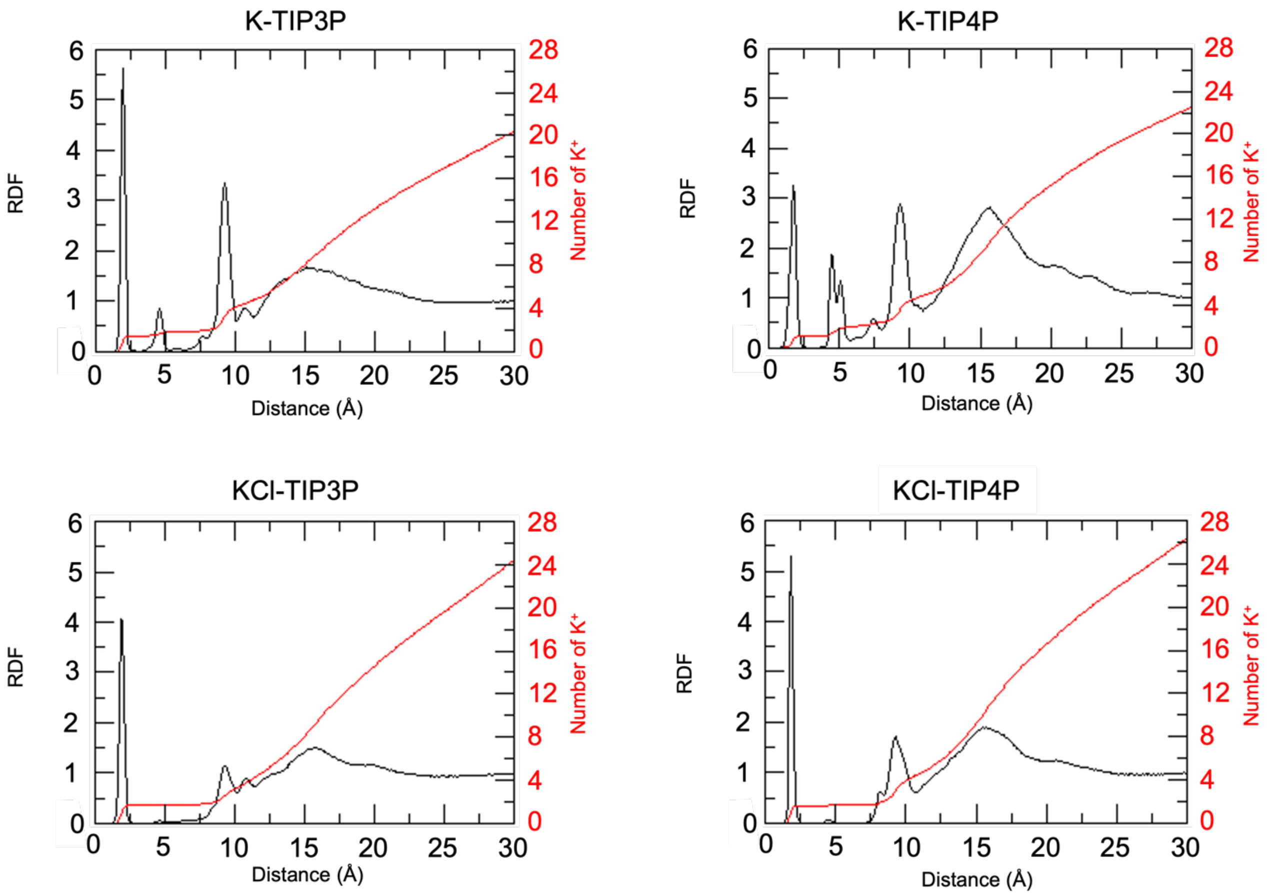

b

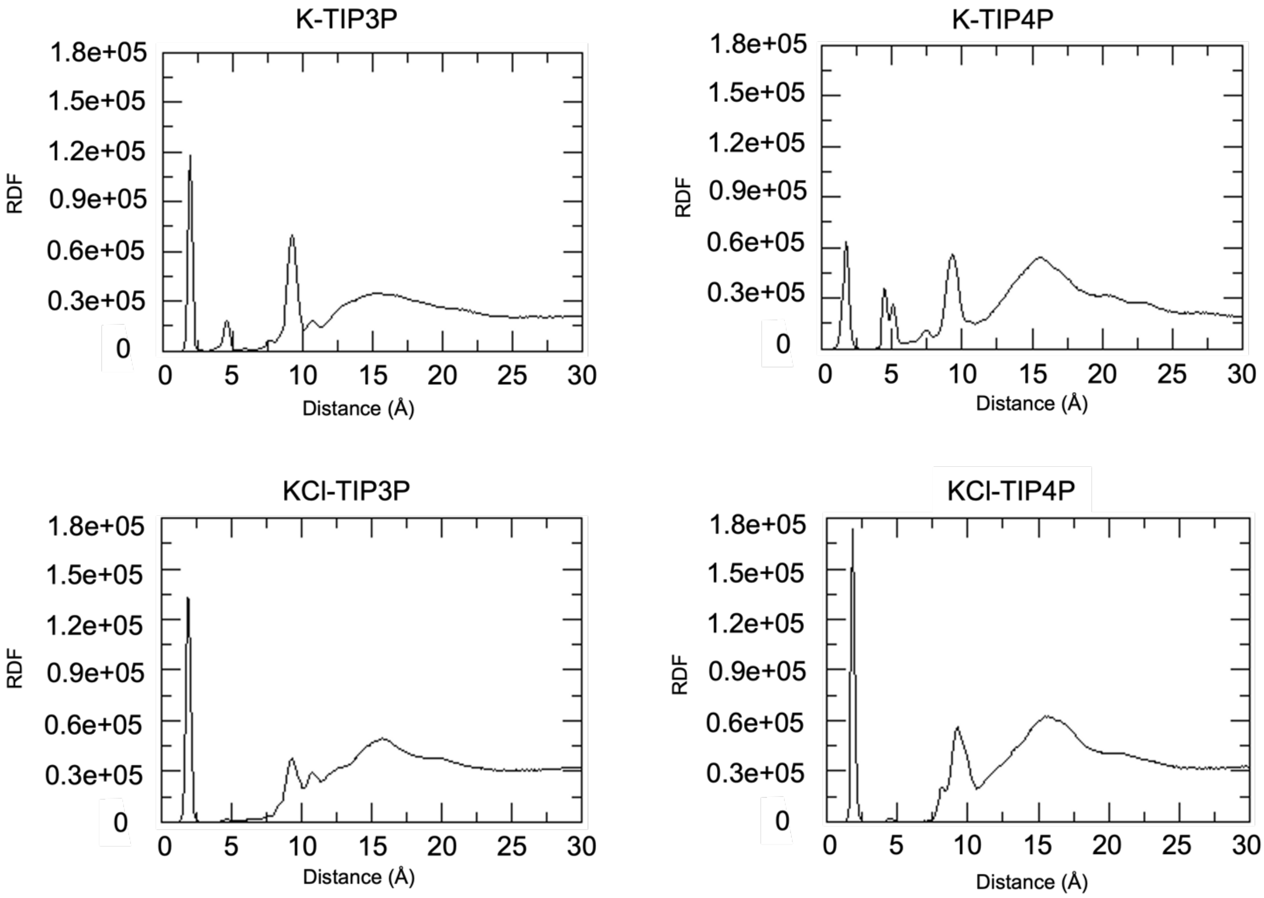

Figure S13: RDFs between O^6-G4 atoms and K^+-ions: (a) Normalized RDFs (b) Raw RDFs.

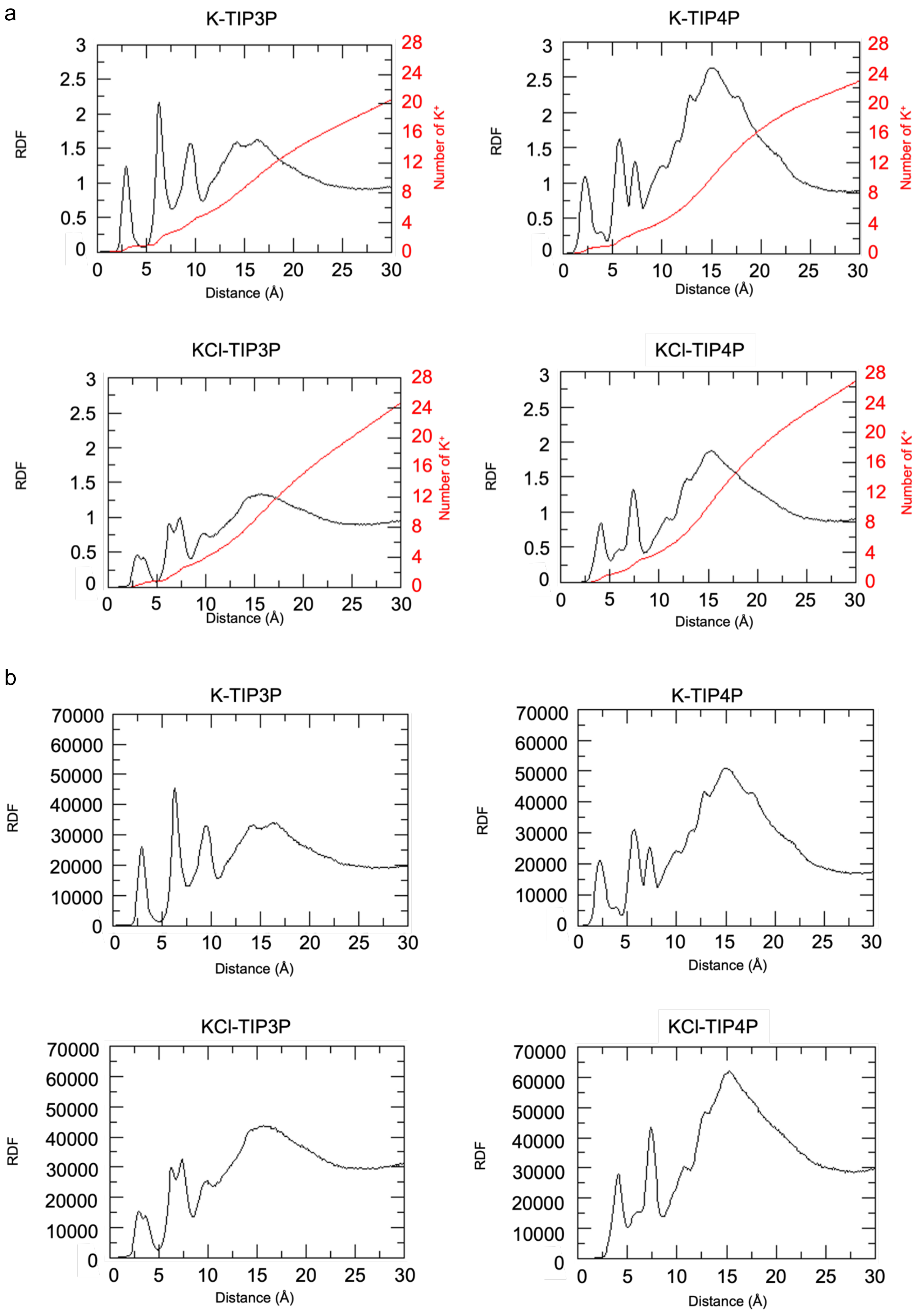

Figure S14: RDFs between backbone OP2 atoms and K^+-ions: (a) Normalized RDFs (b) Raw RDFs.

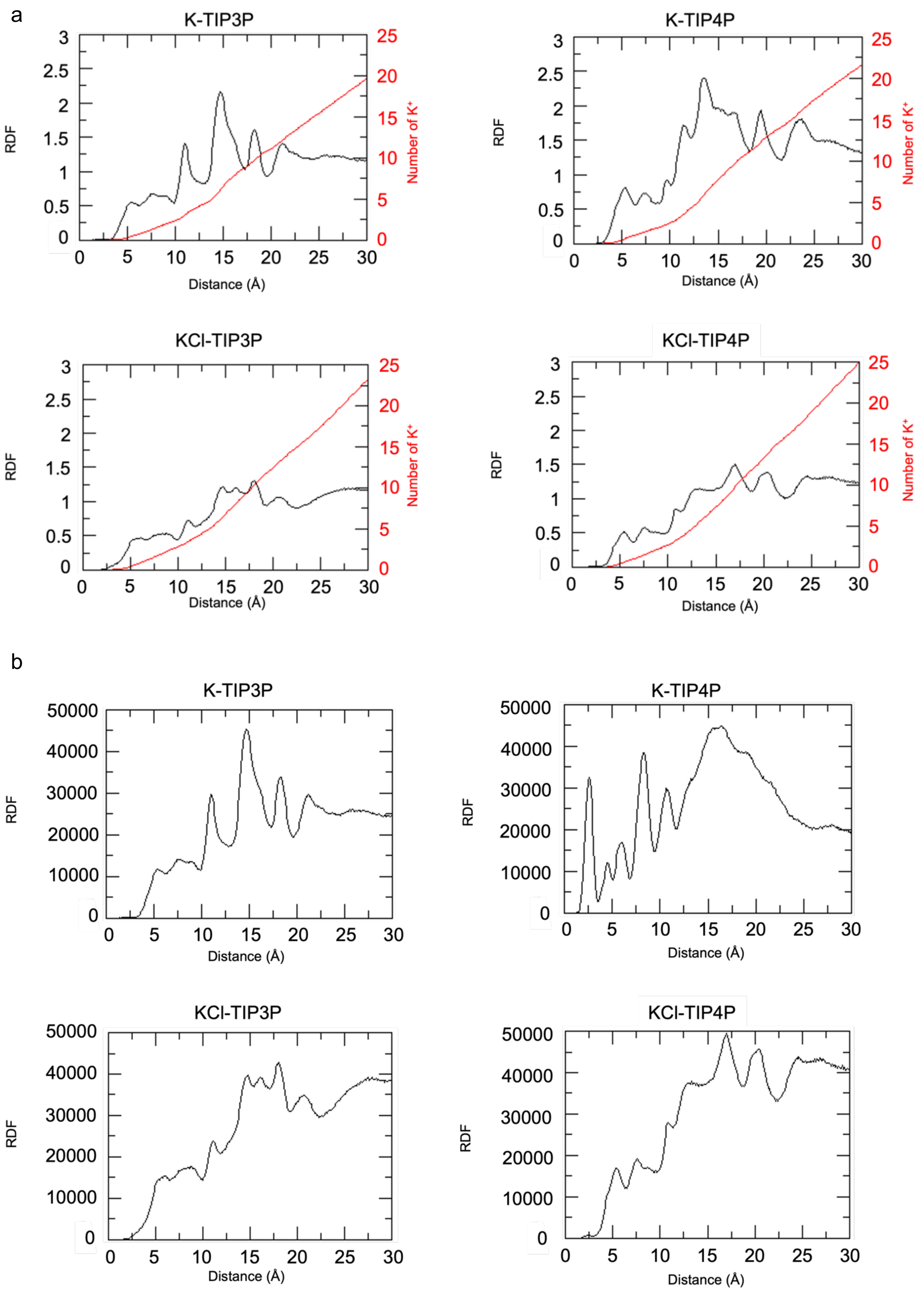

Figure S15: RDFs between duplex-backbone OP2 atoms and K^+-ions: (a) Normalized RDFs (b) Raw RDFs.

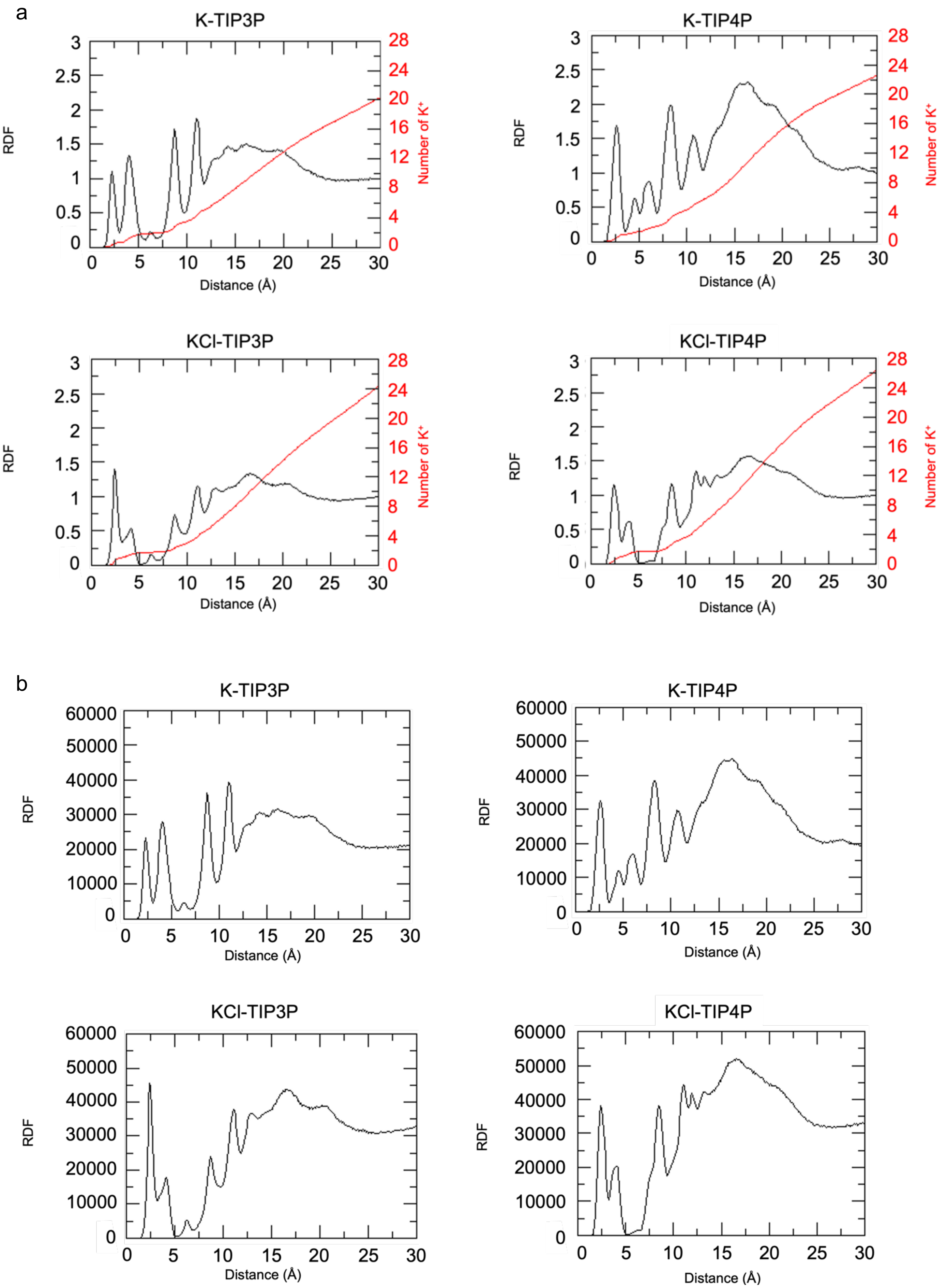

Figure S16: RDFs between G4-backbone OP2 atoms and K^+-ions: (a) Normalized RDFs (b) Raw RDFs.

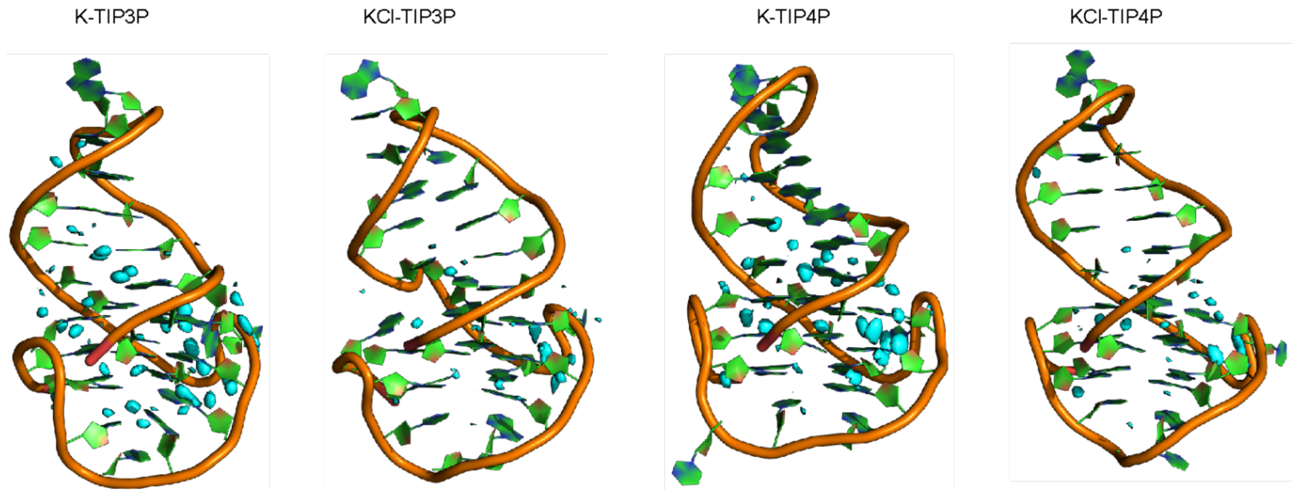

Figure S17: SDFs of water around the most populated cluster for each simulation environment. Water distribution is calculated through a 3D grid and normalized by density (Default particle density for water based on 1.0 g/mL).

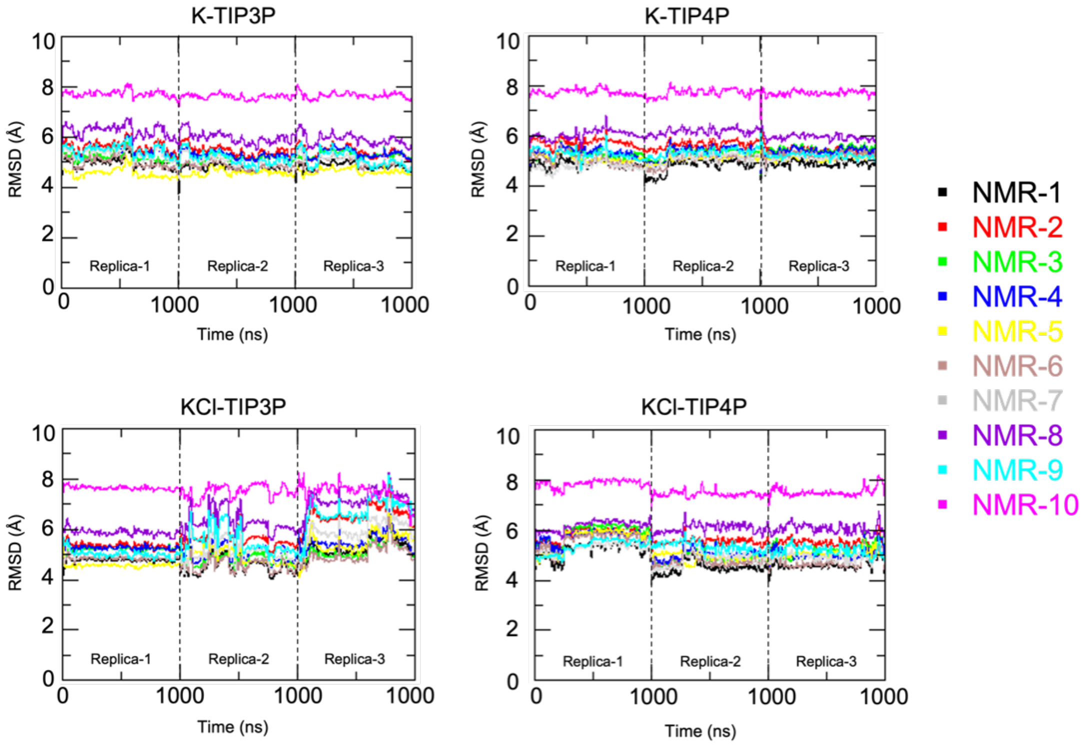

Figure S18: Time evolution of RMSD of all atomic position from the ten structures of NMR bundle during simulation with different solvent environments.

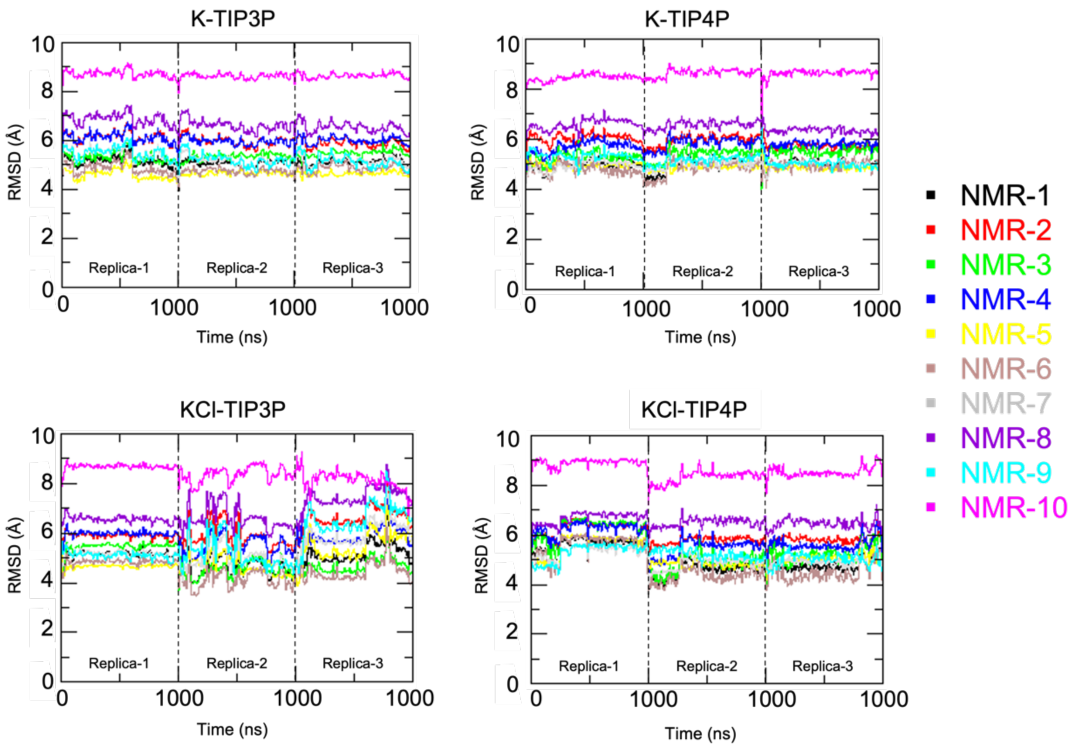

Figure S19: Time evolution of RMSD of backbone atomic position from the ten structures of NMR bundle during simulation with different solvent environments.

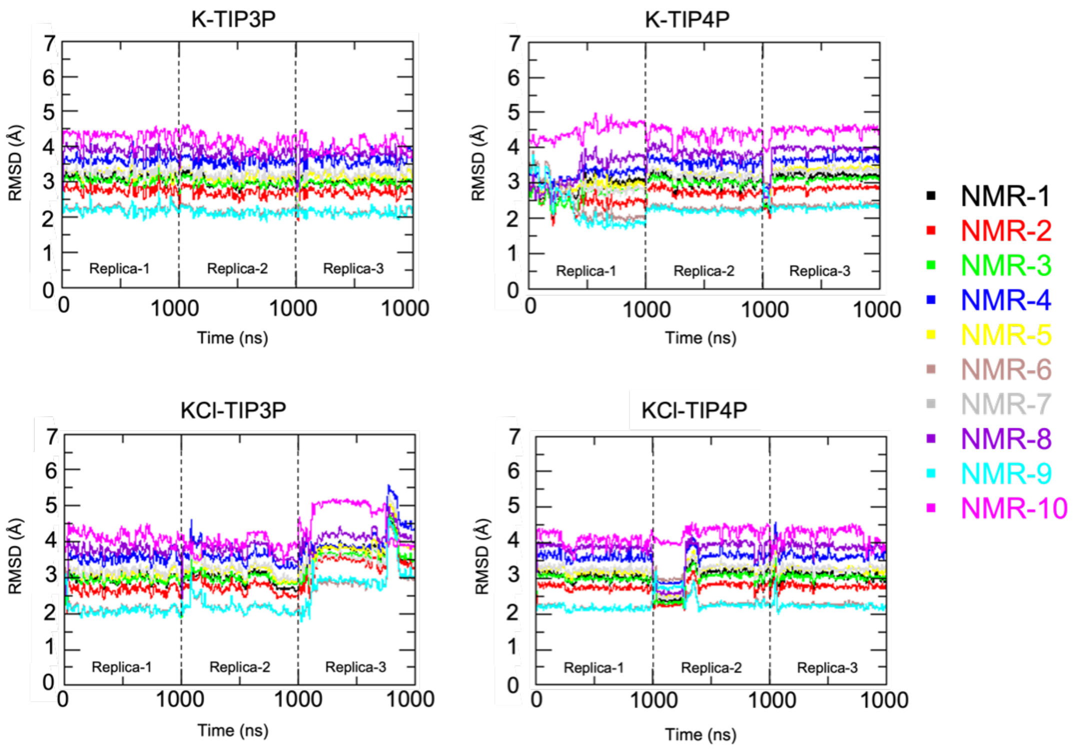

Figure S20: Time evolution of RMSD of duplex-backbone (from residue 5 to residue 13) atomic position from the ten structures of NMR bundle during simulation with different solvent environments.

- H-bond analysis:

We analyzed the distribution of the distances and angles which describe H-bond formation between bases. We differentiated the analysis between adjacent guanines of the three tetrads in the G4 (Figures S21 to S38) and three base-pairs of guanine-cytosine in the duplex (Figures S39 to S44), and among solvent environments.
Each figure is identified by the title reporting the specific quantity being reported and single panel refers to the reported simulation conditions. The distribution obtained from MD simulation was compared with the average value from NMR bundle (vertical red line).

- Dihedral angles:

We computed the time distribution of dihedrals in the 28 deoxyribonucleotides forming the simulated system and compared with experimental values of the 10 NMR structures available in 6H1K. The distributions were obtained using a bin size of 15° and afterward normalized. The results have been reported in a compact matrix representation (Figures S45 to S72).
Each figure is identified by the title reporting the specific residue being considered and the simulation conditions. Each panel describes the 6 monitored dihedrals represented by the 6 columns, while the rows correspond to the clustered torsion angle values. The color intensity reflects the percentage of occupation of each dihedral obtained from MD simulations, while the black dots indicate the corresponding torsion angle value in each of the 10 NMR structures.

NOE Violations										
K-TIP3P			KCl-TIP3P			K-TIP4P			KCl-TIP4P	
Res A	Res B		Res A	Res B		Res A	Res B		Res A	Res B
G2-H1'	G3-H8		G10-H1’	T9-H4'		G3-H8	G3-H1'		G5-H8	A4-H2''
G5-H8	A4-H2'		G5-H8	A4-H2''		G5-H8	A4-H2''		T14-H6	C13-H2''
G10-H8	G10-H2'		C7-H6	G8-H8		G8-H8	C7-H2'		C7-H6	G8-H8
T14-H6	C13-H2''		G10-H8	G10-H3'		T14-H6	C13-H2''		G10-H8	G10-H3'
C7-H6	G8-H8		C13-H6	T14-H3'		C23-H6	G21-H2'		C13-H6	T14-H3'
G10-H8	G10-H3'		G3-H8	A4-H4'		C7-H6	G8-H8		G10-H8	T9-H4'
C13-H6	T14-H3'		G8-H1’	T9-H1'		G3-H8	G2-H3'		G10-H1'	T9-H4'
G3-H8	A4-H4'					G10-H8	G10-H3'		T14-H6	G15-H1
G8-H8	C7-H4'					C13-H6	T14-H3'			
G10-H8	T9-H4'					G10-H1'	T9-H4'			
G10-H1’	T9-H4'					T14-C7	G19-H1			
T14-C7	G19-H1					T14-H6	G15-H1			
T14-H6	G15-H1					T24-C7	G21-H1			
T24-C7	G28-H1									

Table S1: Summary of NOE violations from simulations. For each solvent environment the atom couple involved in violations are reported.

NMR Model	Number of Violations
NMR-1	0
NMR-2	0
NMR-3	0
NMR-4	0
NMR-5	1 (G3-H1’/A4-H4')
NMR-6	0
NMR-7	0
NMR-8	0
NMR-9	1 (C13-H2” T14-H5')
NMR-10	0

Table S2: Summary of NOE violations from NMR-bundle. For each NMR structure is reported the number of violations and eventually the atoms involved.

a

H-bond persistence												
	K-TIP3P			KCl-TIP3P			K-TIP4P			KCl-TIP4P		
	tet1	tet2	tet3	tet1	tet2	tet3	tet1	tet2	tet3	tet1	tet2	tet3
N2-N7	42,50%	58,60%	41,20%	57,90%	68,10%	31,80%	46,70%	64,20%	46,80%	56,20%	65,60%	43,20%
O6-N1	61,50%	43,10%	73,70%	65,60%	49,10%	42,40%	41,70%	66,80%	52,60%	64,00%	43,80%	57,20%
N1-N7	0,02%	0,30%	0,01%	0,01%	0,05%	0,23%	0,2%	0,02%	0,03%	0,01%	0,20%	0,04%

b

H-bond persistence				
	K-TIP3P	KCl-TIP3P	K-TIP4P	KCl-TIP4P
	duplex	duplex	duplex	duplex
O6-N4	73,20%	46,00%	63,50%	71,10%
N2-O2	82,00%	53,60%	81,40%	80,60%
N1-N3	71,40%	44,70%	71,40%	69,60%

Table S3: H-bond persistence is reported as percentage over the entire trajectory. Each H-bond type is identified by the participating acceptor and donor heavy atoms (a) G4 portion is defined as: tet1 (res 2-15-19-26), tet2 (res 1-16-20-27) and tet3 (res 17-21-25-28) (b) Duplex refers to G-C pairing of residues 13C-5G, 12C-21G and 11G-7G.

Bifurcated H-bond persistence				
	K-TIP3P	KCl-TIP3P	K-TIP4P	KCl-TIP4P
	bifurcated	bifurcated	bifurcated	bifurcated
tet1	0,006%	0,004%	0,087%	0,001%
tet2	0,072%	0,024%	0,008%	0,095%
tet3	0,004%	0,048%	0,018%	0,015%

Table S4: Bifurcated N1–N7/N2-N7 H-bond persistence is represented as percentage over the entire trajectory. Tetrads are defined as: tet1 (res 2-15-19-26), tet2 (res 1-16-20-27) and tet3 (res 17-21-25-28).

- Free Energy estimation:

We computed an approximate estimation of the free energy for ion binding in each simulated system/solvent environment. The equilibrium constant was derived from counting the representation of two states, namely bound and unbound, along the trajectories. States were defined as bound if the distances between the K^+ and the O^6 atoms of the interacting guanines are less than 3 Å; when this distance is greater than 3 Å the configuration is considered part of the unbounded population. Gibbs free energy was calculated as: $\Delta G = -RTln(K_{eq})$, with K_{eq} representing the ratio between bound and unbound state counts. The results are reported in Table S5. Favorable ion binding was observed for each solvent environment. Small differences were seen in different environments.

Ion Binding				
System	K-TIP3P	KCl-TIP3P	K-TIP4P	KCl-TIP4P
ΔG (kJ/mol)	-1,70	-1,38	-1,01	-2,90

Table S5: Free energy barrier for ion binding process is roughly estimated from the distribution of the bound and unbound states along the trajectories.
