# Supplementary figures and images for: Studying the Dynamics of a Complex G-Quadruplex System: Insights into the Comparison of MD and NMR Data
Source: J Chem Theory Comput. 2022 Jun 6;18(7):4515–28. doi: 10.1021/acs.jctc.2c00291 (PMC9281369; doi:10.1021/acs.jctc.2c00291)

# Distance G4 tetrad-1 N2-N7

## K-TIP3P

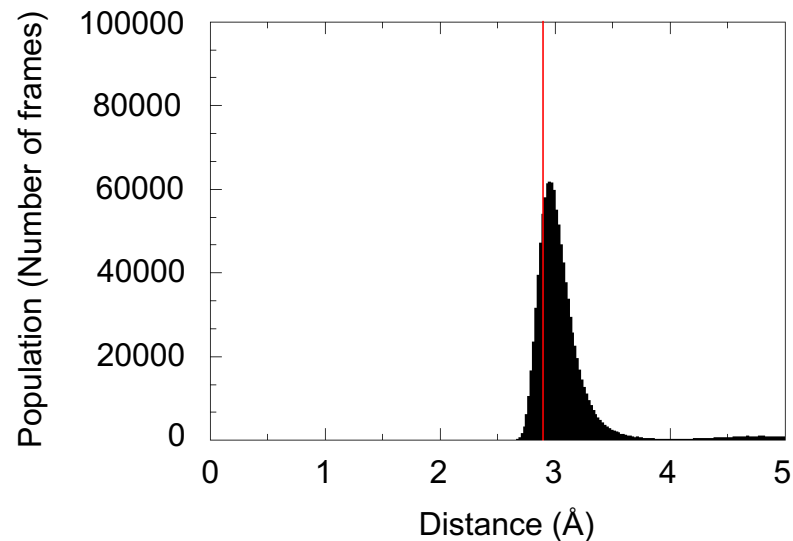

## K-TIP4P

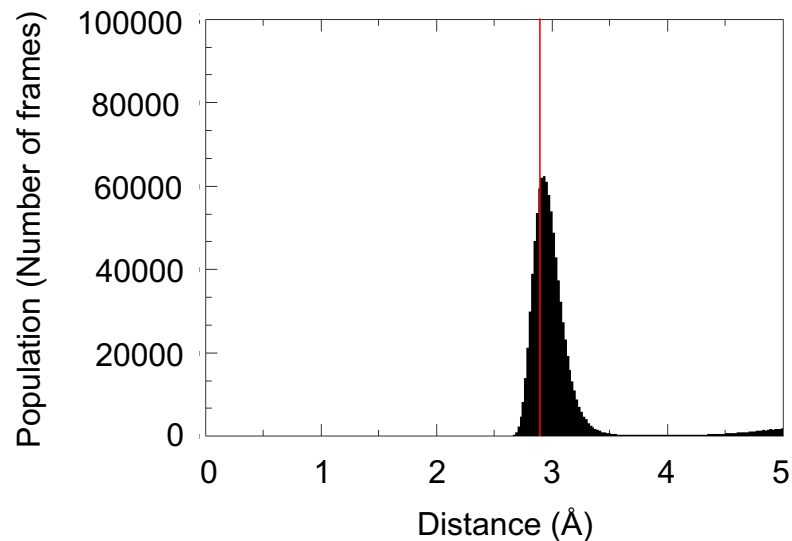

## KCI-TIP3P

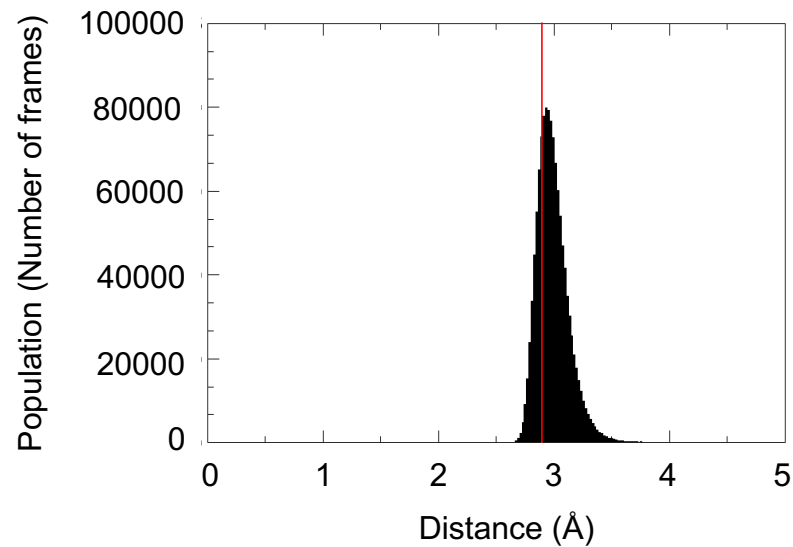

## KCI-TIP4P

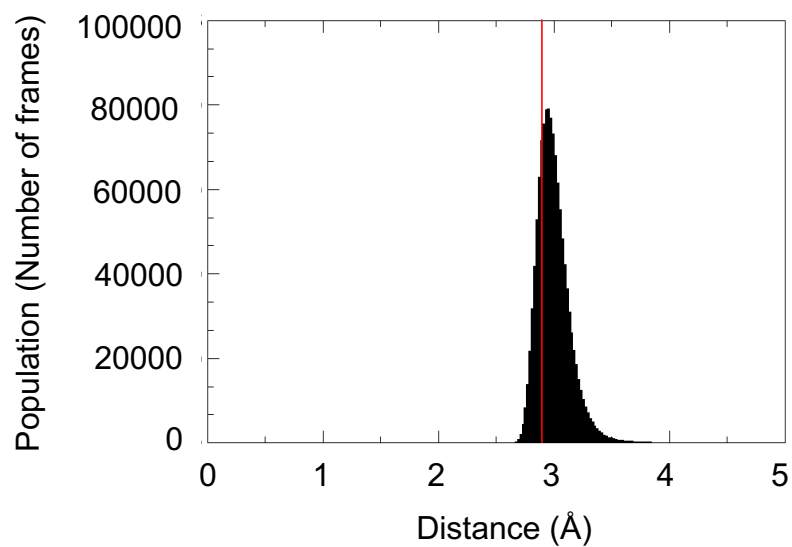

Supplement: Supplementary file 2 — ct2c00291_si_002.zip [file ct2c00291_si_002.zip › Figure S21.pdf]

# Angle G4 tetrad-1 N2-N7

K-TIP3P

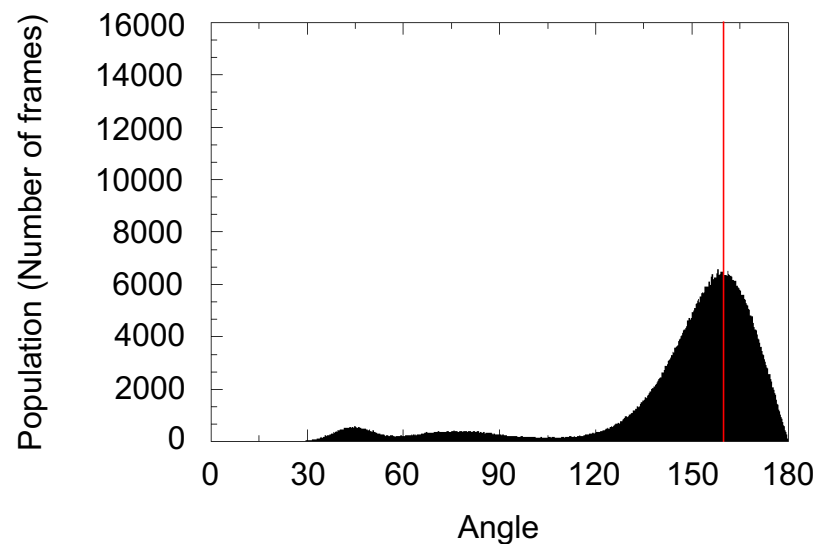

K-TIP4P

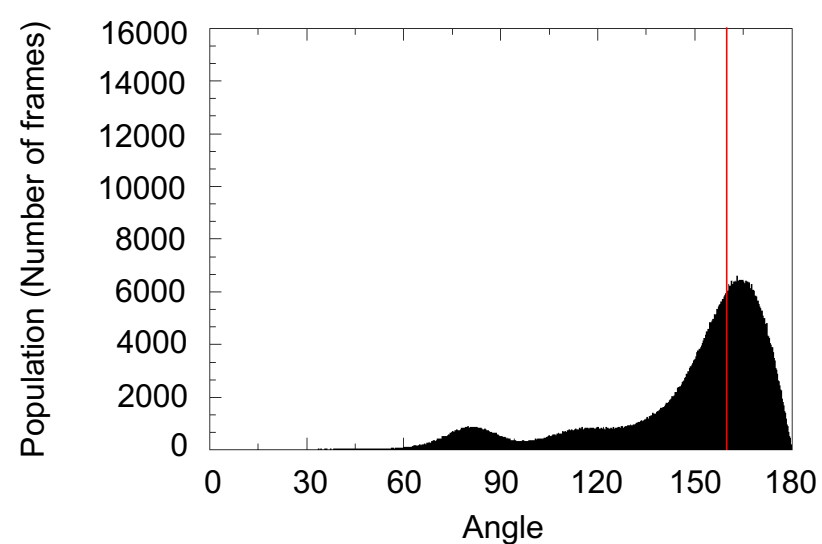

KCI-TIP3P

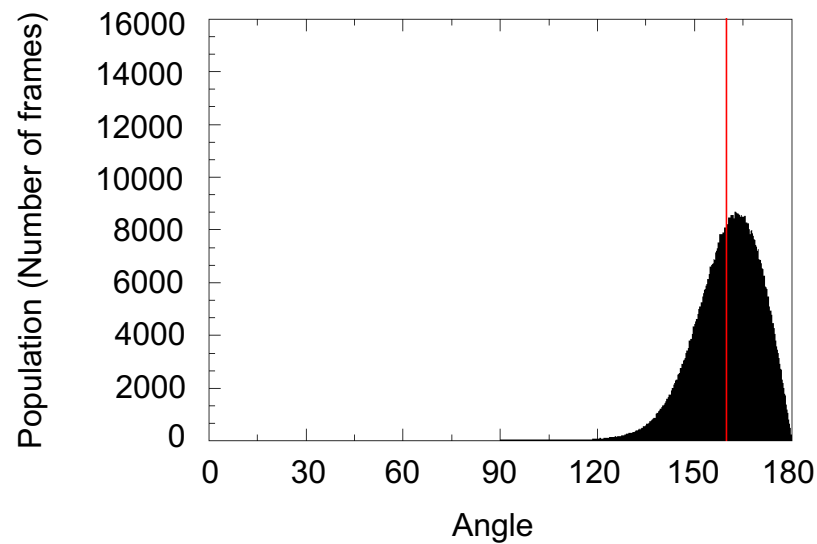

KCI-TIP4P

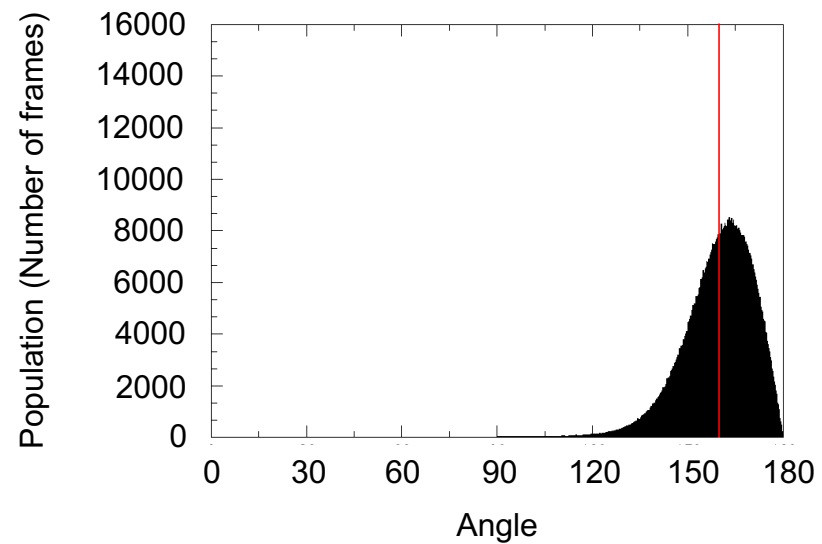

Supplement: Supplementary file 2 — ct2c00291_si_002.zip [file ct2c00291_si_002.zip › Figure S22.pdf]

# Distance G4 tetrad-2 N2-N7

K-TIP3P

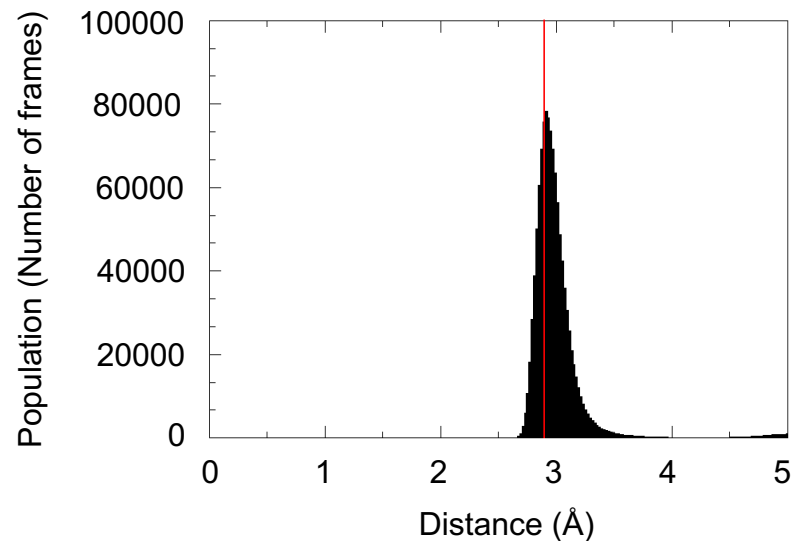

K-TIP4P

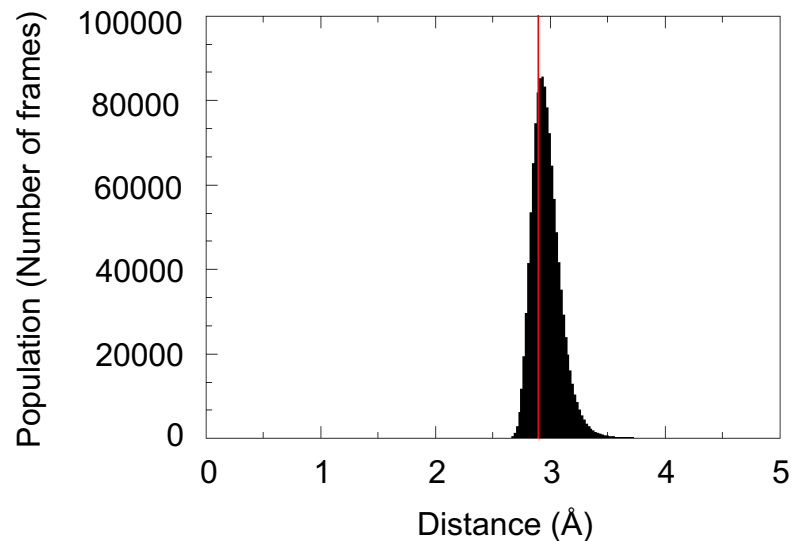

KCI-TIP3P

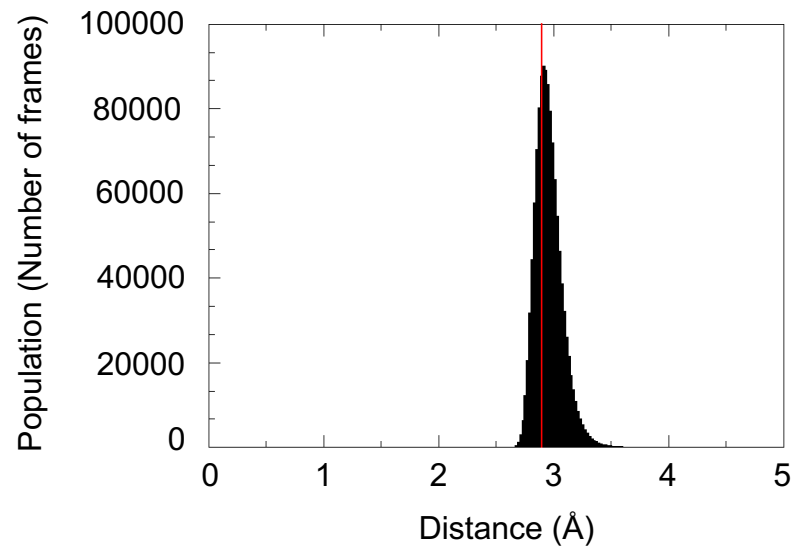

KCI-TIP4P

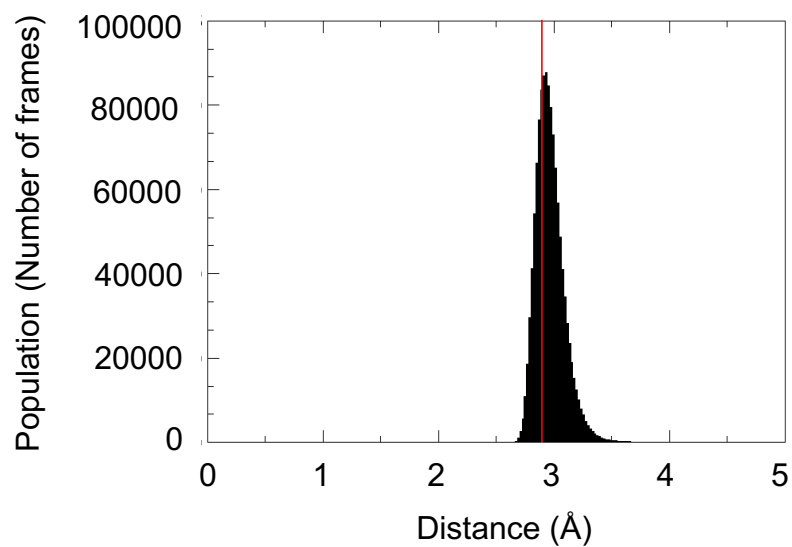

Supplement: Supplementary file 2 — ct2c00291_si_002.zip [file ct2c00291_si_002.zip › Figure S23.pdf]

# Angle G4 tetrad-2 N2-N7

K-TIP3P

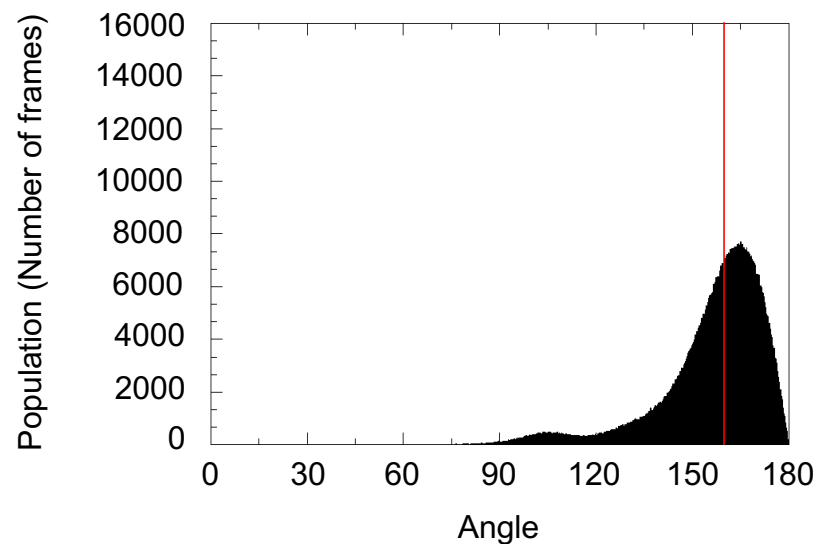

K-TIP4P

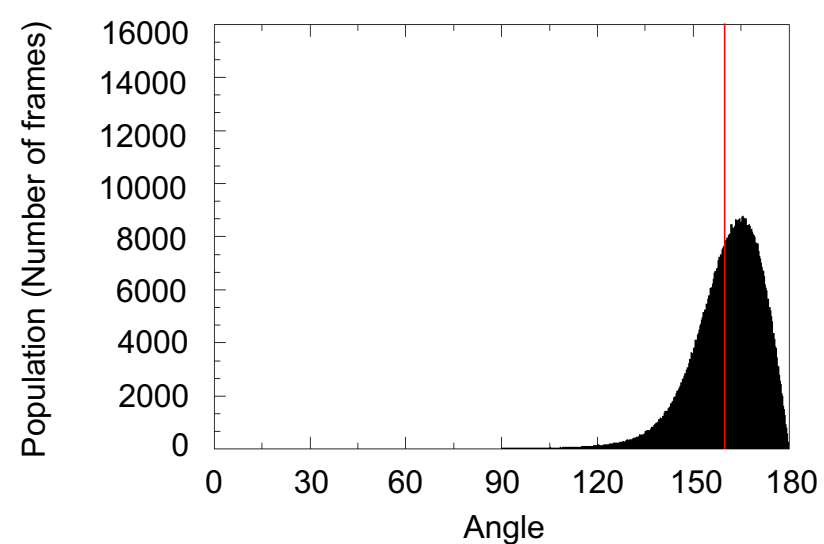

KCI-TIP3P

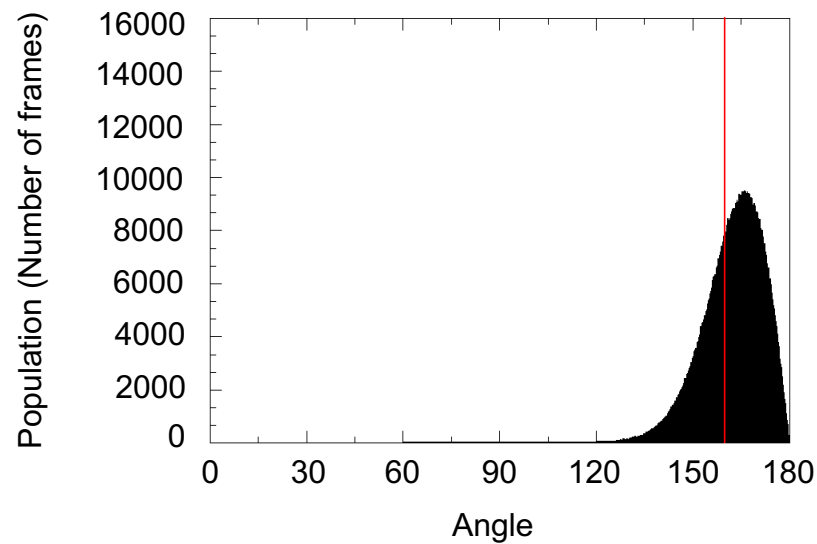

KCI-TIP4P

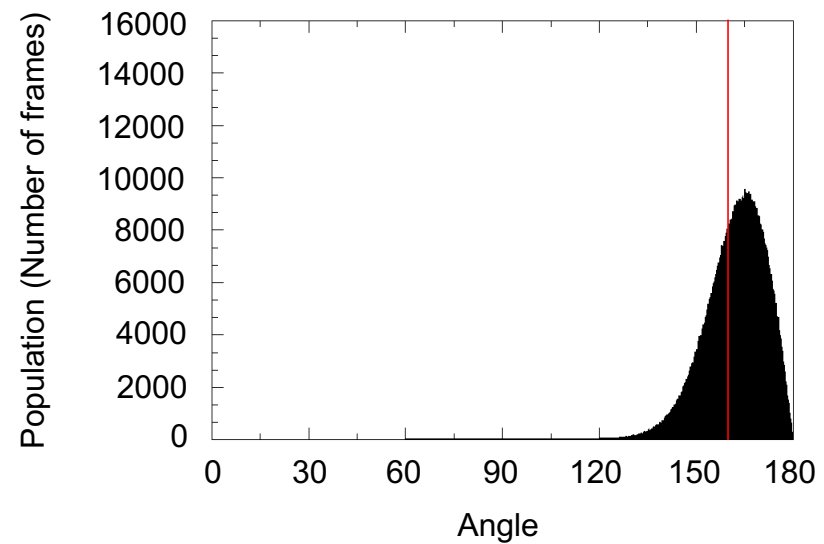

Supplement: Supplementary file 2 — ct2c00291_si_002.zip [file ct2c00291_si_002.zip › Figure S24.pdf]

# Distance G4 tetrad-3 N2-N7

K-TIP3P

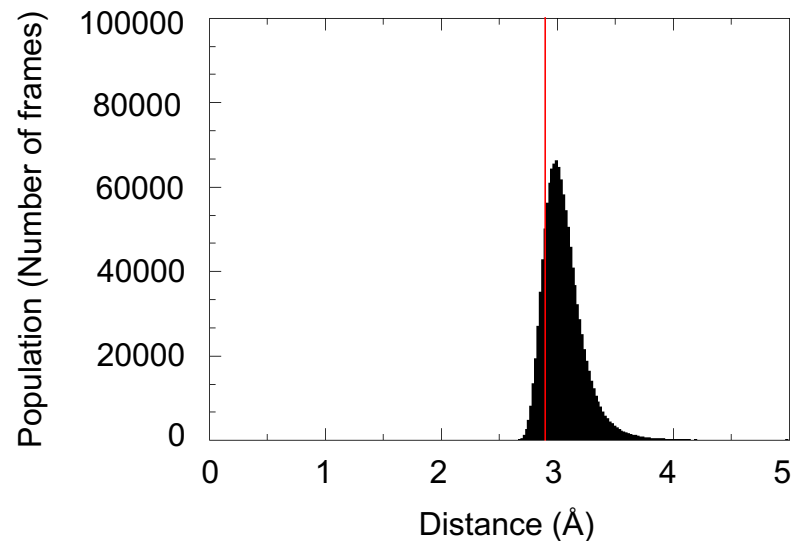

K-TIP4P

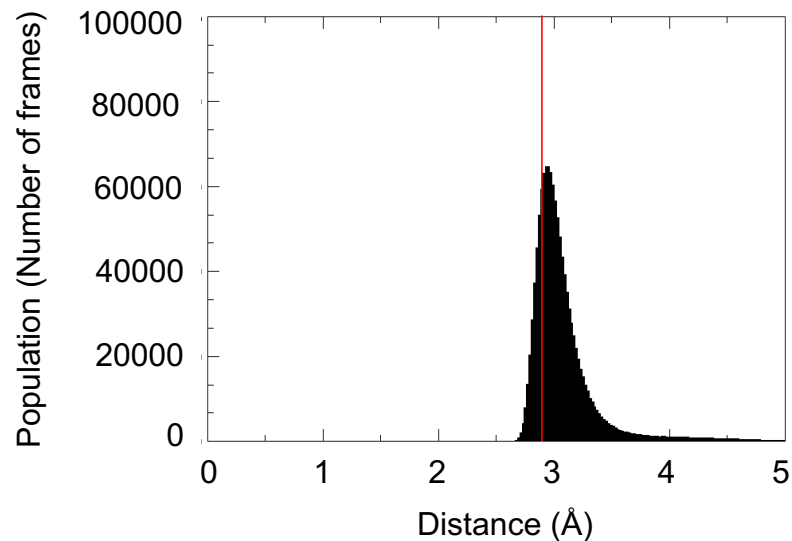

KCI-TIP3P

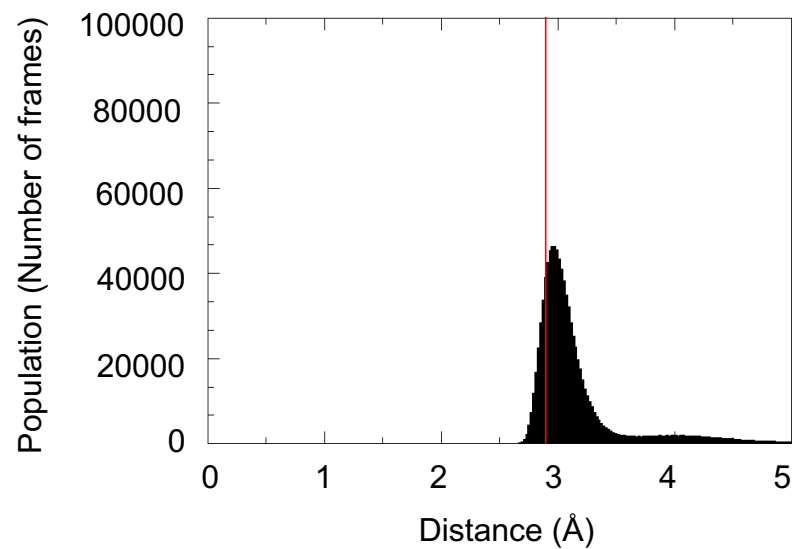

KCI-TIP4P

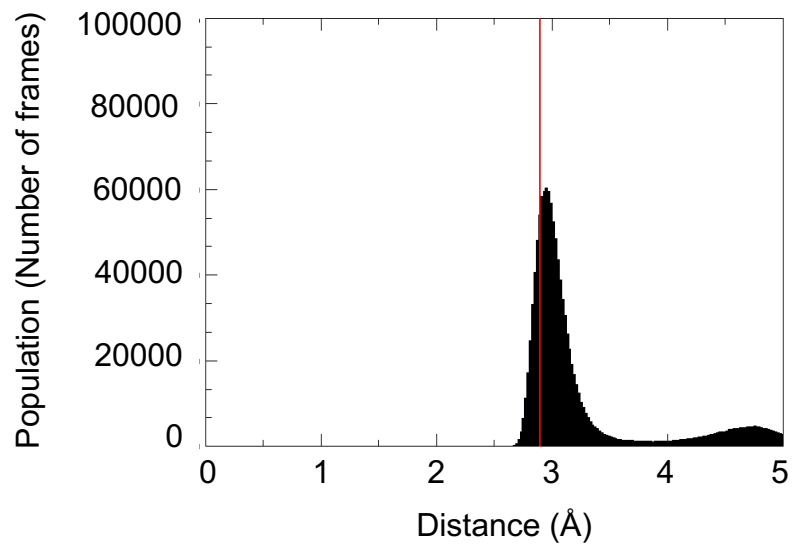

Supplement: Supplementary file 2 — ct2c00291_si_002.zip [file ct2c00291_si_002.zip › Figure S25.pdf]

# Angle G4 tetrad-3 N2-N7

## K-TIP3P

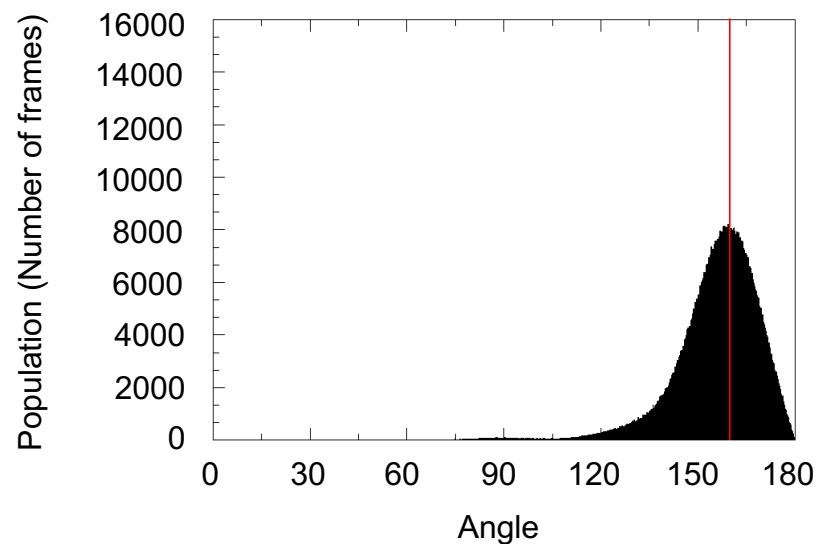

## K-TIP4P

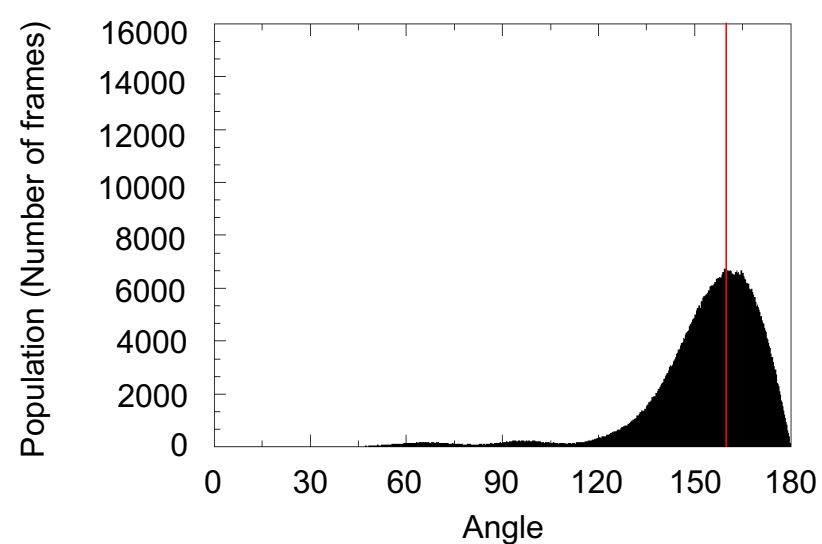

## KCI-TIP3P

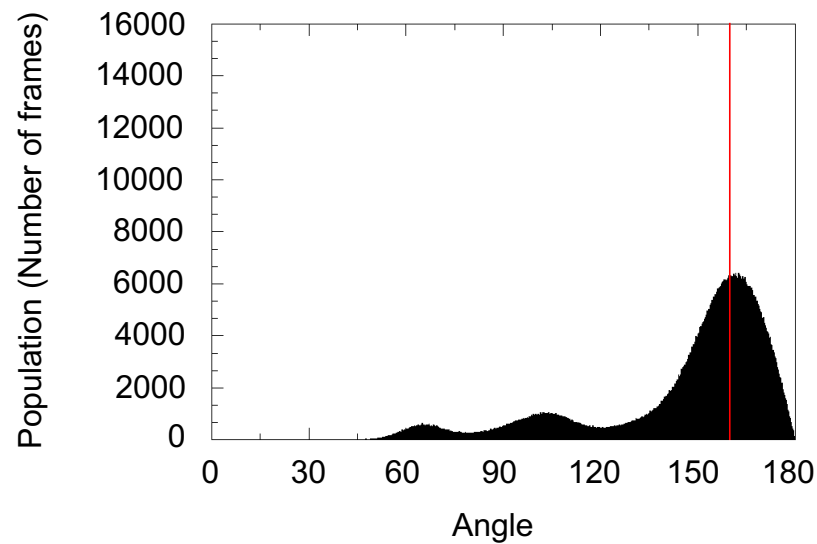

## KCI-TIP4P

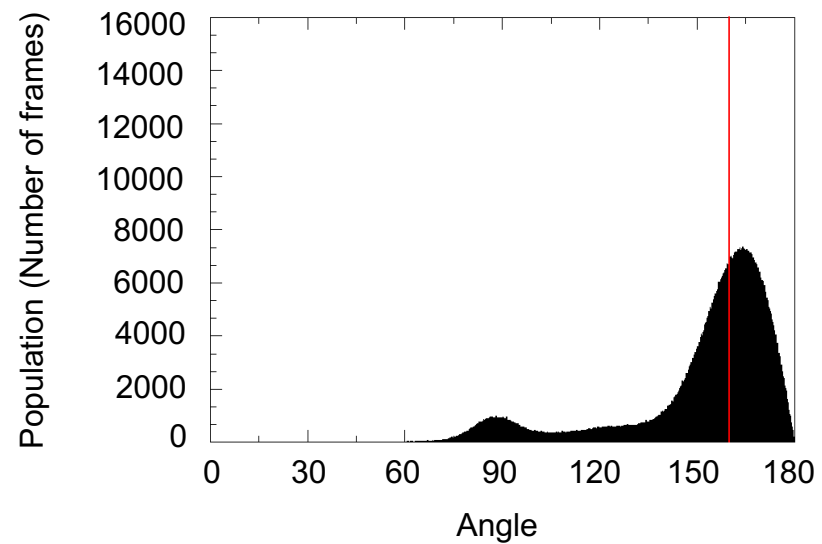

Supplement: Supplementary file 2 — ct2c00291_si_002.zip [file ct2c00291_si_002.zip › Figure S26.pdf]

# Distance G4 tetrad-1 O6-N1

K-TIP3P

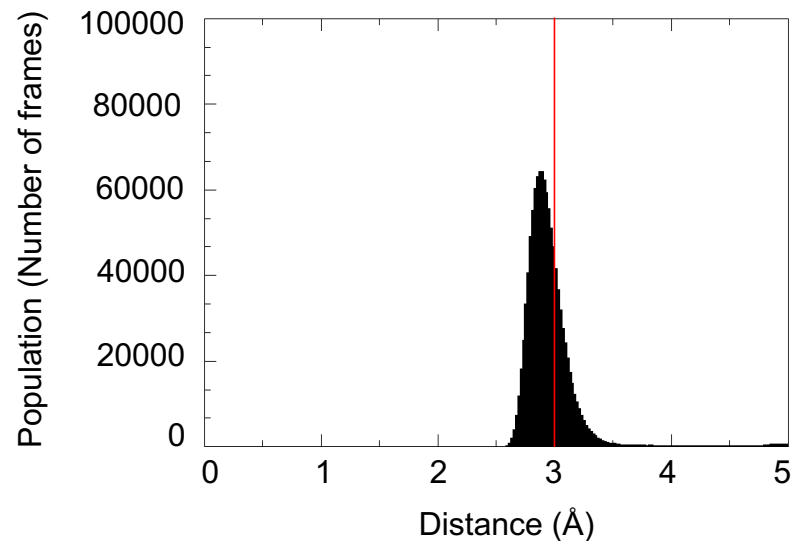

K-TIP4P

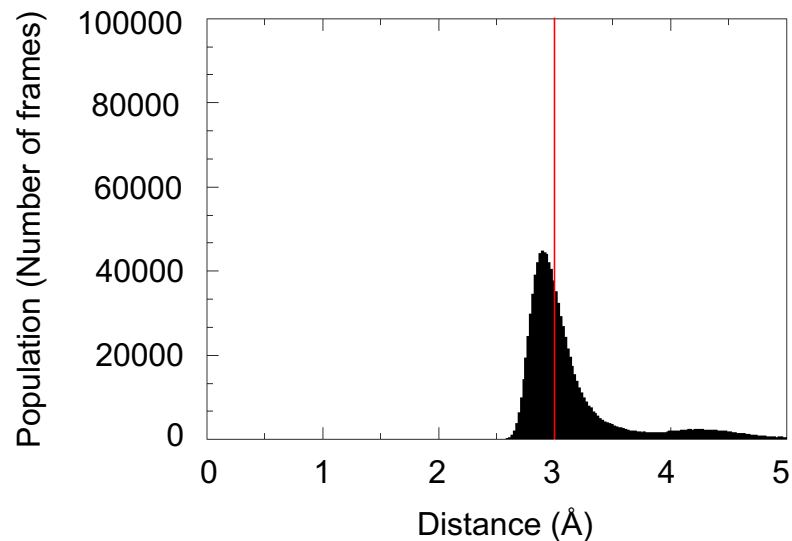

KCI-TIP3P

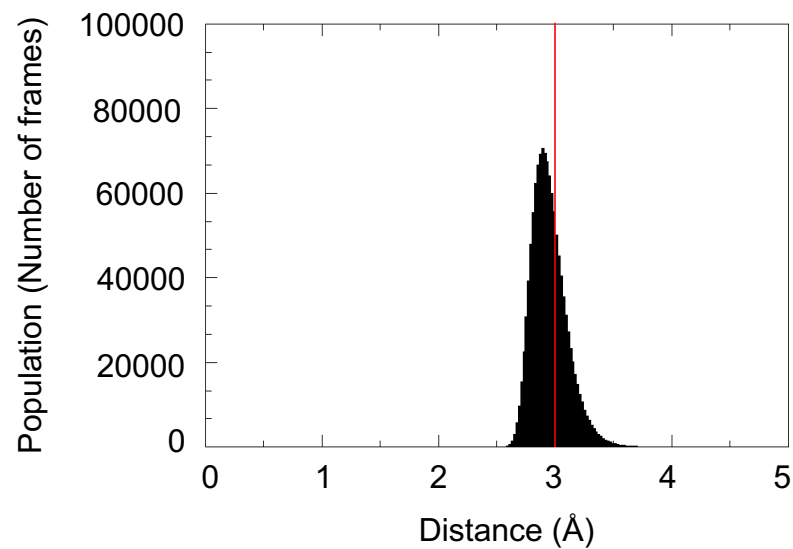

KCI-TIP4P

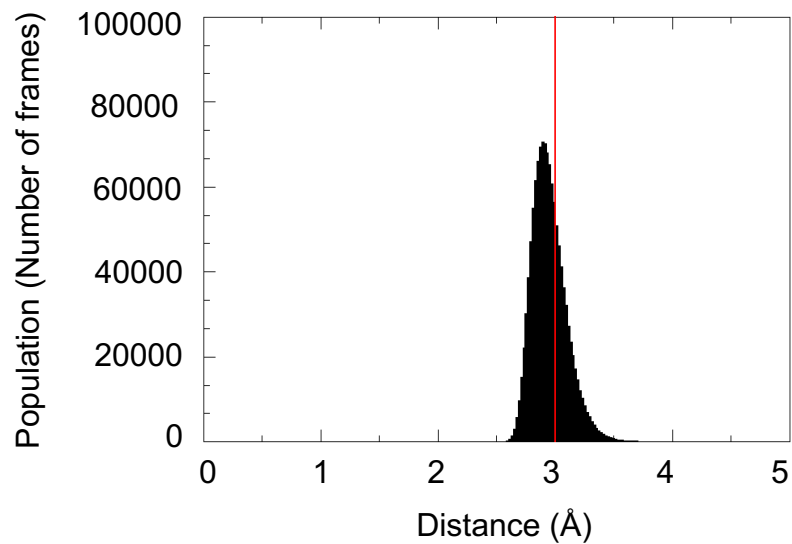

Supplement: Supplementary file 2 — ct2c00291_si_002.zip [file ct2c00291_si_002.zip › Figure S27.pdf]

# Angle G4 tetrad-1 O6-N1

## K-TIP3P

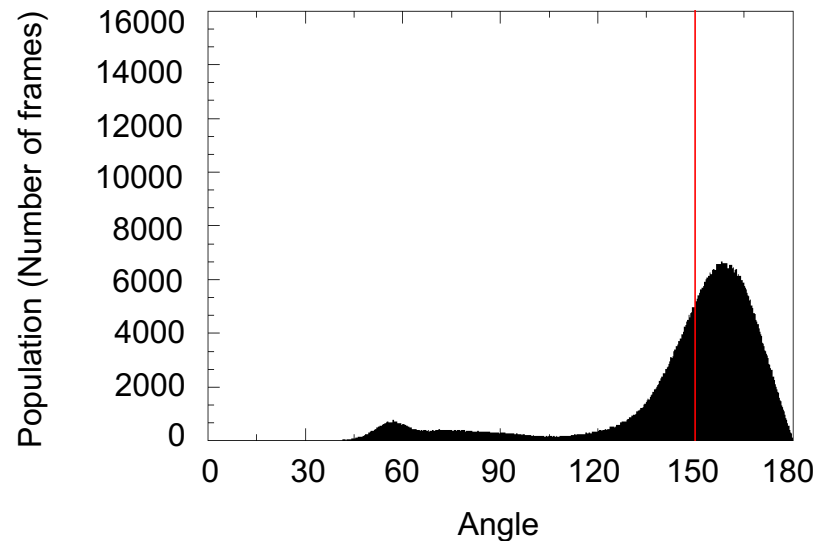

## K-TIP4P

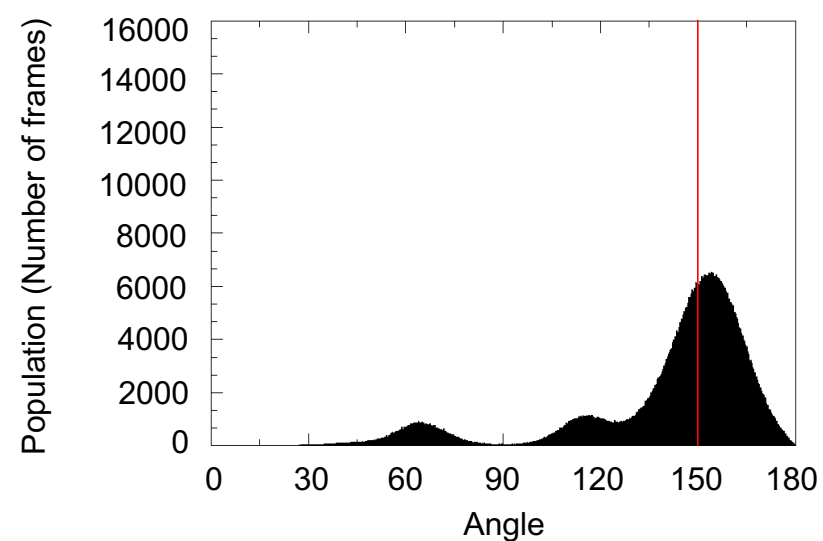

## KCI-TIP3P

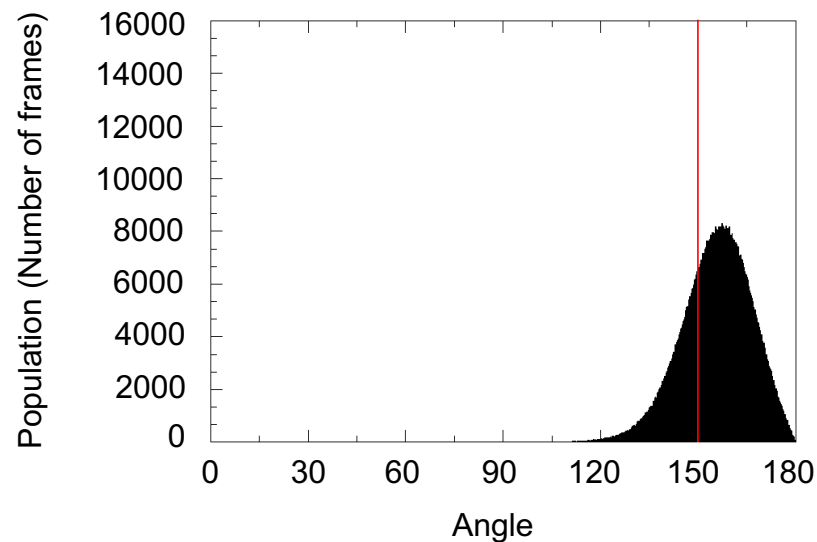

## KCI-TIP4P

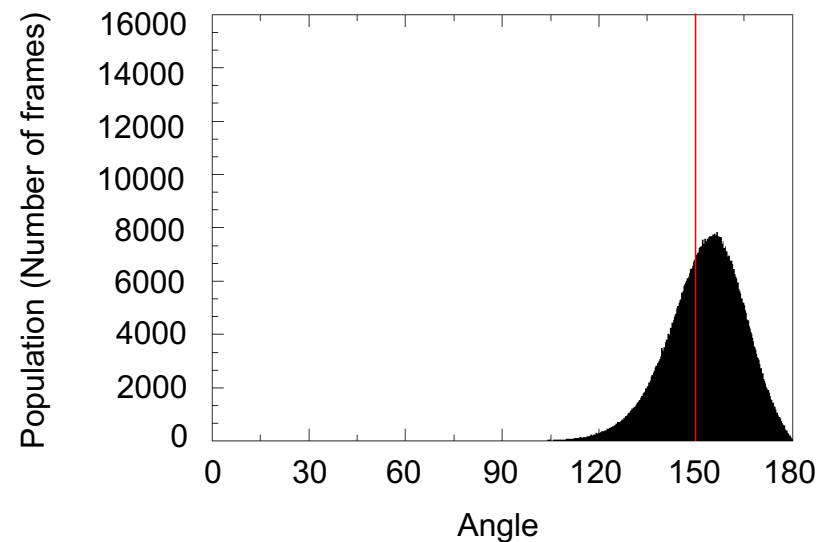

Supplement: Supplementary file 2 — ct2c00291_si_002.zip [file ct2c00291_si_002.zip › Figure S28.pdf]

# Distance G4 tetrad-2 O6-N1

K-TIP3P

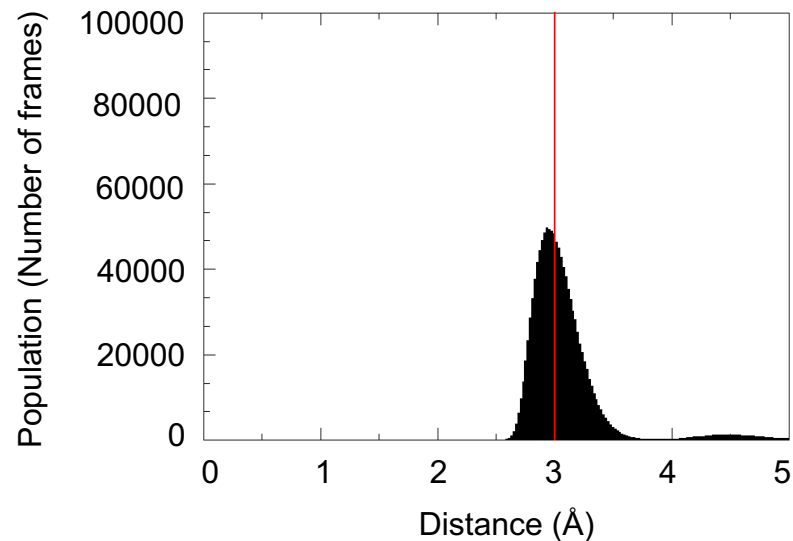

K-TIP4P

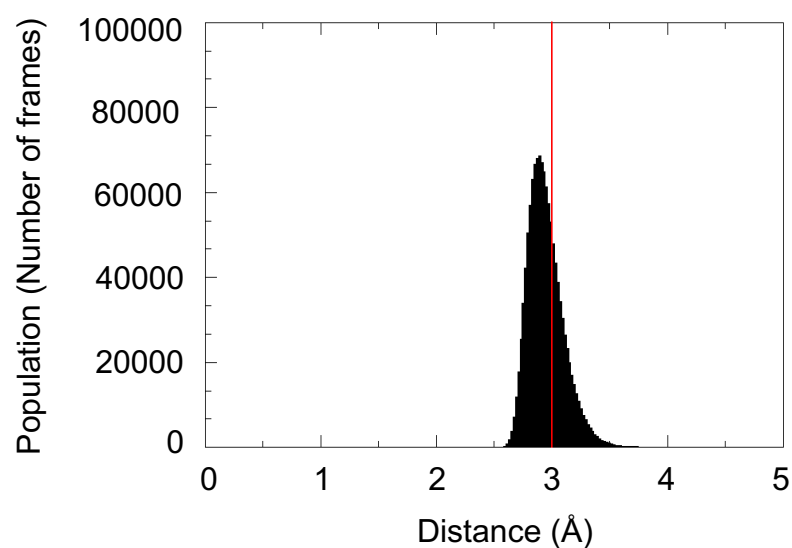

KCI-TIP3P

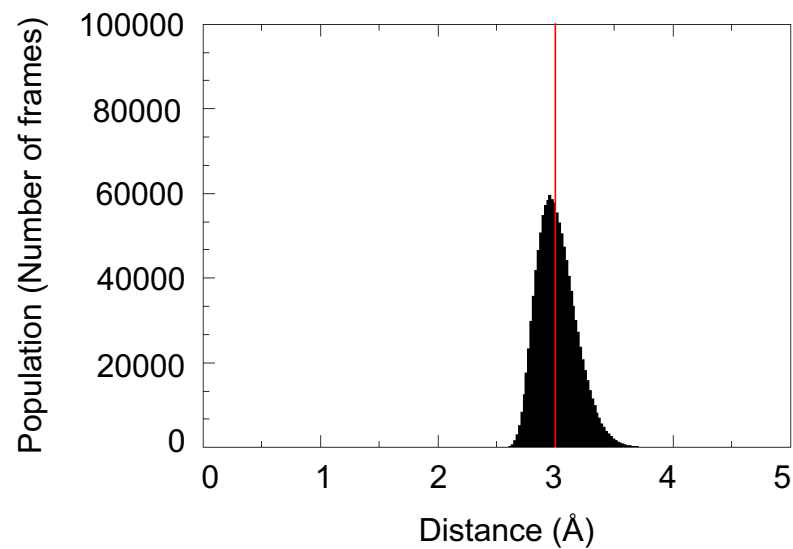

KCI-TIP4P

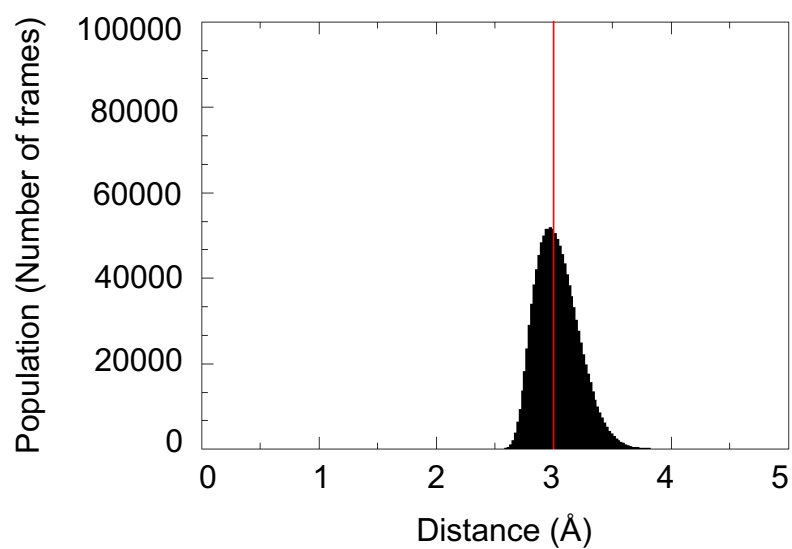

Supplement: Supplementary file 2 — ct2c00291_si_002.zip [file ct2c00291_si_002.zip › Figure S29.pdf]

# Angle G4 tetrad-2 O6-N1

K-TIP3P

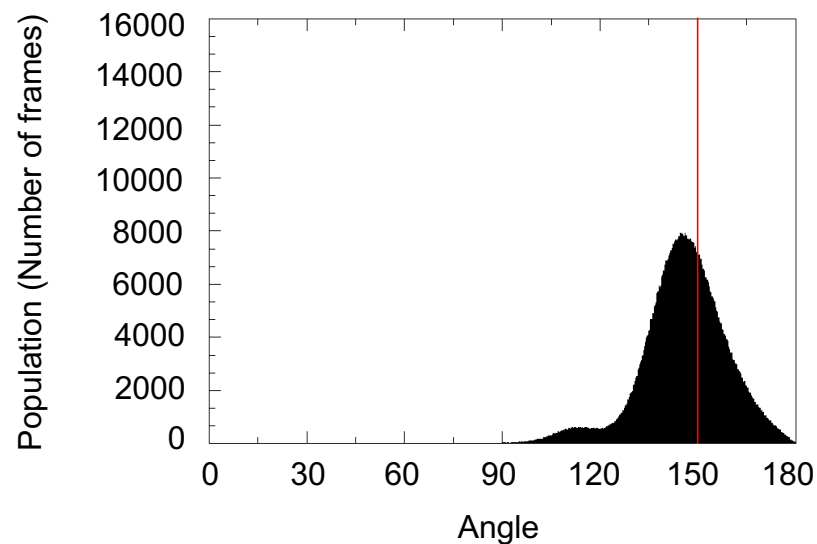

K-TIP4P

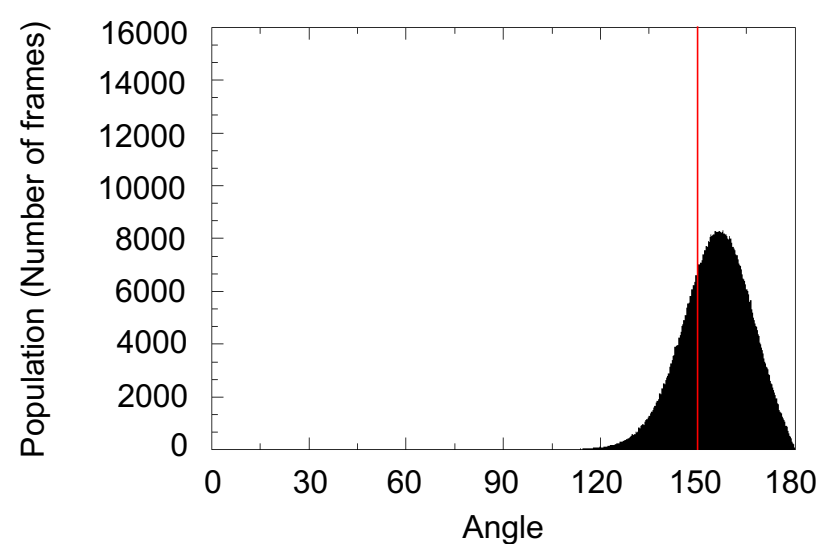

KCI-TIP3P

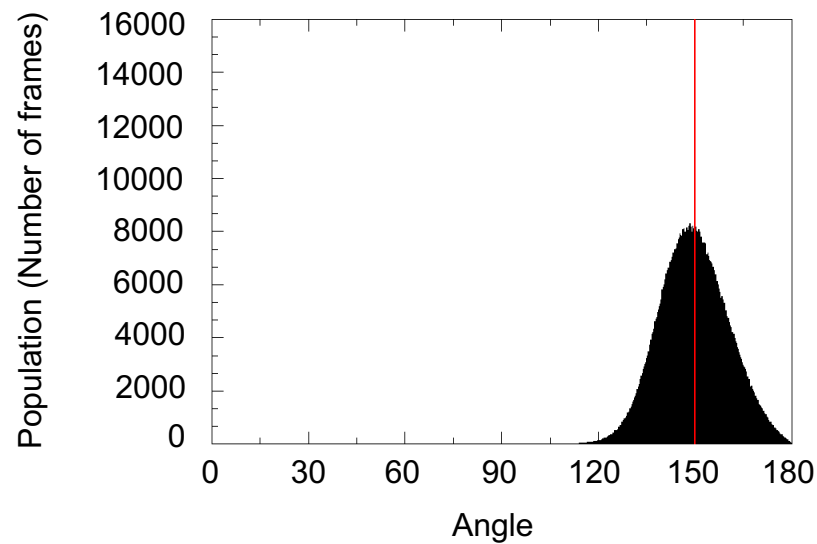

KCI-TIP4P

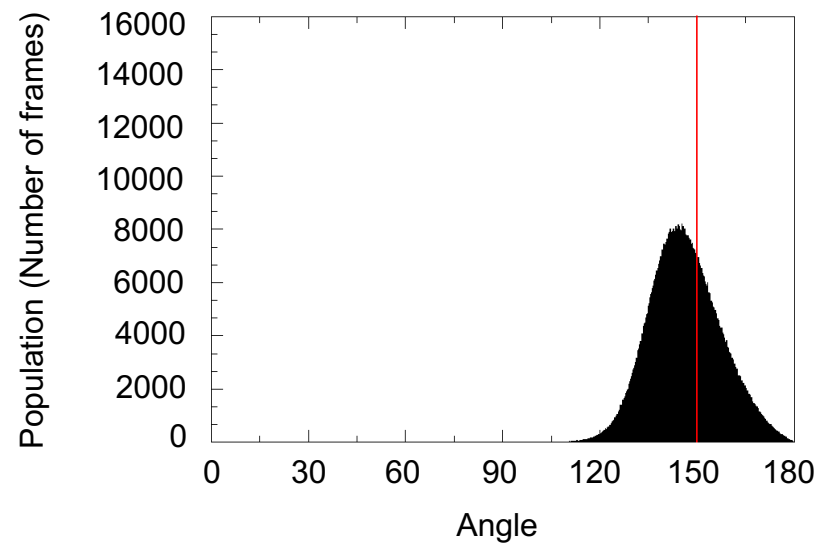

Supplement: Supplementary file 2 — ct2c00291_si_002.zip [file ct2c00291_si_002.zip › Figure S30.pdf]

# Distance G4 tetrad-3 O6-N1

K-TIP3P

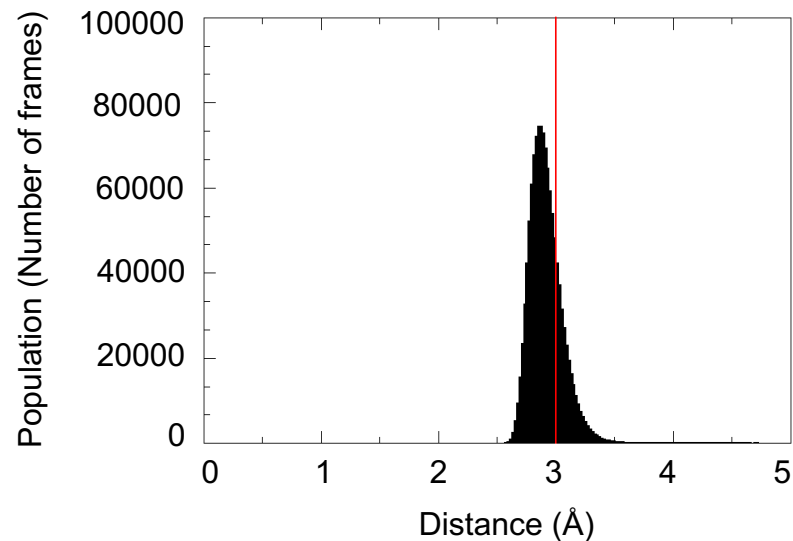

K-TIP4P

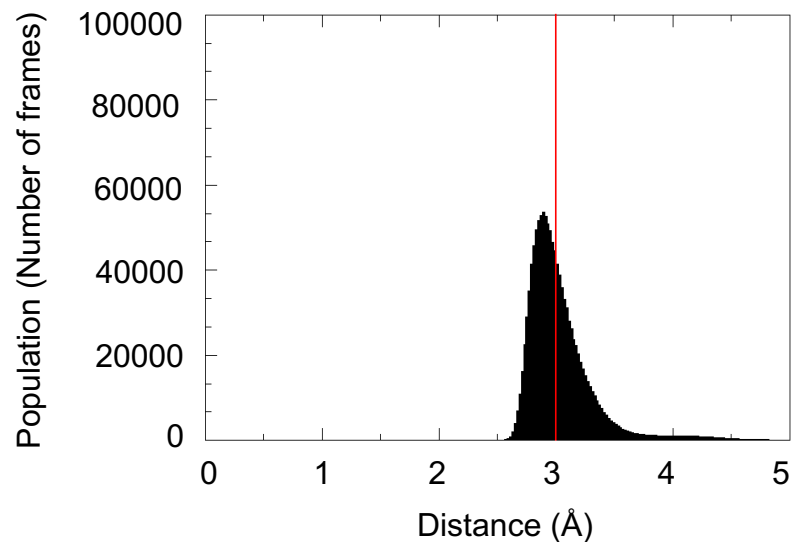

KCI-TIP3P

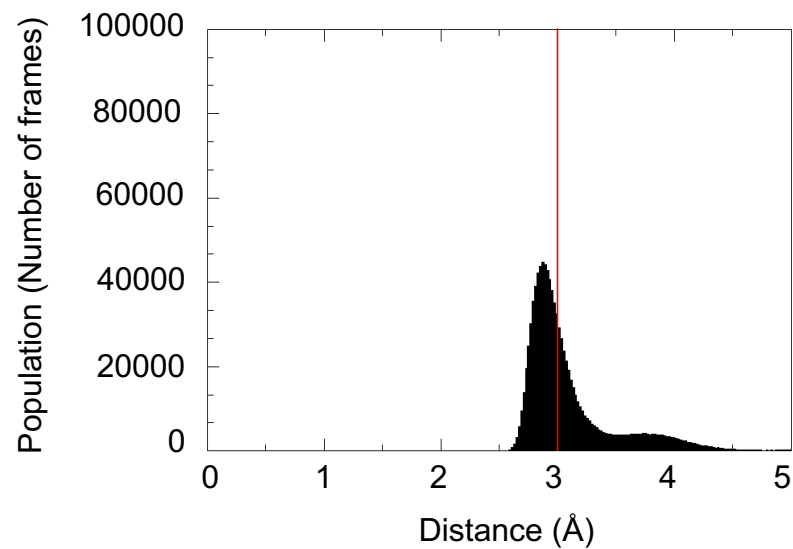

KCI-TIP4P

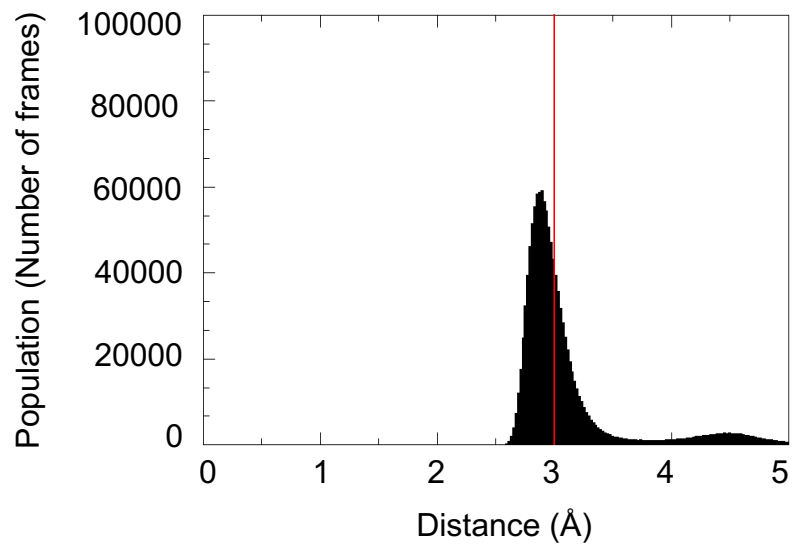

Supplement: Supplementary file 2 — ct2c00291_si_002.zip [file ct2c00291_si_002.zip › Figure S31.pdf]

# Angle G4 tetrad-3 O6-N1

## K-TIP3P

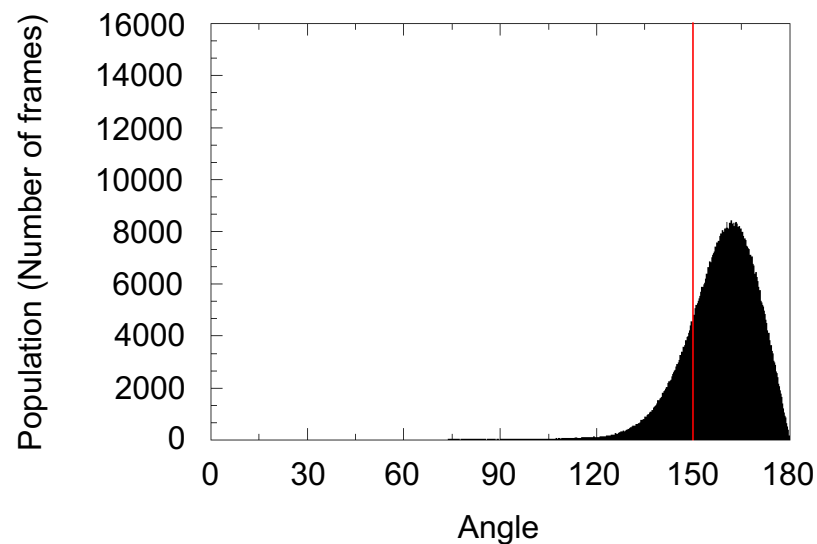

## K-TIP4P

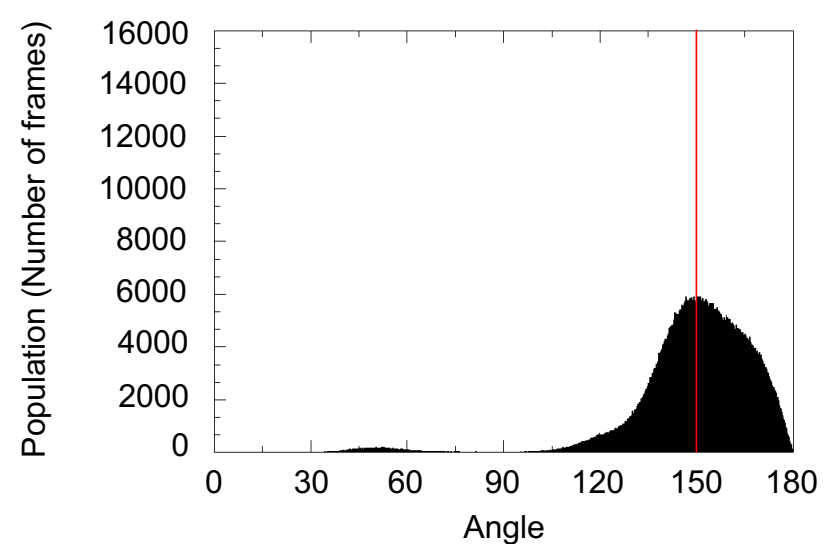

## KCI-TIP3P

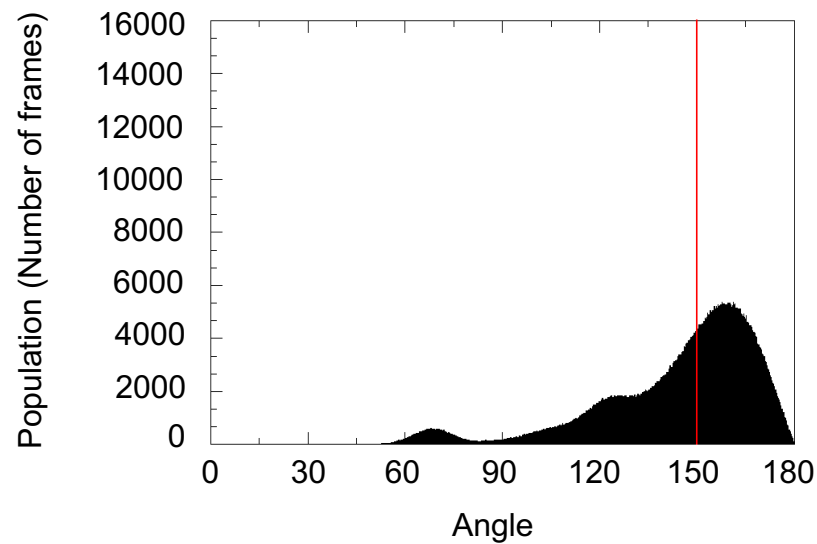

## KCI-TIP4P

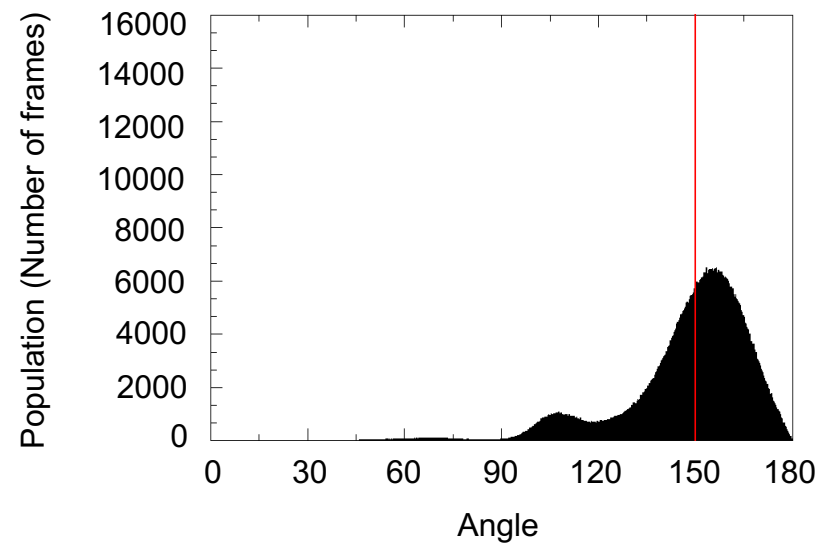

Supplement: Supplementary file 2 — ct2c00291_si_002.zip [file ct2c00291_si_002.zip › Figure S32.pdf]

# Distance G4 tetrad-1 N1-N7

K-TIP3P

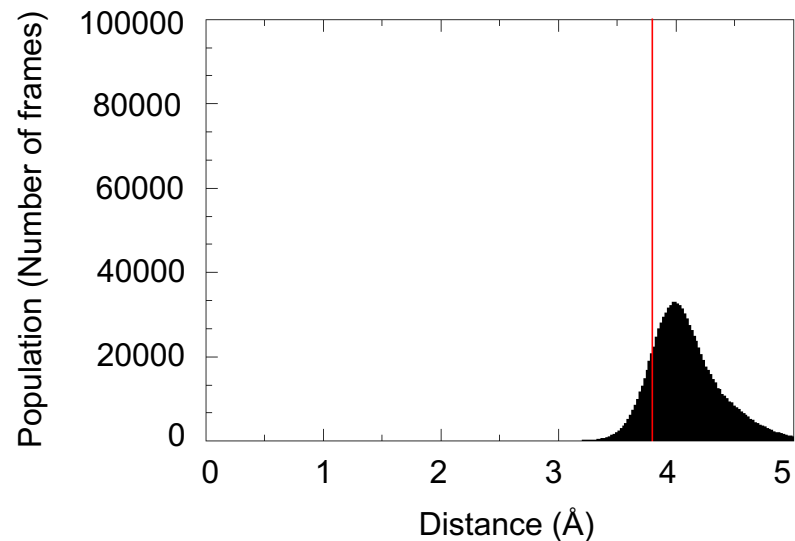

K-TIP4P

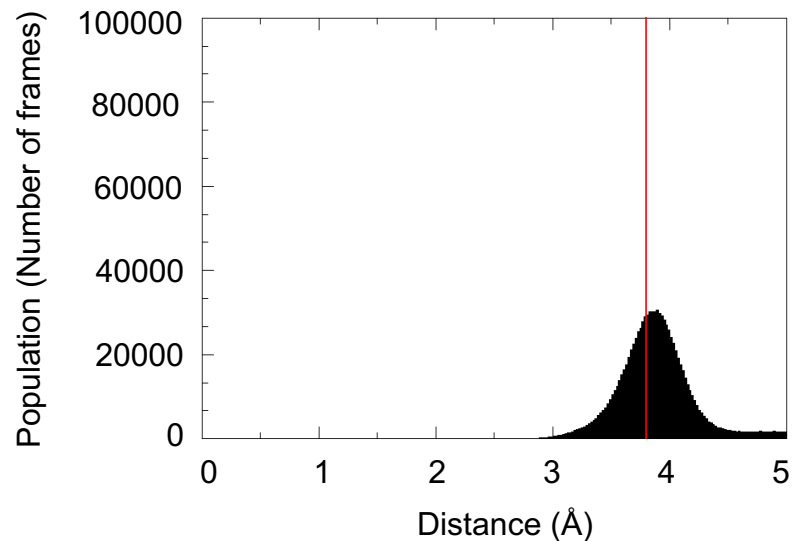

KCI-TIP3P

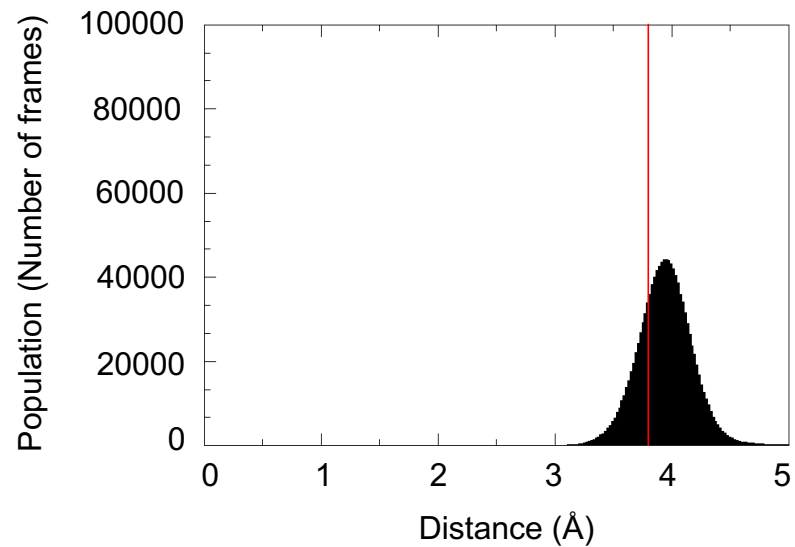

KCI-TIP4P

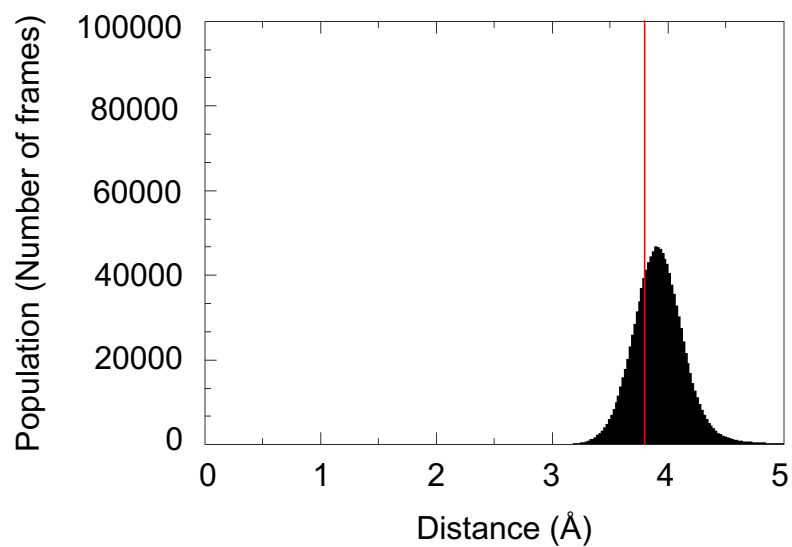

Supplement: Supplementary file 2 — ct2c00291_si_002.zip [file ct2c00291_si_002.zip › Figure S33.pdf]

# Angle G4 tetrad-1 N1-N7

## K-TIP3P

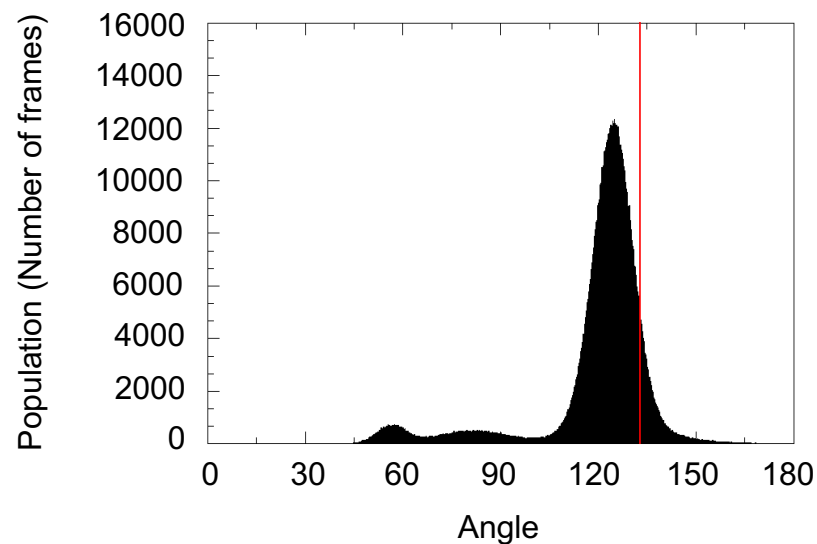

## K-TIP4P

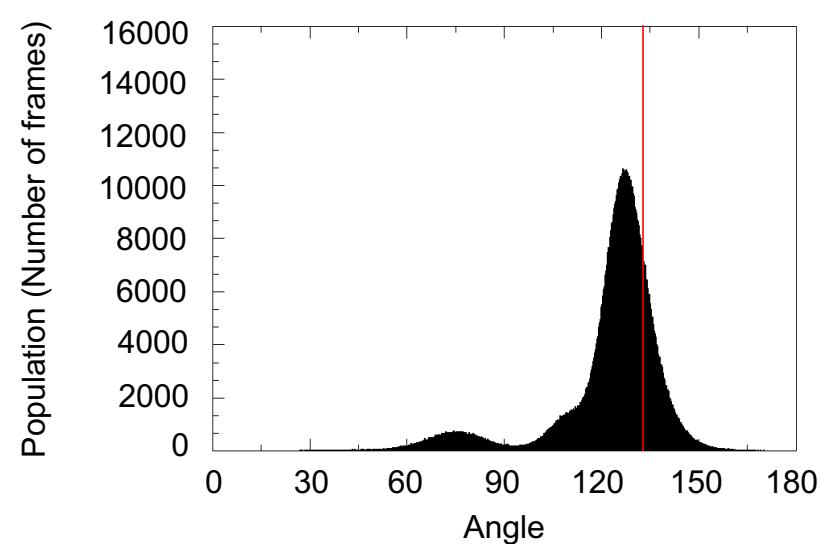

## KCI-TIP3P

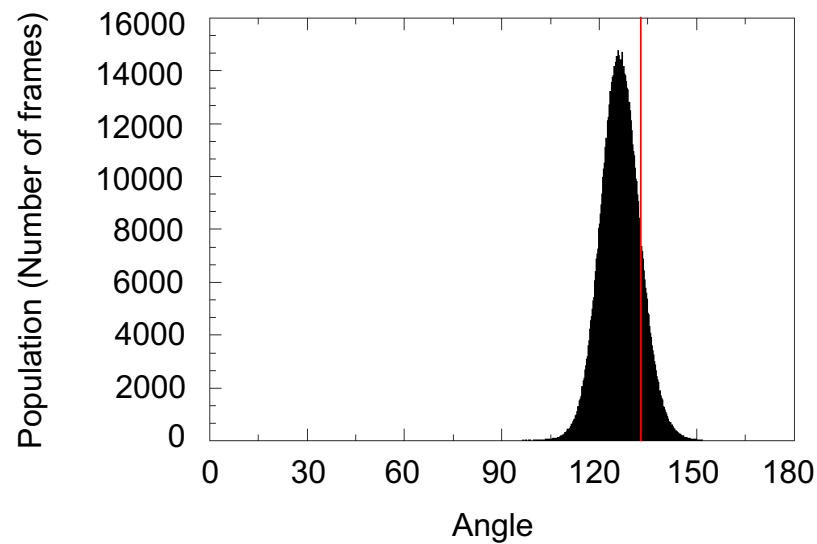

## KCI-TIP4P

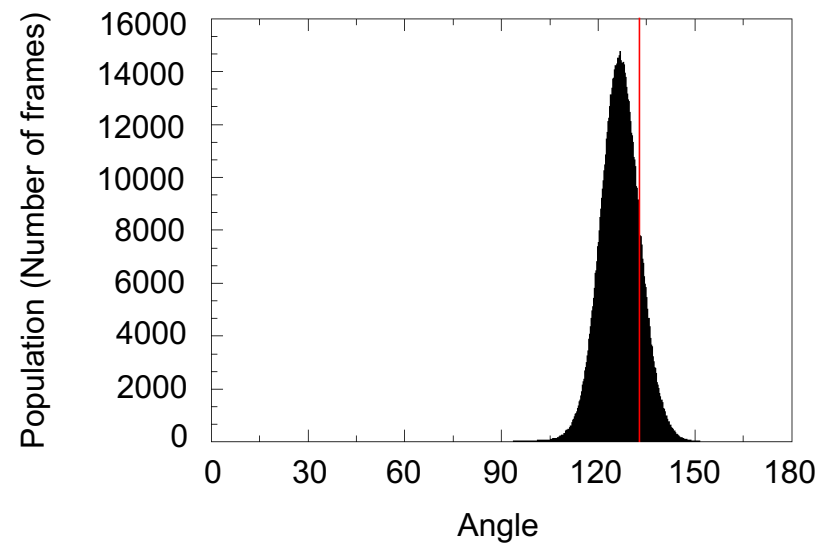

Supplement: Supplementary file 2 — ct2c00291_si_002.zip [file ct2c00291_si_002.zip › Figure S34.pdf]

# Distance G4 tetrad-2 N1-N7

K-TIP3P

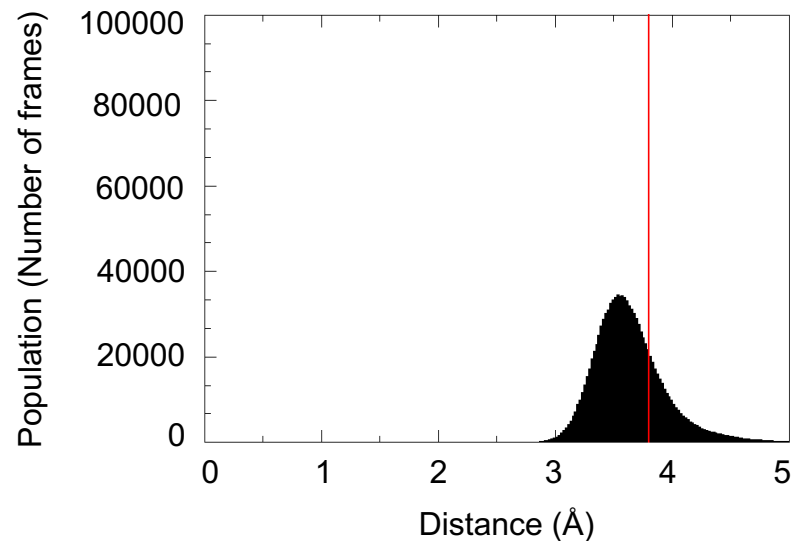

K-TIP4P

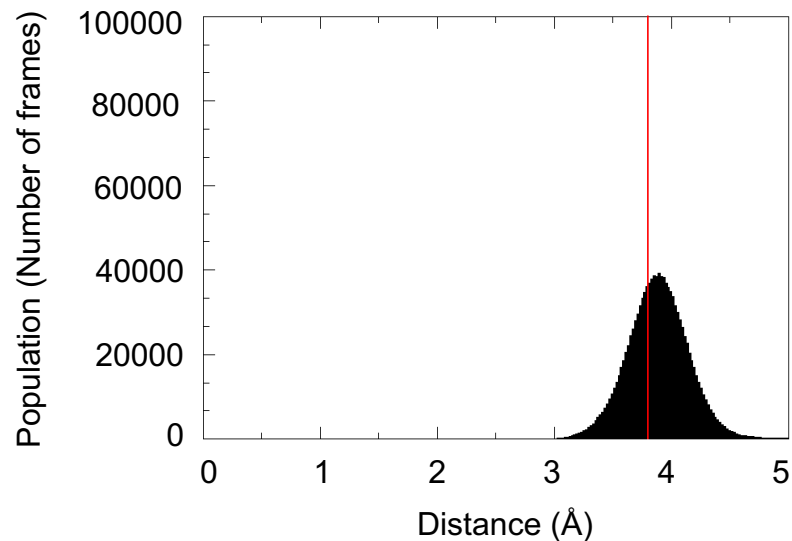

KCI-TIP3P

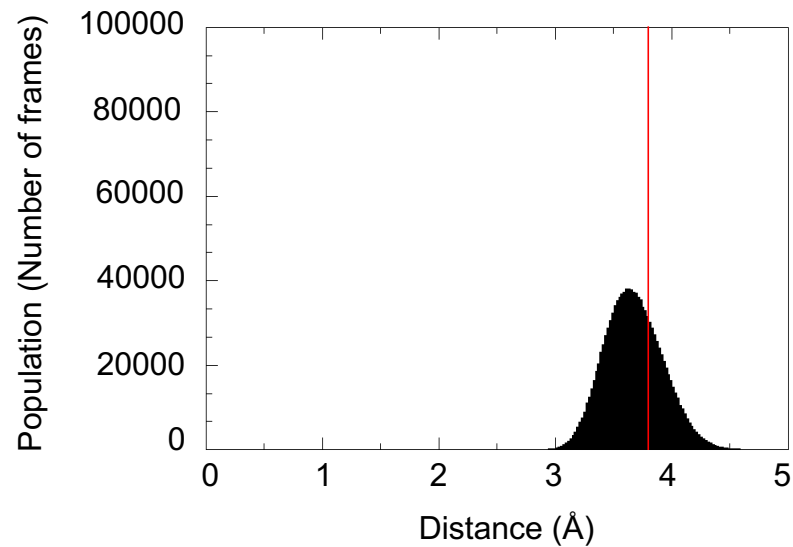

KCI-TIP4P

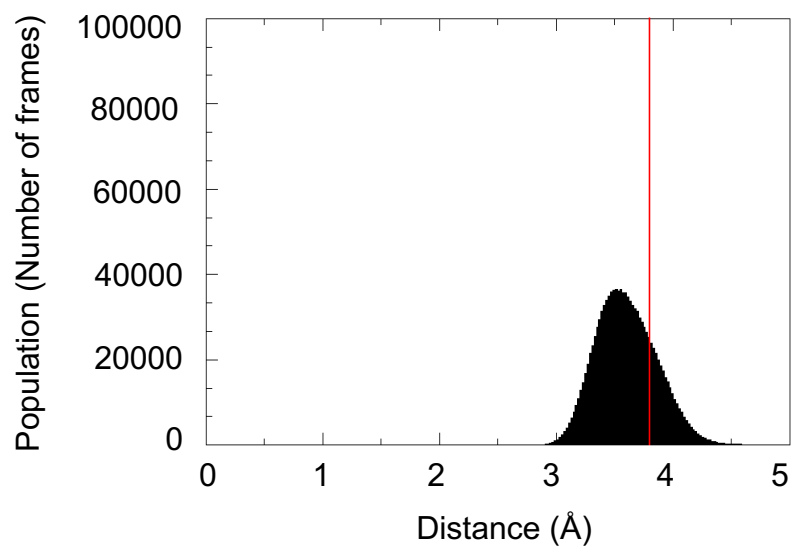

Supplement: Supplementary file 2 — ct2c00291_si_002.zip [file ct2c00291_si_002.zip › Figure S35.pdf]

# Angle G4 tetrad-2 N1-N7

K-TIP3P

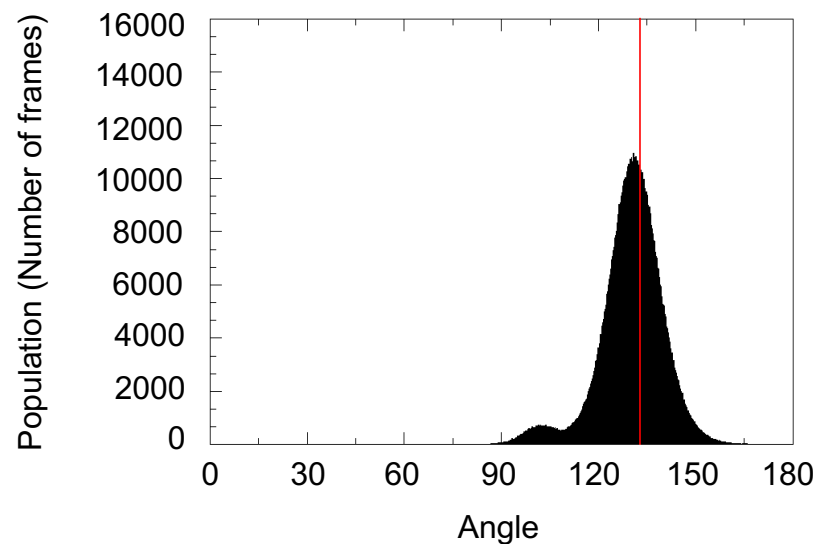

K-TIP4P

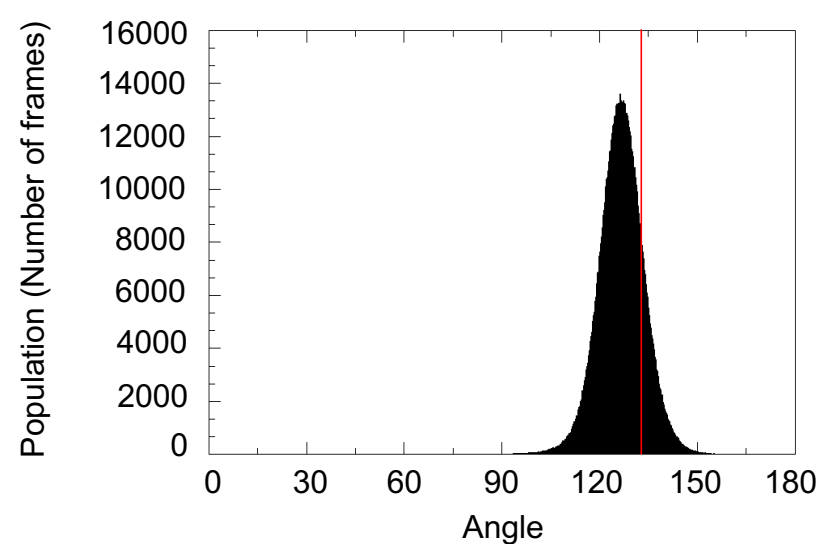

KCI-TIP3P

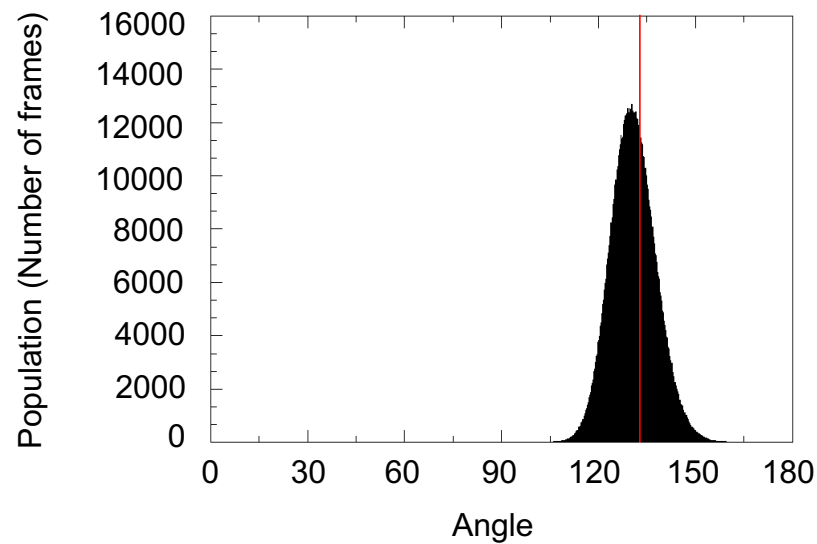

KCI-TIP4P

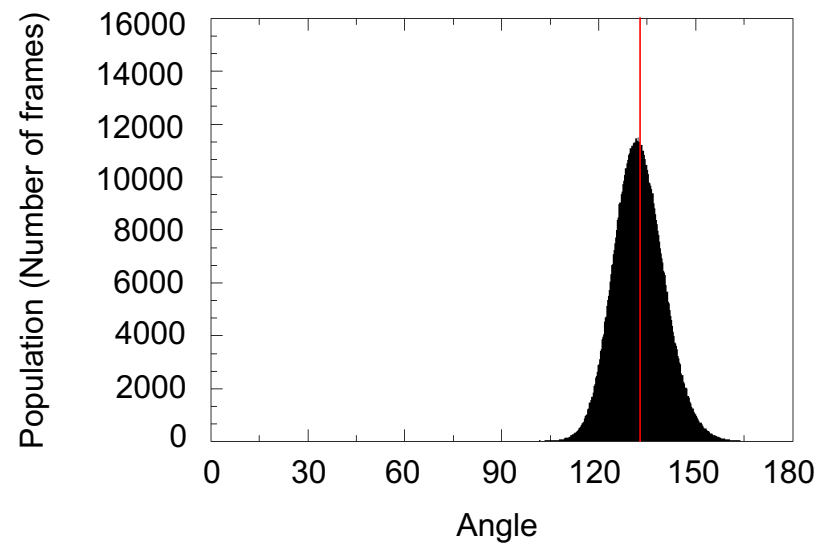

Supplement: Supplementary file 2 — ct2c00291_si_002.zip [file ct2c00291_si_002.zip › Figure S36.pdf]

# Distance G4 tetrad-3 N1-N7

K-TIP3P

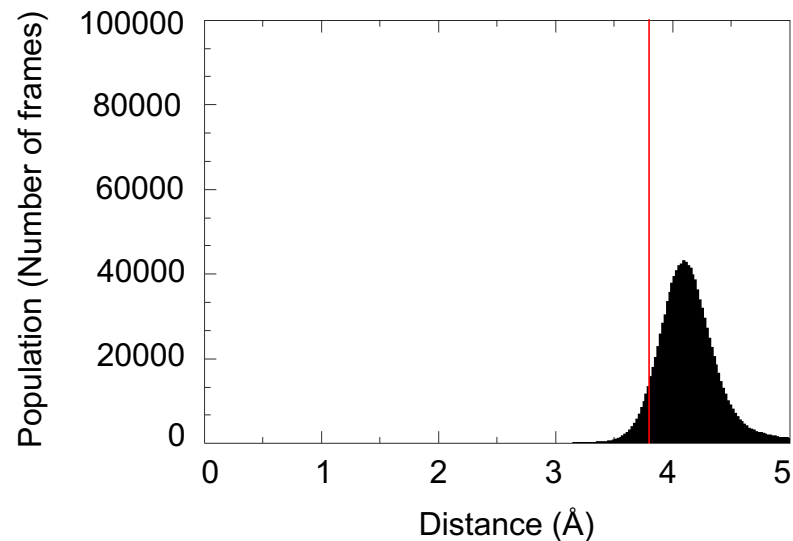

K-TIP4P

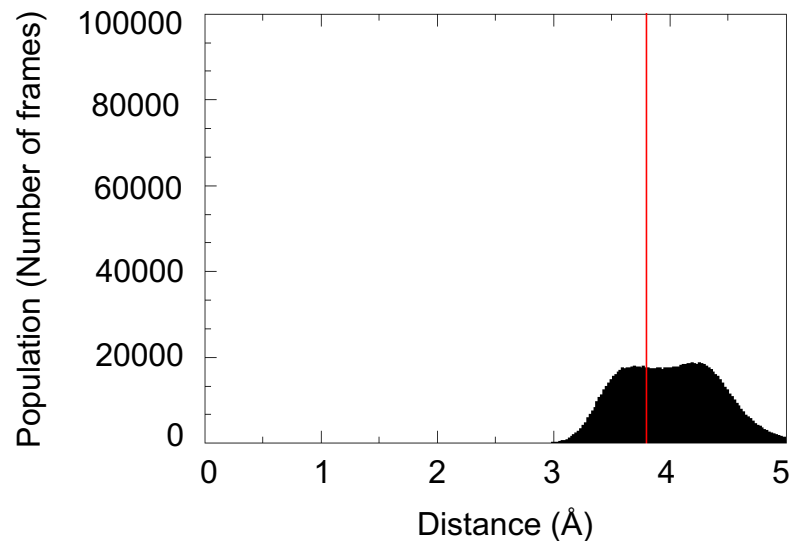

KCI-TIP3P

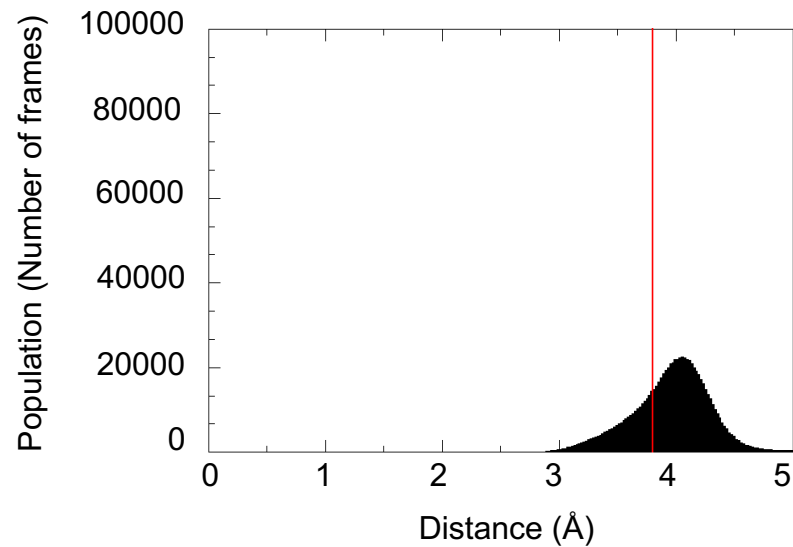

KCI-TIP4P

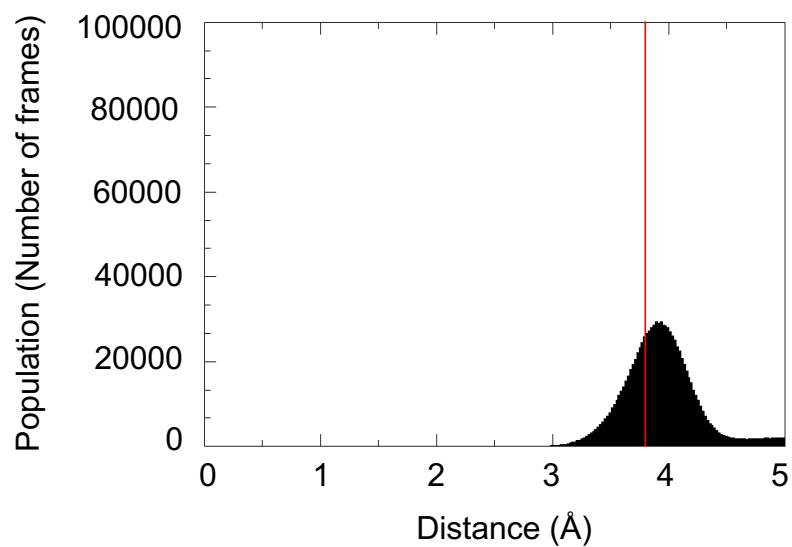

Supplement: Supplementary file 2 — ct2c00291_si_002.zip [file ct2c00291_si_002.zip › Figure S37.pdf]

# Angle G4 tetrad-3 N1-N7

K-TIP3P

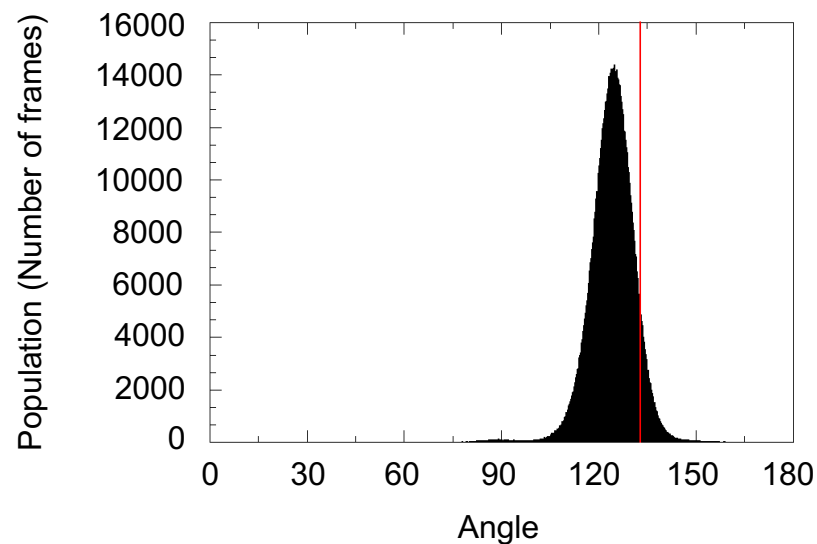

K-TIP4P

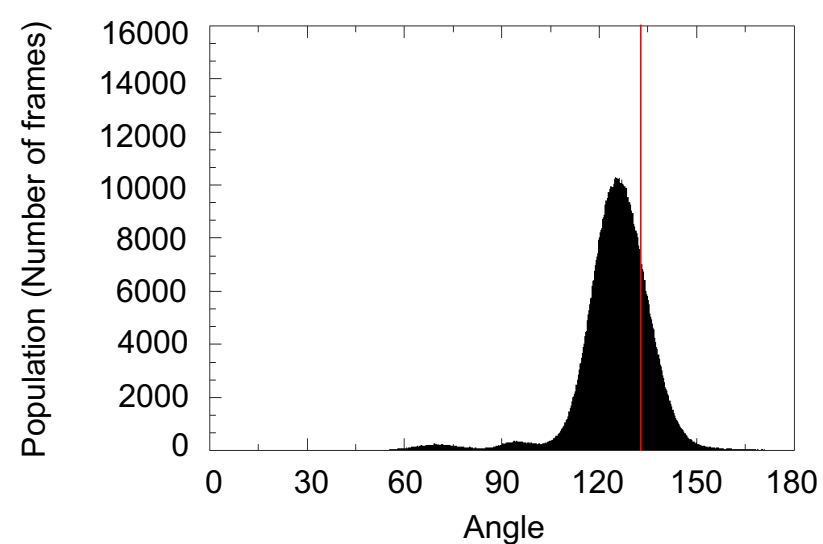

KCI-TIP3P

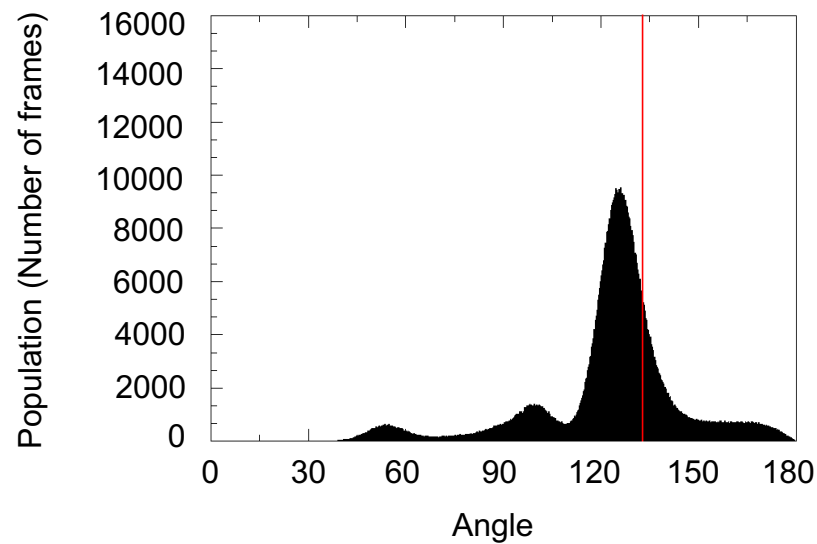

KCI-TIP4P

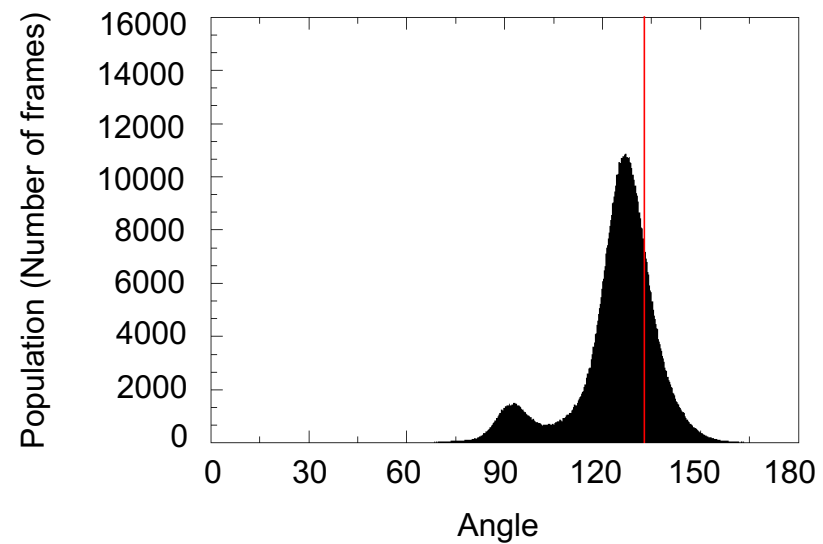

Supplement: Supplementary file 2 — ct2c00291_si_002.zip [file ct2c00291_si_002.zip › Figure S38.pdf]

# Distance duplex N1-N3

K-TIP3P

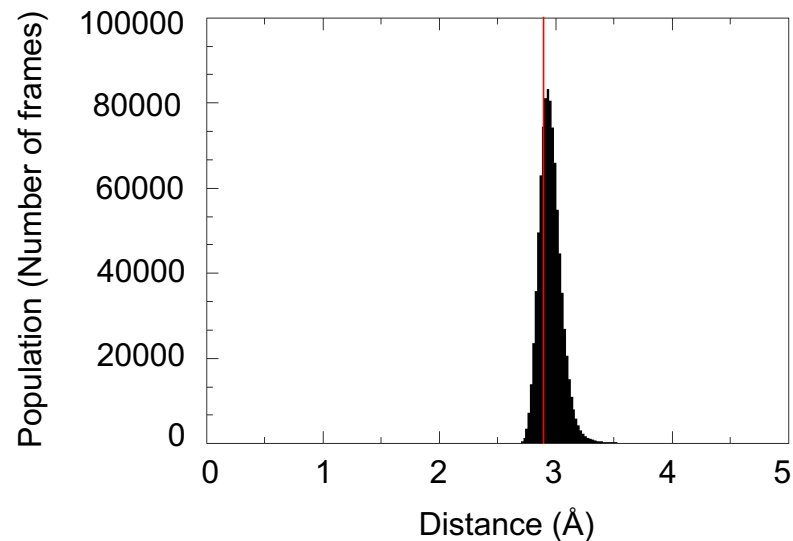

K-TIP4P

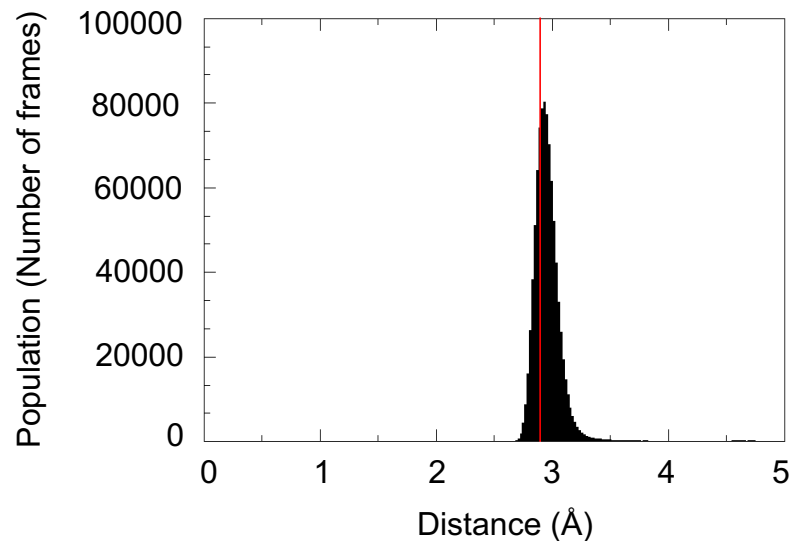

KCI-TIP3P

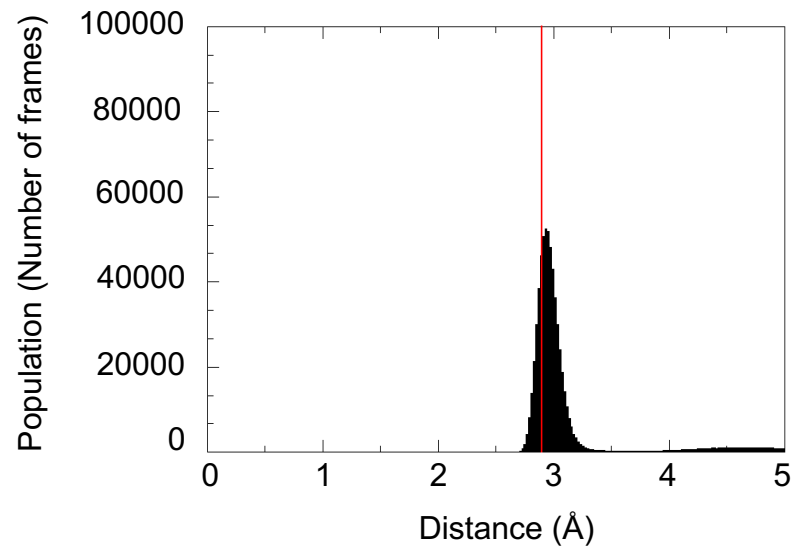

KCI-TIP4P

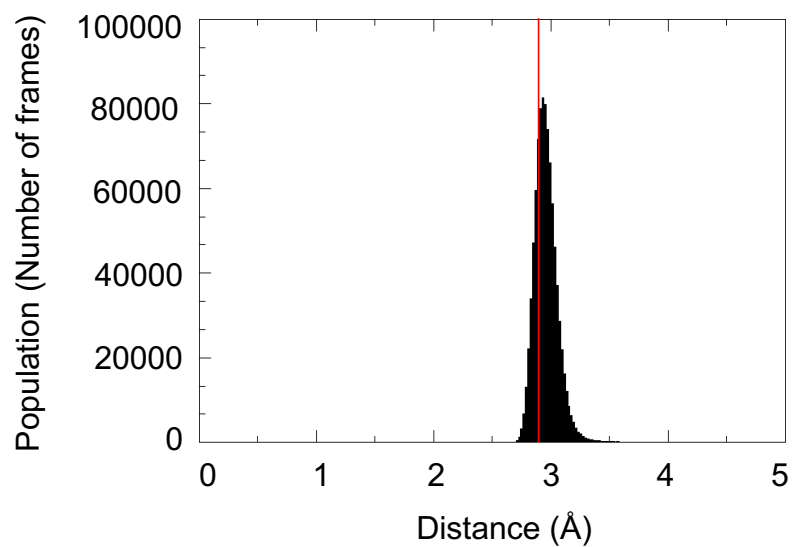

Supplement: Supplementary file 2 — ct2c00291_si_002.zip [file ct2c00291_si_002.zip › Figure S39.pdf]

# Angle duplex N1-N3

## K-TIP3P

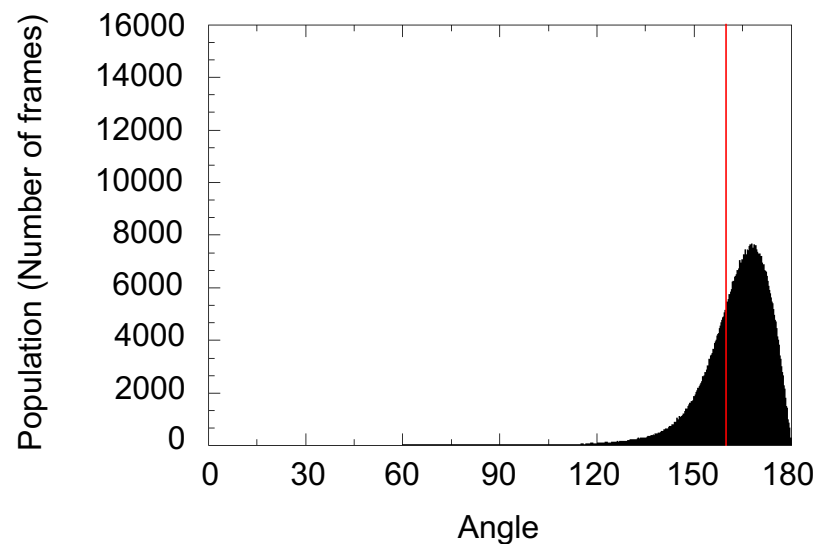

## K-TIP4P

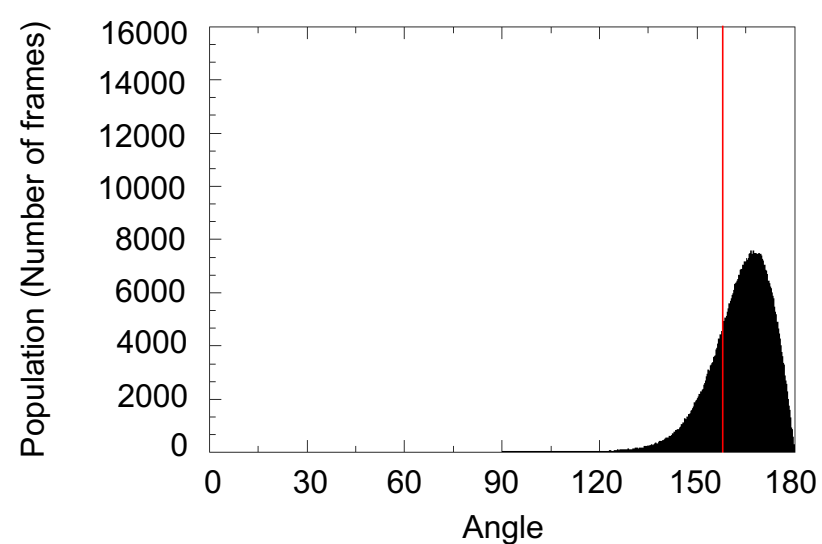

## KCI-TIP3P

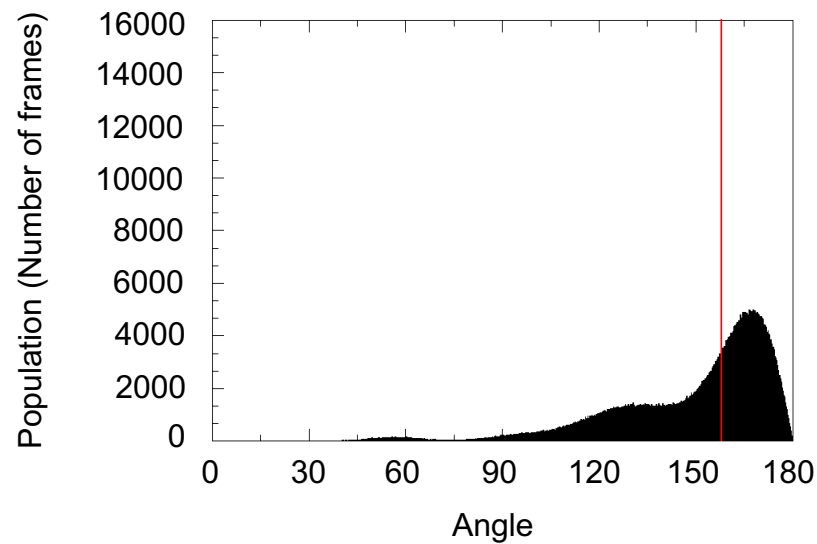

## KCI-TIP4P

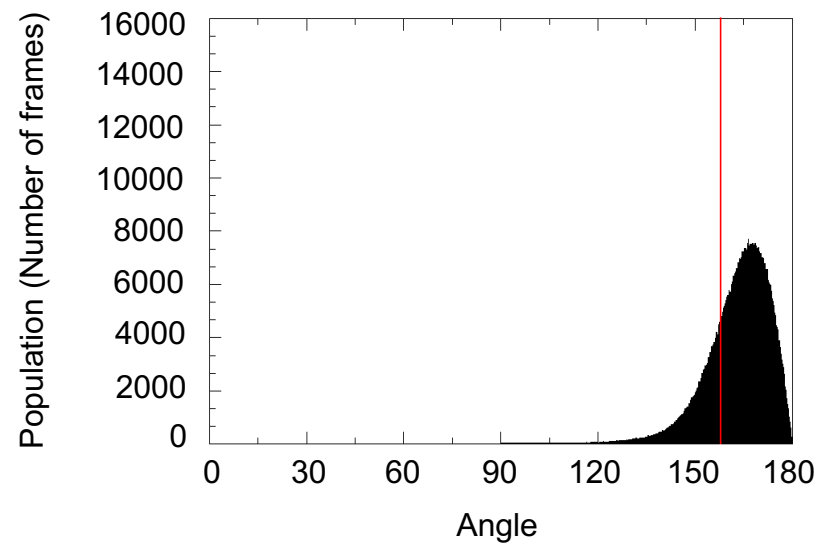

Supplement: Supplementary file 2 — ct2c00291_si_002.zip [file ct2c00291_si_002.zip › Figure S40.pdf]

# Distance duplex N2-O2

K-TIP3P

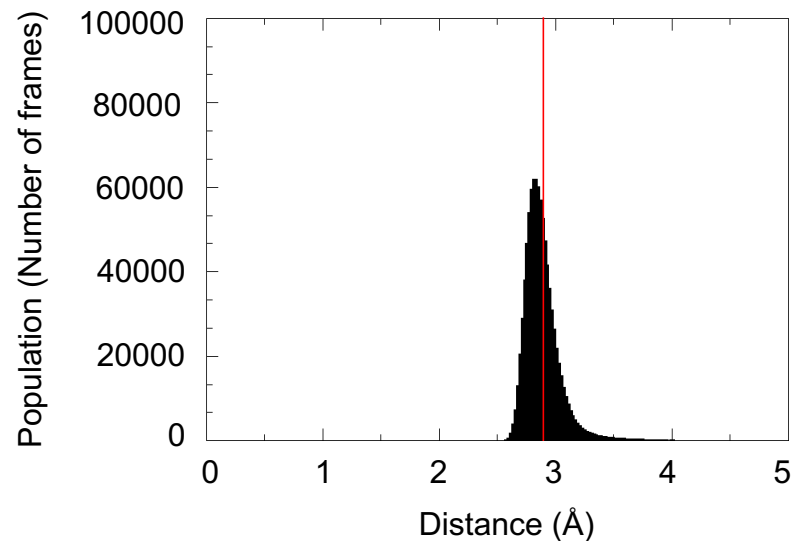

K-TIP4P

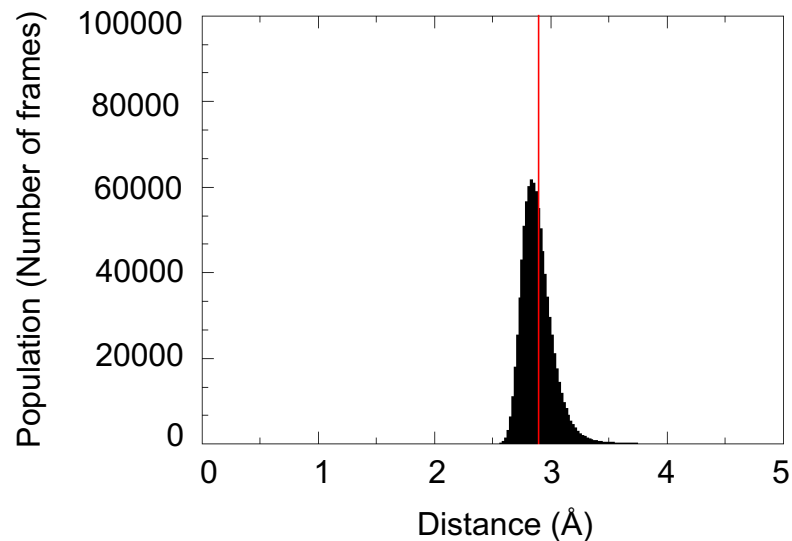

KCI-TIP3P

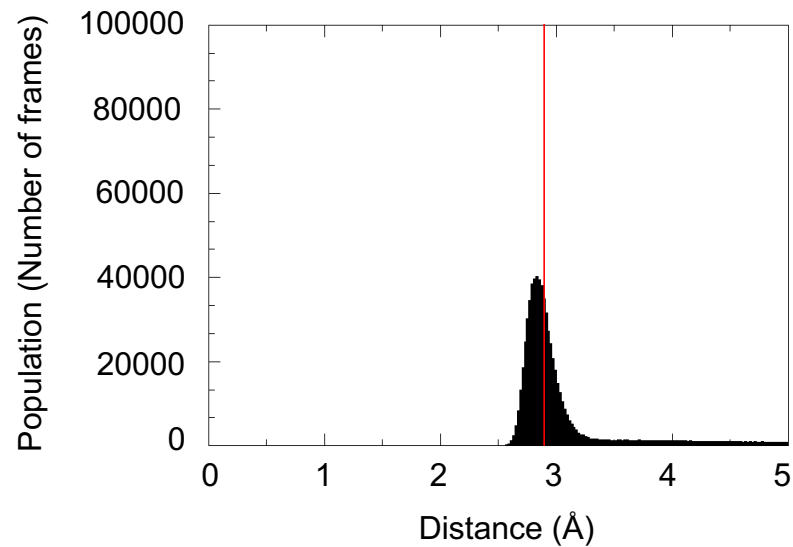

KCI-TIP4P

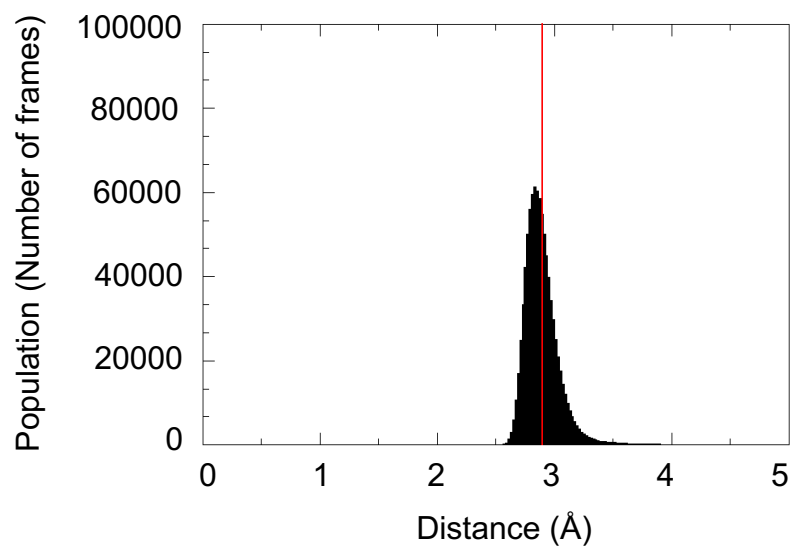

Supplement: Supplementary file 2 — ct2c00291_si_002.zip [file ct2c00291_si_002.zip › Figure S41.pdf]

# Angle duplex N2-O2

## K-TIP3P

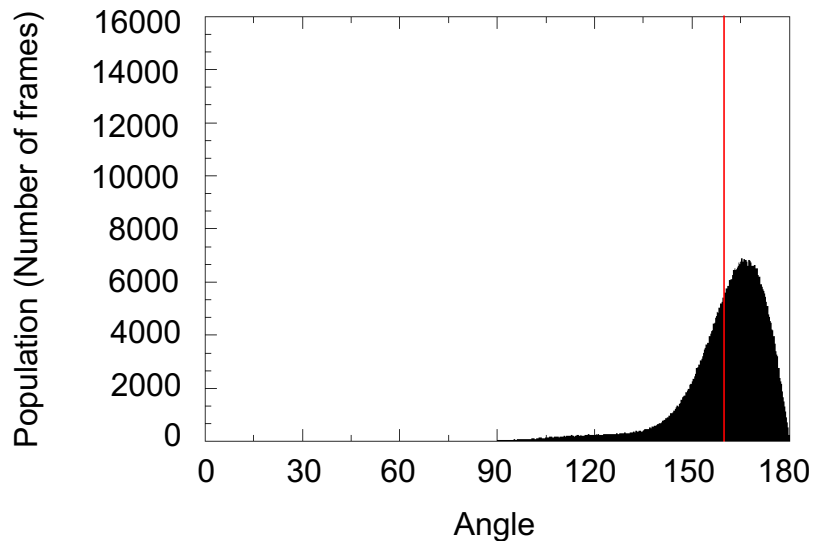

## K-TIP4P

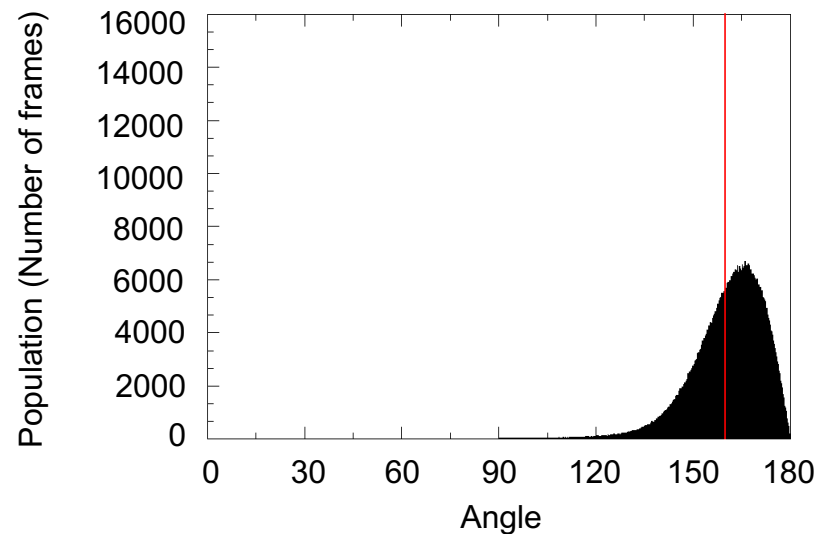

## KCI-TIP3P

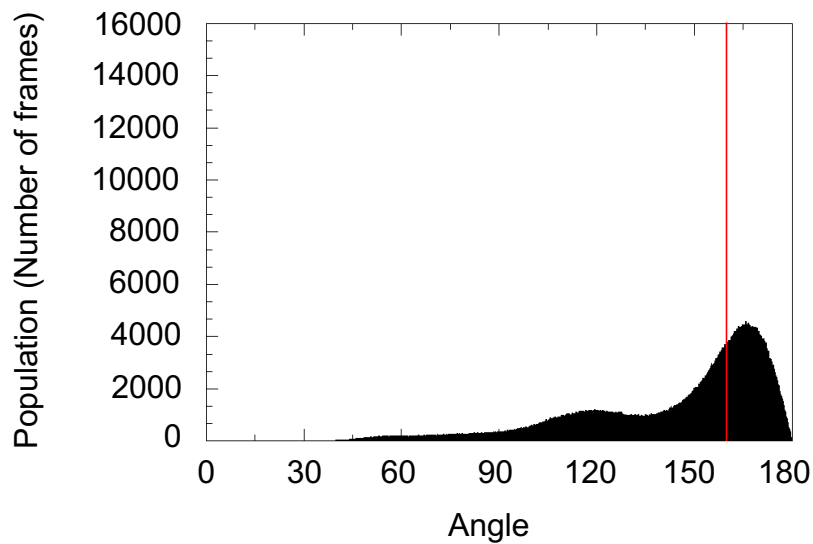

## KCI-TIP4P

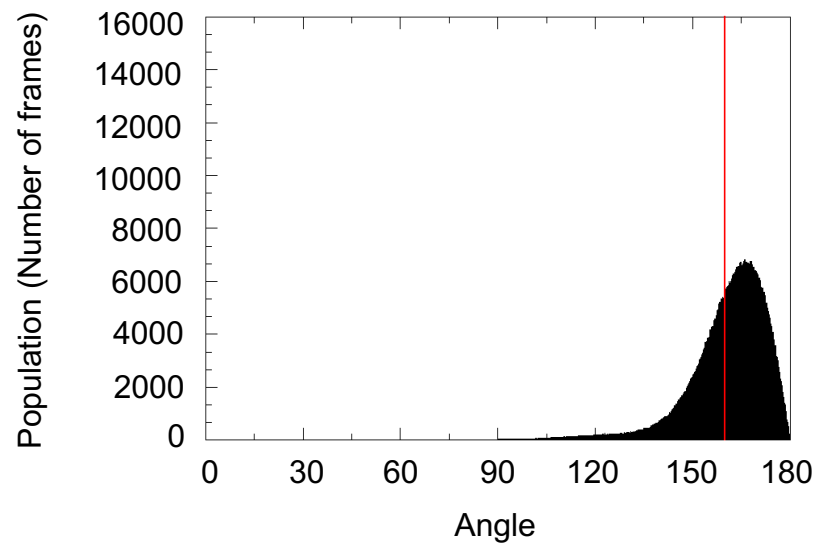

Supplement: Supplementary file 2 — ct2c00291_si_002.zip [file ct2c00291_si_002.zip › Figure S42.pdf]

# Distance duplex O6-N4

## K-TIP3P

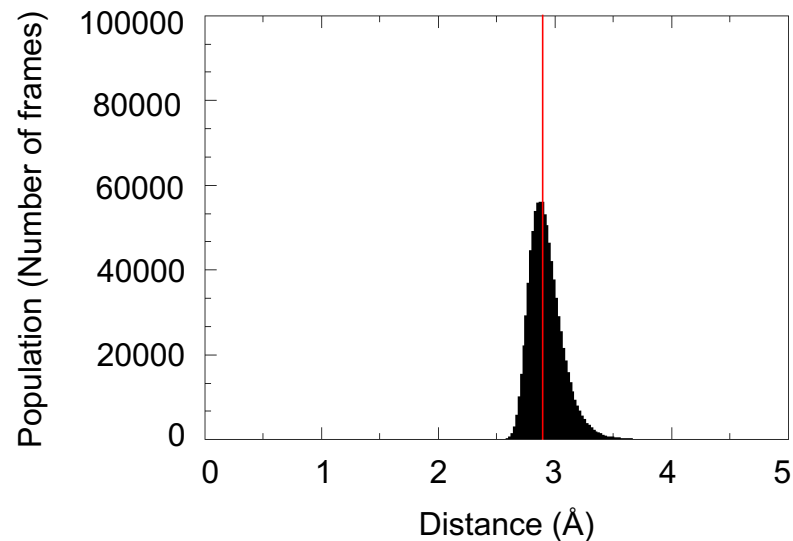

## K-TIP4P

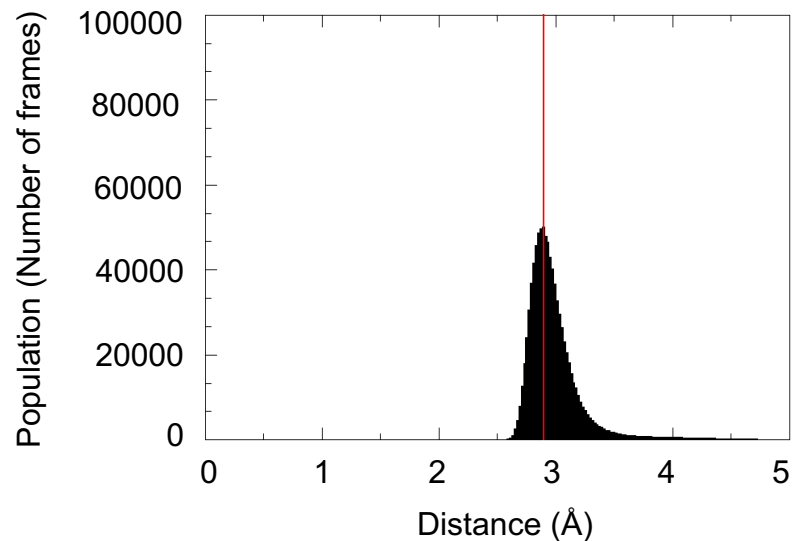

## KCI-TIP3P

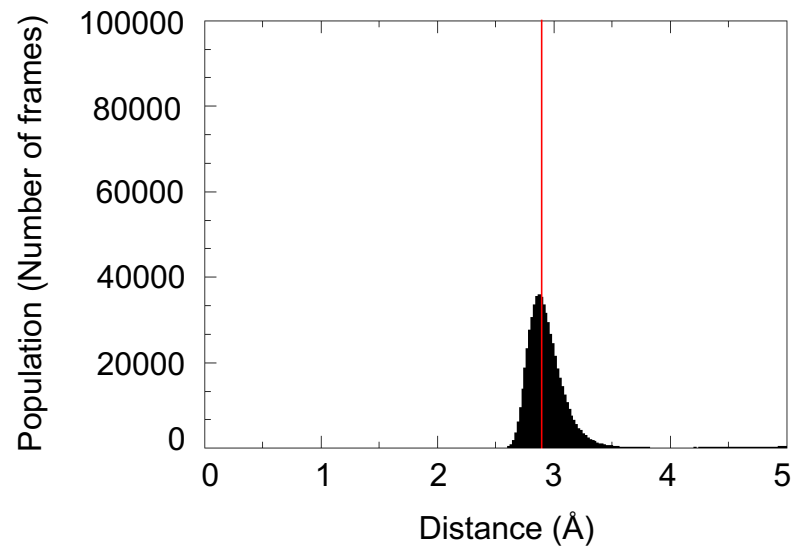

## KCI-TIP4P

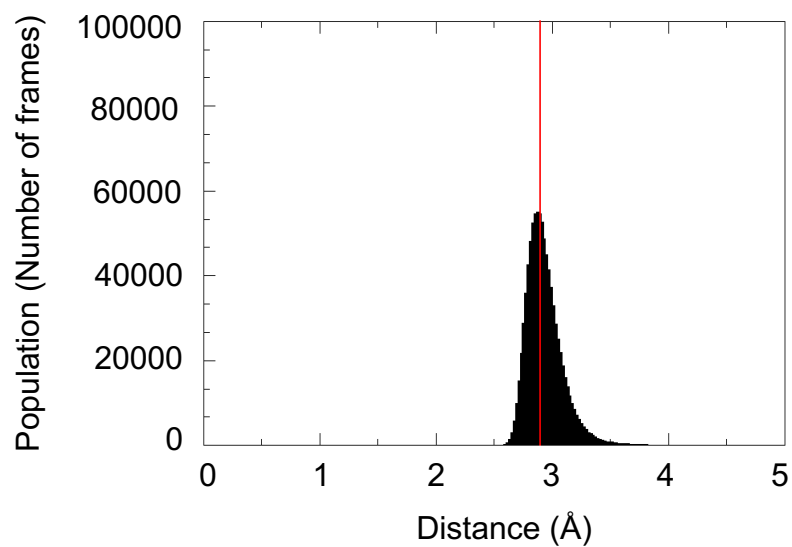

Supplement: Supplementary file 2 — ct2c00291_si_002.zip [file ct2c00291_si_002.zip › Figure S43.pdf]

# Angle duplex O6-N4

## K-TIP3P

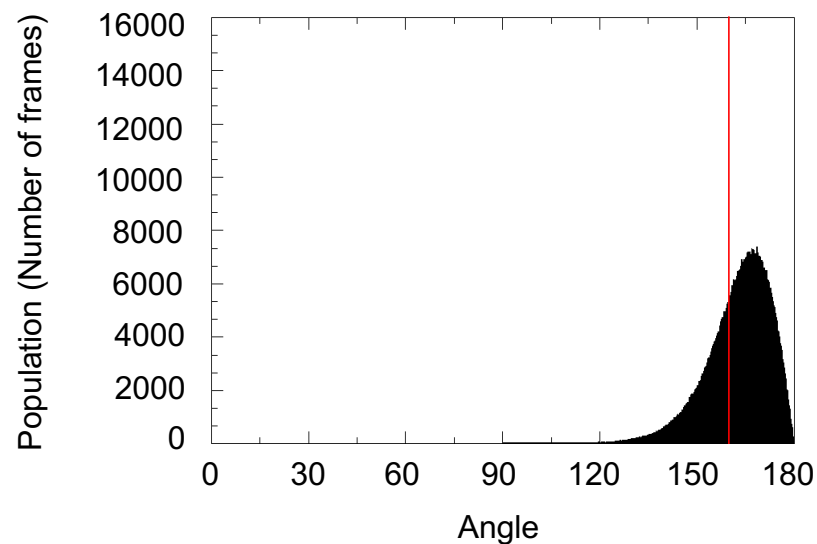

## K-TIP4P

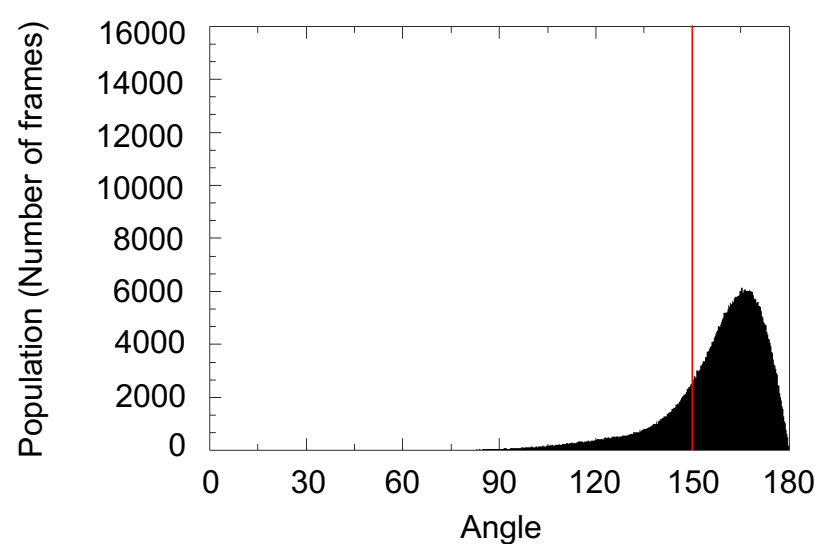

## KCI-TIP3P

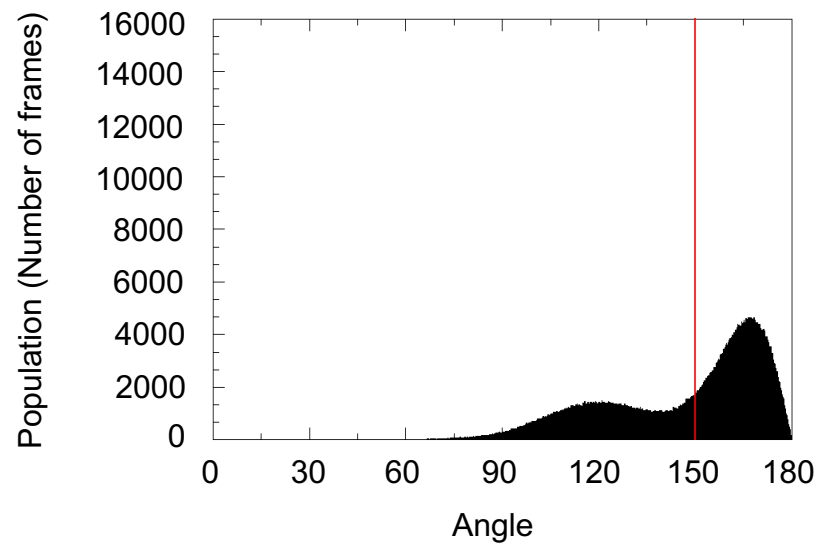

## KCI-TIP4P

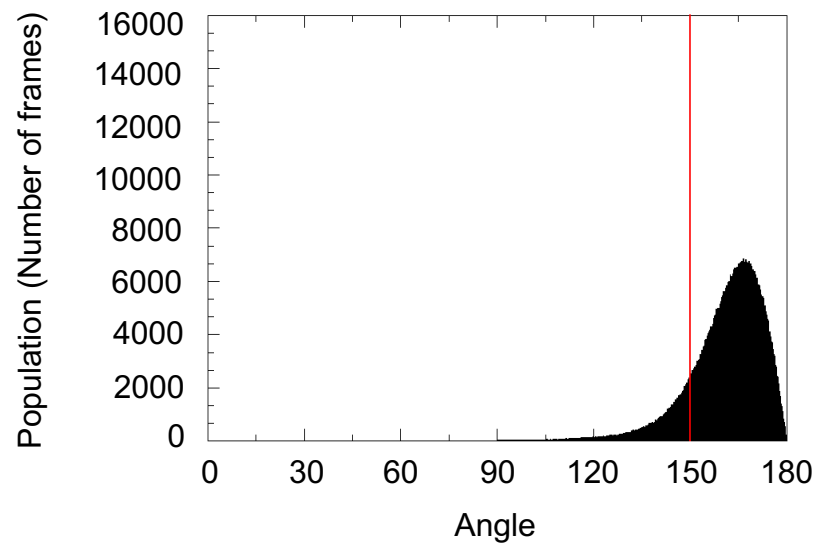

Supplement: Supplementary file 2 — ct2c00291_si_002.zip [file ct2c00291_si_002.zip › Figure S44.pdf]

Residue 1

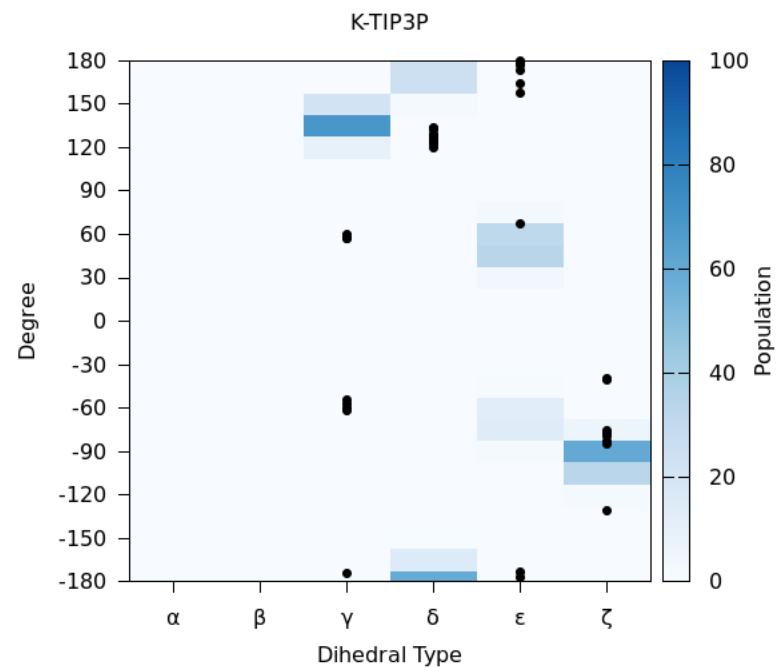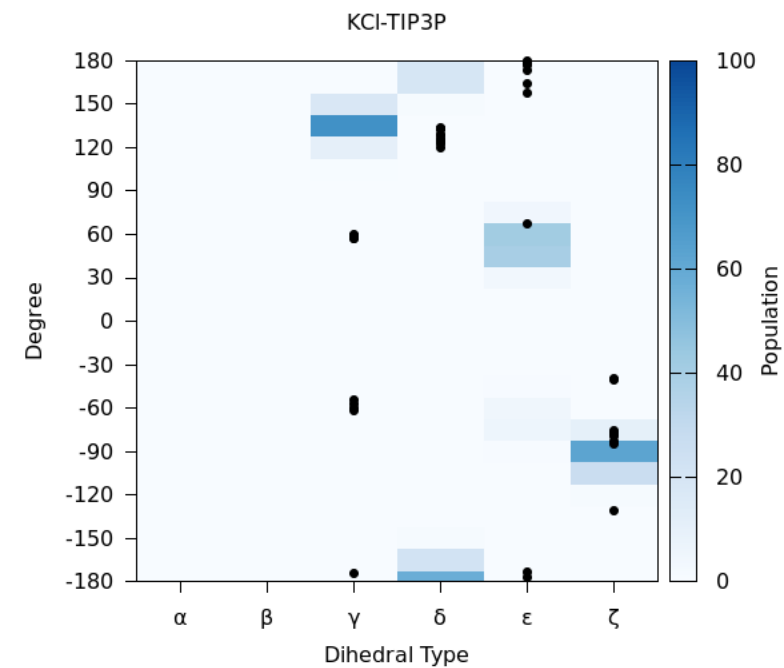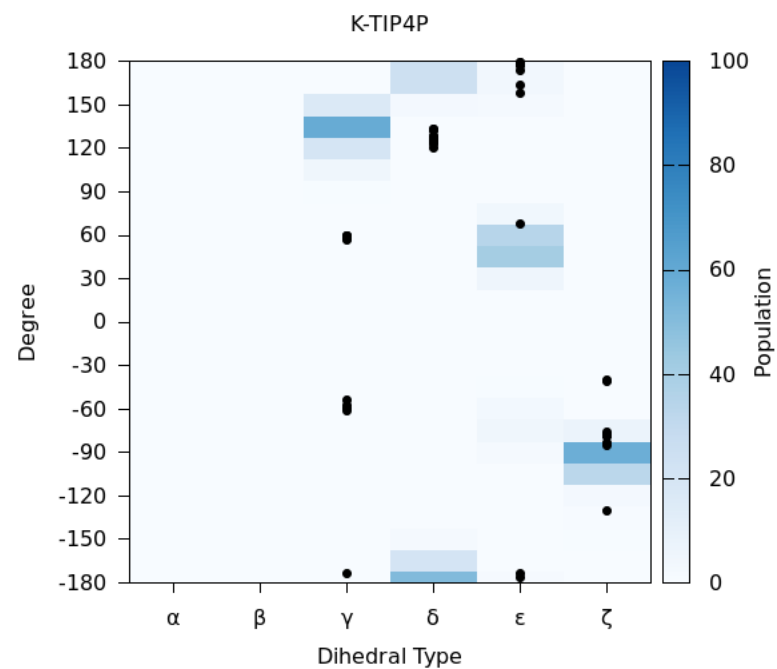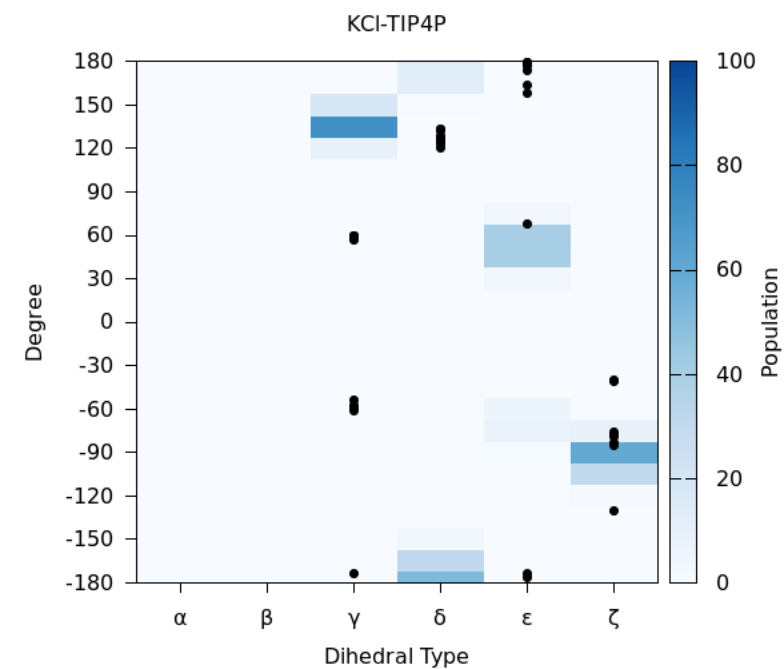

Supplement: Supplementary file 2 — ct2c00291_si_002.zip [file ct2c00291_si_002.zip › Figure S45.pdf]

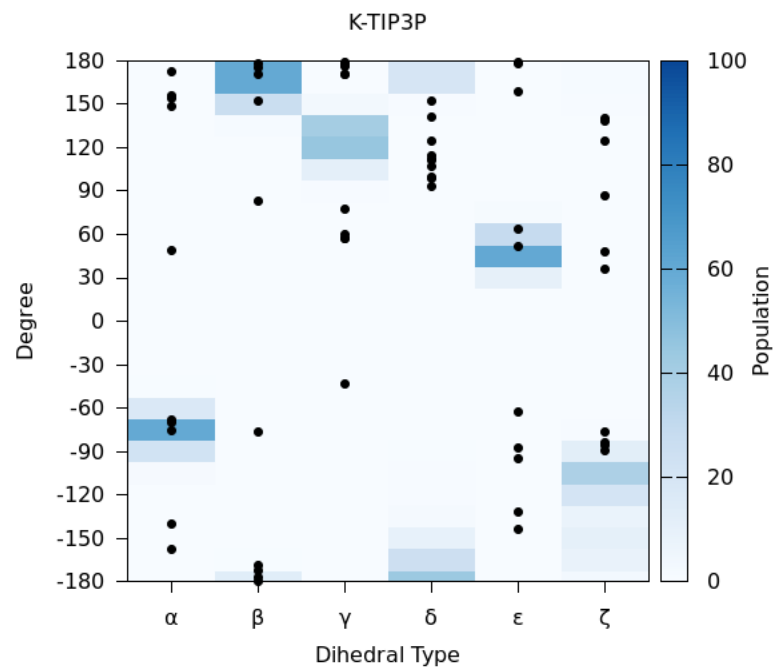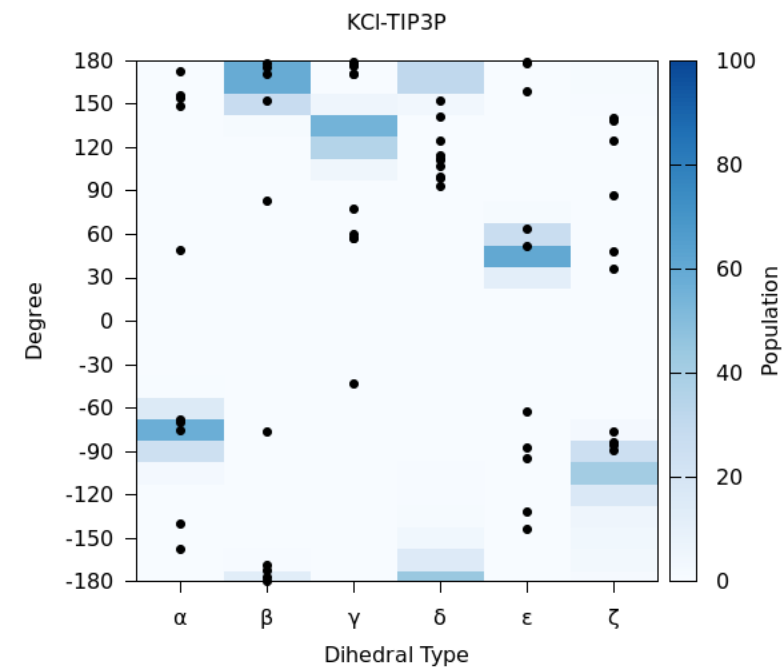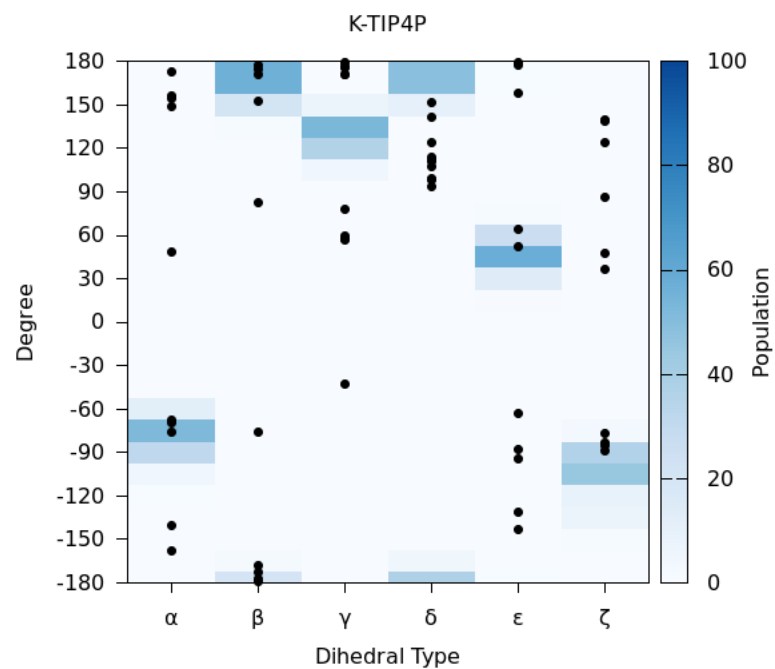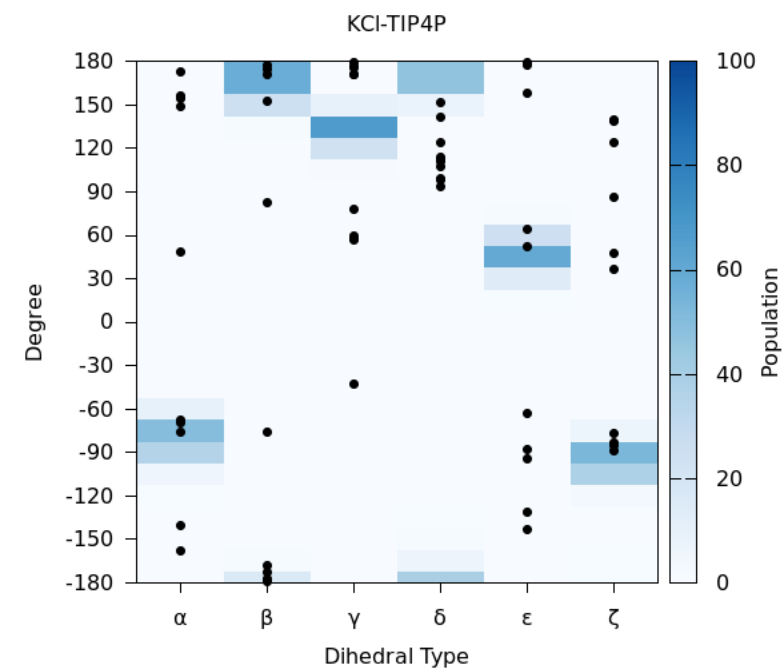

Supplement: Supplementary file 2 — ct2c00291_si_002.zip [file ct2c00291_si_002.zip › Figure S46.pdf]

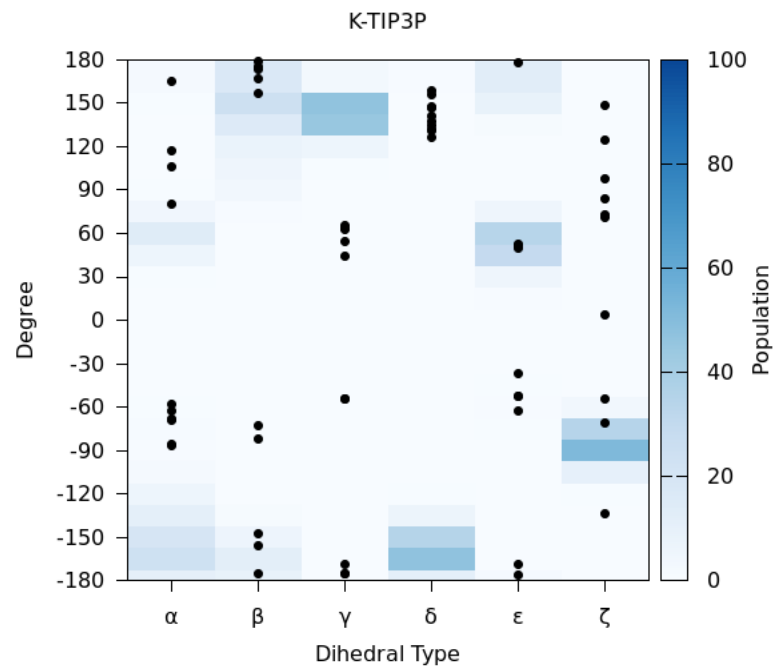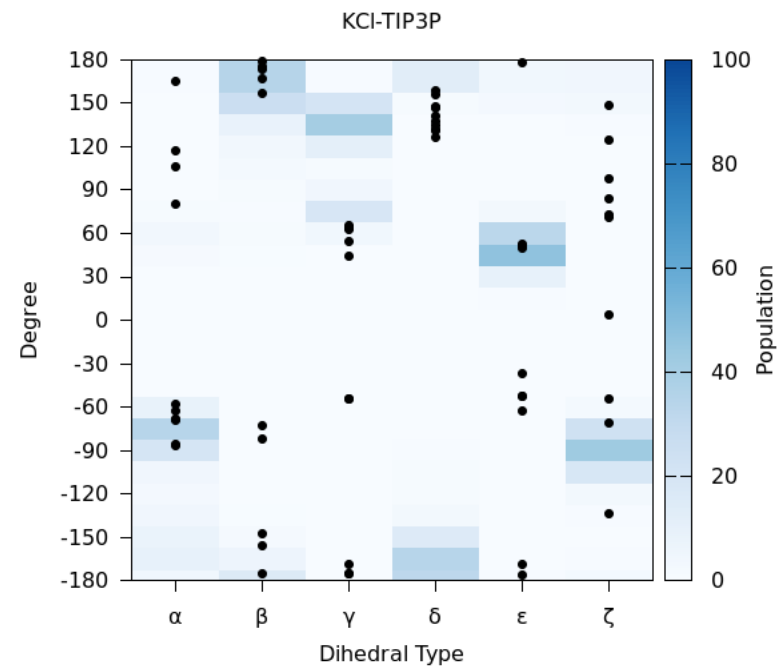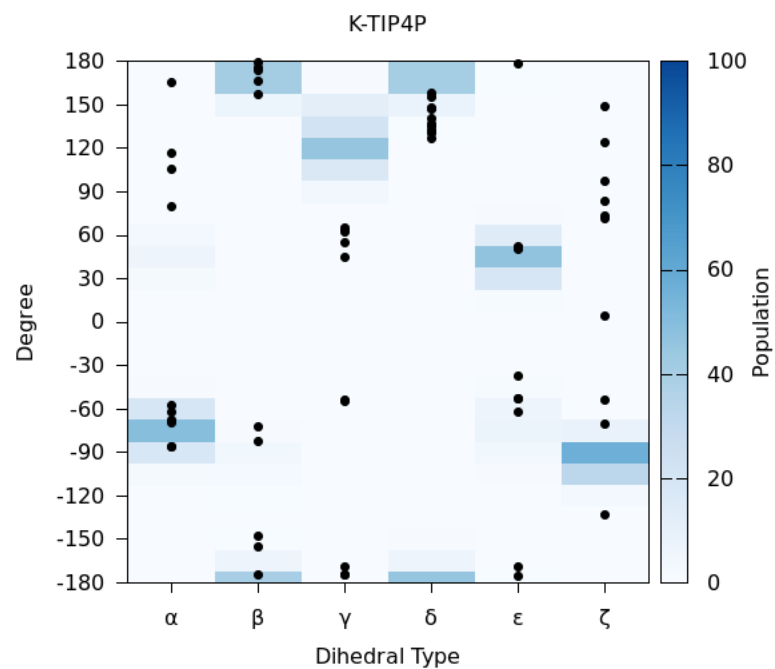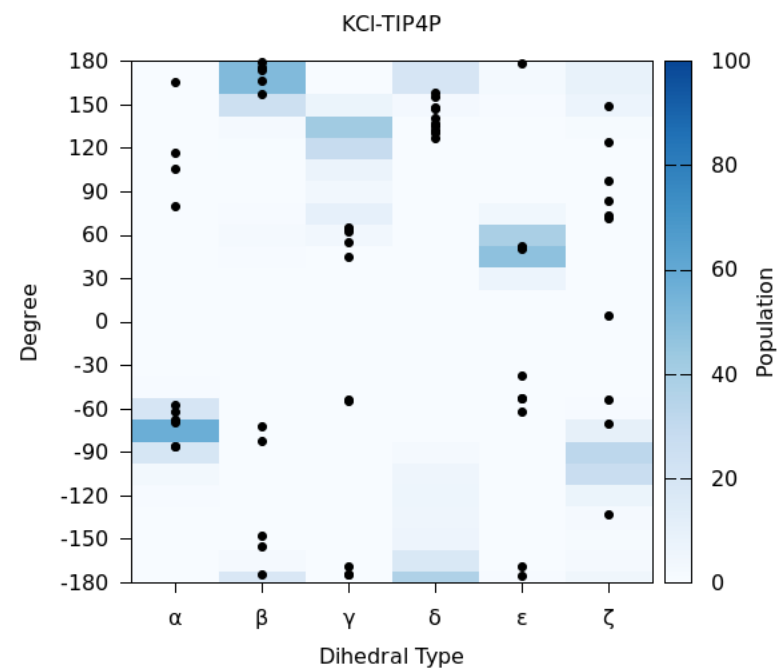

Supplement: Supplementary file 2 — ct2c00291_si_002.zip [file ct2c00291_si_002.zip › Figure S47.pdf]

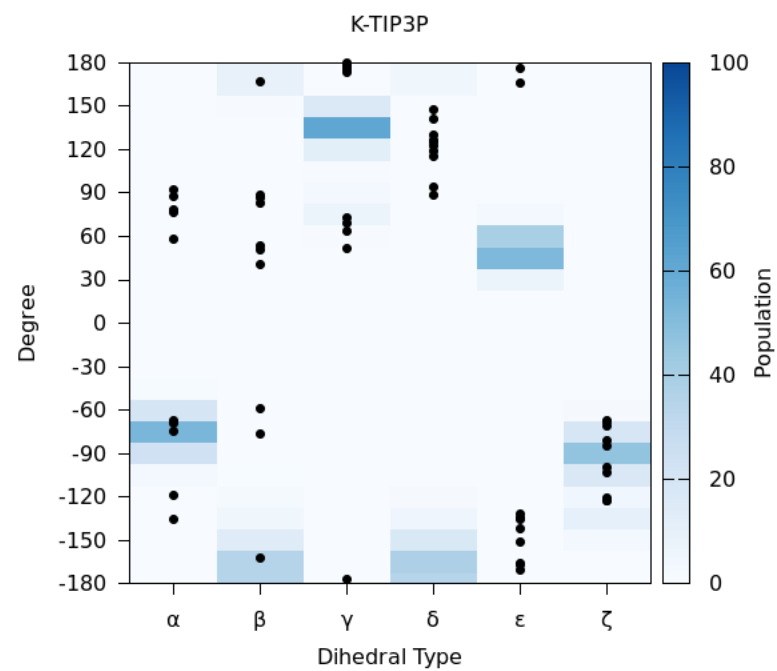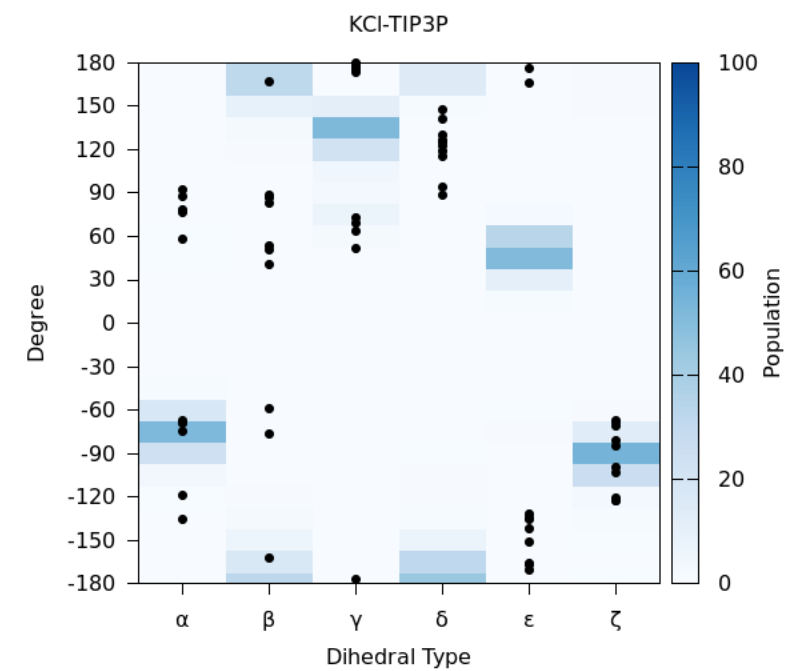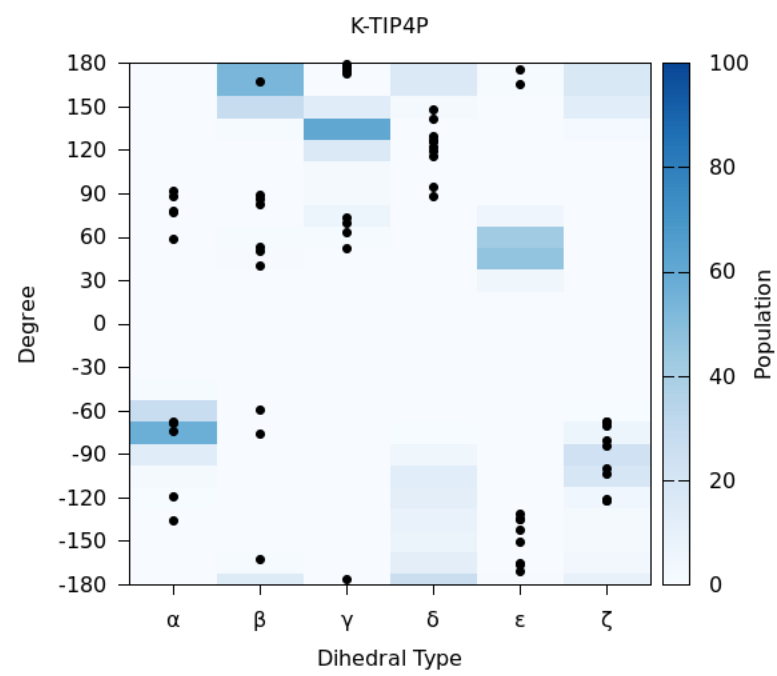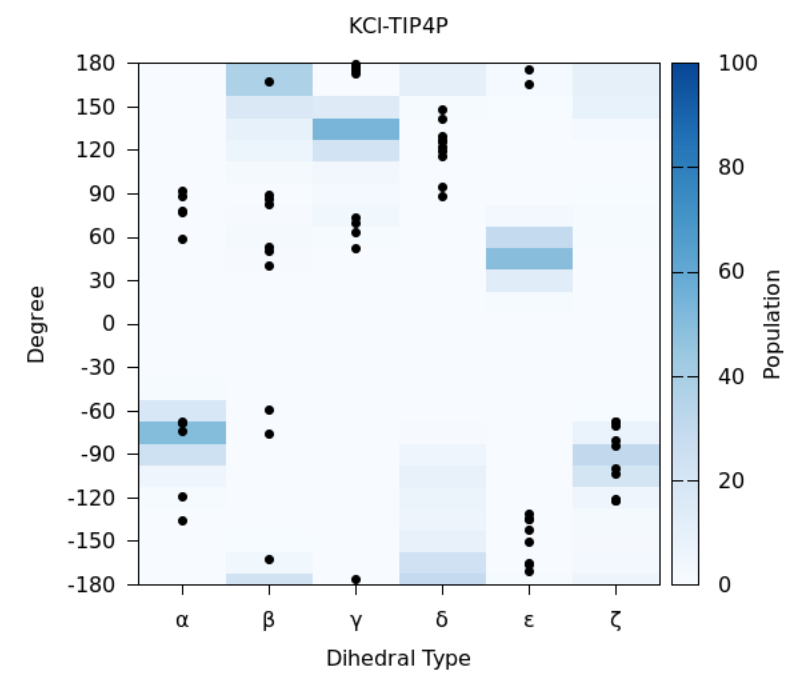

Supplement: Supplementary file 2 — ct2c00291_si_002.zip [file ct2c00291_si_002.zip › Figure S48.pdf]

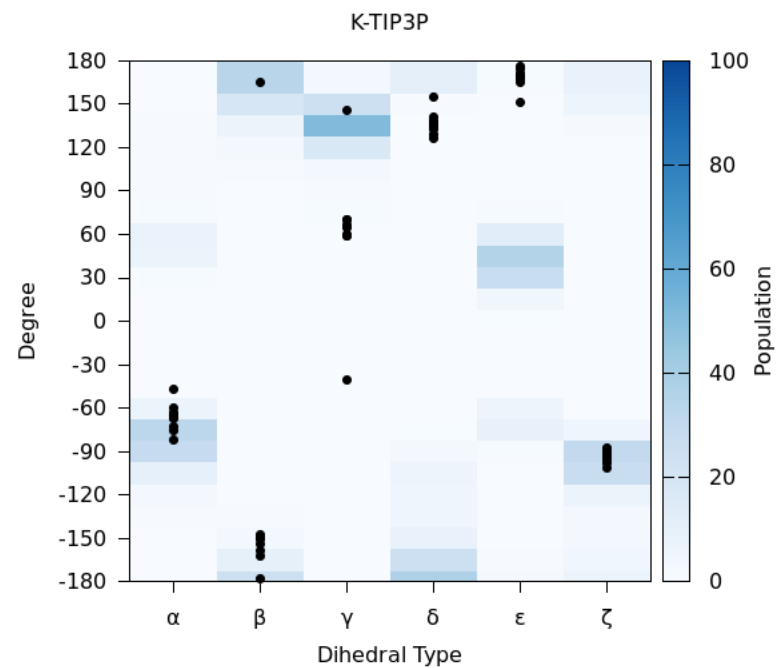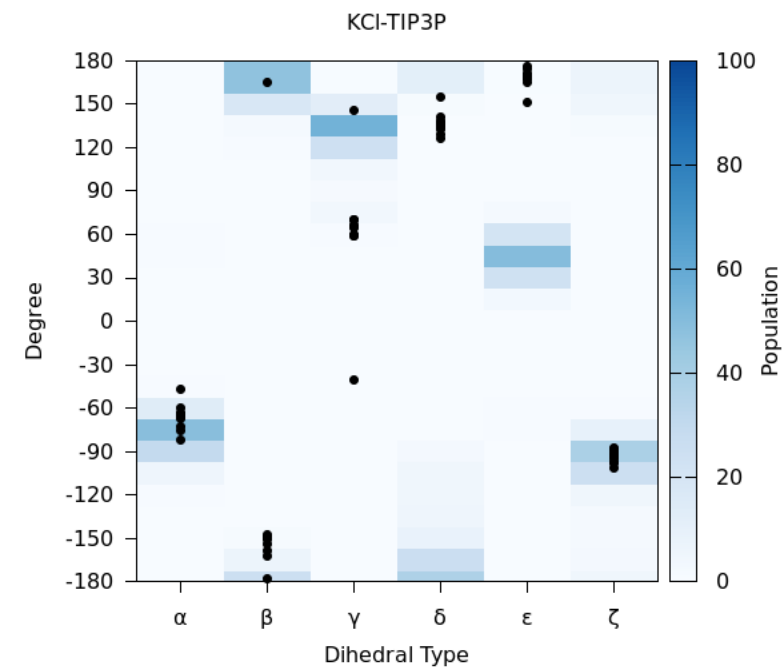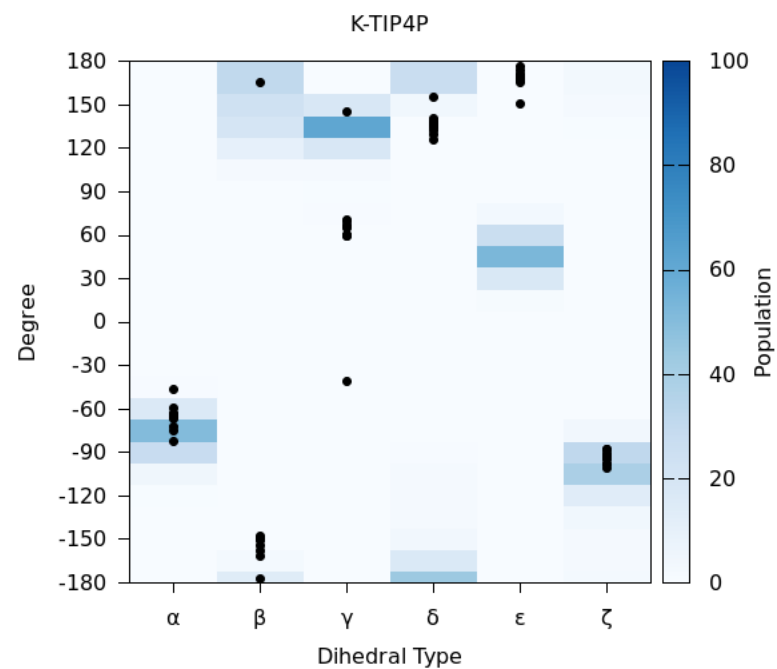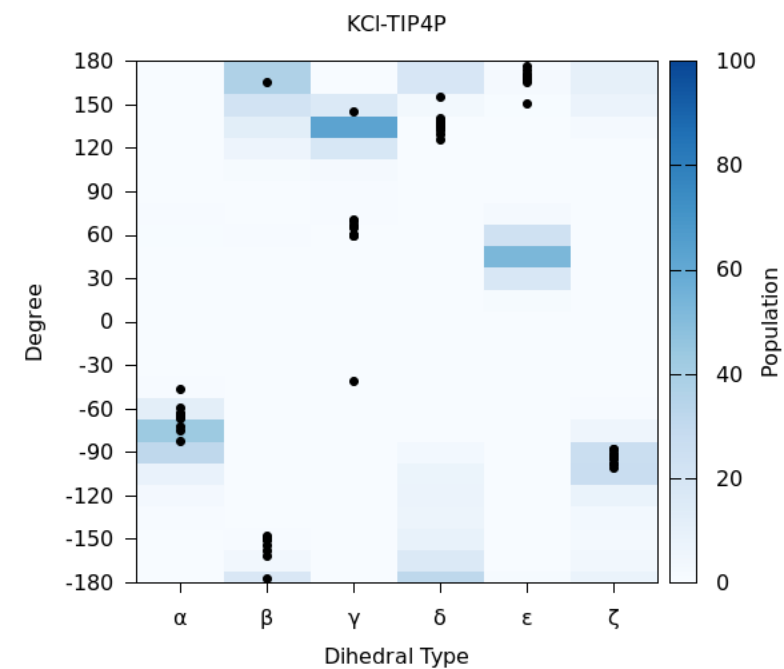

Supplement: Supplementary file 2 — ct2c00291_si_002.zip [file ct2c00291_si_002.zip › Figure S49.pdf]

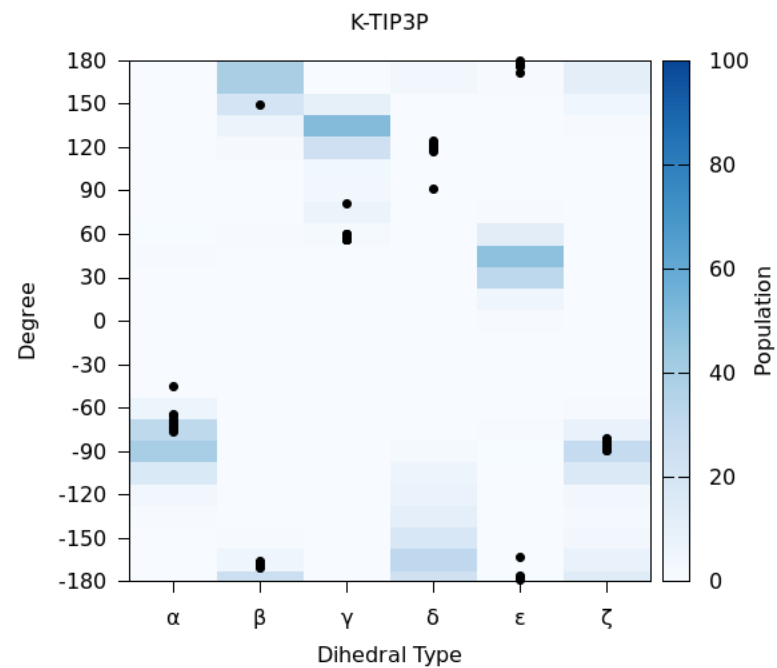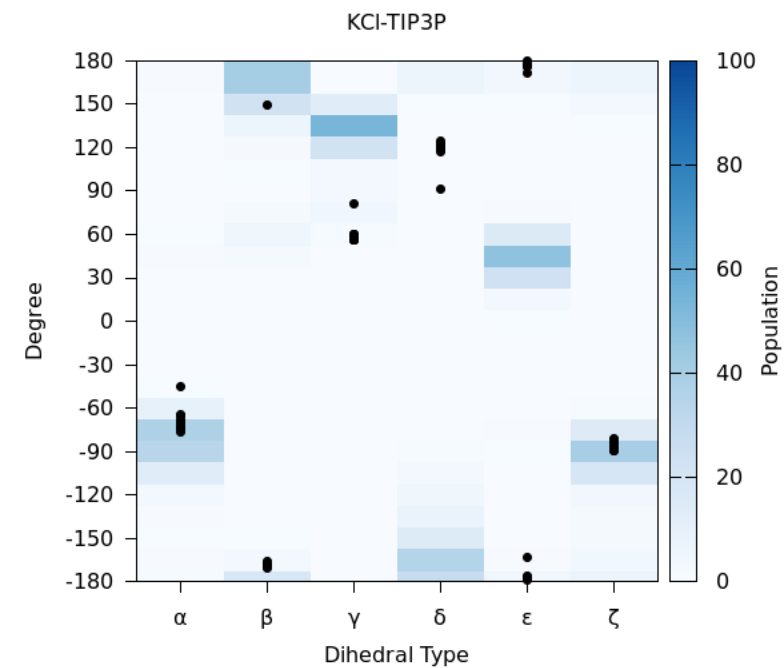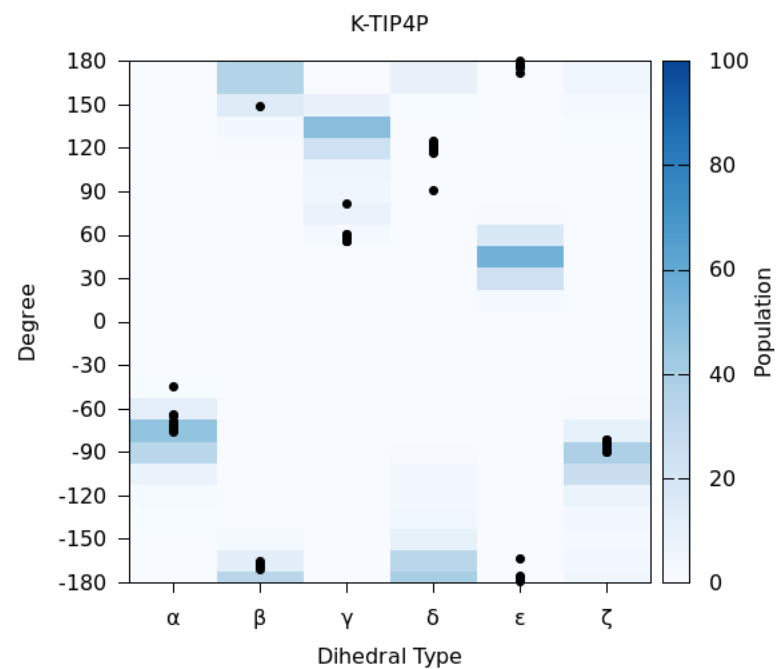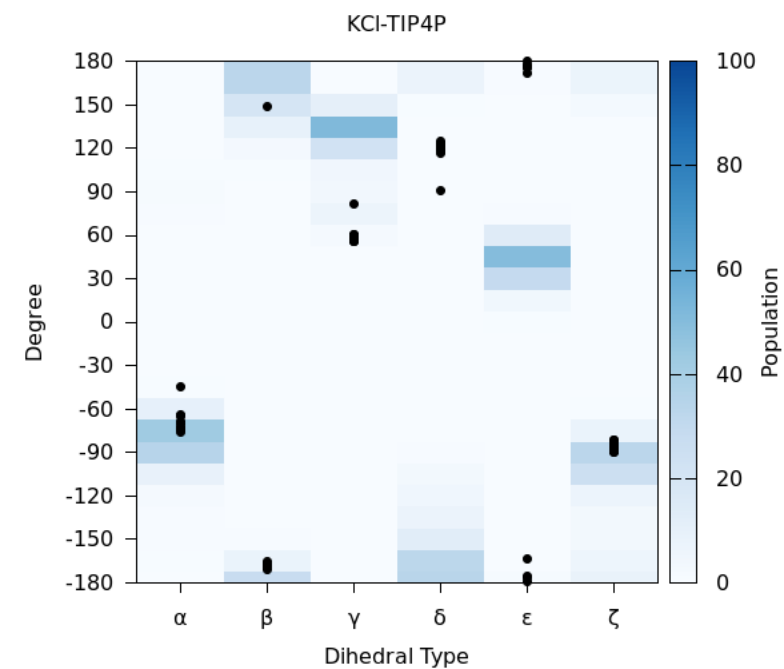

Supplement: Supplementary file 2 — ct2c00291_si_002.zip [file ct2c00291_si_002.zip › Figure S50.pdf]

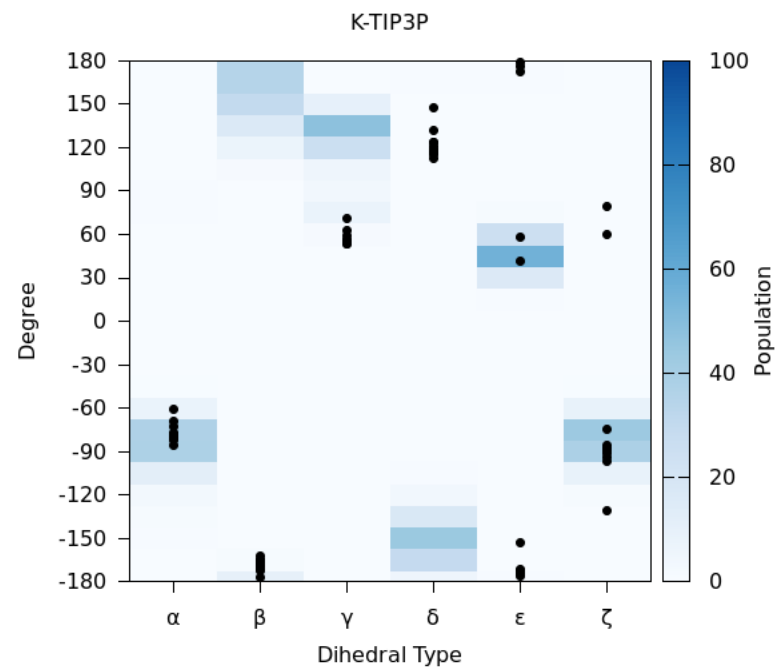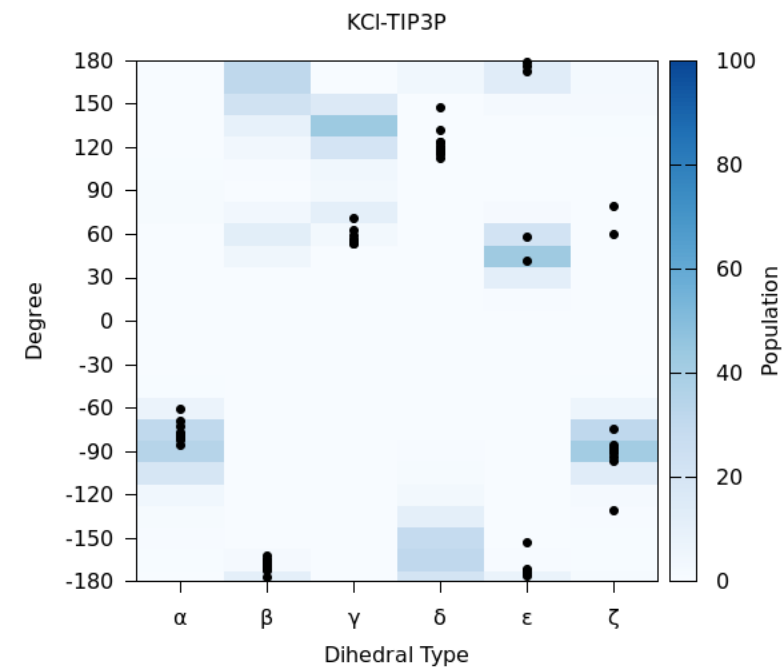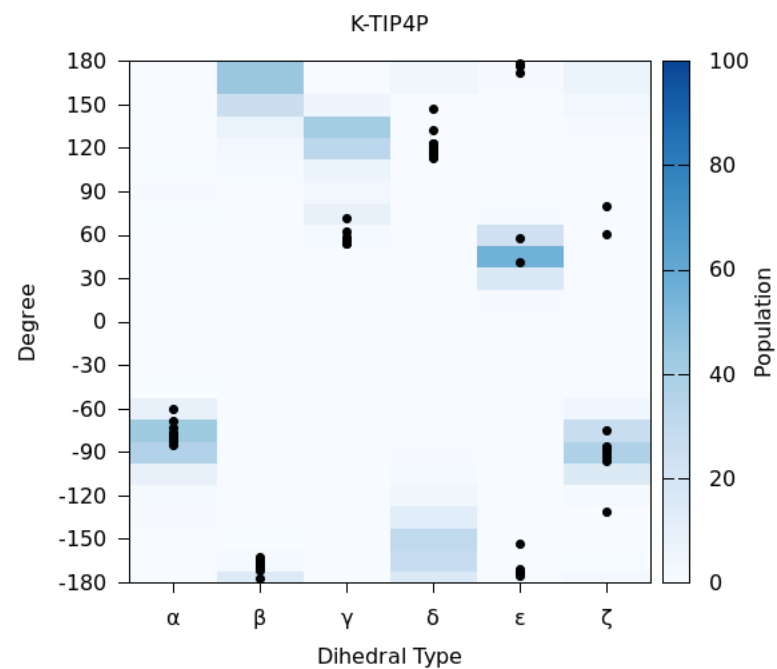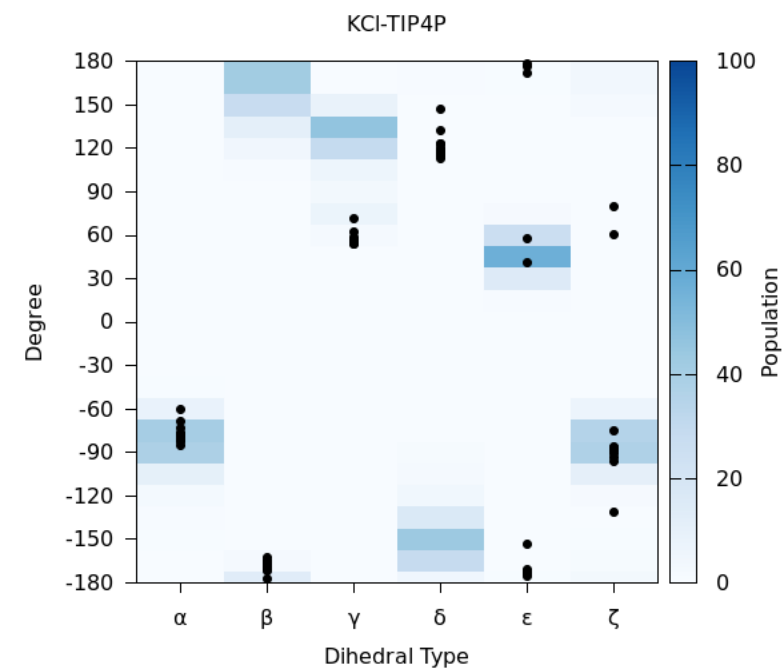

Supplement: Supplementary file 2 — ct2c00291_si_002.zip [file ct2c00291_si_002.zip › Figure S51.pdf]

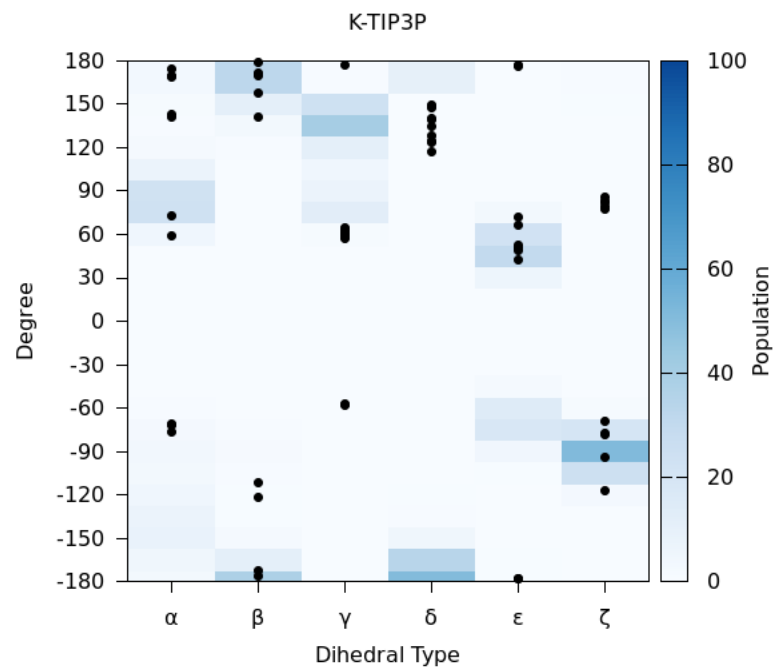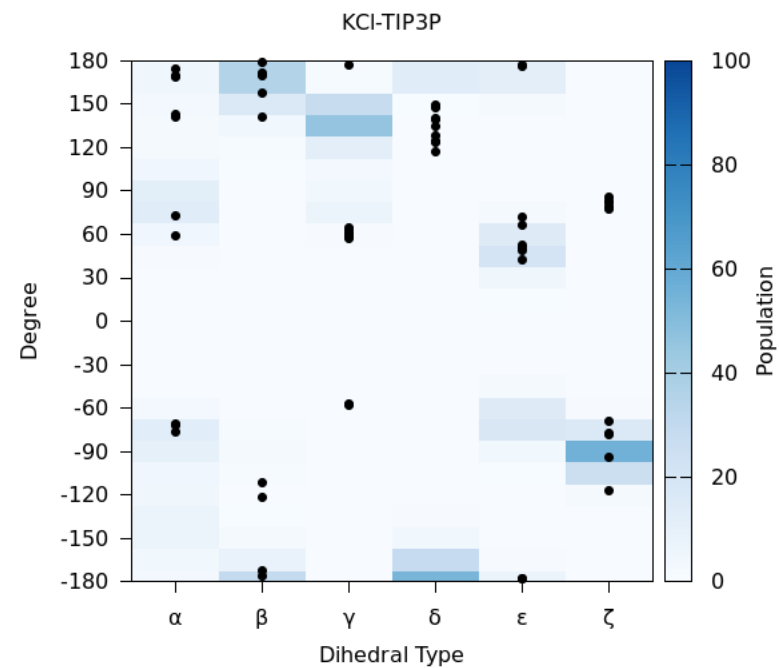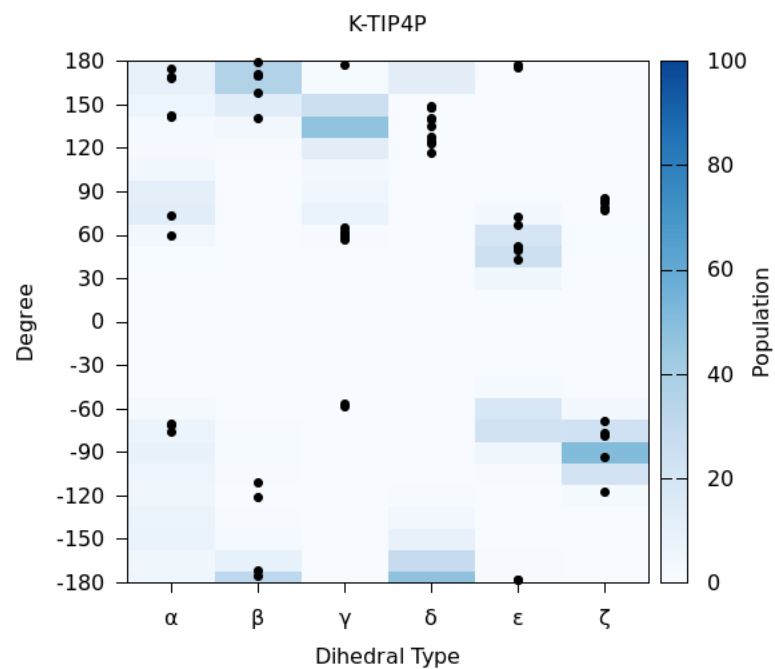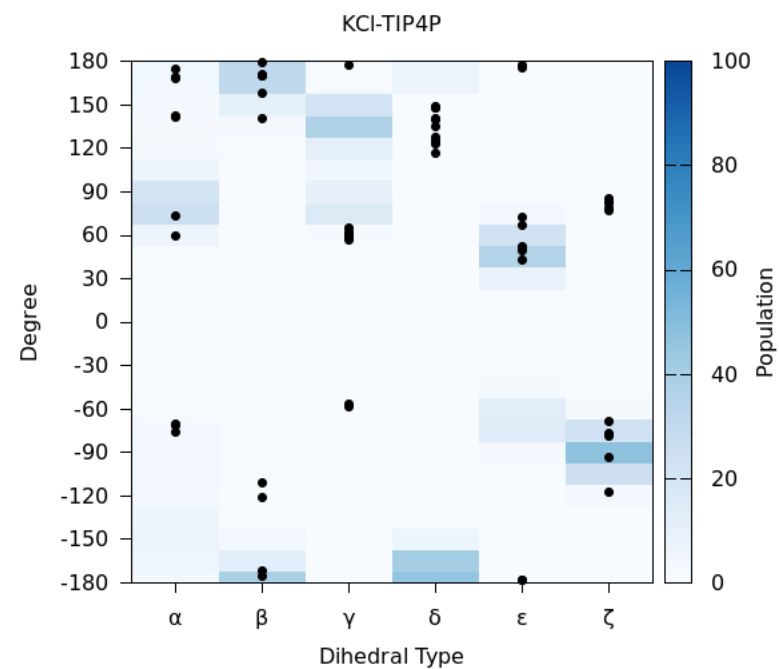

Supplement: Supplementary file 2 — ct2c00291_si_002.zip [file ct2c00291_si_002.zip › Figure S52.pdf]

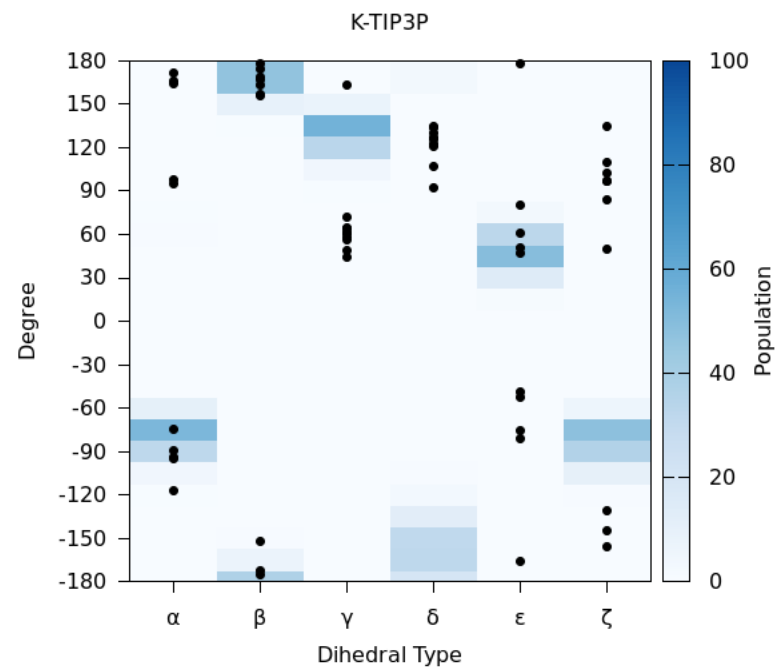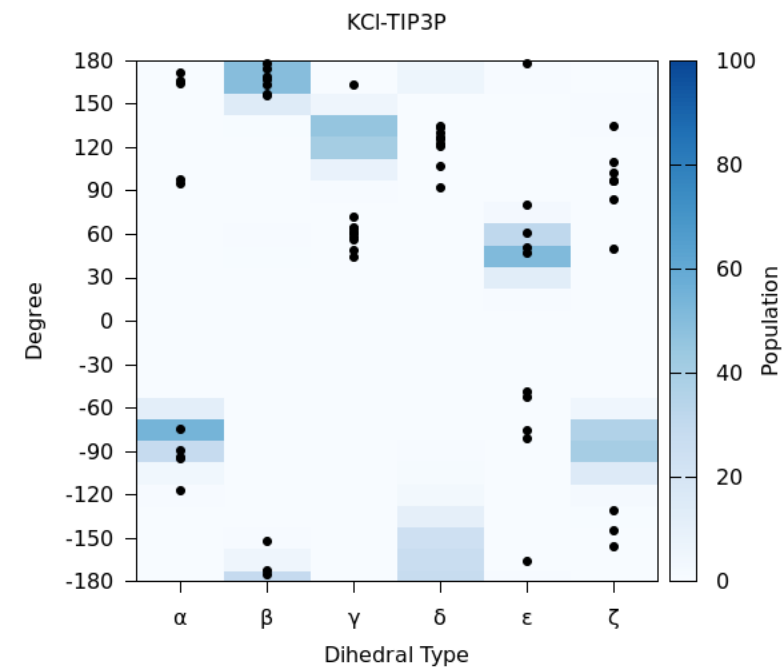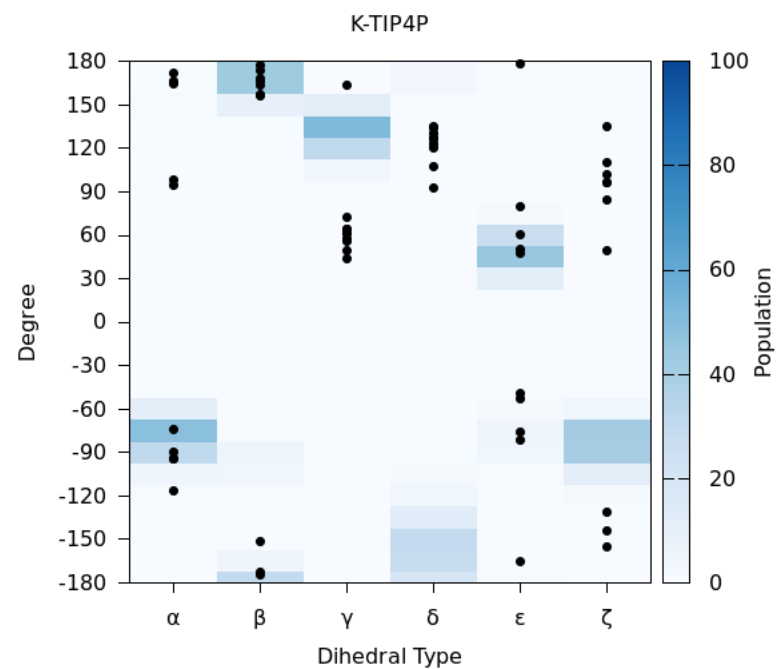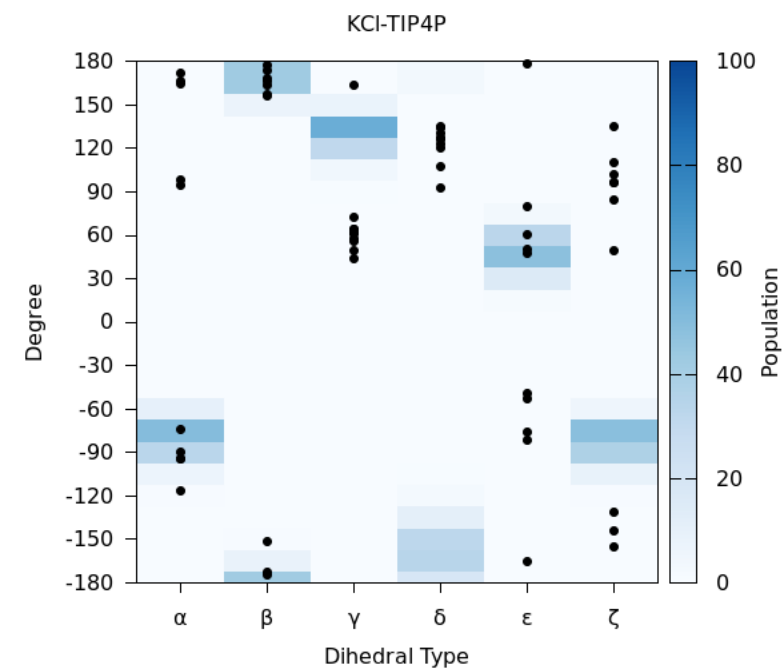

Supplement: Supplementary file 2 — ct2c00291_si_002.zip [file ct2c00291_si_002.zip › Figure S53.pdf]

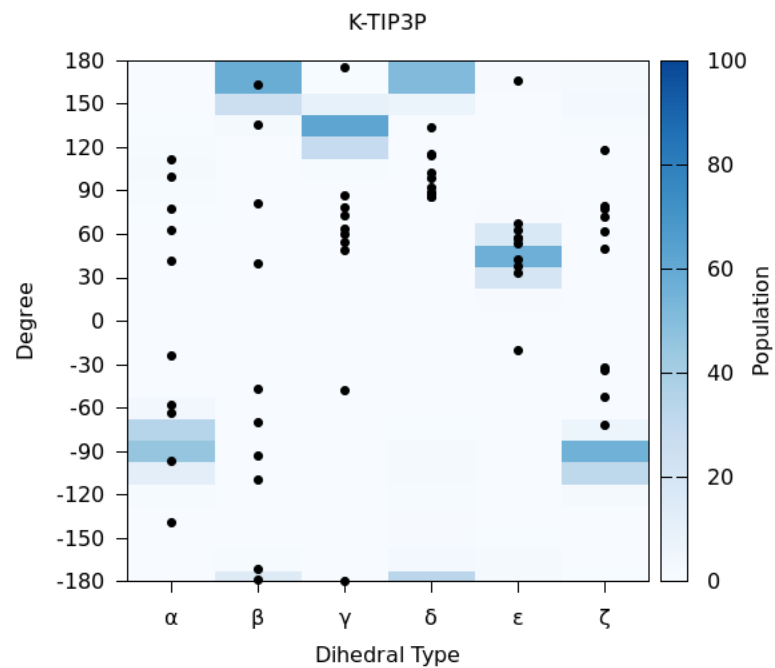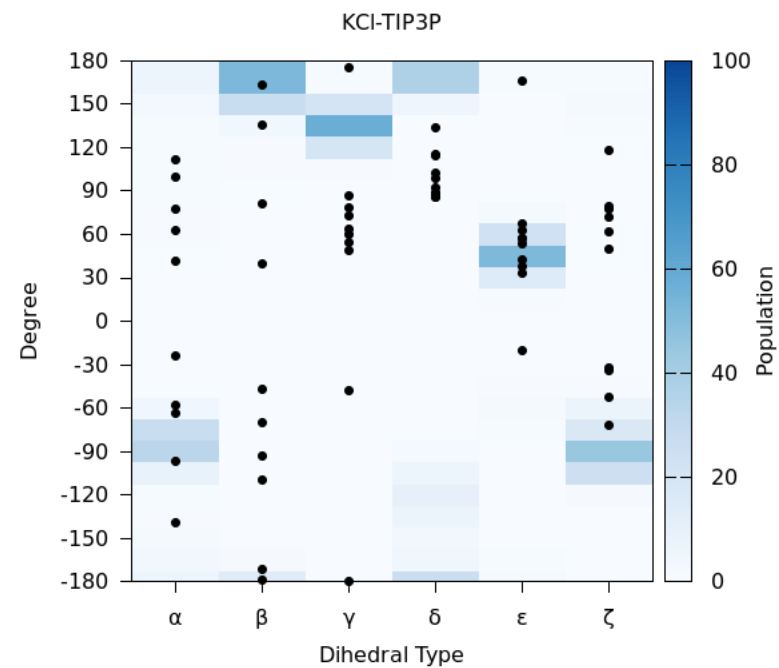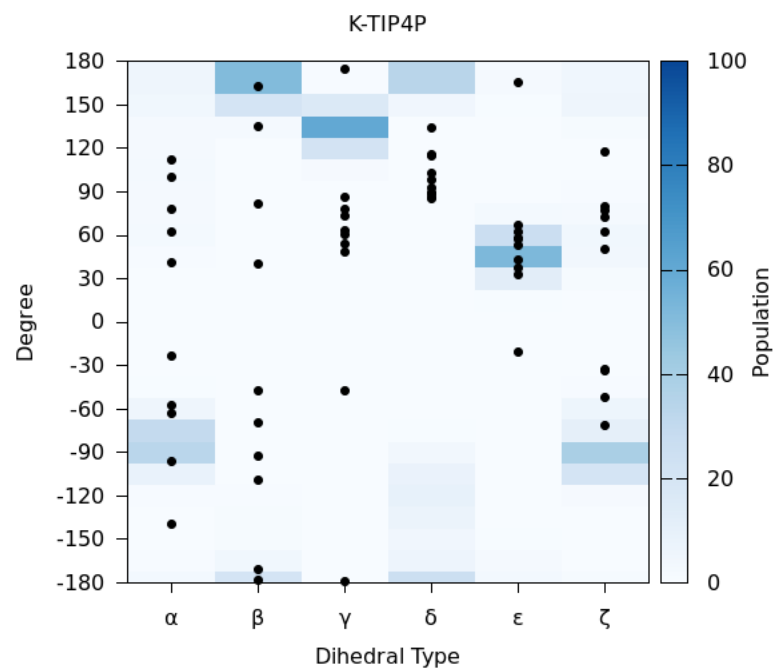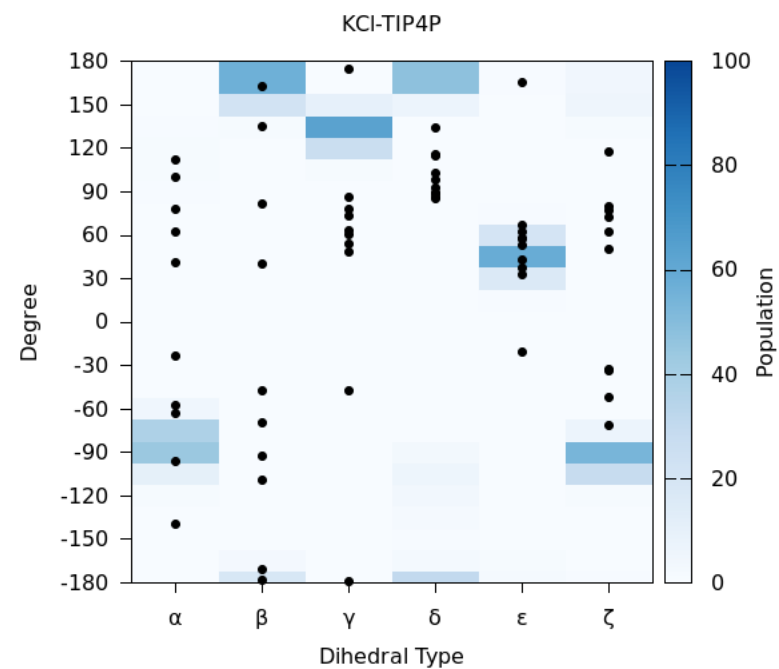

Supplement: Supplementary file 2 — ct2c00291_si_002.zip [file ct2c00291_si_002.zip › Figure S54.pdf]

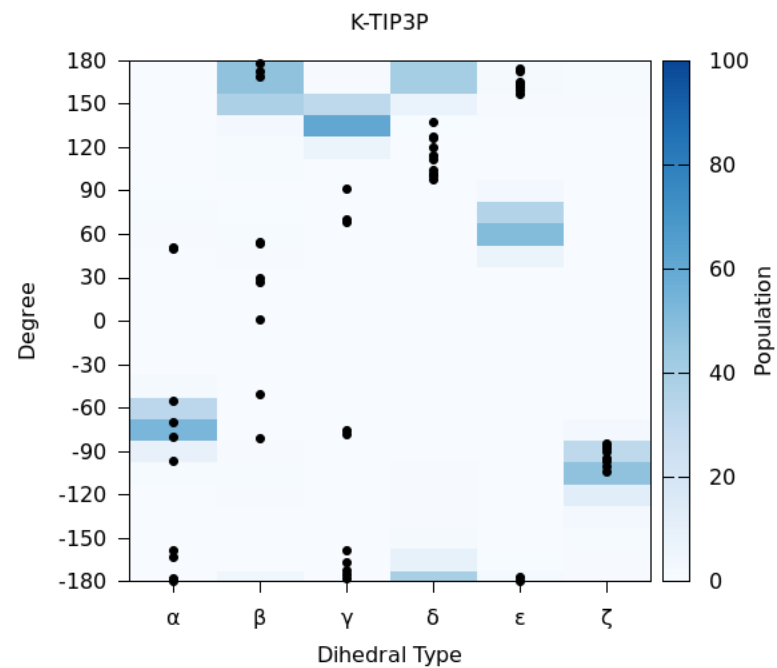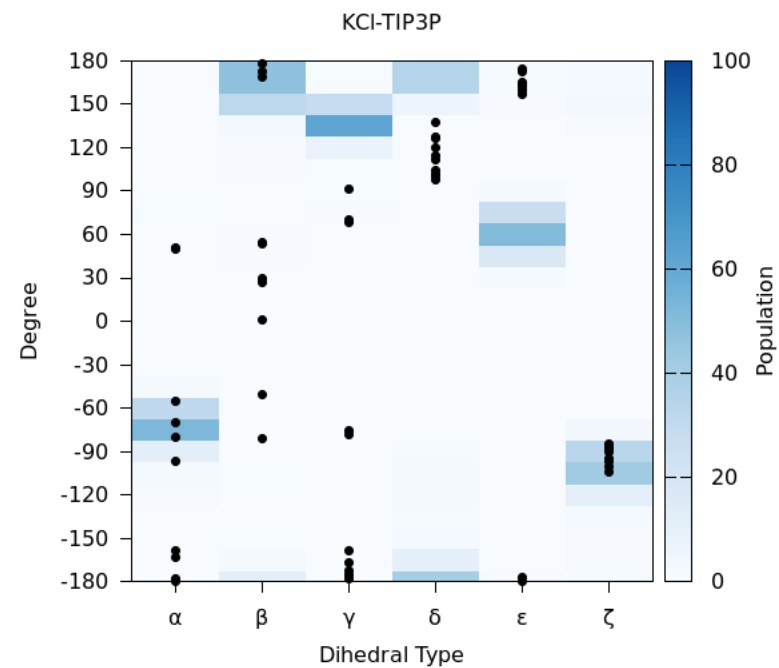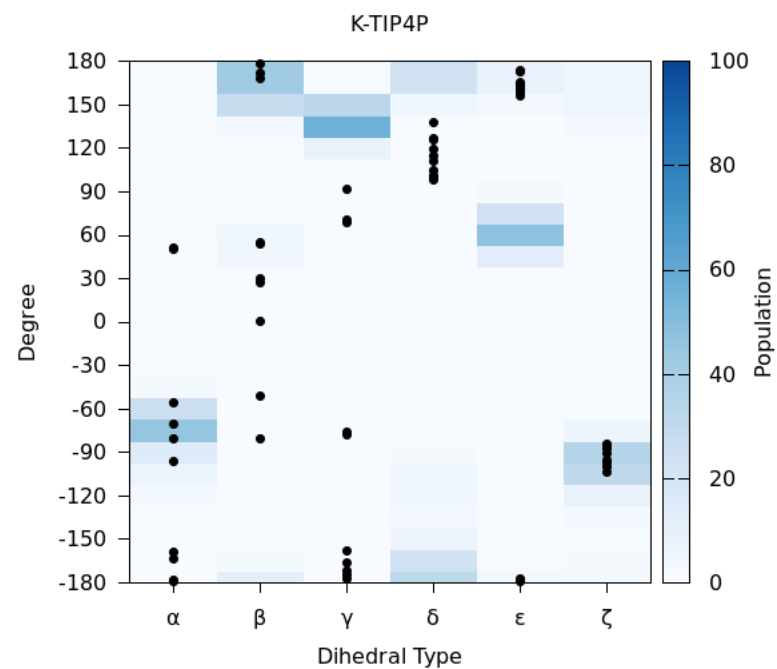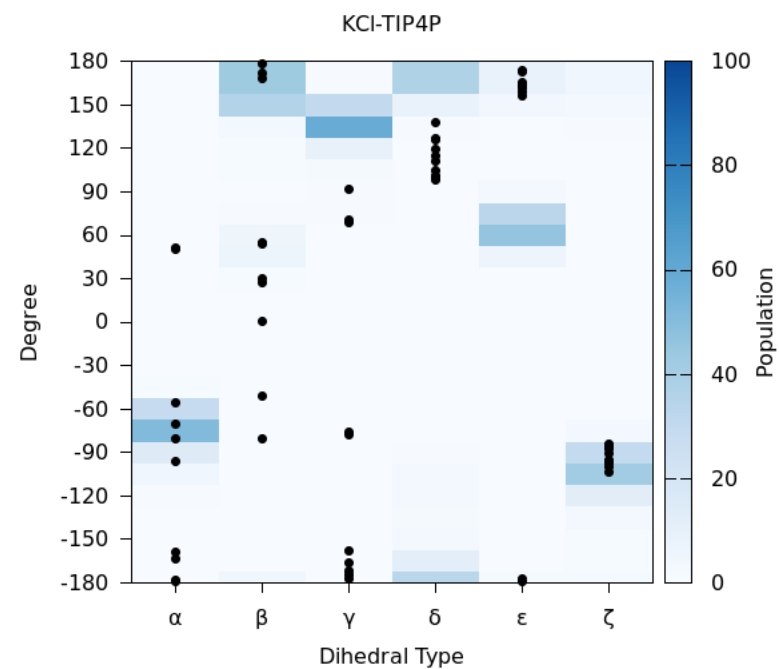

Supplement: Supplementary file 2 — ct2c00291_si_002.zip [file ct2c00291_si_002.zip › Figure S55.pdf]

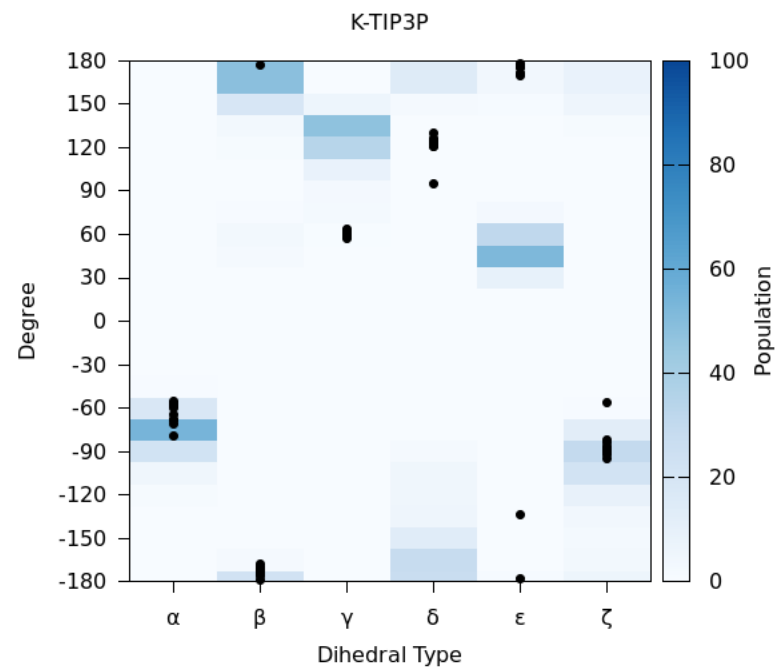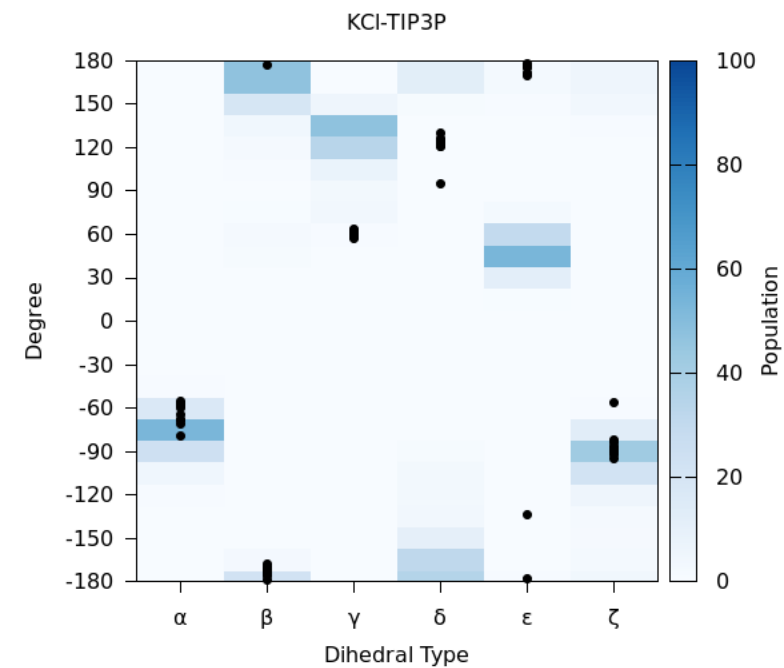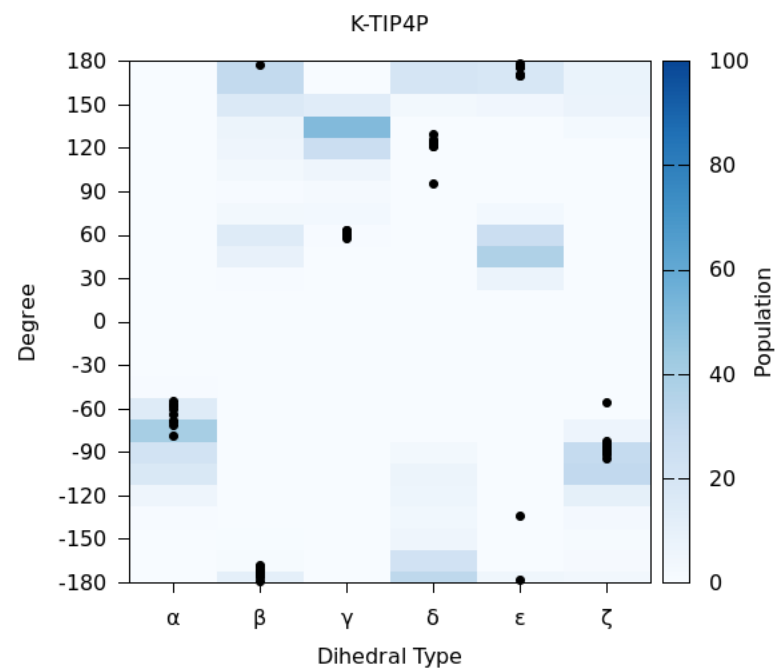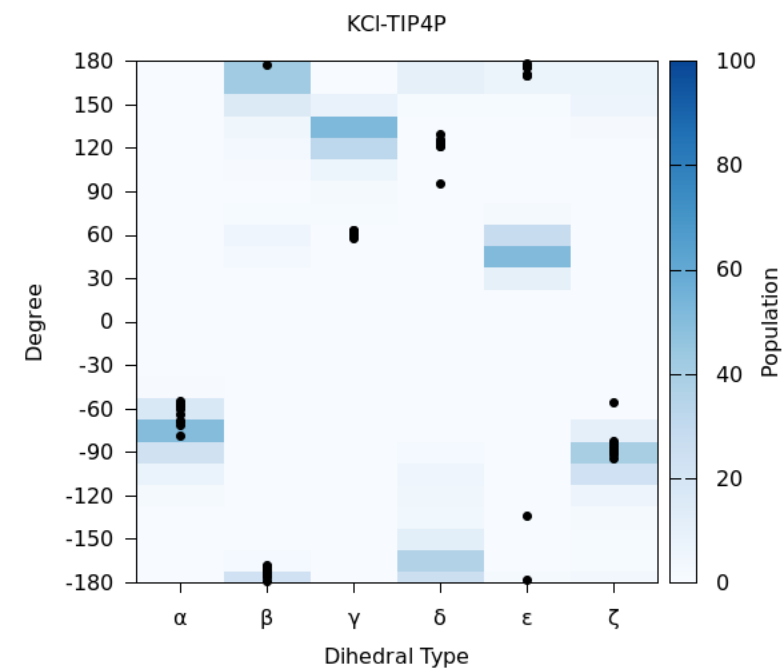

Supplement: Supplementary file 2 — ct2c00291_si_002.zip [file ct2c00291_si_002.zip › Figure S56.pdf]

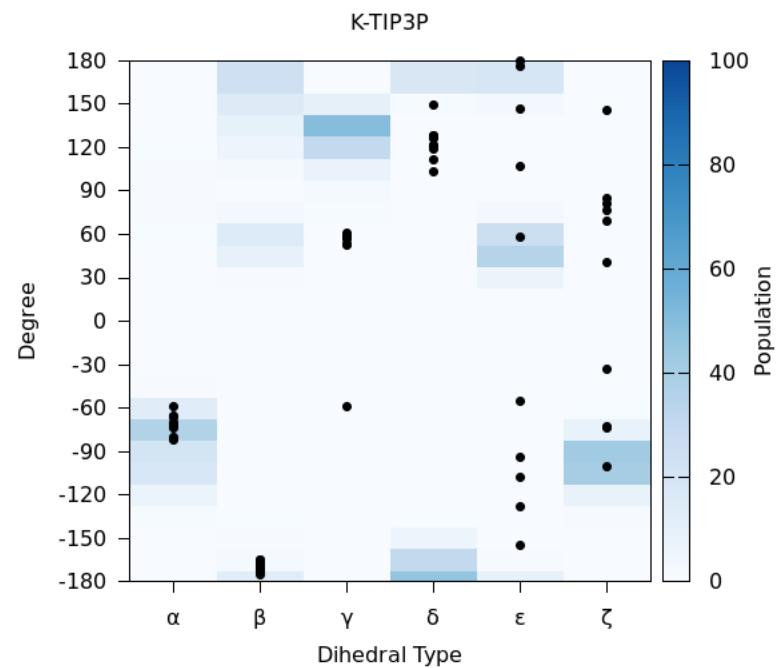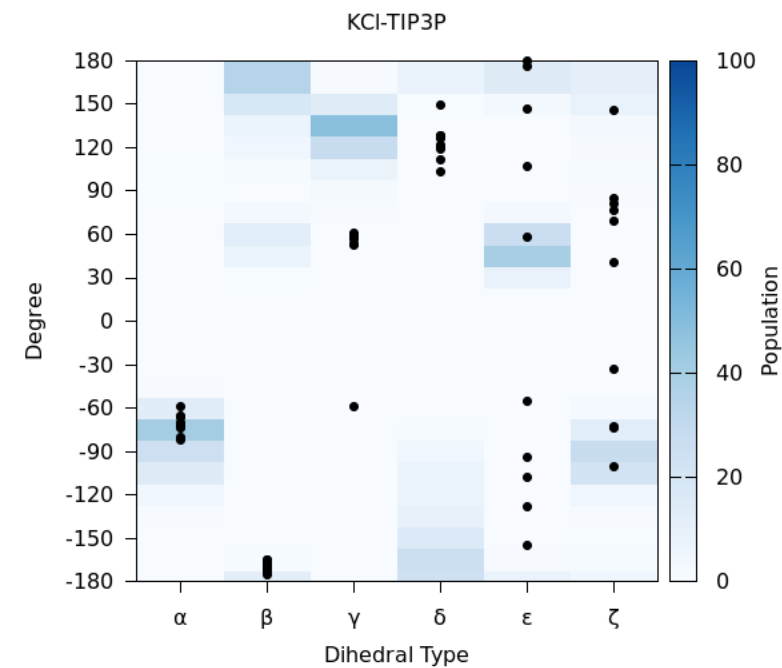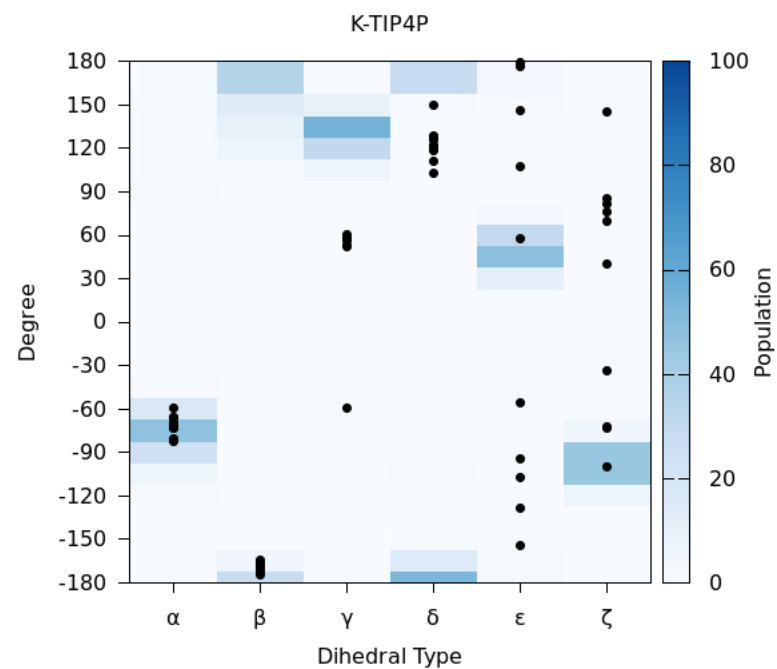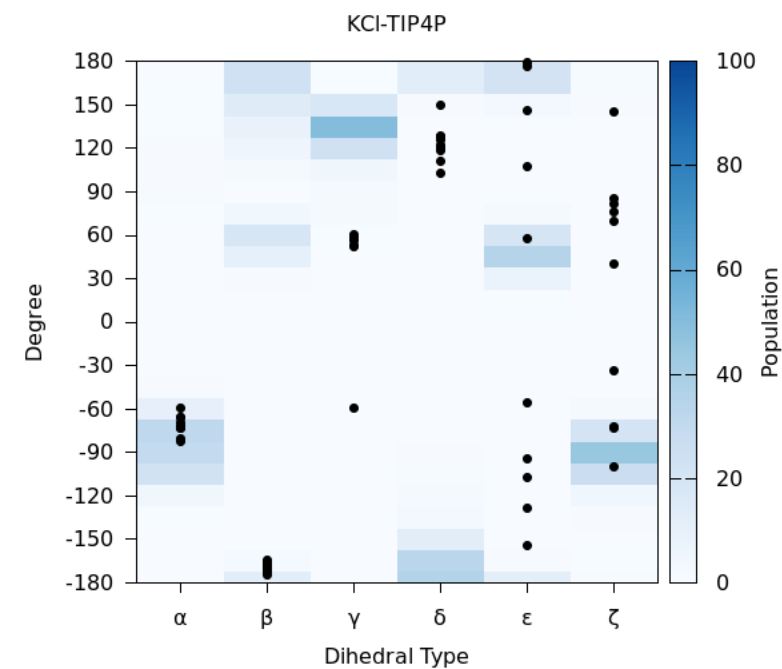

Supplement: Supplementary file 2 — ct2c00291_si_002.zip [file ct2c00291_si_002.zip › Figure S57.pdf]

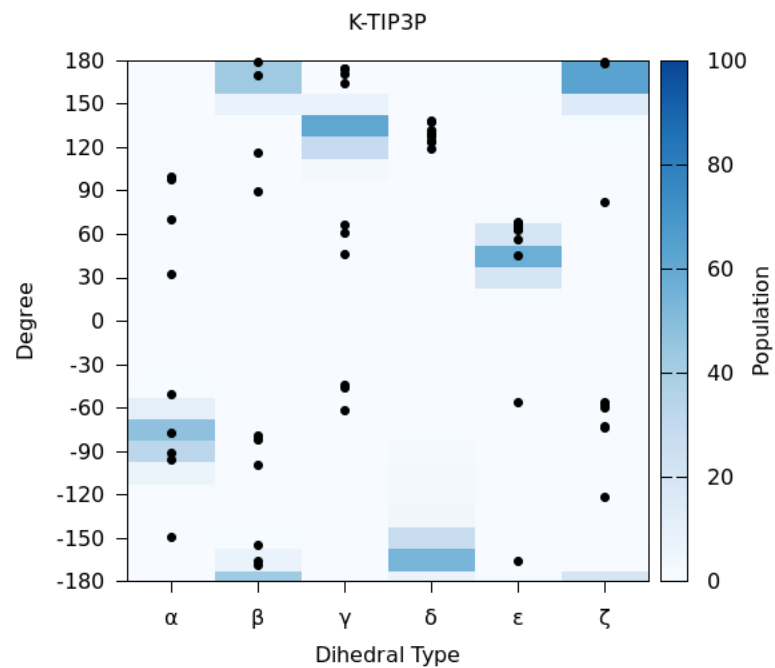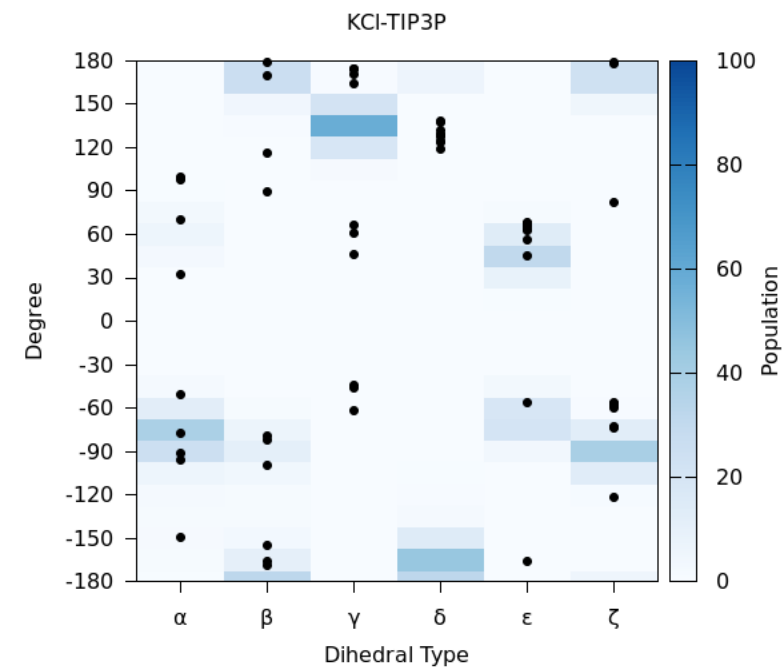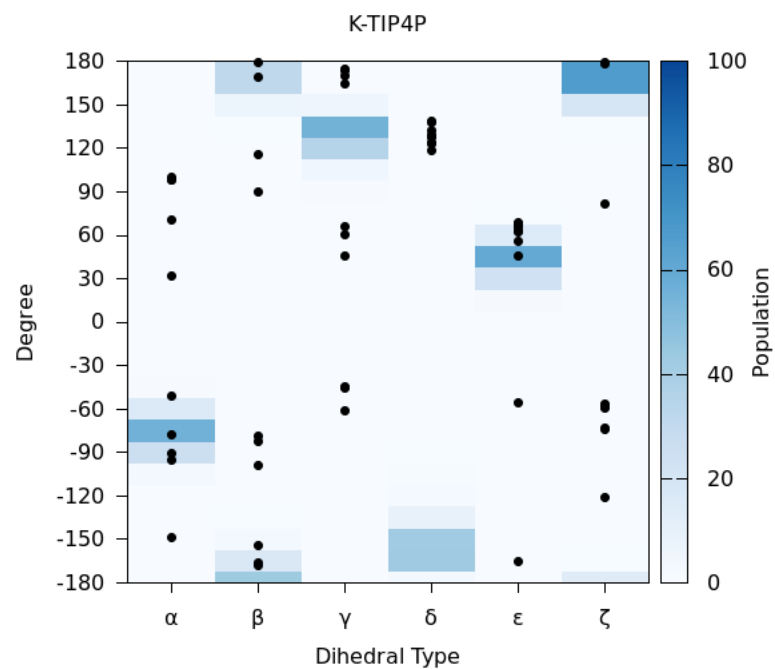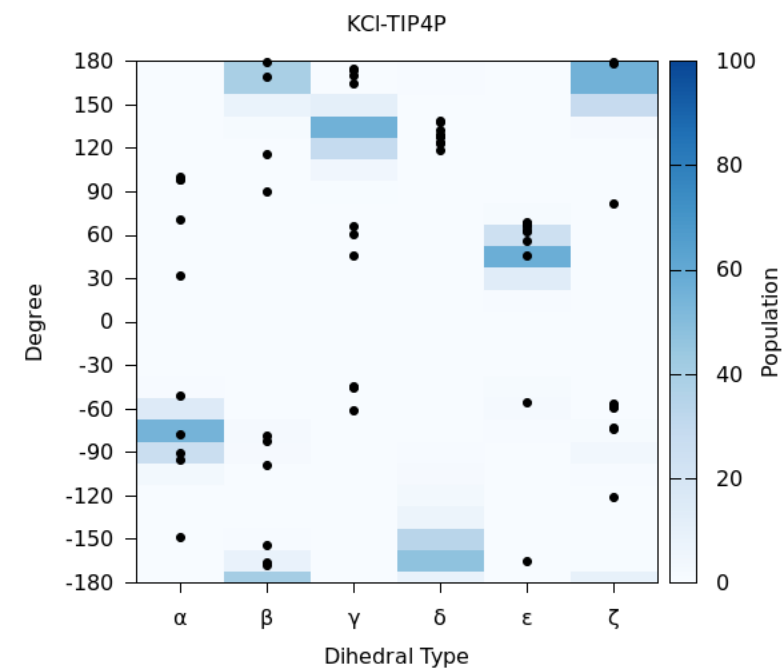

Supplement: Supplementary file 2 — ct2c00291_si_002.zip [file ct2c00291_si_002.zip › Figure S58.pdf]

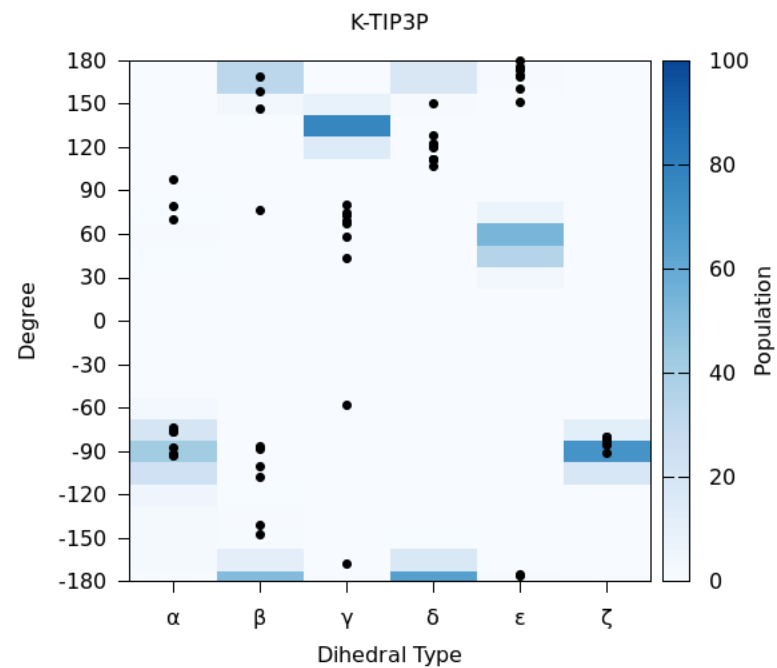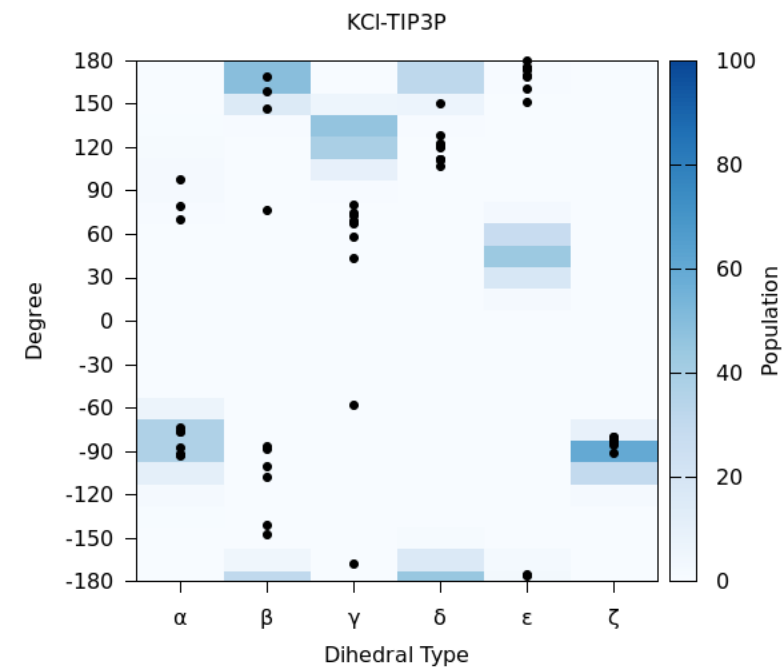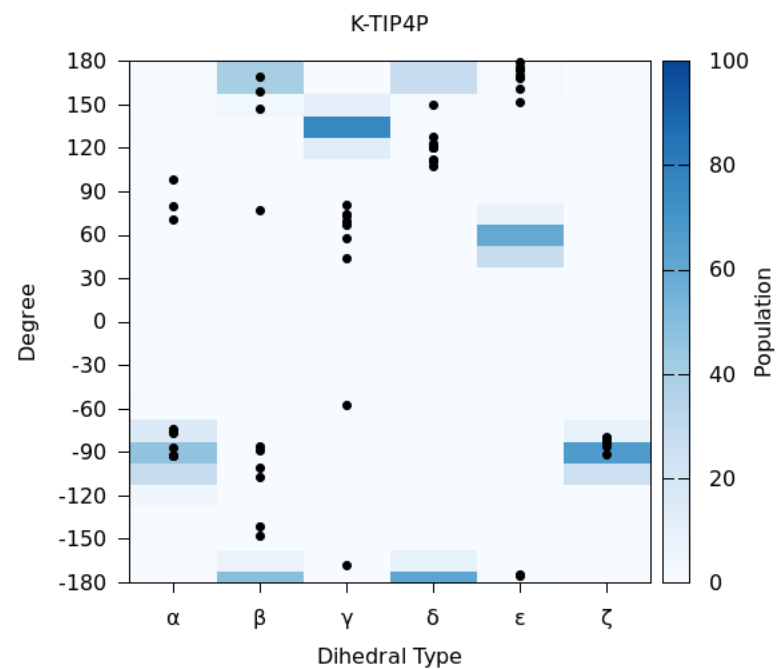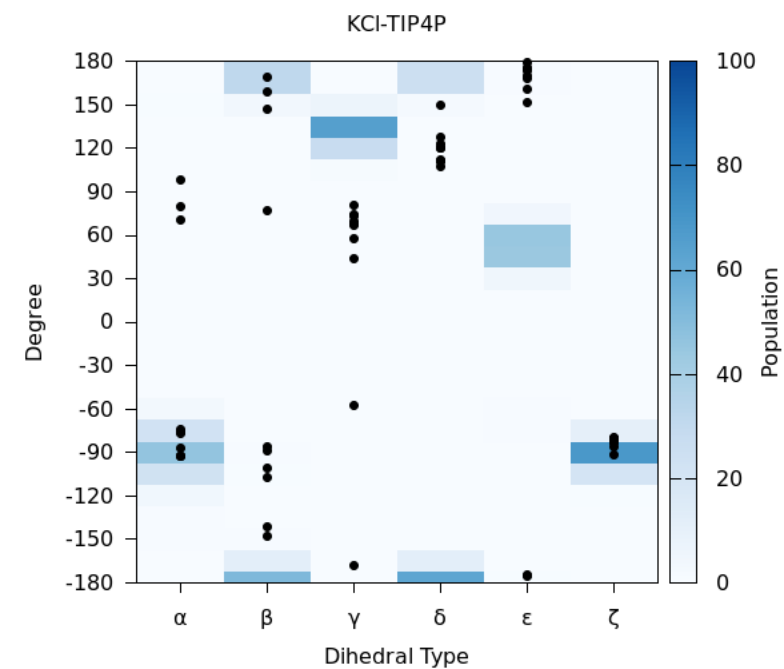

Supplement: Supplementary file 2 — ct2c00291_si_002.zip [file ct2c00291_si_002.zip › Figure S59.pdf]

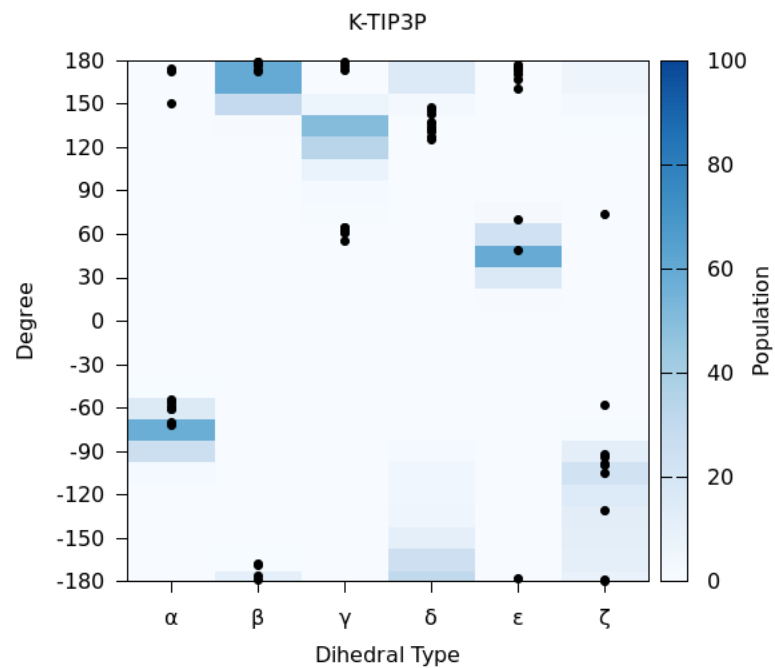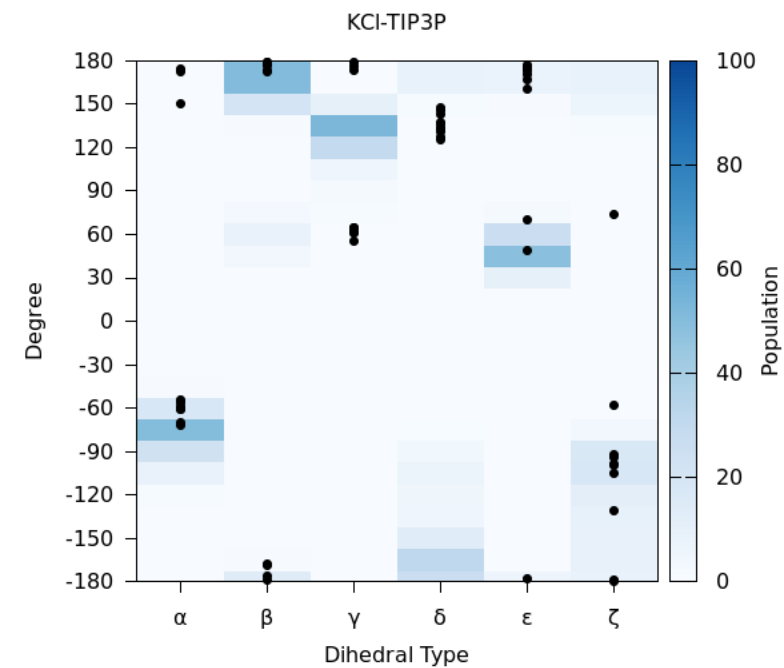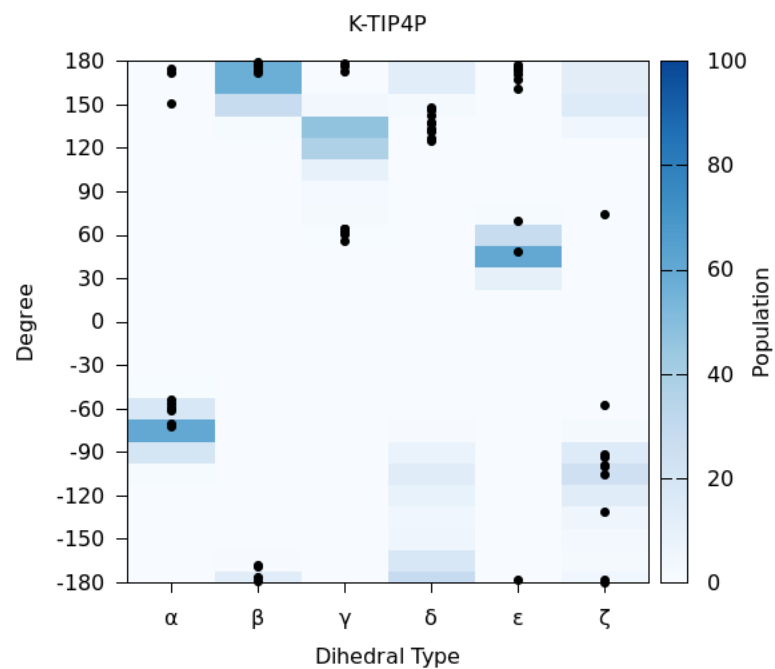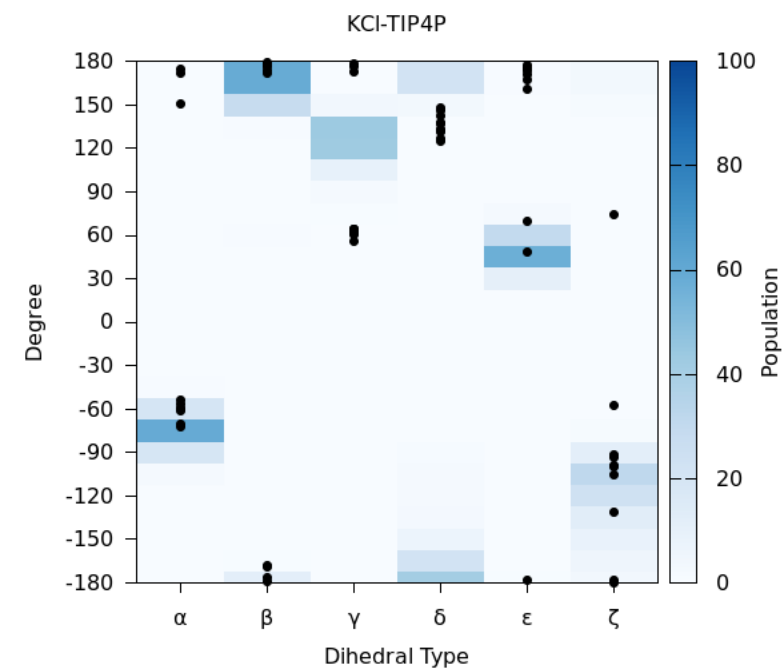

Supplement: Supplementary file 2 — ct2c00291_si_002.zip [file ct2c00291_si_002.zip › Figure S60.pdf]

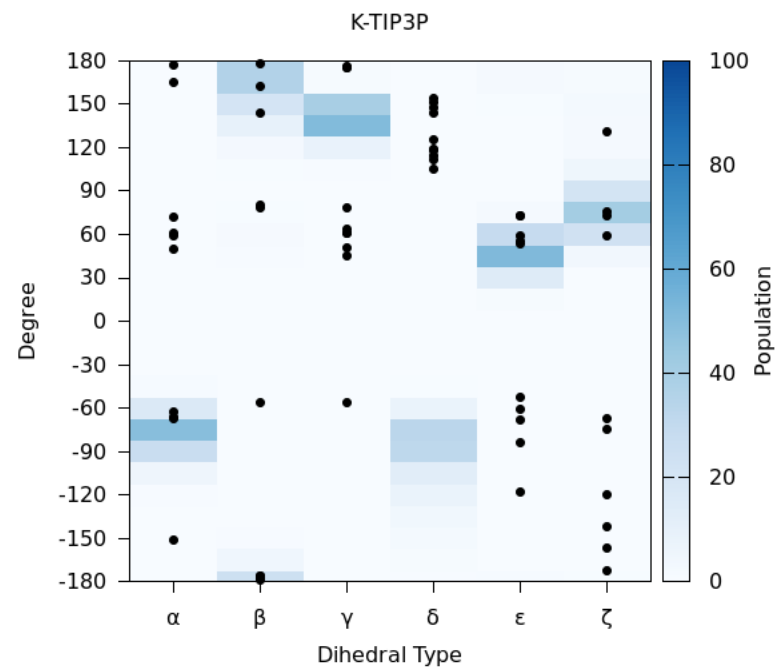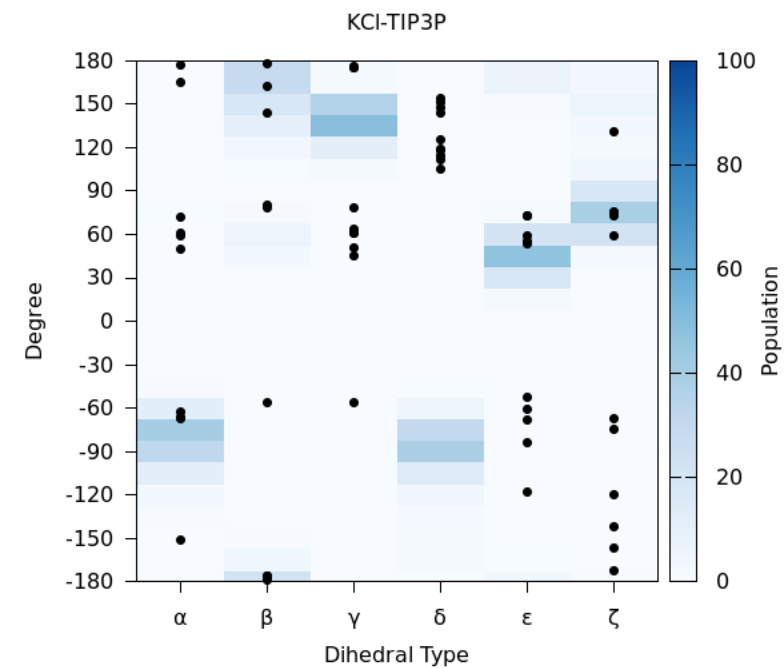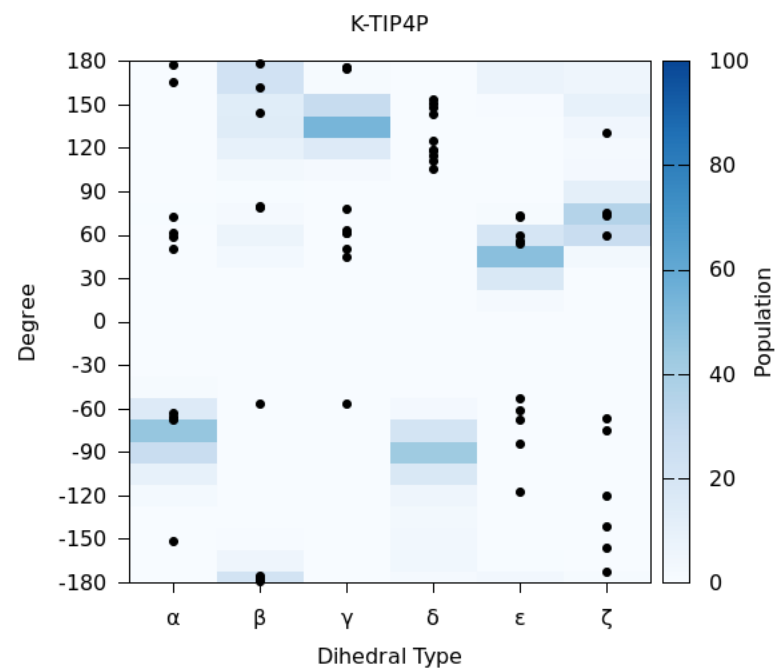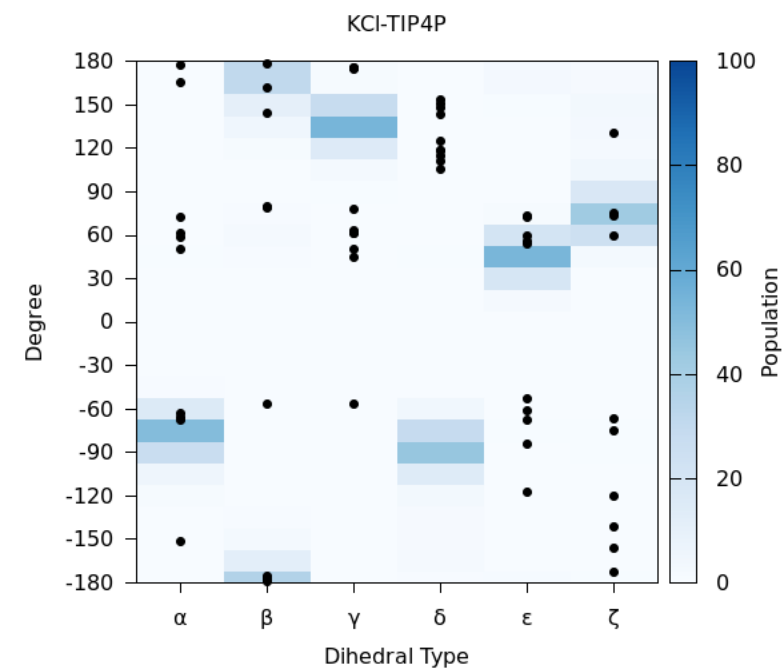

Supplement: Supplementary file 2 — ct2c00291_si_002.zip [file ct2c00291_si_002.zip › Figure S61.pdf]

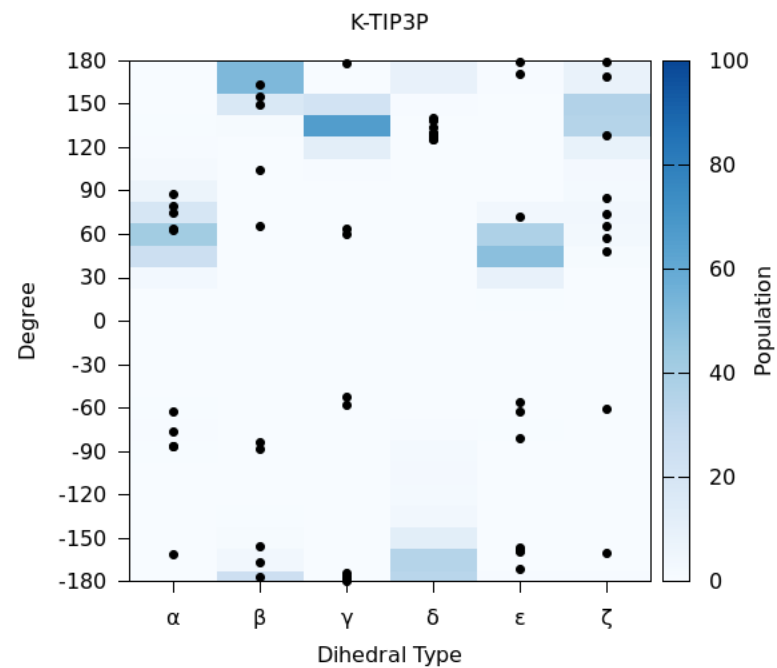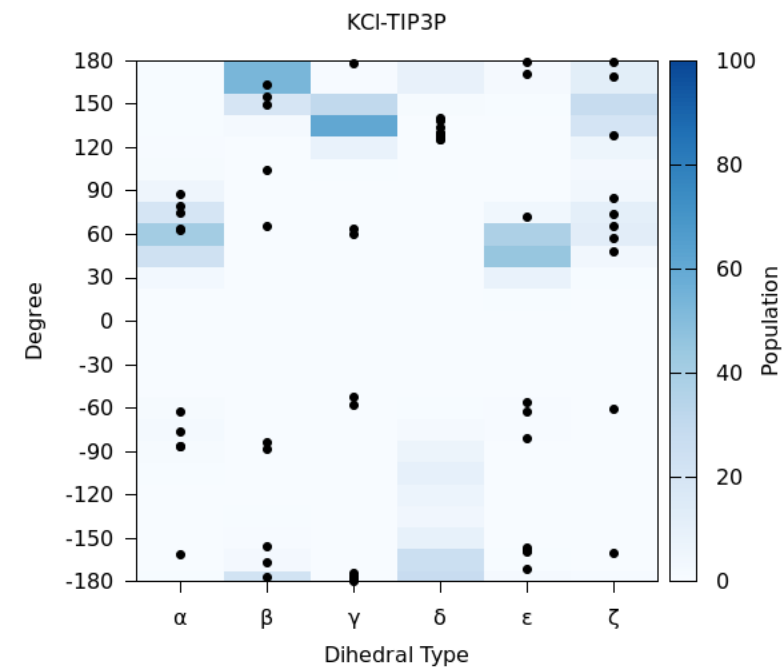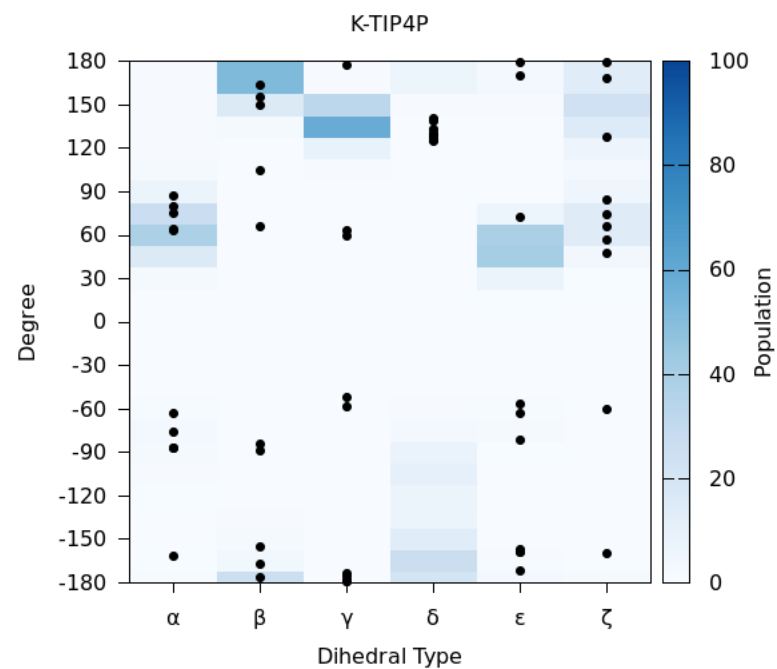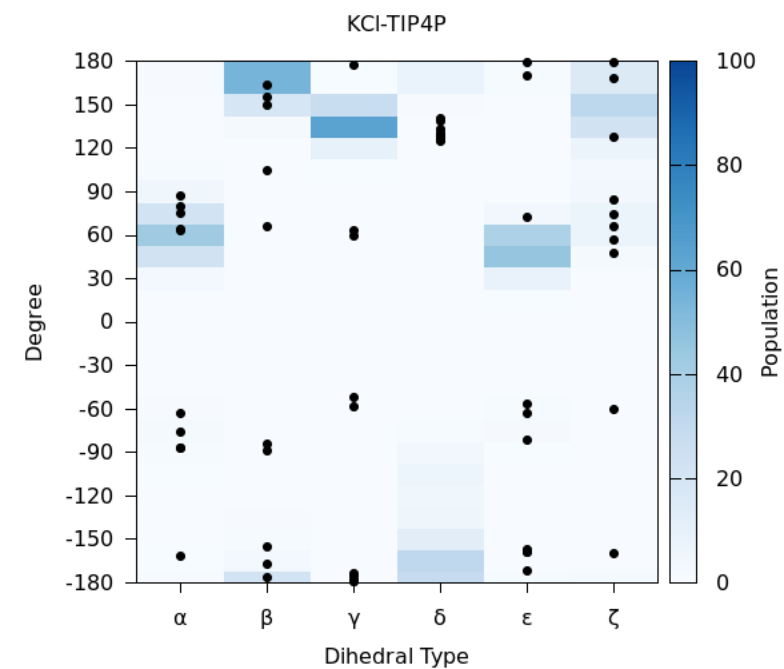

Supplement: Supplementary file 2 — ct2c00291_si_002.zip [file ct2c00291_si_002.zip › Figure S62.pdf]

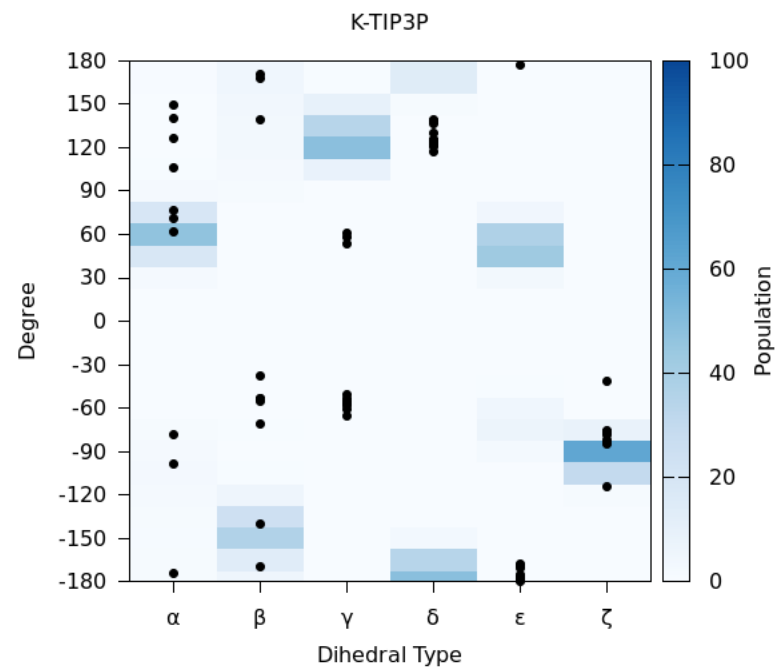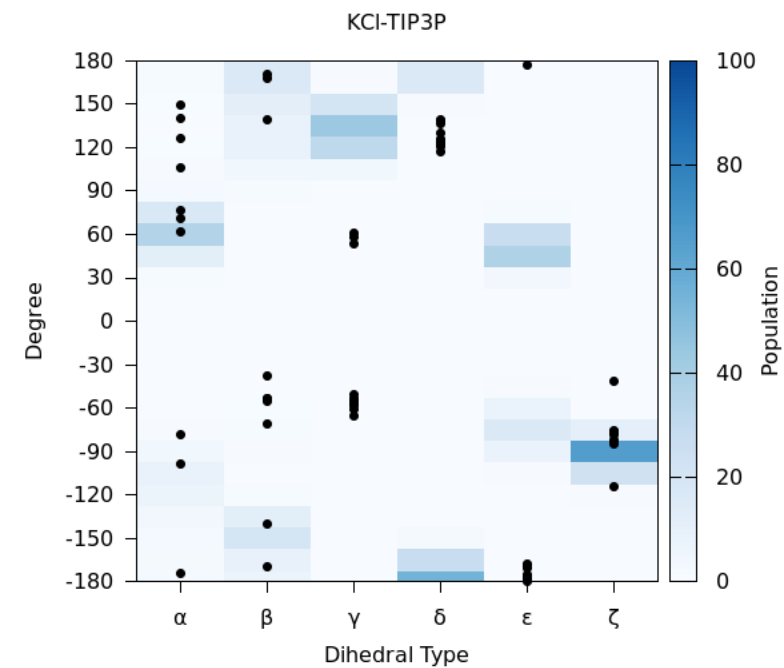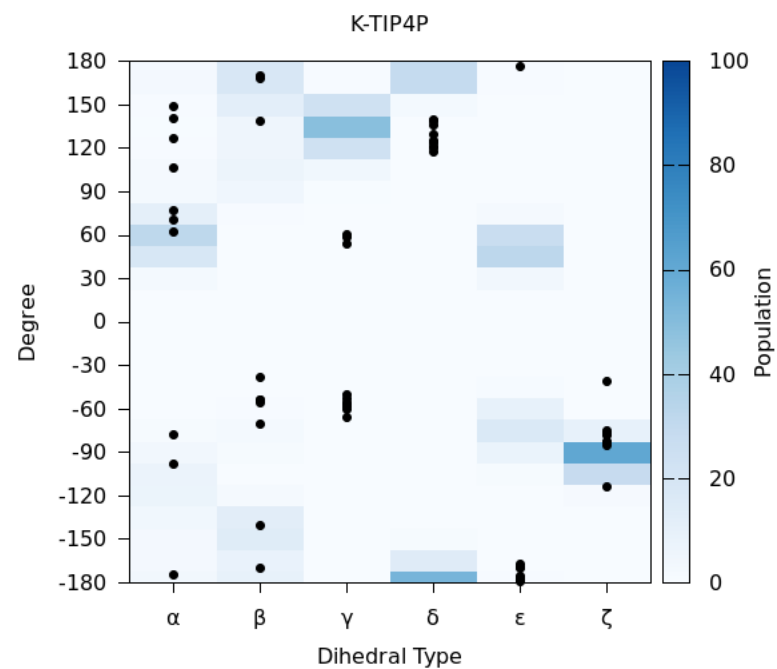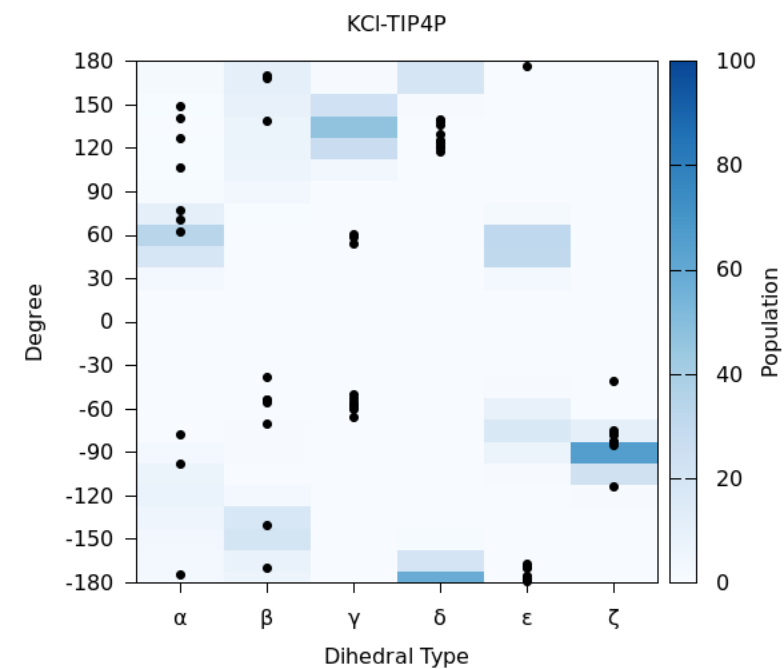

Supplement: Supplementary file 2 — ct2c00291_si_002.zip [file ct2c00291_si_002.zip › Figure S63.pdf]

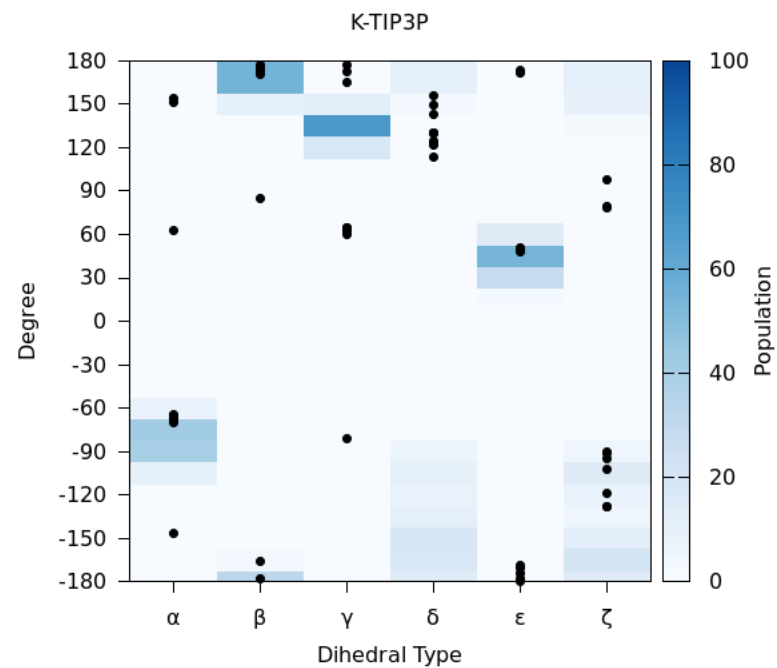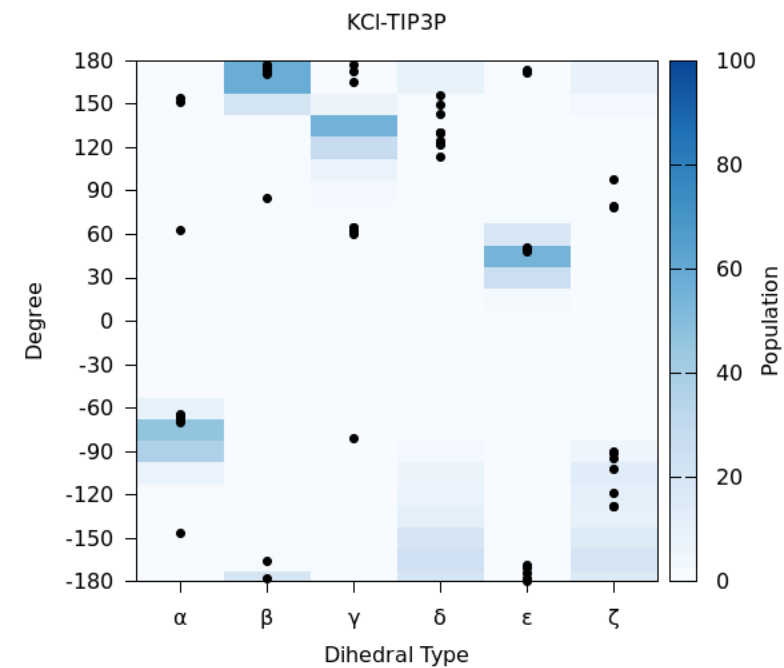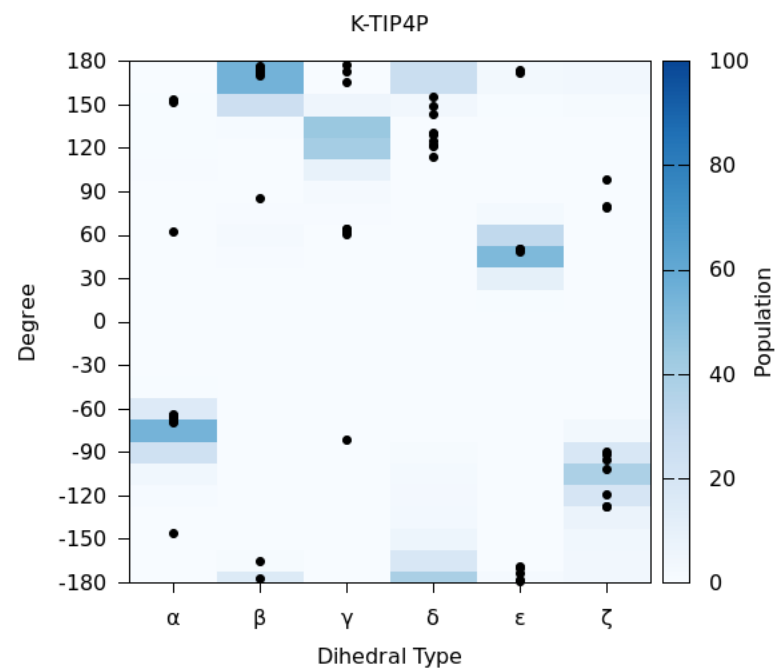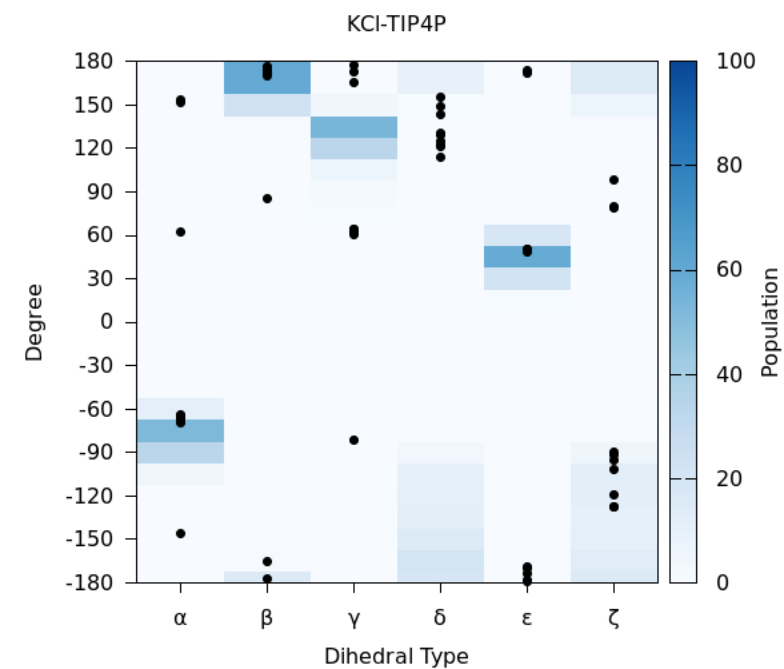

Supplement: Supplementary file 2 — ct2c00291_si_002.zip [file ct2c00291_si_002.zip › Figure S64.pdf]

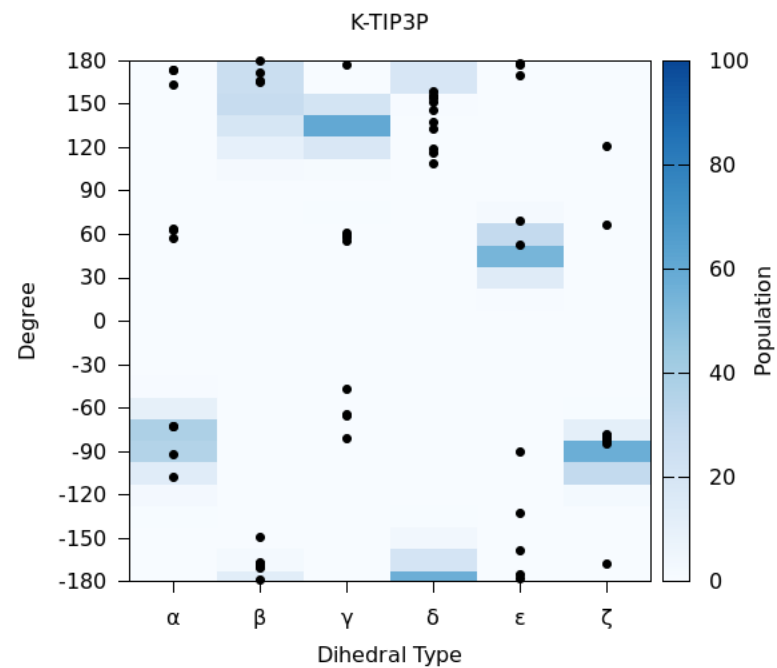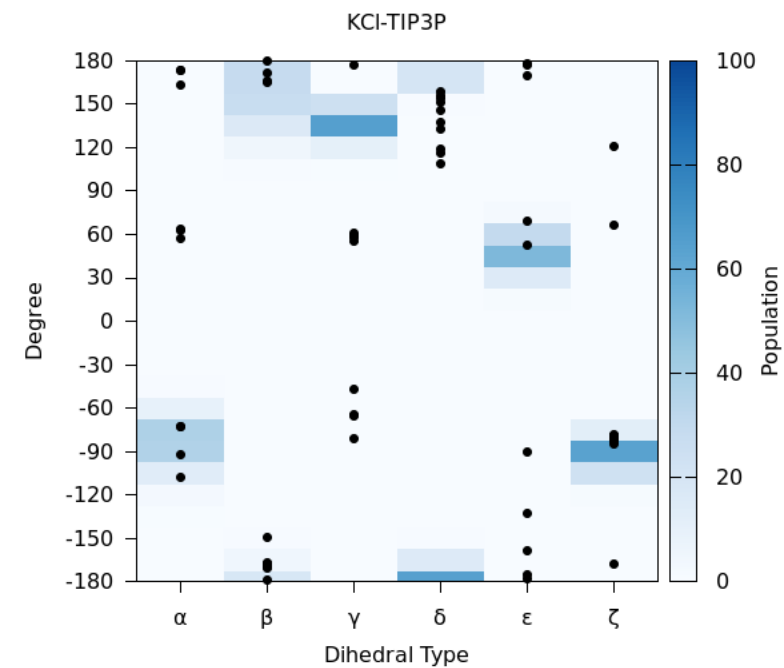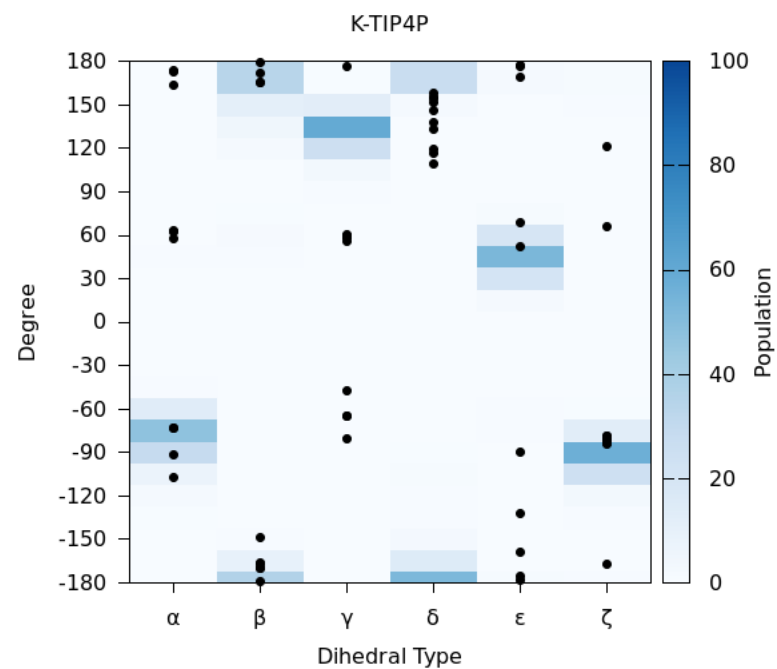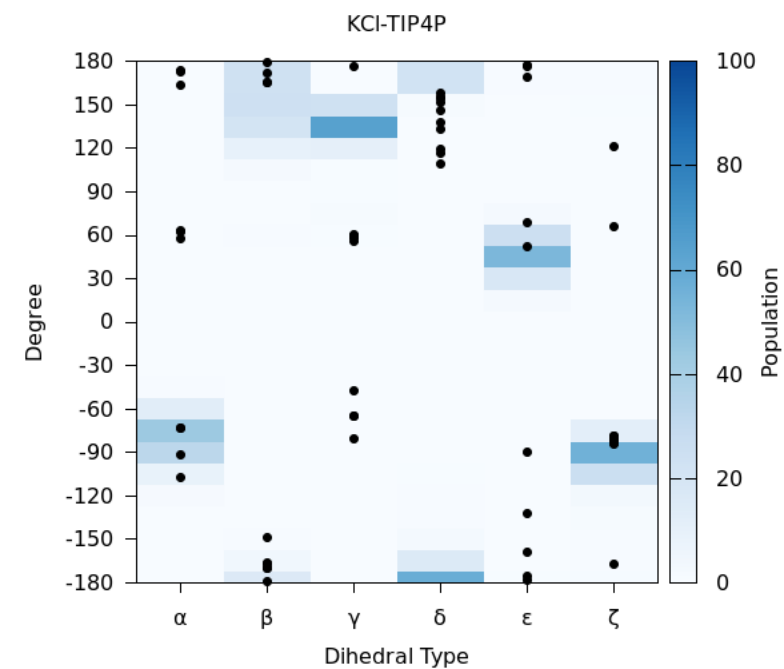

Supplement: Supplementary file 2 — ct2c00291_si_002.zip [file ct2c00291_si_002.zip › Figure S65.pdf]

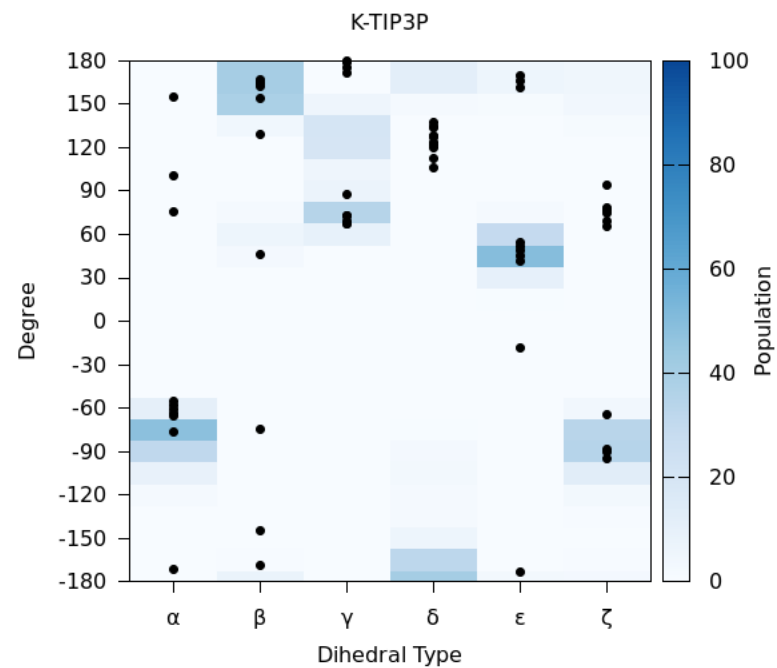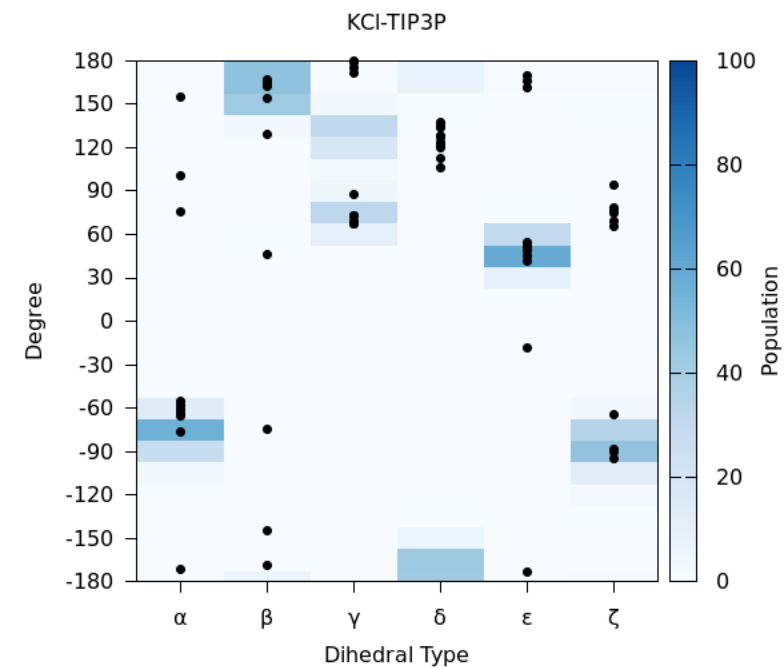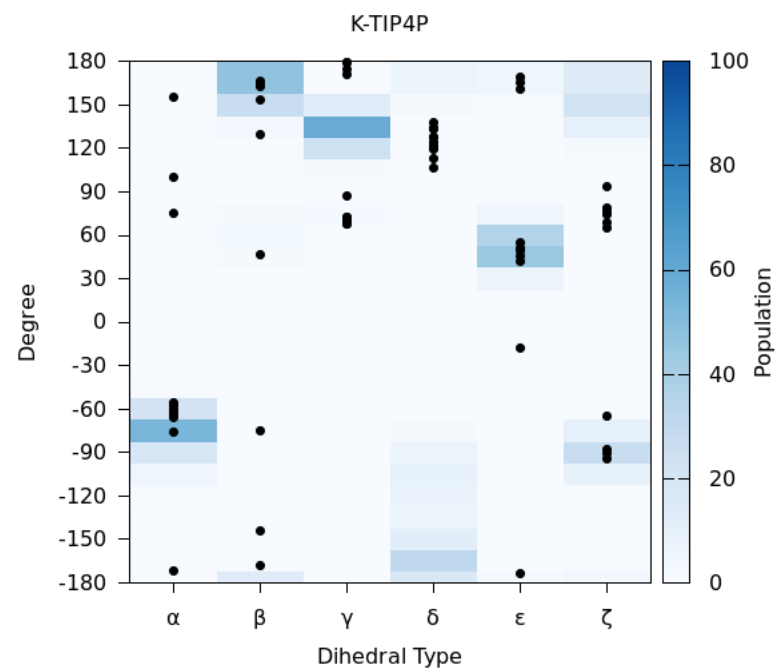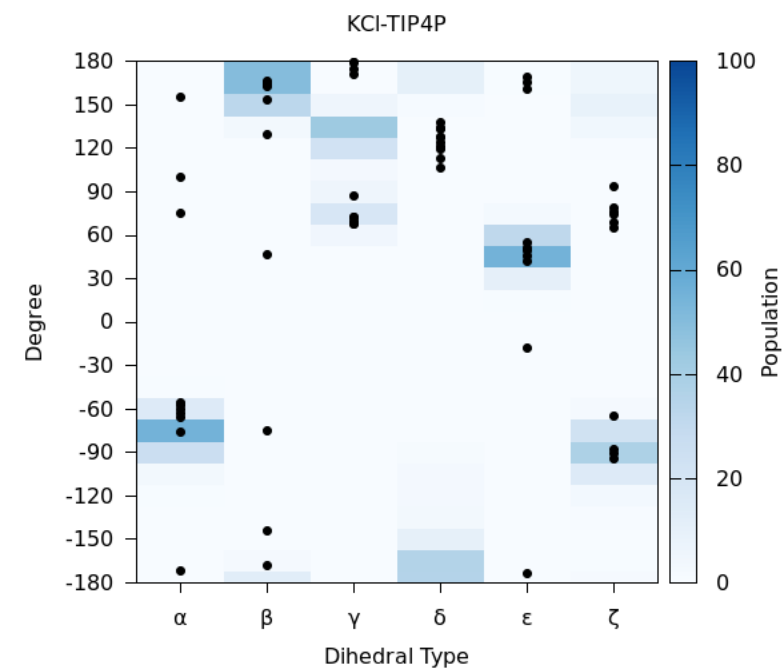

Supplement: Supplementary file 2 — ct2c00291_si_002.zip [file ct2c00291_si_002.zip › Figure S66.pdf]

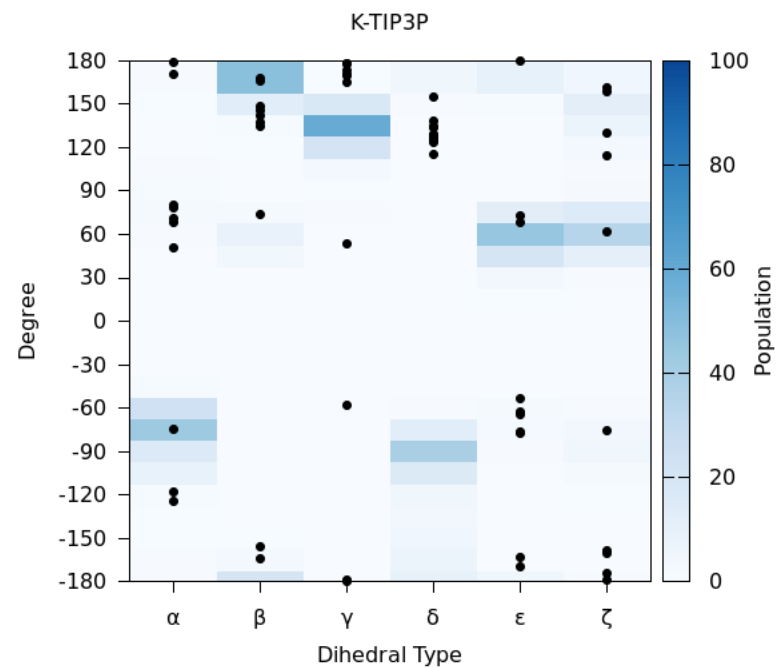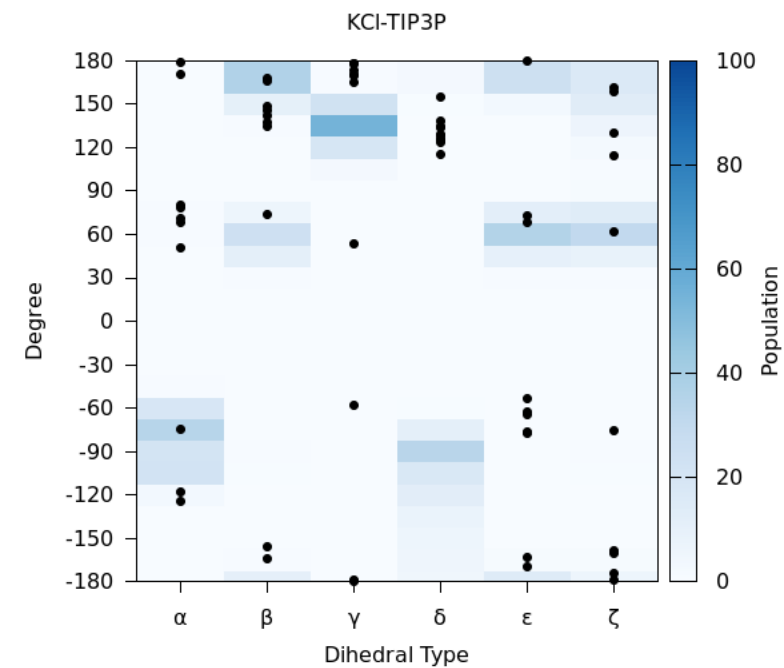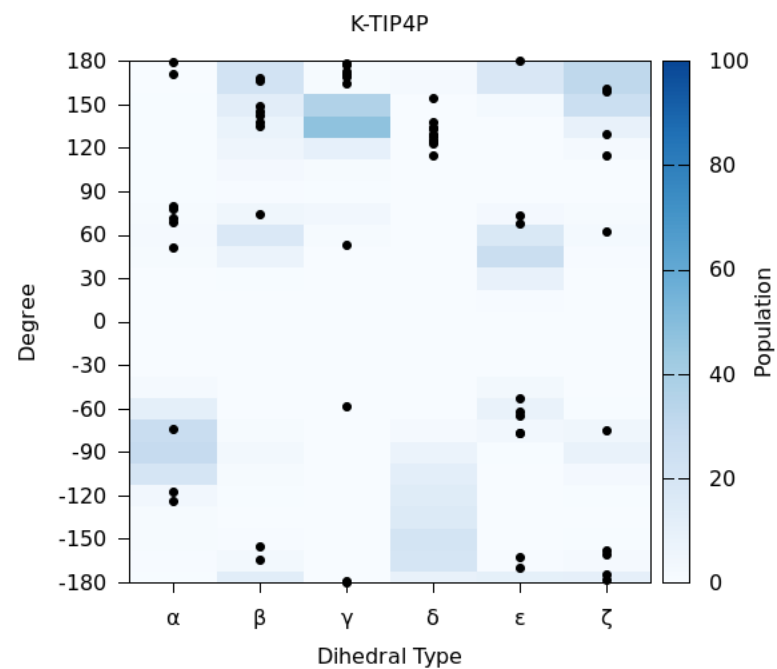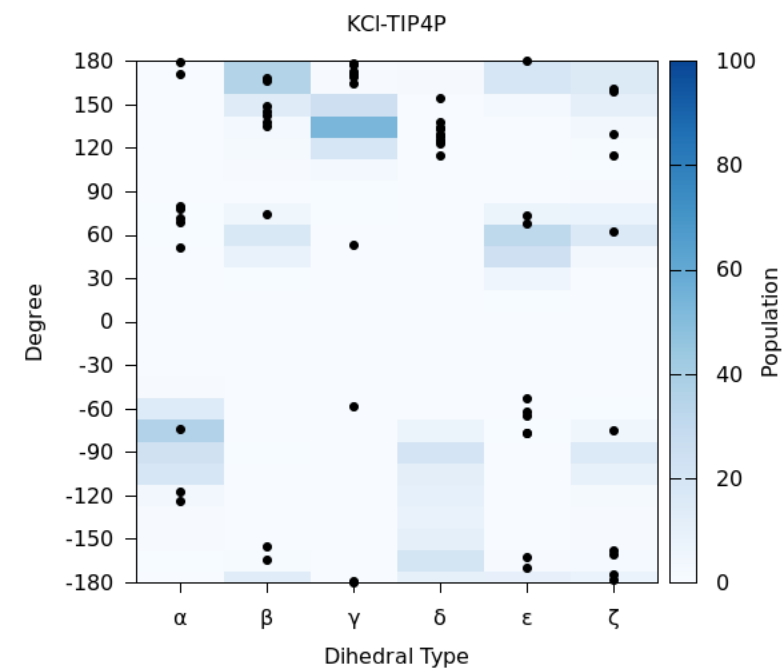

Supplement: Supplementary file 2 — ct2c00291_si_002.zip [file ct2c00291_si_002.zip › Figure S67.pdf]

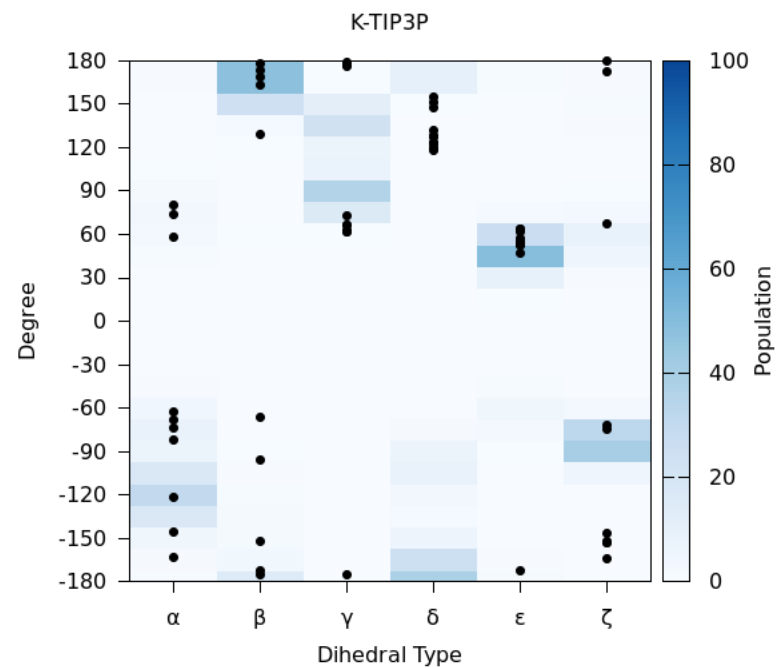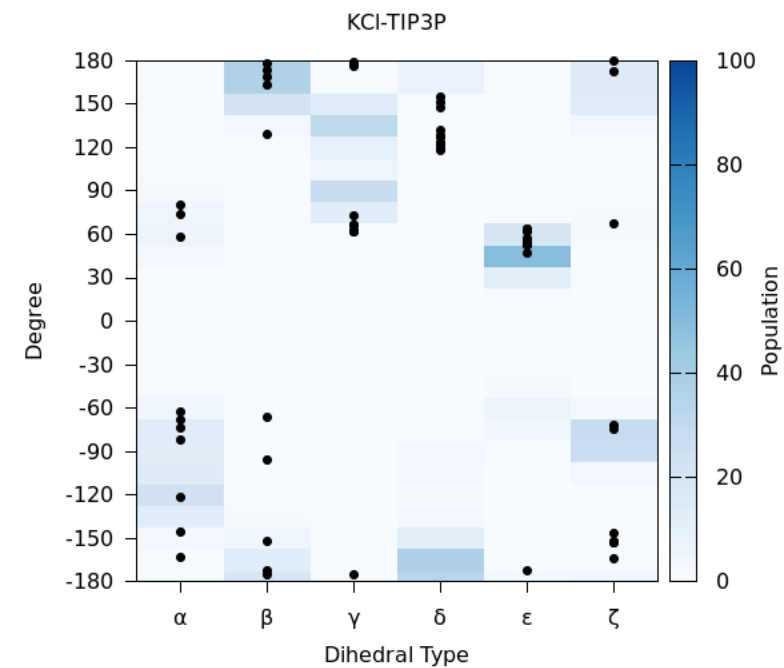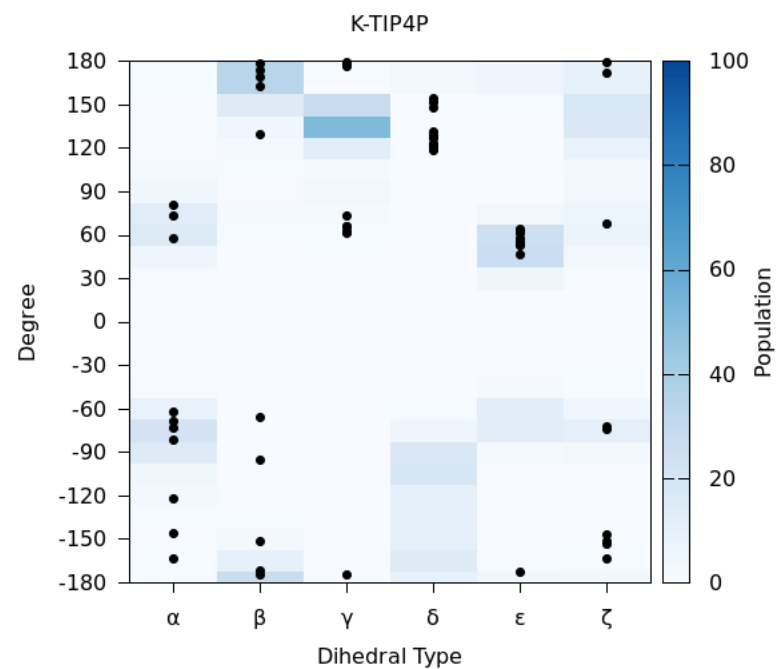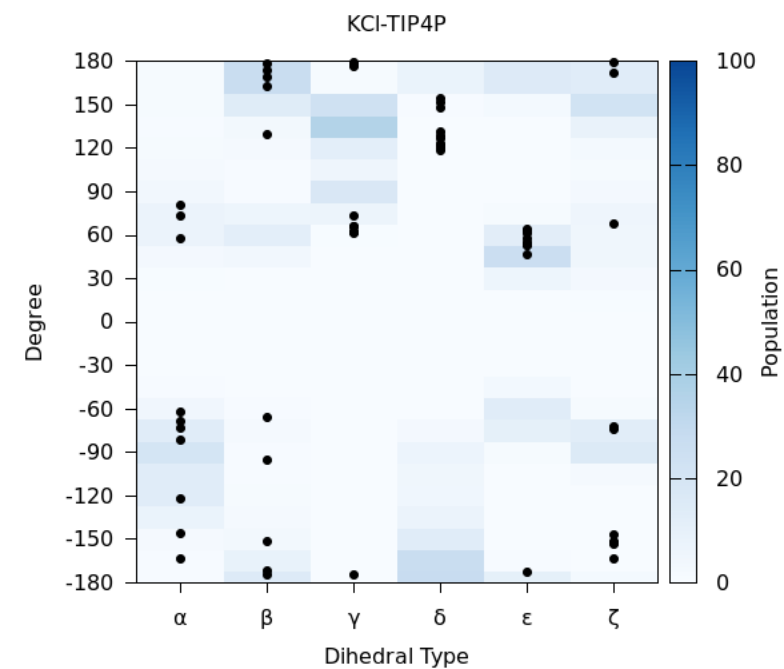

Supplement: Supplementary file 2 — ct2c00291_si_002.zip [file ct2c00291_si_002.zip › Figure S68.pdf]

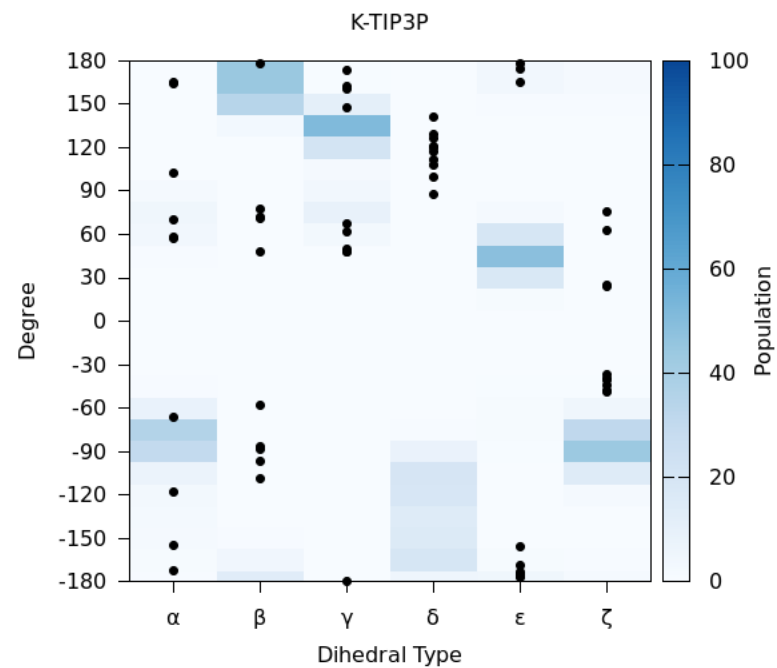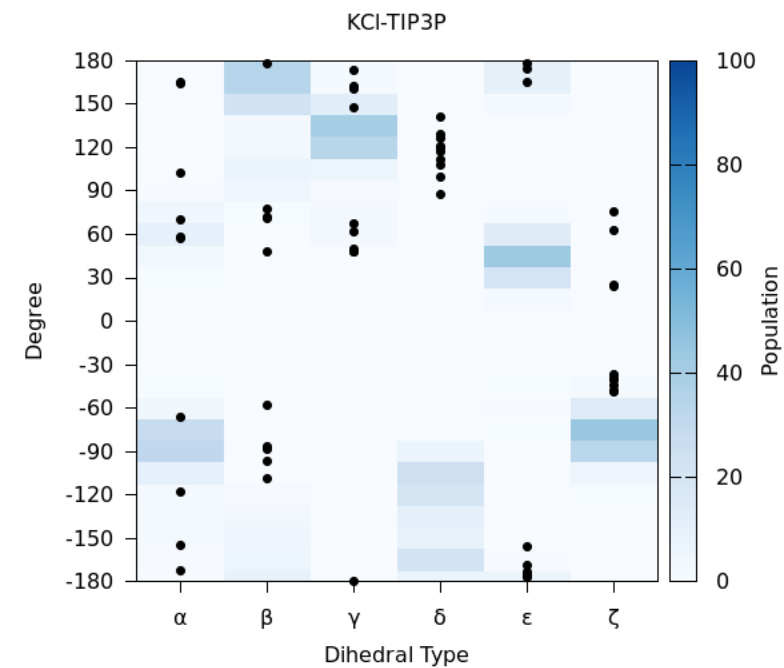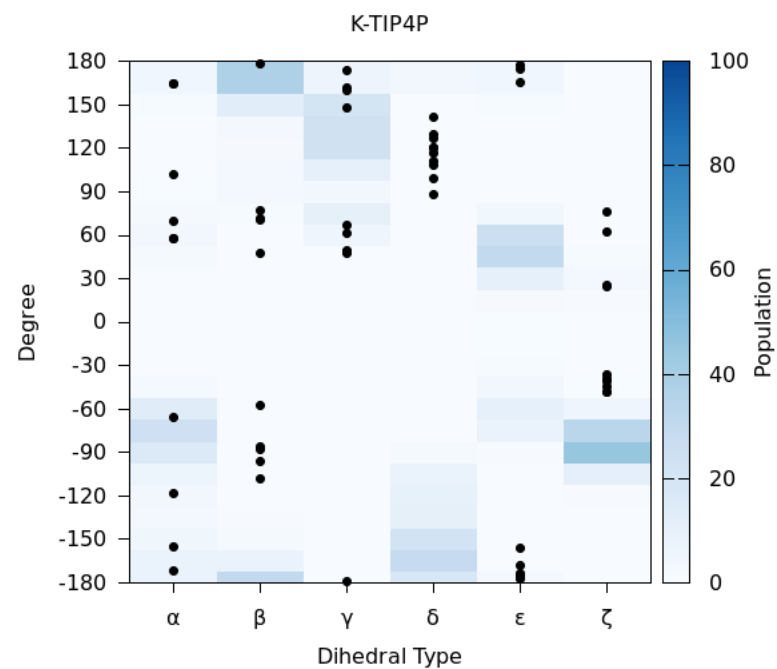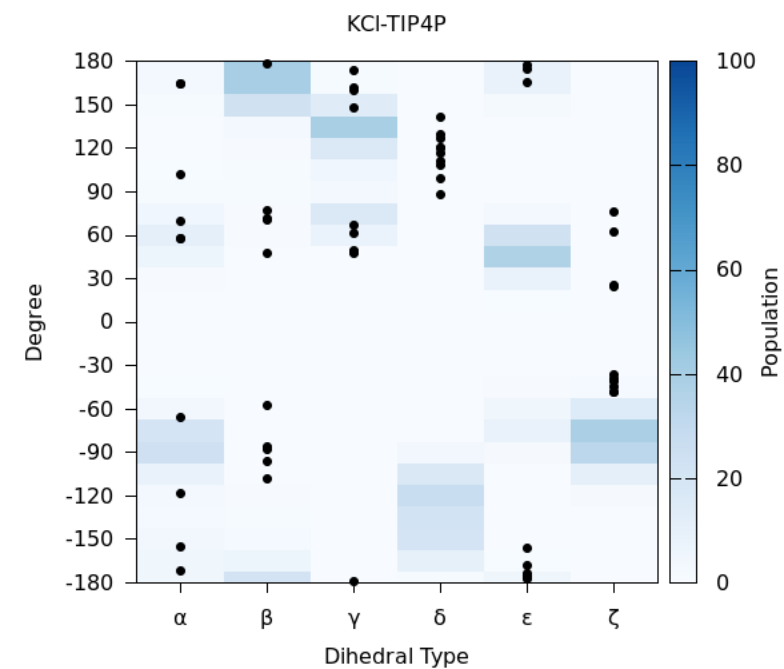

Supplement: Supplementary file 2 — ct2c00291_si_002.zip [file ct2c00291_si_002.zip › Figure S69.pdf]

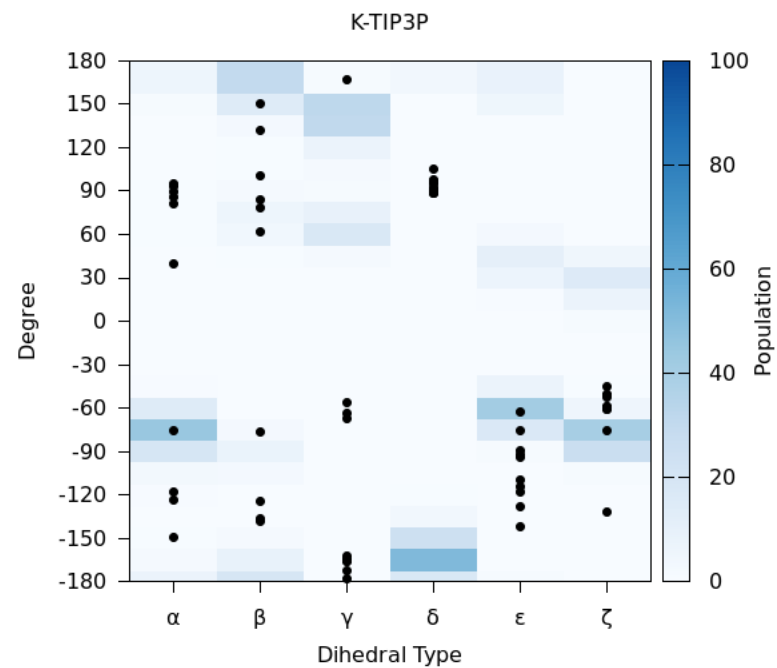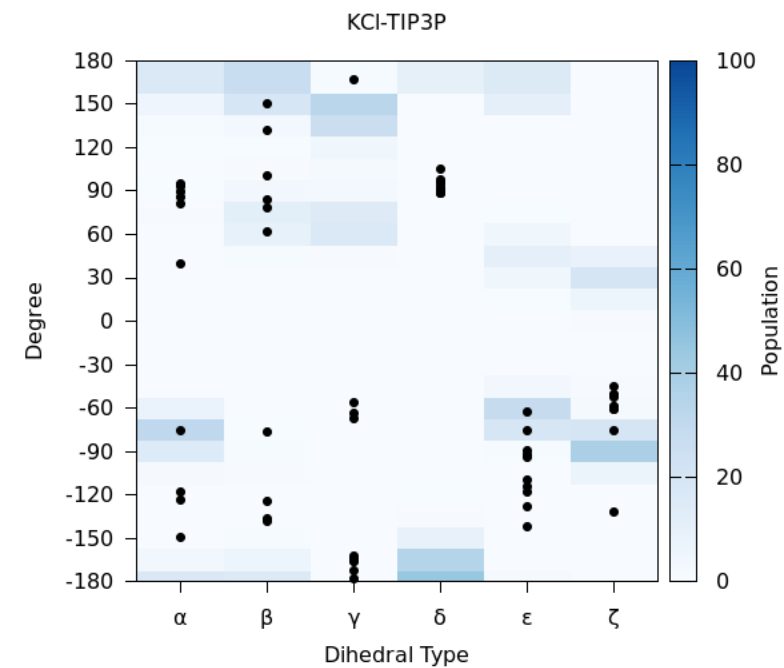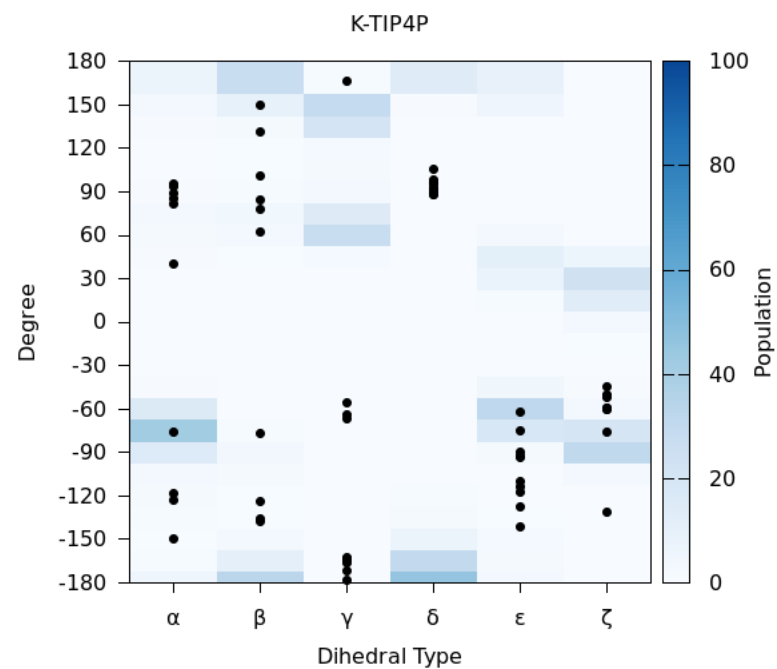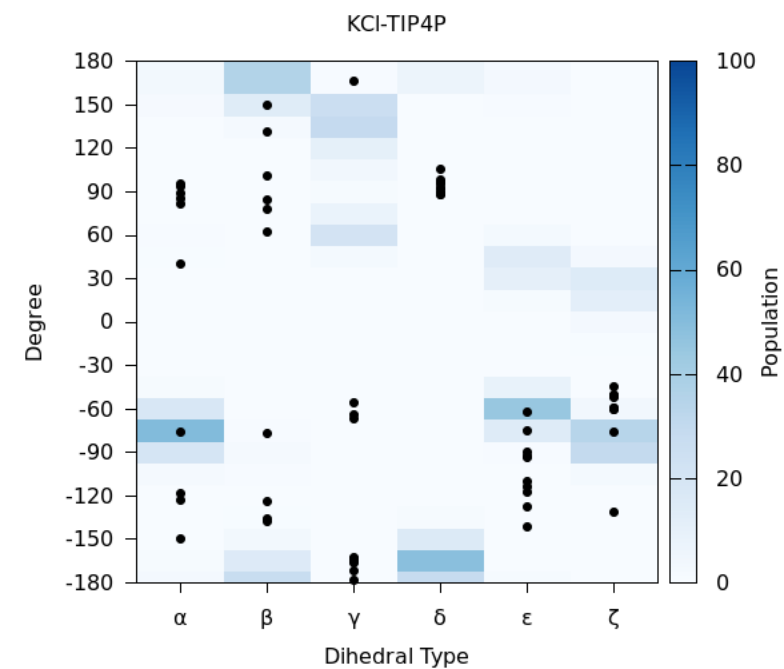

Supplement: Supplementary file 2 — ct2c00291_si_002.zip [file ct2c00291_si_002.zip › Figure S70.pdf]

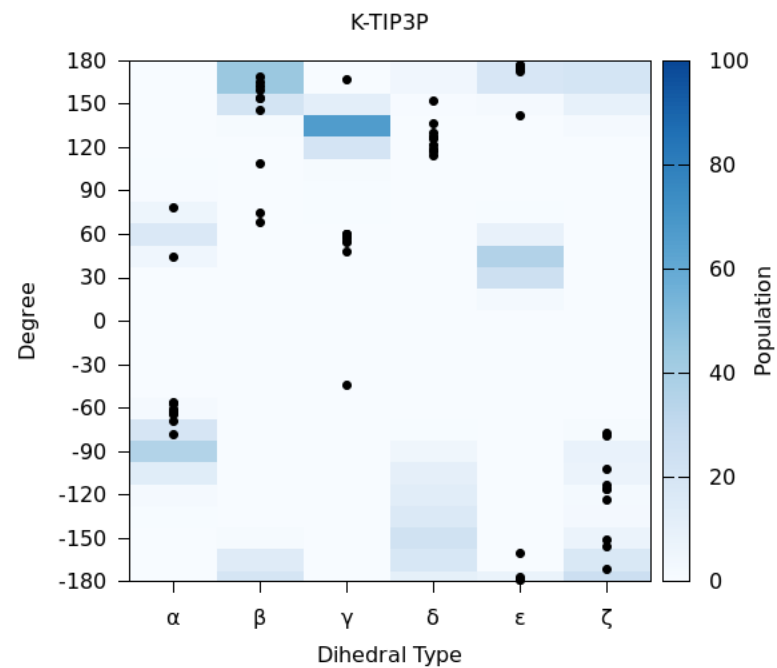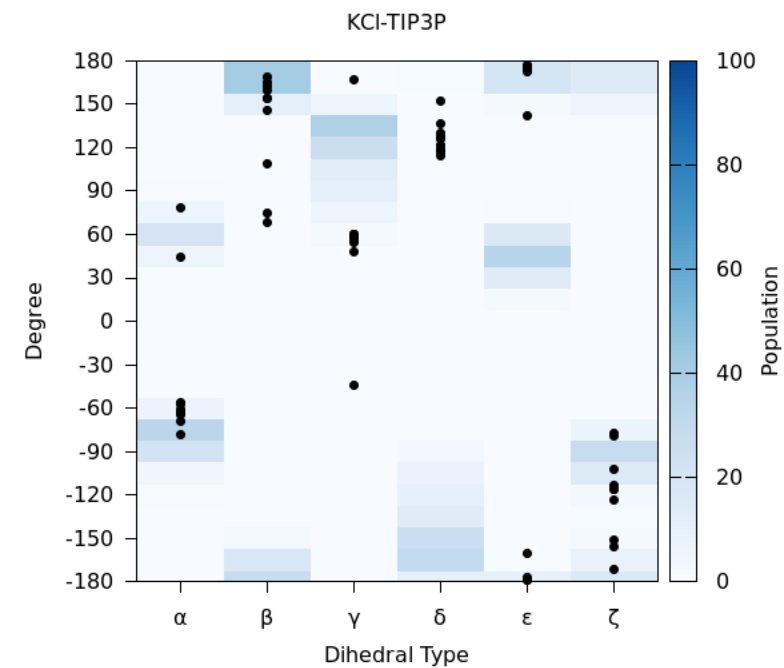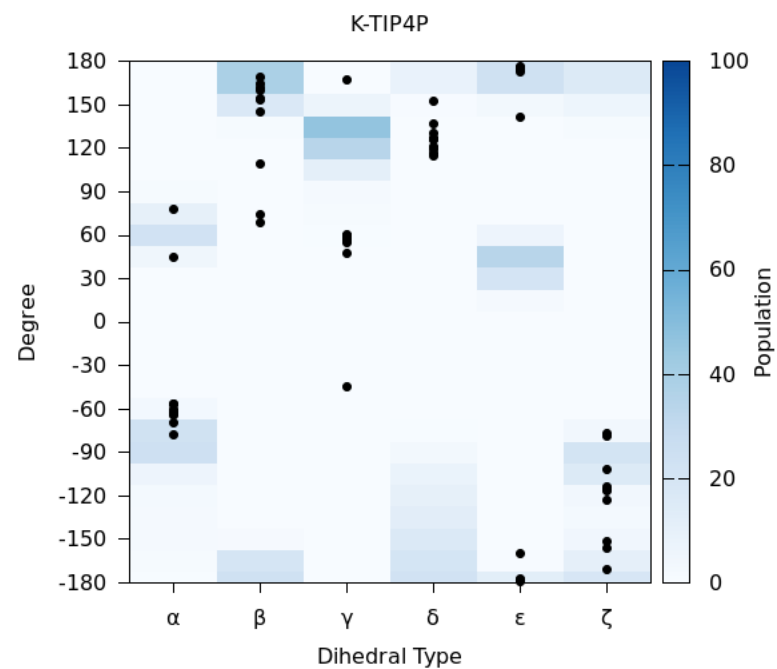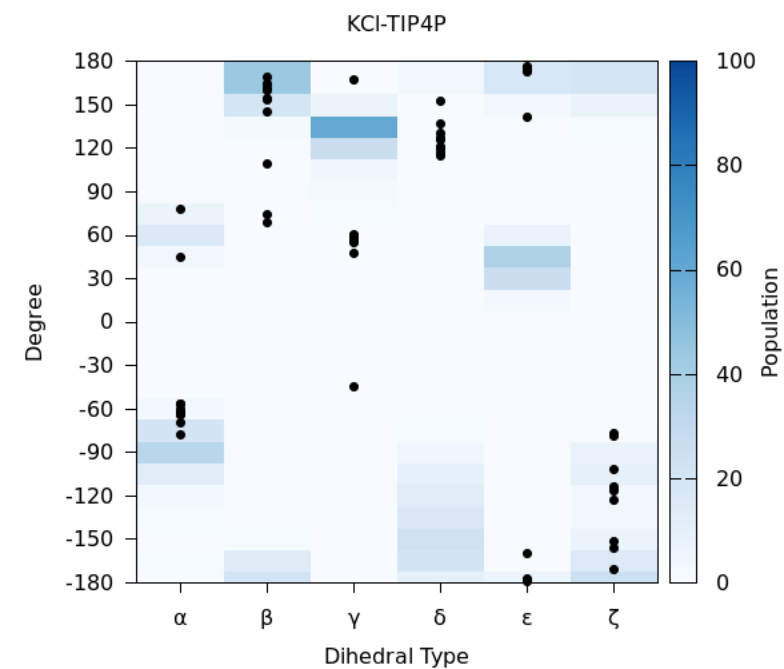

Supplement: Supplementary file 2 — ct2c00291_si_002.zip [file ct2c00291_si_002.zip › Figure S71.pdf]

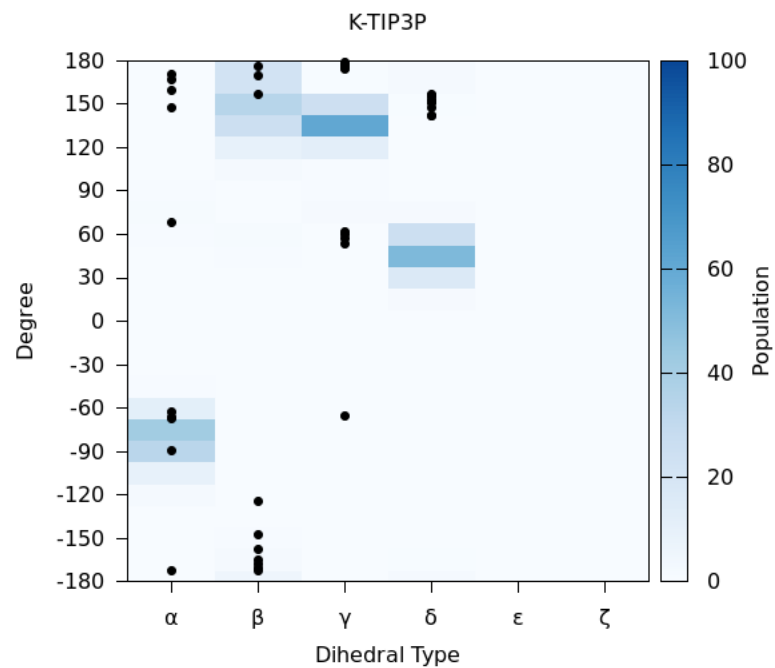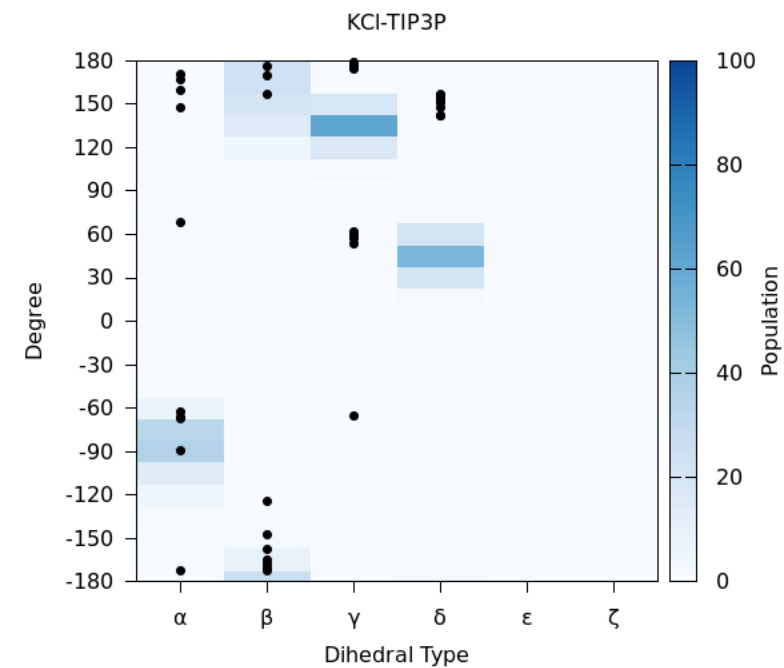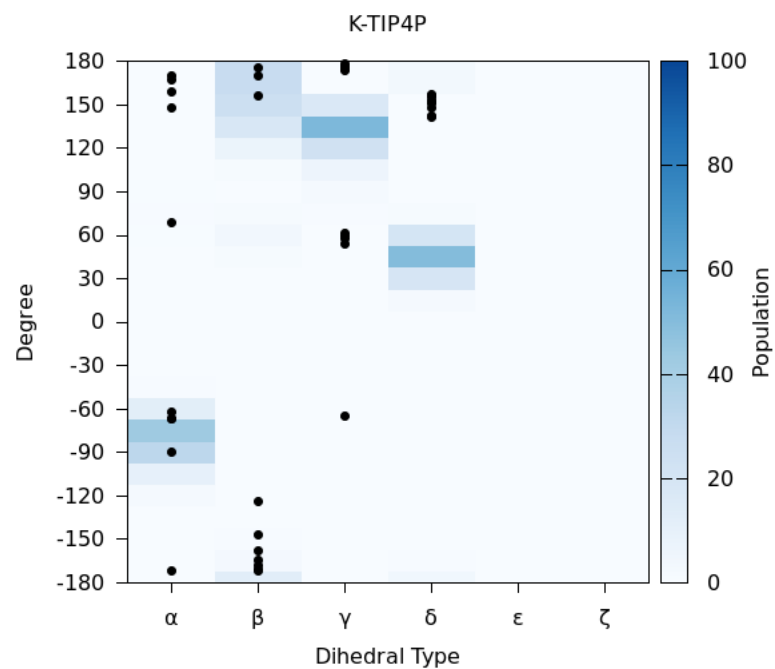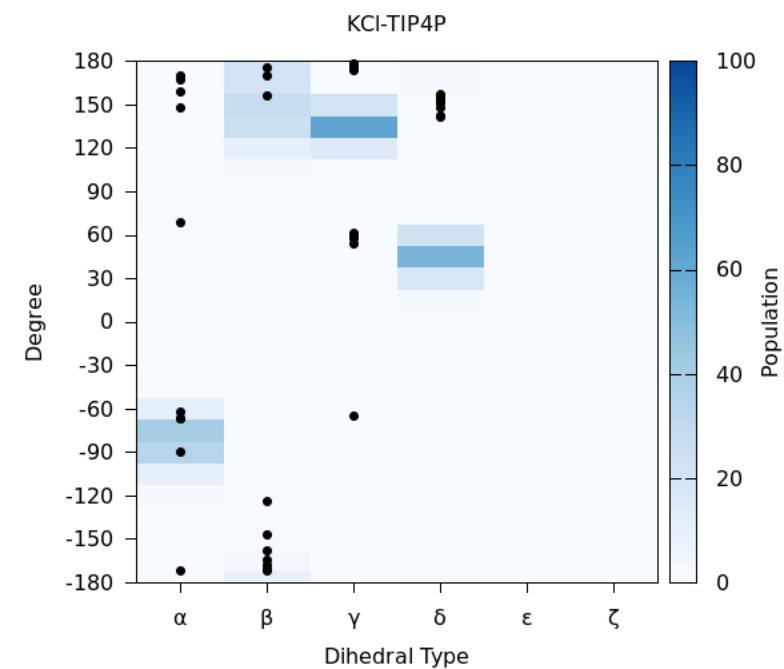

Supplement: Supplementary file 2 — ct2c00291_si_002.zip [file ct2c00291_si_002.zip › Figure S72.pdf]
